# Supplementary material for: Characterizing early drug resistance-related events using geometric ensembles from HIV protease dynamics
Source: Sci Rep. 2018 Dec 18;8:17938. doi: 10.1038/s41598-018-36041-8 (PMC6298995; doi:10.1038/s41598-018-36041-8)
Supplement: Supplementary file 2 — Supplementary Data 1-6 [file 41598_2018_36041_MOESM2_ESM.pdf]

Characterizing early drug resistance-related events using geometric ensembles from HIV protease dynamics

Olivier Sheik Amamuddy[1], Nigel T. Bishop[2] and Özlem Tastan Bishop[1*]

[1] Research Unit in Bioinformatics (RUBi), Department of Biochemistry and Microbiology, Rhodes University, Grahamstown 6140, South Africa.
[2] Department of Mathematics (Pure & Applied), Rhodes University,Grahamstown 6140, South Africa.

[*] o.tastanbishop@ru.ac.za

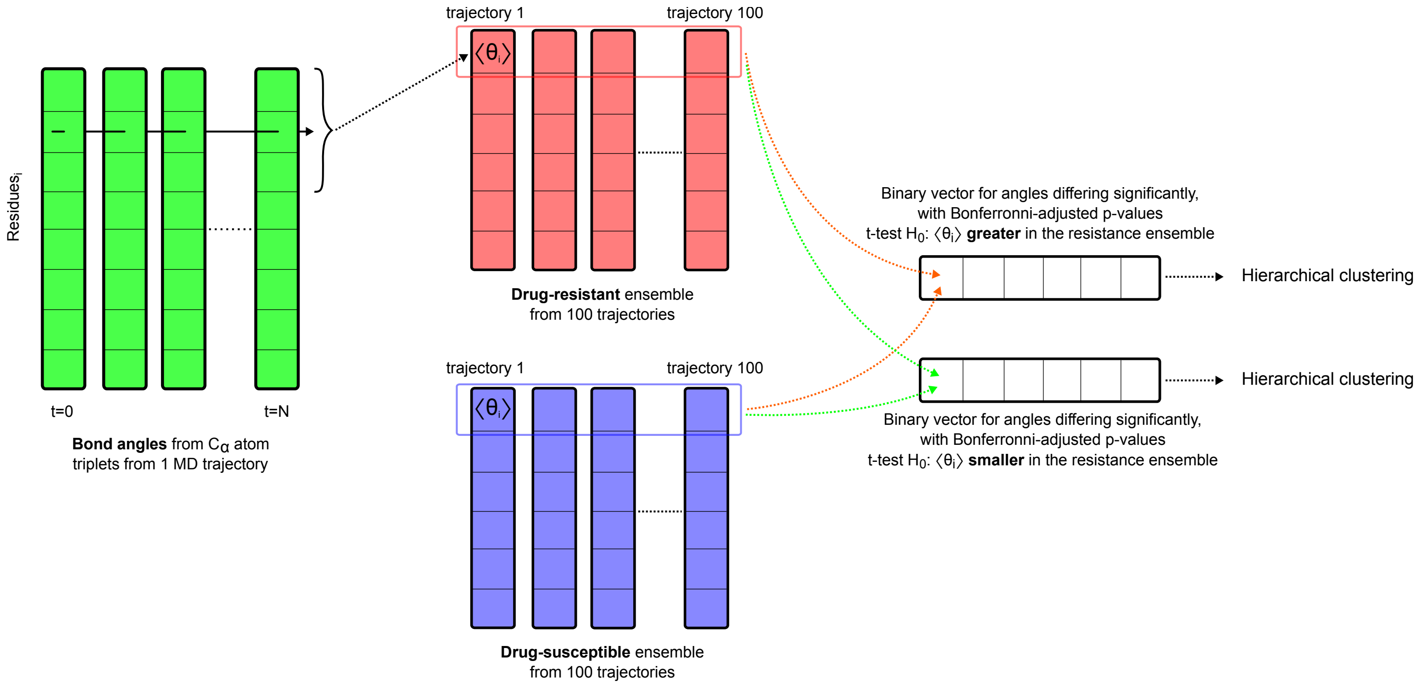

Supplementary Figure 1: Experimental layout for evaluating t-tests from C_α angles for residue triplets from MD trajectories prior to hierarchical clustering.

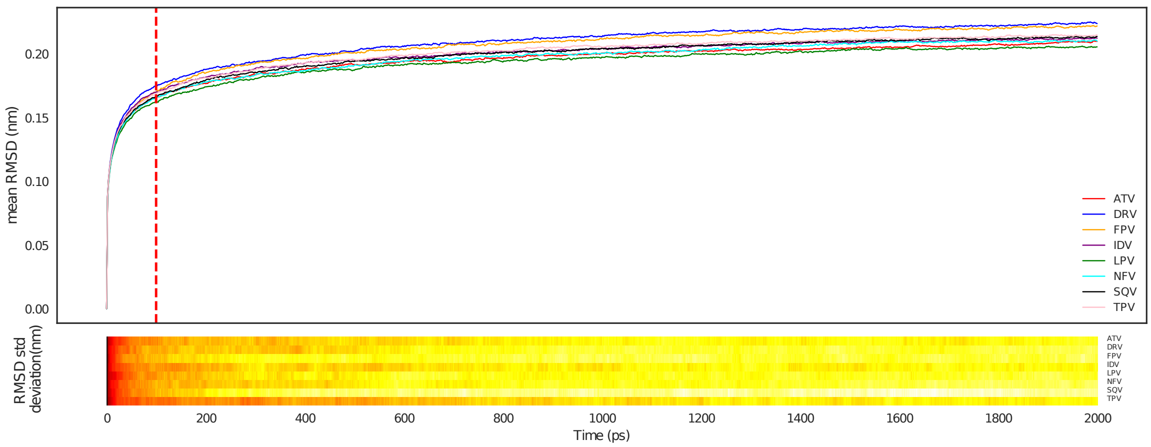

Supplementary Figure S2: Mean Cα RMSD values obtained across 200 complexes over the duration of the MD simulations for each of ATV, DRV, FPV, IDV, LPV, NFV, SQV and TPV. The red dotted line depicts the 100ps region beyond which the MD runs stabilize. At the botom, the heat map shows the standard deviations of the RMSD values for the same drugs, with higher values colored red, through yellow and white, which represent lower values.

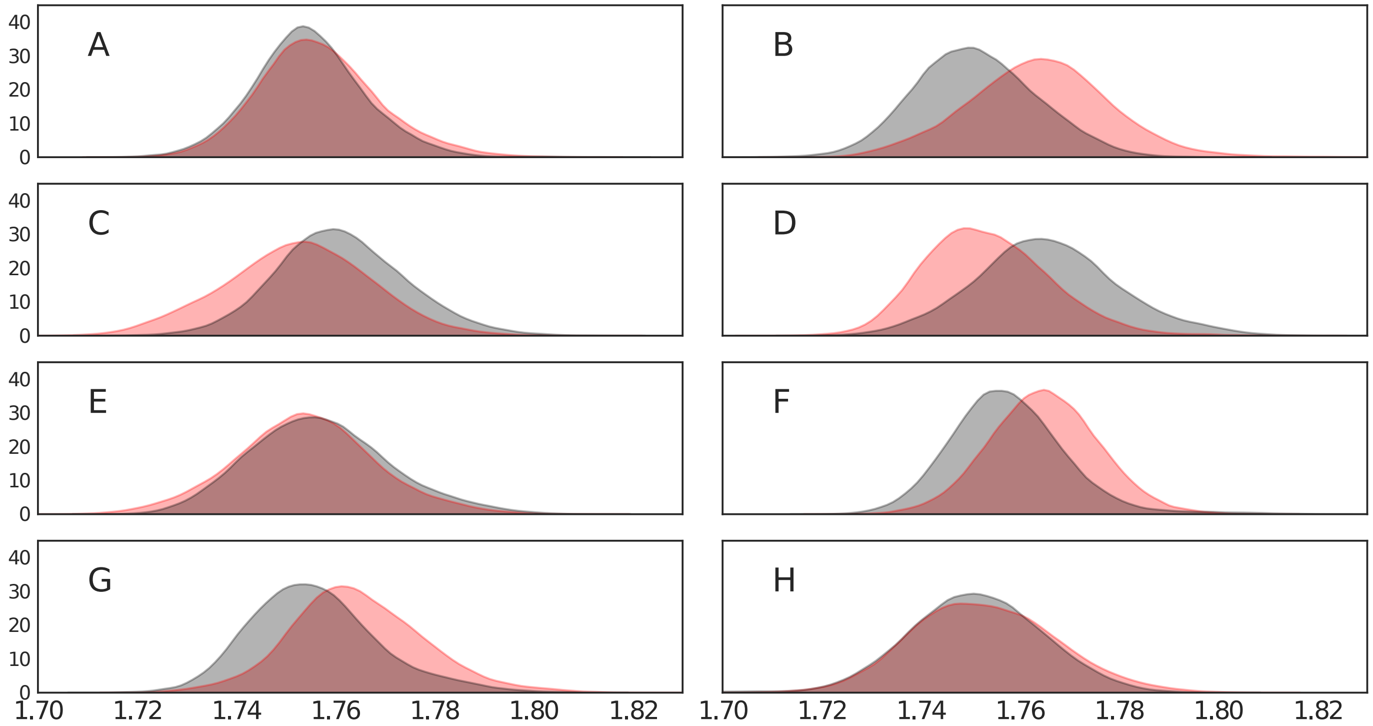

Supplementary Figure S3: Density distributions for the radii of gyration for each HIV protease drug resistance ensemble for (A) ATV, (B) DRV, (C) FPV, (D) IDV, (E) NFV, (F) NFV, (G) SQV and (H) TPV, obtained from the replicate molecular dynamics simulations. Drug resistance ensembles are shaded in red while the susceptible ones are in grey.

**Supplementary Table S4:** Mutations present in each drug resistance ensemble (according to the 2017 update)
**ATV**
4S, **10IVF,** 11LI, 12I, 13VM, 14R, 15V, 16A, 19IV, 20RIVTM, 21D, 22V, 24IM, **32I**, **33FV**, **34Q**F, 35DNG, 36IV, 37DS, 41K, 43IT, **46LI**, 47V, **48QV**, 51A, **53L**, **54LVM**, 55RN, 57K, 58E, **60E**, 61HDN, **62V**, 63P, **64VM**, 66V, 67YFE, 69RYQ, 70E, **71IV**, 72LTVMF, **73STA**, 74PS, 75I, 76V, 77I, 79SA, 82LSTA, 83D, 84VA, 89V, **90M**, 91S, 92K, **93LM**, 95F, 96S
**DRV**
10IFV, **11LI**, 12KPA, 13V, 14R, 15V, 16A, 19QV, 20RTVM, 24M, 30N, **32I**, **33F**, 34Q, 35DG, 36IVT, 37QDT, 43IQT, 46LI, 47V, 51A, 53L, 54LM, 55RN, 57KG, 58E, 60E, 62V, 63P, 64VM, 66V, 67WY, 69Q, 71IV, 72LVM, 73STA, 77IT, 79S, 82LA, 84V, 85L, 88D, **89V**, 90M, 91S, 92K, 93L
**FPV**
**10IVF**, 11I, 12KDPV, 13V, 14RT, 15V, 16A, 19QTI, 20RITVM, 24M, 30N, **32I**, 33F, 35KDNG, 36LI, 37HDQTP, 41K, 43IT, **46LI**, **47V**, 48Q, 53L, **54LVM**, 55RN, 57KG, 58E, 60E, 61DN, 62V, 63P, 64LV, 66FV, 67YGFE, 68E, 69KLQ, 70TE, 71LIVT, 72LTM, **73S**T, 74PS, **76V**, 77IT, 79SA, **82LTA**, 84VA, 87G, 88D, 89VM, **90M**, 91S, 92KR, 93L, 95F
**IDV**
4A, 7R, **10IFV**, 11I, 12KP, 13VM, 15V, 19QIPT, **20R**IT, 21D, 22V, 24M, **32I**, 33IF, 34TQ, 35DNG, **36LI**, 37SDE, 41K, 43TE, 46LI, 47V, 48LVM, 50V, 53LY, **54TV**SACM, 57K, 58E, 60NE, 61HNE, 62V, 63P, 64VM, 66VF, 67FE, 68E, 69RYQ, **71TV**, 72LTV, **73CSTA**, 74DS, 75I, **76V**, **77I**, 82SFTA, 83D, 84VA, 85V, 89V, **90M**, 92K, 93L
**LPV**
**10IFV**, 11LI, 12KP, 13V, 14R, 15V, 16A, 19IPQV, **20RTVM**, **24I**, 30N, 32I, **33FV**, 34TQ, 35DAG, 36I, 37EDQTS, 41K, 43QITE, 45R, **46LI**, 47VA, 48V, **50V**, 51A, **53L**, **54LTVMS**, 55RN, 57K, 58E, 60E, 61HE, 62V, **63P**, 64VM, 65D, 66VF, 67Y, 68E, 69Y, 70E, **71IVT**, 72TVM, **73**CSTA, 74PAS, 75I, 76V, 77IT, 79S, 82SFTAI, **84V**, 85LV, 87G, 88D, 89TV, **90M**, 91S, 92K, 93L, 95F
**NFV**
7R, **10IVF**, 11LI, 12IA, 13V, 15V, 16E, 18H, 19I, 20RIVT, 21D, 22V, 24M, 30N, 33IVFM, 35DNG, **36I**, 37DSTE, 38F, 41K, 43TN, 45R, **46LI**, 48M, 53L, 54VM, 55R, 57K, 58E, 60E, 61HNE, 62V, 63PT, 64V, 65D, 66VF, 67W, 68E, 69R, **71TV**I, 72LTV, 73STA, **77I**, **82FA**, 83D, **84V**A, 85V, **88D**, 90M, 93L
**SQV**
**10I**F, 11LI, 12IP, 13VM, 15V, 18H, 19I, 20RIVT, 21D, 22V, 24M, 32I, 33IVF, 34D, 35DNG, 36IV, 37DS, 41K, 43T, 46LI, 48QV, 53L, **54LV**M, 55R, 57K, 58E, 60E, 61HN, **62V**, 63PE, 64V, 66FV, 67WFE, 69Q, **71TV**I, 72LTVM, **73S**T, 74SP, 75I, 76V, **77I**, 79A, **82A**, 83D, **84V**A, 85V, 89V, 90M, 91S, 92K, 93LM, 95V
**TPV**
**10IV**, 11LI, 12K, 13V, 14R, 15V, 16A, 19V, 20RTV, 21D, 22V, 24IM, 32I, **33F**, 34QDF, 35DG, **36IV**, 37D, **43T**, 45R, **46LI**, 47V, 53L, **54LVM**, 55R, 57K, 58E, 60E, 61HN, 62V, 63P, 64V, 66V, 70T, 71IVM, 72TVM, 73ST, 74P, 77I, 82LTA, 83D, 84V, 85L, 87G, **89VM**, 90M, 91S, 93LM

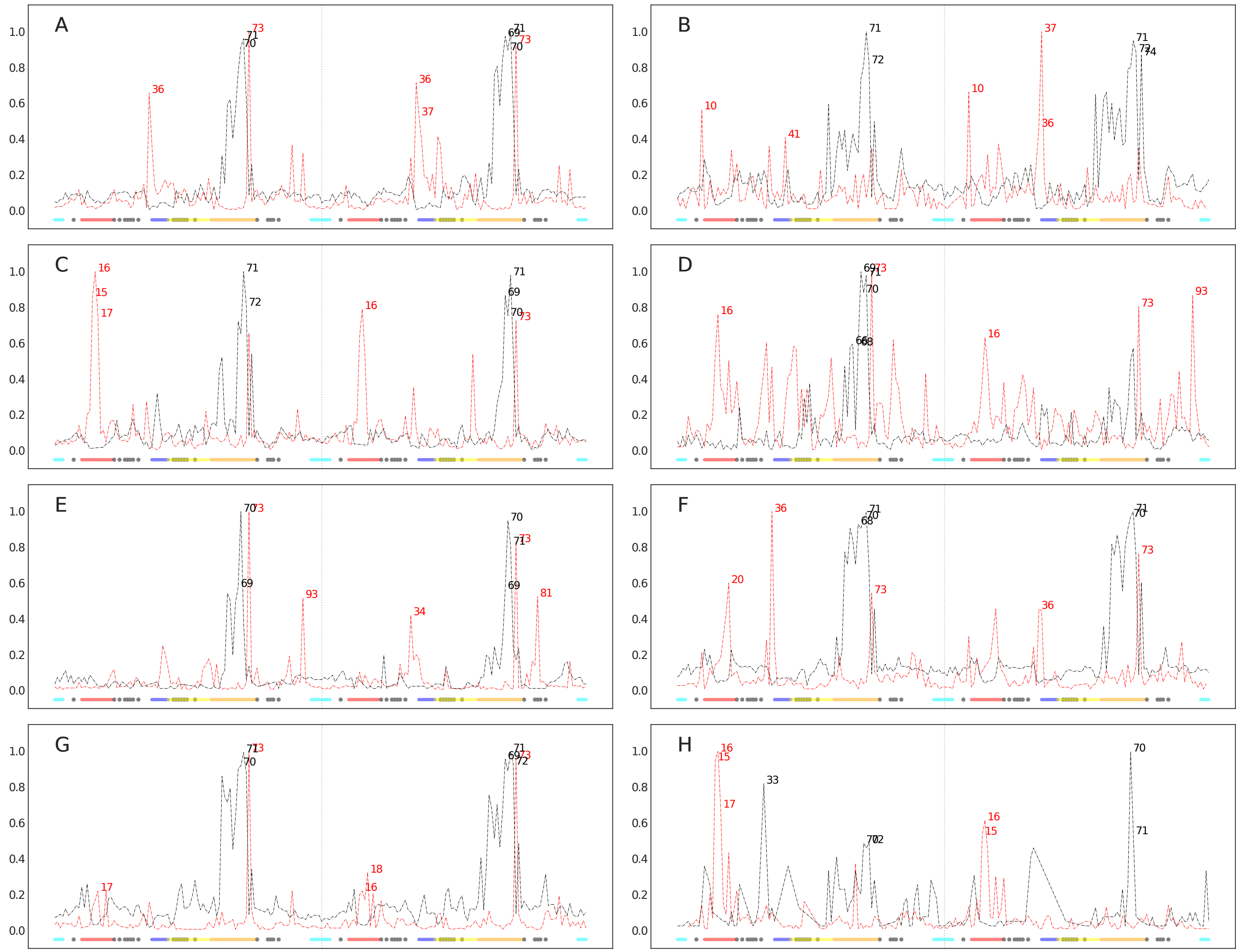

Supplementary Figure S5: Normalized degree centralities for replicate molecular dynamics simulations showing significantly larger (red lines) and smaller (black lines) distances observed in resistant ensembles for 8 FDA-approved protease inhibitor complexes, namely ATV (A), DRV (B), FPV (C), IDV (D), LPV (E), NFV (F), SQV (G) and TPV (H). The top 5 residue positions with the highest connectivities are labelled at the peaks in each graph. Inserted underneath are the functional protease residues depicted as colored dots, namely the fulcrum (red), the elbow (blue), the flap (yellow), the cantilever (orange), the interface (cyan) and the binding cavity residues (grey).

Supplementary dataset S6: HIV protease sequences used for the ensembles. Sequence headers consist of the sequence IDs and the fold resistance scores. An additional number is appended after an underscore as an internal representation of the mutant after sequence expansion.

Sequences for the ATV-resistant ensemble
>105312_1 700.0
PQITLWQRPVLTVKIGGQLKEALLDTGADDTVFEEMDLPGKWKPKLIGGIGGFVRVRQYEDIPIEICGYKVTSP
VLIGPTPTNVIGRNVMTKIGCTLNF
>105312_3 700.0
PQITLWQRPVLTVKIGGQLKEALLDTGADDTVFEEVDLPGKWKPKLIGGIGGFVRVRQYEDIPIEICGYKVTSP
VLIGPTPTNVIGRNVMTKIGCTLNF
>105312_5 700.0
PQITLWQRPVLTVRIGGQLKEALLDTGADDTVFEEMDLPGKWKPKLIGGIGGFVRVRQYEDIPIEICGYKVTSP
VLIGPTPTNVIGRNVMTKIGCTLNF
>105312_7 700.0
PQITLWQRPVLTVRIGGQLKEALLDTGADDTVFEEVDLPGKWKPKLIGGIGGFVRVRQYEDIPIEICGYKVTSP
VLIGPTPTNVIGRNVMTKIGCTLNF
>117039_2 700.0
PQITLWQRPVITVKIGGQLVEALMDTGADDTIFEGINLPGRWKPKMIGGIGGFLNVRQYEQIPIEICGHKILSTVL
VGSTPVNVIGRNVMSKIGCTLNF
>117075_0 700.0
PQITLWQRPLITVKIGGQLVEALMDTGADDTIFEGINLPGRWKPKMIGGIGGFLNVRQYEQIPIEICGHKILSTVL
VGSTPLNVIGRNVMSKIGCTLNF
>117075_11 700.0
PQITLWQRPVITVKVGGQLVEALMDTGADDTIFEGINLPGRWKPKMIGGIGGFLNVREYEQIPIEICGHKILSTV
LVGSTPLNVIGRNVMSKIGCTLNF
>105312_4 700.0
PQITLWQRPVLTVRIGGQLKEALLDTGADDTVFEEMDLPGKWKPKLIGGIGGFVRVRQYEDIPIEICGHKVTSP
VLIGPTPTNVIGRNVMTKIGCTLNF
>105312_6 700.0
PQITLWQRPVLTVRIGGQLKEALLDTGADDTVFEEVDLPGKWKPKLIGGIGGFVRVRQYEDIPIEICGHKVTSP
VLIGPTPTNVIGRNVMTKIGCTLNF
>115755_0 700.0
PQITLWQRPIVTIKVGGQIMEALIDTGADDTVLEDINLPGRWKPKMIQGIGGLVKVRQYDQIPIEICGHEVMSTV
LVGPTPANIIGRNLLTQIGCTLNF
>115755_1 700.0
PQITLWQRPIVTIKVGGQIMEALIDTGADDTVLEDINLPGRWKPKMIQGIGGLVKVRQYDQIPIEICGHEVMSTV
LVGPTPANIIGRNLLTQMGCTLNF
>115755_2 700.0
PQITLWQRPIVTIKVGGQIMEALIDTGADDTVLEDINLPGRWKPKMIQGIGGLVKVRQYDQIPIEICGHEVMSTIL
VGPTPANIIGRNLLTQIGCTLNF
>115755_3 700.0
PQITLWQRPIVTIKVGGQIMEALIDTGADDTVLEDINLPGRWKPKMIQGIGGLVKVRQYDQIPIEICGHEVMSTIL
VGPTPANIIGRNLLTQMGCTLNF
>143473_17 700.0
PQITLWQRPIVIVKIGGQLREALLDTGADDTVFEDIDLPGRWTPKLIGGIGGFVKVRQYDQVPIEICGHKITSTV
LVGPTPANIIGRNLMTQLGCTLNF
>143473_19 700.0
PQITLWQRPIVIVKIGGQLREALLDTGADDTVFEDIDLPGRWTPKLIGGIGGFVKVRQYDQVPIEICGHKVTSTV
LVGPTPANIIGRNLMTQLGCTLNF
>143473_21 700.0
PQITLWQRPIVIVKIGGQLREALLDTGADDTVFEDIDLPGRWTPKLIGGIGGFVRVRQYDQVPIEICGHKITSTV
LVGPTPANIIGRNLMTQLGCTLNF
>143473_23 700.0
PQITLWQRPIVIVKIGGQLREALLDTGADDTVFEDIDLPGRWTPKLIGGIGGFVRVRQYDQVPIEICGHKVTSTV
LVGPTPANIIGRNLMTQLGCTLNF
>143473_27 700.0
PQITLWQRPIVIVKIGGQIREALLDTGADDTVFEDIDLPGRWTPKLIGGIGGFVKVRQYDQVPIEICGHKVTSTV
LVGPTPANIIGRNLMTQLGCTLNF
>98601_0 700.0
PQITLWQRPFVTIKIGGQLIEALLDTGADDTVLEEMNLPGRWKPKIIGGIGGFMKVRQYDQIPIEICGHKVITTVL
IGPTPVNAIGRNLLTQIGCTLNF
>98604_0 700.0

PQITLWQRPFVTIKIGGQLIEALLDTGADDTVLEEMNLPGRWKPKIIGGIGGFVKVRQYDQIPIEICGHKVISTVL
VGPTPVNAIGRNLLTQIGCTLNF
>98604_1 700.0
PQITLWQRPFVTIKIGGQLIEALLDTGADDTVLEEINLPGRWKPKIIGGIGGFVKVRQYDQIPIEICGHKVISTVLV
GPTPVNAIGRNLLTQIGCTLNF
>123049_0 700.0
PQITLWQRPIITIKIGGQLKDVLMDTGADDTVLEDIDLPGRWKPKLIGGIGGFVKVRQYDHVPVEVCGHKVTTT
VLVGPTPVDVIGRNLLTQIGCTLNF
>123049_1 700.0
PQITLWQRPIITIKIGGQLKDVLMDTGADDTVLEDIDLPGRWKPKLIGGIGGFVKVRQYDNVPVEVCGHKVTTT
VLVGPTPVDVIGRNLLTQIGCTLNF
>123049_2 700.0
PQITLWQRPIITIKIGGQLKDVLMDTGADDTVLEDIDLPGRWKPKLIGGIGGFVKVKQYDHVPVEVCGHKVTTT
VLVGPTPVDVIGRNLLTQIGCTLNF
>123049_3 700.0
PQITLWQRPIITIKIGGQLKDVLMDTGADDTVLEDIDLPGRWKPKLIGGIGGFVKVKQYDNVPVEVCGHKVTTT
VLVGPTPVDVIGRNLLTQIGCTLNF
>123049_4 700.0
PQITLWQRPIITIKIGGQLKDVLMDTGADDTVLEDIDLPGKWKPKLIGGIGGFVKVRQYDHVPVEVCGHKVTTT
VLVGPTPVDVIGRNLLTQIGCTLNF
>123049_5 700.0
PQITLWQRPIITIKIGGQLKDVLMDTGADDTVLEDIDLPGKWKPKLIGGIGGFVKVRQYDNVPVEVCGHKVTTT
VLVGPTPVDVIGRNLLTQIGCTLNF
>123049_6 700.0
PQITLWQRPIITIKIGGQLKDVLMDTGADDTVLEDIDLPGKWKPKLIGGIGGFVKVKQYDHVPVEVCGHKVTTT
VLVGPTPVDVIGRNLLTQIGCTLNF
>123049_7 700.0
PQITLWQRPIITIKIGGQLKDVLMDTGADDTVLEDIDLPGKWKPKLIGGIGGFVKVKQYDNVPVEVCGHKVTTT
VLVGPTPVDVIGRNLLTQIGCTLNF
>123049_8 700.0
PQITLWQRPIITIKIGGQLKDVLMDTGADDTVLENIDLPGRWKPKLIGGIGGFVKVRQYDHVPVEVCGHKVTTT
VLVGPTPVDVIGRNLLTQIGCTLNF
>123049_9 700.0
PQITLWQRPIITIKIGGQLKDVLMDTGADDTVLENIDLPGRWKPKLIGGIGGFVKVRQYDNVPVEVCGHKVTTT
VLVGPTPVDVIGRNLLTQIGCTLNF
>123049_10 700.0
PQITLWQRPIITIKIGGQLKDVLMDTGADDTVLENIDLPGRWKPKLIGGIGGFVKVKQYDHVPVEVCGHKVTTT
VLVGPTPVDVIGRNLLTQIGCTLNF
>123049_11 700.0
PQITLWQRPIITIKIGGQLKDVLMDTGADDTVLENIDLPGRWKPKLIGGIGGFVKVKQYDNVPVEVCGHKVTTT
VLVGPTPVDVIGRNLLTQIGCTLNF
>123049_12 700.0
PQITLWQRPIITIKIGGQLKDVLMDTGADDTVLENIDLPGKWKPKLIGGIGGFVKVRQYDHVPVEVCGHKVTTT
VLVGPTPVDVIGRNLLTQIGCTLNF
>123049_13 700.0
PQITLWQRPIITIKIGGQLKDVLMDTGADDTVLENIDLPGKWKPKLIGGIGGFVKVRQYDNVPVEVCGHKVTTT
VLVGPTPVDVIGRNLLTQIGCTLNF
>123049_15 700.0
PQITLWQRPIITIKIGGQLKDVLMDTGADDTVLENIDLPGKWKPKLIGGIGGFVKVKQYDNVPVEVCGHKVTTT
VLVGPTPVDVIGRNLLTQIGCTLNF
>107987_0 700.0
PQISLWQRPLITVKIGGQVIEALLDTGADDTVVEDINLPGRWKPKIIGGIGGFVRVKQYDQIPVEIFGRKIFTTVL
VGATPVNVIGRNLMTQLGCSLNF
>107987_1 700.0
PQISLWQRPLITVKIGGQVIEALLDTGADDTVVEDINLPGRWKPKIIGGIGGFVRVKQYDQIPVEIFGRKIFTTVL
VGATPLNVIGRNLMTQLGCSLNF
>107987_2 700.0
PQISLWQRPLITVKIGGQVIEALLDTGADDTVVEDINLPGRWKPKLIGGIGGFVRVKQYDQIPVEIFGRKIFTTVL
VGATPVNVIGRNLMTQLGCSLNF
>107987_3 700.0
PQISLWQRPLITVKIGGQVIEALLDTGADDTVVEDINLPGRWKPKLIGGIGGFVRVKQYDQIPVEIFGRKIFTTVL
VGATPLNVIGRNLMTQLGCSLNF
>107987_4 700.0

PQISLWQRPLITVKIGGQVIEALLDTGADDTVVEDISLPGRWKPKIIGGIGGFVRVKQYDQIPVEIFGRKIFTTVL
VGATPVNVIGRNLMTQLGCSLNF
>107987_5 700.0
PQISLWQRPLITVKIGGQVIEALLDTGADDTVVEDISLPGRWKPKIIGGIGGFVRVKQYDQIPVEIFGRKIFTTVL
VGATPLNVIGRNLMTQLGCSLNF
>107987_6 700.0
PQISLWQRPLITVKIGGQVIEALLDTGADDTVVEDISLPGRWKPKLIGGIGGFVRVKQYDQIPVEIFGRKIFTTVL
VGATPVNVIGRNLMTQLGCSLNF
>107987_7 700.0
PQISLWQRPLITVKIGGQVIEALLDTGADDTVVEDISLPGRWKPKLIGGIGGFVRVKQYDQIPVEIFGRKIFTTVL
VGATPLNVIGRNLMTQLGCSLNF
>107987_8 700.0
PQISLWQRPLITVKIGGQVVEALLDTGADDTVVEDINLPGRWKPKIIGGIGGFVRVKQYDQIPVEIFGRKIFTTVL
VGATPVNVIGRNLMTQLGCSLNF
>107987_9 700.0
PQISLWQRPLITVKIGGQVVEALLDTGADDTVVEDINLPGRWKPKIIGGIGGFVRVKQYDQIPVEIFGRKIFTTVL
VGATPLNVIGRNLMTQLGCSLNF
>107987_10 700.0
PQISLWQRPLITVKIGGQVVEALLDTGADDTVVEDINLPGRWKPKLIGGIGGFVRVKQYDQIPVEIFGRKIFTTV
LVGATPVNVIGRNLMTQLGCSLNF
>107987_11 700.0
PQISLWQRPLITVKIGGQVVEALLDTGADDTVVEDINLPGRWKPKLIGGIGGFVRVKQYDQIPVEIFGRKIFTTV
LVGATPLNVIGRNLMTQLGCSLNF
>107987_12 700.0
PQISLWQRPLITVKIGGQVVEALLDTGADDTVVEDISLPGRWKPKIIGGIGGFVRVKQYDQIPVEIFGRKIFTTVL
VGATPVNVIGRNLMTQLGCSLNF
>107987_13 700.0
PQISLWQRPLITVKIGGQVVEALLDTGADDTVVEDISLPGRWKPKIIGGIGGFVRVKQYDQIPVEIFGRKIFTTVL
VGATPLNVIGRNLMTQLGCSLNF
>107987_14 700.0
PQISLWQRPLITVKIGGQVVEALLDTGADDTVVEDISLPGRWKPKLIGGIGGFVRVKQYDQIPVEIFGRKIFTTV
LVGATPVNVIGRNLMTQLGCSLNF
>107987_15 700.0
PQISLWQRPLITVKIGGQVVEALLDTGADDTVVEDISLPGRWKPKLIGGIGGFVRVKQYDQIPVEIFGRKIFTTV
LVGATPLNVIGRNLMTQLGCSLNF
>113050_0 700.0
PQITLWQRPFVTVKIGGQITEALLDTGADDTILENINLPGRWKPKIIQGIGGFLKVRQYDQIPIEIEGHKVIGSVV
VGPTPVNVIGRNVMSQLGCTLNF
>115739_0 700.0
PQITLWQRPVVTVKVGGQLREALLDTGADDTIFEDISLPGKWIPKIVGGIGGLIKVKQYDQIPIEICGHKVISTVL
VGPTPANIIGRNLMTQLGCTLNF
>115739_1 700.0
PQITLWQRPVVTVKVGGQLREALLDTGADDTIFEDISLPGKWIPKIVGGIGGLIKVKQYDQIPIEICGHKVISTVL
VGPTPANIIGRNLMTQLGFTLNF
>113192_1 700.0
PQITLWQRPFVTMKIGGQLIEALLDTGADDTVLEEISLPGRWKPKIIGGIGGFVKVRQYDQVPIEVFGHKATTT
VLVGPTPVNVIGRNLMTQLGCTLNF
>113192_3 700.0
PQITLWQRPFVTMKIGGQLIEALLDTGADDTVLEEISLPGRWKPKIIGGIGGFVKVRQYDQVPIEVFGQKATTT
VLVGPTPVNVIGRNLMTQLGCTLNF
>113192_5 700.0
PQITLWQRPFVTMKIGGQLIEALLDTGADDTVLEEISLPGRWKPKIIGGIGGFVKVRQYDQVPVEVFGHKATTT
VLVGPTPVNVIGRNLMTQLGCTLNF
>113192_7 700.0
PQITLWQRPFVTMKIGGQLIEALLDTGADDTVLEEISLPGRWKPKIIGGIGGFVKVRQYDQVPVEVFGQKATTT
VLVGPTPVNVIGRNLMTQLGCTLNF
>113192_9 700.0
PQITLWQRPFVTMKIGGQIIEALLDTGADDTVLEEISLPGRWKPKIIGGIGGFVKVRQYDQVPIEVFGHKATTTV
LVGPTPVNVIGRNLMTQLGCTLNF
>113192_11 700.0
PQITLWQRPFVTMKIGGQIIEALLDTGADDTVLEEISLPGRWKPKIIGGIGGFVKVRQYDQVPIEVFGQKATTTV
LVGPTPVNVIGRNLMTQLGCTLNF
>113192_13 700.0

PQITLWQRPFVTMKIGGQIIEALLDTGADDTVLEEISLPGRWKPKIIGGIGGFVKVRQYDQVPVEVFGHKATTT
VLVGPTPVNVIGRNLMTQLGCTLNF
>113192_15 700.0
PQITLWQRPFVTMKIGGQIIEALLDTGADDTVLEEISLPGRWKPKIIGGIGGFVKVRQYDQVPVEVFGQKATTT
VLVGPTPVNVIGRNLMTQLGCTLNF
>143473_16 700.0
PQITLWQRPIVIVKIGGQLREALLDTGADDTVFEDIDLPGRWTPKLIGGIGGFVKVRQYDQVPIEICGHKIISTVL
VGPTPANIIGRNLMTQLGCTLNF
>143473_18 700.0
PQITLWQRPIVIVKIGGQLREALLDTGADDTVFEDIDLPGRWTPKLIGGIGGFVKVRQYDQVPIEICGHKVISTV
LVGPTPANIIGRNLMTQLGCTLNF
>143473_20 700.0
PQITLWQRPIVIVKIGGQLREALLDTGADDTVFEDIDLPGRWTPKLIGGIGGFVRVRQYDQVPIEICGHKIISTVL
VGPTPANIIGRNLMTQLGCTLNF
>143473_22 700.0
PQITLWQRPIVIVKIGGQLREALLDTGADDTVFEDIDLPGRWTPKLIGGIGGFVRVRQYDQVPIEICGHKVISTV
LVGPTPANIIGRNLMTQLGCTLNF
>143473_24 700.0
PQITLWQRPIVIVKIGGQIREALLDTGADDTVFEDIDLPGRWTPKLIGGIGGFVKVRQYDQVPIEICGHKIISTVL
VGPTPANIIGRNLMTQLGCTLNF
>143473_26 700.0
PQITLWQRPIVIVKIGGQIREALLDTGADDTVFEDIDLPGRWTPKLIGGIGGFVKVRQYDQVPIEICGHKVISTVL
VGPTPANIIGRNLMTQLGCTLNF
>143473_28 700.0
PQITLWQRPIVIVKIGGQIREALLDTGADDTVFEDIDLPGRWTPKLIGGIGGFVRVRQYDQVPIEICGHKIISTVL
VGPTPANIIGRNLMTQLGCTLNF
>143473_30 700.0
PQITLWQRPIVIVKIGGQIREALLDTGADDTVFEDIDLPGRWTPKLIGGIGGFVRVRQYDQVPIEICGHKVISTVL
VGPTPANIIGRNLMTQLGCTLNF
>115065_2 700.0
PQITLWQRPVVTVKIAGQLKEALIDTGADDTIFFEMNLPGRWKPKLIGGIGGFVKVRQYDQIPIEICGHKVVGT
VLIGPTPTNVIGRNLLTQIGCTLNF
>115065_3 700.0
PQITLWQRPVVTVKIAGQLKEALIDTGADDTIFFEMDLPGRWKPKLIGGIGGFVKVRQYDQIPIEICGHKVVGT
VLIGPTPTNVIGRNLLTQIGCTLNF
>143473_1 700.0
PQITLWQRPIVTVKIGGQLREALLDTGADDTVFEDIDLPGRWTPKLIGGIGGFVKVRQYDQVPIEICGHKITSTV
LVGPTPANIIGRNLMTQLGCTLNF
>143473_3 700.0
PQITLWQRPIVTVKIGGQLREALLDTGADDTVFEDIDLPGRWTPKLIGGIGGFVKVRQYDQVPIEICGHKVTST
VLVGPTPANIIGRNLMTQLGCTLNF
>143473_5 700.0
PQITLWQRPIVTVKIGGQLREALLDTGADDTVFEDIDLPGRWTPKLIGGIGGFVRVRQYDQVPIEICGHKITSTV
LVGPTPANIIGRNLMTQLGCTLNF
>143473_7 700.0
PQITLWQRPIVTVKIGGQLREALLDTGADDTVFEDIDLPGRWTPKLIGGIGGFVRVRQYDQVPIEICGHKVTST
VLVGPTPANIIGRNLMTQLGCTLNF
>143473_9 700.0
PQITLWQRPIVTVKIGGQIREALLDTGADDTVFEDIDLPGRWTPKLIGGIGGFVKVRQYDQVPIEICGHKITSTV
LVGPTPANIIGRNLMTQLGCTLNF
>143473_11 700.0
PQITLWQRPIVTVKIGGQIREALLDTGADDTVFEDIDLPGRWTPKLIGGIGGFVKVRQYDQVPIEICGHKVTSTV
LVGPTPANIIGRNLMTQLGCTLNF
>143473_13 700.0
PQITLWQRPIVTVKIGGQIREALLDTGADDTVFEDIDLPGRWTPKLIGGIGGFVRVRQYDQVPIEICGHKITSTV
LVGPTPANIIGRNLMTQLGCTLNF
>143473_15 700.0
PQITLWQRPIVTVKIGGQIREALLDTGADDTVFEDIDLPGRWTPKLIGGIGGFVRVRQYDQVPIEICGHKVTSTV
LVGPTPANIIGRNLMTQLGCTLNF
>115081_0 700.0
PQITLWQRPIVTIKIGGQLREALLDTGADDTVLEDIDLPGRWKPKLIVGIGGFVKVRQYEQVPIEICGHKVVGTV
LIGPTPSNIIGRNLMTQLGCTLNF
>113192_0 700.0

PQITLWQRPFVTMKIGGQLIEALLDTGADDTVLEEISLPGRWKPKIIGGIGGFVKVRQYDQVPIEVFGHKAITTV
LVGPTPVNVIGRNLMTQLGCTLNF
>113192_2 700.0
PQITLWQRPFVTMKIGGQLIEALLDTGADDTVLEEISLPGRWKPKIIGGIGGFVKVRQYDQVPIEVFGQKAITTV
LVGPTPVNVIGRNLMTQLGCTLNF
>103067_1 700.0
PQITLWQRPFITVKIAGQVKEALLDTGADDTVFQEMNLPGRWIPKLIGGIAGFMKVRQYDQIPMEIYGHKVMA
TVLVGPTPVNVIGRNLMTQIGCTLNF
>145212_0 700.0
PQITLWQRPIVTVKIAGQLKEALLDTGADDTIFEEMNLPGRWTPKLIGGIGGFLKVRQYDQVPIEICGHKVIGPV
LVGPTPVNVIGRNLMSQIGCTLNF
>145212_2 700.0
PQITLWQRPIVTVRIAGQLKEALLDTGADDTIFEEMNLPGRWTPKLIGGIGGFLKVRQYDQVPIEICGHKVIGPV
LVGPTPVNVIGRNLMSQIGCTLNF
>145212_4 700.0
PQITLWQRPIITVKIAGQLKEALLDTGADDTIFEEMNLPGRWTPKLIGGIGGFLKVRQYDQVPIEICGHKVIGPV
LVGPTPVNVIGRNLMSQIGCTLNF
>145212_6 700.0
PQITLWQRPIITVRIAGQLKEALLDTGADDTIFEEMNLPGRWTPKLIGGIGGFLKVRQYDQVPIEICGHKVIGPV
LVGPTPVNVIGRNLMSQIGCTLNF
>115739_2 700.0
PQITLWQRPVVTVKVGGQLREALLDTGADDTIFEDISLPGKWTPKIVGGIGGLIKVKQYDQIPIEICGHKVISTVL
VGPTPANIIGRNLMTQLGCTLNF
>115739_3 700.0
PQITLWQRPVVTVKVGGQLREALLDTGADDTIFEDISLPGKWTPKIVGGIGGLIKVKQYDQIPIEICGHKVISTVL
VGPTPANIIGRNLMTQLGFTLNF
>143473_0 700.0
PQITLWQRPIVTVKIGGQLREALLDTGADDTVFEDIDLPGRWTPKLIGGIGGFVKVRQYDQVPIEICGHKIISTV
LVGPTPANIIGRNLMTQLGCTLNF
>143473_4 700.0
PQITLWQRPIVTVKIGGQLREALLDTGADDTVFEDIDLPGRWTPKLIGGIGGFVRVRQYDQVPIEICGHKIISTV
LVGPTPANIIGRNLMTQLGCTLNF
>143473_6 700.0
PQITLWQRPIVTVKIGGQLREALLDTGADDTVFEDIDLPGRWTPKLIGGIGGFVRVRQYDQVPIEICGHKVISTV
LVGPTPANIIGRNLMTQLGCTLNF
>143473_8 700.0
PQITLWQRPIVTVKIGGQIREALLDTGADDTVFEDIDLPGRWTPKLIGGIGGFVKVRQYDQVPIEICGHKIISTVL
VGPTPANIIGRNLMTQLGCTLNF
>143473_10 700.0
PQITLWQRPIVTVKIGGQIREALLDTGADDTVFEDIDLPGRWTPKLIGGIGGFVKVRQYDQVPIEICGHKVISTV
LVGPTPANIIGRNLMTQLGCTLNF
>143473_12 700.0
PQITLWQRPIVTVKIGGQIREALLDTGADDTVFEDIDLPGRWTPKLIGGIGGFVRVRQYDQVPIEICGHKIISTVL
VGPTPANIIGRNLMTQLGCTLNF
>143473_14 700.0
PQITLWQRPIVTVKIGGQIREALLDTGADDTVFEDIDLPGRWTPKLIGGIGGFVRVRQYDQVPIEICGHKVISTV
LVGPTPANIIGRNLMTQLGCTLNF
>115065_0 700.0
PQITLWQRPVVTVKIAGQLKEALIDTGADDTIFEEMNLPGRWKPKLIGGIGGFVKVRQYDQIPIEICGHKVVGT
VLIGPTPTNVIGRNLLTQIGCTLNF
>115065_1 700.0
PQITLWQRPVVTVKIAGQLKEALIDTGADDTIFEEMDLPGRWKPKLIGGIGGFVKVRQYDQIPIEICGHKVVGT
VLIGPTPTNVIGRNLLTQIGCTLNF

Sequences for the ATV-susceptible ensemble
>259249_3 0.5
PQITLWQRPLVAVKIGEQLIEALLDTGADDTVLEEINLPGKWKPKMIGGIGGFIKVGQYDQIPIEICGKRALGTVL
VGPTPVNIIGRNILTQIGCTLNF
>102783_3 0.4
PQITLWQRPITTIKIGGQLKEALLDTGADDTVLEDMNLPGRWKPKMIGGIGGFIKVRQYDEVPLEICGHKVTGT
VLIGSTPVNIIGRNLLTQLGCTLNF
>102783_2 0.4
PQITLWQRPITTIKIGGQLKEALLDTGADDTVLEDMNLPGRWKPKMIGGIGGFIKVRQYDEVPLEICGHKVTGT

VLIGATPVNIIGRNLLTQLGCTLNF
>102795_0 0.5
PQITLWQRPVVTIKVEGQLKEALLDTGADDTVLEEMNLPGRWKPKMIGGIGGFIKVRQYDQILIEICGHKARGT
VLVGPTPVNIIGRNLLTQIGCTLNF
>102795_1 0.5
PQITLWQRPVVTIKVEGQLKEALLDTGADDTVLEEMNLPGRWKPKMIGGIGGFIKVRQYDQILIEICGHKARGT
VLVGPTPVNIIGRTLLTQIGCTLNF
>102795_4 0.5
PQITLWQRPVVTIKVEGQLKEALLDTGADDTVLEEMNLPGRWKPKMIGGLGGFIKVRQYDQILIEICGHKARG
TVLVGPTPVNIIGRNLLTQIGCTLNF
>102795_5 0.5
PQITLWQRPVVTIKVEGQLKEALLDTGADDTVLEEMNLPGRWKPKMIGGLGGFIKVRQYDQILIEICGHKARG
TVLVGPTPVNIIGRTLLTQIGCTLNF
>235727_0 0.5
PQITLWQRPLVPVKIGGQLKEALLDTGADDTVLEEMDLPGRWKPKMIGGIGGFIKVGQYDQIQVDICGHKAIG
TVLVGPTPVNIIGRNLLTQIGCTLNF
>235727_1 0.5
PQITLWQRPLVPVRIGGQLKEALLDTGADDTVLEEMDLPGRWKPKMIGGIGGFIKVGQYDQIQVDICGHKAIG
TVLVGPTPVNIIGRNLLTQIGCTLNF
>235727_2 0.5
PQITLWQRPVVPVKIGGQLKEALLDTGADDTVLEEMDLPGRWKPKMIGGIGGFIKVGQYDQIQVDICGHKAIG
TVLVGPTPVNIIGRNLLTQIGCTLNF
>235727_3 0.5
PQITLWQRPVVPVRIGGQLKEALLDTGADDTVLEEMDLPGRWKPKMIGGIGGFIKVGQYDQIQVDICGHKAIG
TVLVGPTPVNIIGRNLLTQIGCTLNF
>102783_1 0.4
PQITLWQRPIITIKIGGQLKEALLDTGADDTVLEDMNLPGRWKPKMIGGIGGFIKVRQYDEVPLEICGHKVTGT
VLIGSTPVNIIGRNLLTQLGCTLNF
>102783_0 0.4
PQITLWQRPIITIKIGGQLKEALLDTGADDTVLEDMNLPGRWKPKMIGGIGGFIKVRQYDEVPLEICGHKVTGT
VLIGATPVNIIGRNLLTQLGCTLNF
>109470_1 0.5
PQITLWQRPLVTIKIGGQLKEALLDTGADDTVLEEMNLPGRWKPKMIEGIGGFIKVRQYDEISIDICGHKAVGTV
LIGPTPVNIIGRNLLTQIGCTLNF
>187159_1 0.5
PQITLWQRPLVTIKVGGQLKEALLDTGADDTVLEEMCLPGRWKPKMIGGIGGFIKVRQYDQILIEICGHKVIGT
VLIGPTPVNIIGRNLLTQIGCTLNF
>187159_5 0.5
PQITLWQRPLVTIKVGGQLKEALLDTGADDTVLEEMCLPGRWKPKMIGGIGGFIKVRQYDQLLIEICGHKVIGT
VLIGPTPVNIIGRNLLTQIGCTLNF
>187159_9 0.5
PQITLWQRPLVTIKVGGQLKEALLDTGADDTVLEEMCLPGKWKPKMIGGIGGFIKVRQYDQILIEICGHKVIGTV
LIGPTPVNIIGRNLLTQIGCTLNF
>187159_13 0.5
PQITLWQRPLVTIKVGGQLKEALLDTGADDTVLEEMCLPGKWKPKMIGGIGGFIKVRQYDQLLIEICGHKVIGT
VLIGPTPVNIIGRNLLTQIGCTLNF
>187159_17 0.5
PQITLWQRPLVTIKVGGQLKEALLDTGADDTVLEDMCLPGRWKPKMIGGIGGFIKVRQYDQILIEICGHKVIGT
VLIGPTPVNIIGRNLLTQIGCTLNF
>187159_21 0.5
PQITLWQRPLVTIKVGGQLKEALLDTGADDTVLEDMCLPGRWKPKMIGGIGGFIKVRQYDQLLIEICGHKVIGT
VLIGPTPVNIIGRNLLTQIGCTLNF
>187159_25 0.5
PQITLWQRPLVTIKVGGQLKEALLDTGADDTVLEDMCLPGKWKPKMIGGIGGFIKVRQYDQILIEICGHKVIGT
VLIGPTPVNIIGRNLLTQIGCTLNF
>187159_29 0.5
PQITLWQRPLVTIKVGGQLKEALLDTGADDTVLEDMCLPGKWKPKMIGGIGGFIKVRQYDQLLIEICGHKVIGT
VLIGPTPVNIIGRNLLTQIGCTLNF
>187159_33 0.5
PQITLWQRPLVTVKVGGQLKEALLDTGADDTVLEEMCLPGRWKPKMIGGIGGFIKVRQYDQILIEICGHKVIGT
VLIGPTPVNIIGRNLLTQIGCTLNF
>187159_37 0.5
PQITLWQRPLVTVKVGGQLKEALLDTGADDTVLEEMCLPGRWKPKMIGGIGGFIKVRQYDQLLIEICGHKVIG

TVLIGPTPVNIIGRNLLTQIGCTLNF
>187159_41 0.5
PQITLWQRPLVTVKVGGQLKEALLDTGADDTVLEEMCLPGKWKPKMIGGIGGFIKVRQYDQILIEICGHKVIGT
VLIGPTPVNIIGRNLLTQIGCTLNF
>187159_45 0.5
PQITLWQRPLVTVKVGGQLKEALLDTGADDTVLEEMCLPGKWKPKMIGGIGGFIKVRQYDQLLIEICGHKVIGT
VLIGPTPVNIIGRNLLTQIGCTLNF
>187159_49 0.5
PQITLWQRPLVTVKVGGQLKEALLDTGADDTVLEDMCLPGRWKPKMIGGIGGFIKVRQYDQILIEICGHKVIGT
VLIGPTPVNIIGRNLLTQIGCTLNF
>187159_53 0.5
PQITLWQRPLVTVKVGGQLKEALLDTGADDTVLEDMCLPGRWKPKMIGGIGGFIKVRQYDQLLIEICGHKVIG
TVLIGPTPVNIIGRNLLTQIGCTLNF
>187159_57 0.5
PQITLWQRPLVTVKVGGQLKEALLDTGADDTVLEDMCLPGKWKPKMIGGIGGFIKVRQYDQILIEICGHKVIGT
VLIGPTPVNIIGRNLLTQIGCTLNF
>187159_69 0.5
PQITLWQRPIVTIKVGGQLKEALLDTGADDTVLEEMCLPGRWKPKMIGGIGGFIKVRQYDQLLIEICGHKVIGT
VLIGPTPVNIIGRNLLTQIGCTLNF
>187159_73 0.5
PQITLWQRPIVTIKVGGQLKEALLDTGADDTVLEEMCLPGKWKPKMIGGIGGFIKVRQYDQILIEICGHKVIGTV
LIGPTPVNIIGRNLLTQIGCTLNF
>187159_77 0.5
PQITLWQRPIVTIKVGGQLKEALLDTGADDTVLEEMCLPGKWKPKMIGGIGGFIKVRQYDQLLIEICGHKVIGT
VLIGPTPVNIIGRNLLTQIGCTLNF
>187159_81 0.5
PQITLWQRPIVTIKVGGQLKEALLDTGADDTVLEDMCLPGRWKPKMIGGIGGFIKVRQYDQILIEICGHKVIGTV
LIGPTPVNIIGRNLLTQIGCTLNF
>187159_85 0.5
PQITLWQRPIVTIKVGGQLKEALLDTGADDTVLEDMCLPGRWKPKMIGGIGGFIKVRQYDQLLIEICGHKVIGT
VLIGPTPVNIIGRNLLTQIGCTLNF
>187159_89 0.5
PQITLWQRPIVTIKVGGQLKEALLDTGADDTVLEDMCLPGKWKPKMIGGIGGFIKVRQYDQILIEICGHKVIGTV
LIGPTPVNIIGRNLLTQIGCTLNF
>187159_93 0.5
PQITLWQRPIVTIKVGGQLKEALLDTGADDTVLEDMCLPGKWKPKMIGGIGGFIKVRQYDQLLIEICGHKVIGT
VLIGPTPVNIIGRNLLTQIGCTLNF
>187159_97 0.5
PQITLWQRPIVTVKVGGQLKEALLDTGADDTVLEEMCLPGRWKPKMIGGIGGFIKVRQYDQILIEICGHKVIGT
VLIGPTPVNIIGRNLLTQIGCTLNF
>187159_101 0.5
PQITLWQRPIVTVKVGGQLKEALLDTGADDTVLEEMCLPGRWKPKMIGGIGGFIKVRQYDQLLIEICGHKVIGT
VLIGPTPVNIIGRNLLTQIGCTLNF
>187159_109 0.5
PQITLWQRPIVTVKVGGQLKEALLDTGADDTVLEEMCLPGKWKPKMIGGIGGFIKVRQYDQLLIEICGHKVIGT
VLIGPTPVNIIGRNLLTQIGCTLNF
>187159_113 0.5
PQITLWQRPIVTVKVGGQLKEALLDTGADDTVLEDMCLPGRWKPKMIGGIGGFIKVRQYDQILIEICGHKVIGT
VLIGPTPVNIIGRNLLTQIGCTLNF
>187159_117 0.5
PQITLWQRPIVTVKVGGQLKEALLDTGADDTVLEDMCLPGRWKPKMIGGIGGFIKVRQYDQLLIEICGHKVIGT
VLIGPTPVNIIGRNLLTQIGCTLNF
>187159_121 0.5
PQITLWQRPIVTVKVGGQLKEALLDTGADDTVLEDMCLPGKWKPKMIGGIGGFIKVRQYDQILIEICGHKVIGT
VLIGPTPVNIIGRNLLTQIGCTLNF
>187159_125 0.5
PQITLWQRPIVTVKVGGQLKEALLDTGADDTVLEDMCLPGKWKPKMIGGIGGFIKVRQYDQLLIEICGHKVIGT
VLIGPTPVNIIGRNLLTQIGCTLNF
>109422_4 0.5
PQITLWQRPLVTVRIGGQLIEALLDTGADDTVLEEMNLPGRWKPKMIGGIGGFIKVRQYDQILVEICGHKAIGT
VLIGPTPVNIIGRNLLTQIGCTLNF
>109422_5 0.5
PQITLWQRPLVTVRIGGQLIEALLDTGADDTVLEEMNLPGRWKPKMIGGIGGFIKVRQYDQILVEICGHKAIGT

VLLGPTPVNIIGRNLLTQIGCTLNF
>109422_6 0.5
PQITLWQRPLVTVRIGGQLIEALLDTGADDTVLEEMNLPGKWKPKMIGGIGGFIKVRQYDQILVEICGHKAIGTV
LIGPTPVNIIGRNLLTQIGCTLNF
>109422_7 0.5
PQITLWQRPLVTVRIGGQLIEALLDTGADDTVLEEMNLPGKWKPKMIGGIGGFIKVRQYDQILVEICGHKAIGTV
LLGPTPVNIIGRNLLTQIGCTLNF
>109422_8 0.5
PQITLWQRPLVTVRIGGQLMEALLDTGADDTVLEEMNLPGRWKPKMIGGIGGFIKVRQYDQILVEICGHKAIGT
VLIGPTPVNIIGRNLLTQIGCTLNF
>109422_9 0.5
PQITLWQRPLVTVRIGGQLMEALLDTGADDTVLEEMNLPGRWKPKMIGGIGGFIKVRQYDQILVEICGHKAIGT
VLLGPTPVNIIGRNLLTQIGCTLNF
>109422_10 0.5
PQITLWQRPLVTVRIGGQLMEALLDTGADDTVLEEMNLPGKWKPKMIGGIGGFIKVRQYDQILVEICGHKAIGT
VLIGPTPVNIIGRNLLTQIGCTLNF
>109422_11 0.5
PQITLWQRPLVTVRIGGQLMEALLDTGADDTVLEEMNLPGKWKPKMIGGIGGFIKVRQYDQILVEICGHKAIGT
VLLGPTPVNIIGRNLLTQIGCTLNF
>206413_0 0.5
PQITLWQRPLVTIKVGGQLKEALLDTGADDTVLEDMNLPGRWKPKMIGGIGGFIKVGQYDQILIEICGHKAIGT
VLVGPTPVNIIGRNLLTQIGCTLNF
>216593_0 0.4
PQITLWQRPLVTIKIGGQLKEALLDTGADDTVLEDMNLPGRWKPKMIGGIGGFIKVGQYDHILIEICGHKAIGTV
LLGPTPVNIIGRNLLTQLGCTLNF
>216593_1 0.4
PQITLWQRPLVTIKIGGQLKEALLDTGADDTVLEDMNLPGRWKPKMIGGIGGFIKVGQYDHILVEICGHKAIGT
VLLGPTPVNIIGRNLLTQLGCTLNF
>216593_2 0.4
PQITLWQRPLVTIKIGGQLKEALLDTGADDTVLEDMNLPGKWKPKMIGGIGGFIKVGQYDHILIEICGHKAIGTV
LLGPTPVNIIGRNLLTQLGCTLNF
>216593_3 0.4
PQITLWQRPLVTIKIGGQLKEALLDTGADDTVLEDMNLPGKWKPKMIGGIGGFIKVGQYDHILVEICGHKAIGTV
LLGPTPVNIIGRNLLTQLGCTLNF
>187159_3 0.5
PQITLWQRPLVTIKVGGQLKEALLDTGADDTVLEEMCLPGRWKPKMIGGIGGFIKVRQYDQIPIEICGHKVIGT
VLIGPTPVNIIGRNLLTQIGCTLNF
>187159_7 0.5
PQITLWQRPLVTIKVGGQLKEALLDTGADDTVLEEMCLPGRWKPKMIGGIGGFIKVRQYDQLPIEICGHKVIGT
VLIGPTPVNIIGRNLLTQIGCTLNF
>187159_11 0.5
PQITLWQRPLVTIKVGGQLKEALLDTGADDTVLEEMCLPGKWKPKMIGGIGGFIKVRQYDQIPIEICGHKVIGT
VLIGPTPVNIIGRNLLTQIGCTLNF
>187159_15 0.5
PQITLWQRPLVTIKVGGQLKEALLDTGADDTVLEEMCLPGKWKPKMIGGIGGFIKVRQYDQLPIEICGHKVIGT
VLIGPTPVNIIGRNLLTQIGCTLNF
>187159_19 0.5
PQITLWQRPLVTIKVGGQLKEALLDTGADDTVLEDMCLPGRWKPKMIGGIGGFIKVRQYDQIPIEICGHKVIGT
VLIGPTPVNIIGRNLLTQIGCTLNF
>187159_23 0.5
PQITLWQRPLVTIKVGGQLKEALLDTGADDTVLEDMCLPGRWKPKMIGGIGGFIKVRQYDQLPIEICGHKVIGT
VLIGPTPVNIIGRNLLTQIGCTLNF
>187159_31 0.5
PQITLWQRPLVTIKVGGQLKEALLDTGADDTVLEDMCLPGKWKPKMIGGIGGFIKVRQYDQLPIEICGHKVIGT
VLIGPTPVNIIGRNLLTQIGCTLNF
>187159_95 0.5
PQITLWQRPIVTIKVGGQLKEALLDTGADDTVLEDMCLPGKWKPKMIGGIGGFIKVRQYDQLPIEICGHKVIGT
VLIGPTPVNIIGRNLLTQIGCTLNF
>187159_103 0.5
PQITLWQRPIVTVKVGGQLKEALLDTGADDTVLEEMCLPGRWKPKMIGGIGGFIKVRQYDQLPIEICGHKVIGT
VLIGPTPVNIIGRNLLTQIGCTLNF
>187159_107 0.5
PQITLWQRPIVTVKVGGQLKEALLDTGADDTVLEEMCLPGKWKPKMIGGIGGFIKVRQYDQIPIEICGHKVIGT

VLIGPTPVNIIGRNLLTQIGCTLNF
>187159_111 0.5
PQITLWQRPIVTVKVGGQLKEALLDTGADDTVLEEMCLPGKWKPKMIGGIGGFIKVRQYDQLPIEICGHKVIGT
VLIGPTPVNIIGRNLLTQIGCTLNF
>187159_115 0.5
PQITLWQRPIVTVKVGGQLKEALLDTGADDTVLEDMCLPGRWKPKMIGGIGGFIKVRQYDQIPIEICGHKVIGT
VLIGPTPVNIIGRNLLTQIGCTLNF
>187159_119 0.5
PQITLWQRPIVTVKVGGQLKEALLDTGADDTVLEDMCLPGRWKPKMIGGIGGFIKVRQYDQLPIEICGHKVIGT
VLIGPTPVNIIGRNLLTQIGCTLNF
>187159_123 0.5
PQITLWQRPIVTVKVGGQLKEALLDTGADDTVLEDMCLPGKWKPKMIGGIGGFIKVRQYDQIPIEICGHKVIGT
VLIGPTPVNIIGRNLLTQIGCTLNF
>187159_127 0.5
PQITLWQRPIVTVKVGGQLKEALLDTGADDTVLEDMCLPGKWKPKMIGGIGGFIKVRQYDQLPIEICGHKVIGT
VLIGPTPVNIIGRNLLTQIGCTLNF
>257929_0 0.4
PQITLWQRPLVTVKVGGQLKEALLDTGADDTVLEEMNLQGRWKPKMIGGIGGFIKVKQYDQIPVEICGHKAIG
TVLVGPTPVNIIGRNLLTQIGCTLNF
>187159_14 0.5
PQITLWQRPLVTIKVGGQLKEALLDTGADDTVLEEMCLPGKWKPKMIGGIGGFIKVRQYDQLPIEICGHKAIGT
VLIGPTPVNIIGRNLLTQIGCTLNF
>187159_18 0.5
PQITLWQRPLVTIKVGGQLKEALLDTGADDTVLEDMCLPGRWKPKMIGGIGGFIKVRQYDQIPIEICGHKAIGT
VLIGPTPVNIIGRNLLTQIGCTLNF
>187159_22 0.5
PQITLWQRPLVTIKVGGQLKEALLDTGADDTVLEDMCLPGRWKPKMIGGIGGFIKVRQYDQLPIEICGHKAIGT
VLIGPTPVNIIGRNLLTQIGCTLNF
>187159_26 0.5
PQITLWQRPLVTIKVGGQLKEALLDTGADDTVLEDMCLPGKWKPKMIGGIGGFIKVRQYDQIPIEICGHKAIGT
VLIGPTPVNIIGRNLLTQIGCTLNF
>187159_30 0.5
PQITLWQRPLVTIKVGGQLKEALLDTGADDTVLEDMCLPGKWKPKMIGGIGGFIKVRQYDQLPIEICGHKAIGT
VLIGPTPVNIIGRNLLTQIGCTLNF
>187159_34 0.5
PQITLWQRPLVTVKVGGQLKEALLDTGADDTVLEEMCLPGRWKPKMIGGIGGFIKVRQYDQIPIEICGHKAIGT
VLIGPTPVNIIGRNLLTQIGCTLNF
>187159_38 0.5
PQITLWQRPLVTVKVGGQLKEALLDTGADDTVLEEMCLPGRWKPKMIGGIGGFIKVRQYDQLPIEICGHKAIG
TVLIGPTPVNIIGRNLLTQIGCTLNF
>187159_42 0.5
PQITLWQRPLVTVKVGGQLKEALLDTGADDTVLEEMCLPGKWKPKMIGGIGGFIKVRQYDQIPIEICGHKAIGT
VLIGPTPVNIIGRNLLTQIGCTLNF
>187159_46 0.5
PQITLWQRPLVTVKVGGQLKEALLDTGADDTVLEEMCLPGKWKPKMIGGIGGFIKVRQYDQLPIEICGHKAIG
TVLIGPTPVNIIGRNLLTQIGCTLNF
>187159_50 0.5
PQITLWQRPLVTVKVGGQLKEALLDTGADDTVLEDMCLPGRWKPKMIGGIGGFIKVRQYDQIPIEICGHKAIGT
VLIGPTPVNIIGRNLLTQIGCTLNF
>187159_54 0.5
PQITLWQRPLVTVKVGGQLKEALLDTGADDTVLEDMCLPGRWKPKMIGGIGGFIKVRQYDQLPIEICGHKAIG
TVLIGPTPVNIIGRNLLTQIGCTLNF
>187159_58 0.5
PQITLWQRPLVTVKVGGQLKEALLDTGADDTVLEDMCLPGKWKPKMIGGIGGFIKVRQYDQIPIEICGHKAIGT
VLIGPTPVNIIGRNLLTQIGCTLNF
>187159_66 0.5
PQITLWQRPIVTIKVGGQLKEALLDTGADDTVLEEMCLPGRWKPKMIGGIGGFIKVRQYDQIPIEICGHKAIGTV
LIGPTPVNIIGRNLLTQIGCTLNF
>187159_70 0.5
PQITLWQRPIVTIKVGGQLKEALLDTGADDTVLEEMCLPGRWKPKMIGGIGGFIKVRQYDQLPIEICGHKAIGT
VLIGPTPVNIIGRNLLTQIGCTLNF
>187159_98 0.5
PQITLWQRPIVTVKVGGQLKEALLDTGADDTVLEEMCLPGRWKPKMIGGIGGFIKVRQYDQIPIEICGHKAIGT

VLIGPTPVNIIGRNLLTQIGCTLNF
>187159_102 0.5
PQITLWQRPIVTVKVGGQLKEALLDTGADDTVLEEMCLPGRWKPKMIGGIGGFIKVRQYDQLPIEICGHKAIGT
VLIGPTPVNIIGRNLLTQIGCTLNF
>187159_106 0.5
PQITLWQRPIVTVKVGGQLKEALLDTGADDTVLEEMCLPGKWKPKMIGGIGGFIKVRQYDQIPIEICGHKAIGT
VLIGPTPVNIIGRNLLTQIGCTLNF
>187159_110 0.5
PQITLWQRPIVTVKVGGQLKEALLDTGADDTVLEEMCLPGKWKPKMIGGIGGFIKVRQYDQLPIEICGHKAIGT
VLIGPTPVNIIGRNLLTQIGCTLNF
>187159_114 0.5
PQITLWQRPIVTVKVGGQLKEALLDTGADDTVLEDMCLPGRWKPKMIGGIGGFIKVRQYDQIPIEICGHKAIGT
VLIGPTPVNIIGRNLLTQIGCTLNF
>187159_118 0.5
PQITLWQRPIVTVKVGGQLKEALLDTGADDTVLEDMCLPGRWKPKMIGGIGGFIKVRQYDQLPIEICGHKAIGT
VLIGPTPVNIIGRNLLTQIGCTLNF
>187159_122 0.5
PQITLWQRPIVTVKVGGQLKEALLDTGADDTVLEDMCLPGKWKPKMIGGIGGFIKVRQYDQIPIEICGHKAIGT
VLIGPTPVNIIGRNLLTQIGCTLNF
>187159_126 0.5
PQITLWQRPIVTVKVGGQLKEALLDTGADDTVLEDMCLPGKWKPKMIGGIGGFIKVRQYDQLPIEICGHKAIGT
VLIGPTPVNIIGRNLLTQIGCTLNF
>87104_0 0.4
PQITLWQRPLVAIKIGGQLKEALLDTGADDTVLEEINLPGRWKPKMIGGIGGFIKVKQYDQVPIEICGHKAIGTV
LVGPTPVNIIGRNLLTQLGCTLNF
>87104_1 0.4
PQITLWQRPLVAIRIGGQLKEALLDTGADDTVLEEINLPGRWKPKMIGGIGGFIKVKQYDQVPIEICGHKAIGTV
LVGPTPVNIIGRNLLTQLGCTLNF
>198533_0 0.5
PQITLWQRPLVTIKIGGQLKEALLDTGADDTVLEEMELPGRWKPKMIGGIGGFIKVRQYDQIPIEICGHTARGT
VLVGPTPVNIIGRNLLTQIGCTLNF
>148111_0 0.3
PQITLWQRPLVTVKIGGQLKEALLDTGADDTVLEEMNLPGRWKPRMIGGIGGFIKVRQYDQISMEICGHKAIG
TVLIGPTPVNIIGRNLLTQIGCTLNF
>148111_1 0.3
PQITLWQRPLVTVKIGGQLKEALLDTGADDTVLEEMNLPGRWKPRMIGGIGGFIKVRQYDQVSMEICGHKAIG
TVLIGPTPVNIIGRNLLTQIGCTLNF
>198527_0 0.4
PQITLWQRPLVTIKIGGQLKEALLDTGADDTVLEEMNLPGRWKPKMIGGIGGFIKVRQYDEVTIDICGHKAIGT
VLIGPTPVNIIGRNLLTQIGCTLNF

Sequences for the DRV-resistant ensemble
>205693_6 580.0
PQITLWQRPFIPVKIGGQQMEALLDTGADDTILEGTNLPGRWTPKIVGGIGGFMRVGQYDQIPIEICGQKIIGTV
LVGPTPANIIGRNVMTQIGCTLNF
>205693_7 580.0
PQITLWQRPFIPVKIGGQQMEALLDTGADDTILEGTNLPGRWTPKIVGGIGGFMRVGQYDQIPIEIYGQKIIGTV
LVGPTPANIIGRNVMTQIGCTLNF
>205693_2 580.0
PQITLWQRPFIPVKIGGQQMEALLDTGADDTILEETNLPGRWTPKIVGGIGGFMRVGQYDQIPIEICGQKIIGTV
LVGPTPANIIGRNVMTQIGCTLNF
>205693_3 580.0
PQITLWQRPFIPVKIGGQQMEALLDTGADDTILEETNLPGRWTPKIVGGIGGFMRVGQYDQIPIEIYGQKIIGTV
LVGPTPANIIGRNVMTQIGCTLNF
>205693_4 580.0
PQITLWQRPFIPVKIGGQQMEALLDTGADDTILEGINLPGRWTPKIVGGIGGFMRVGQYDQIPIEICGQKIIGTV
LVGPTPANIIGRNVMTQIGCTLNF
>205693_5 580.0
PQITLWQRPFIPVKIGGQQMEALLDTGADDTILEGINLPGRWTPKIVGGIGGFMRVGQYDQIPIEIYGQKIIGTV
LVGPTPANIIGRNVMTQIGCTLNF
>205693_0 580.0
PQITLWQRPFIPVKIGGQQMEALLDTGADDTILEEINLPGRWTPKIVGGIGGFMRVGQYDQIPIEICGQKIIGTVL
VGPTPANIIGRNVMTQIGCTLNF

>205693_1 580.0
PQITLWQRPFIPVKIGGQQMEALLDTGADDTILEEINLPGRWTPKIVGGIGGFMRVGQYDQIPIEIYGQKIIGTVL
VGPTPANIIGRNVMTQIGCTLNF
>117075_0 500.0
PQITLWQRPLITVKIGGQLVEALMDTGADDTIFEGINLPGRWKPKMIGGIGGFLNVRQYEQIPIEICGHKILSTVL
VGSTPLNVIGRNVMSKIGCTLNF
>117075_1 500.0
PQITLWQRPLITVKIGGQLVEALMDTGADDTIFEGINLPGRWKPKMIGGIGGFLNVREYEQIPIEICGHKILSTVL
VGSTPLNVIGRNVMSKIGCTLNF
>117075_2 500.0
PQITLWQRPLITVKVGGQLVEALMDTGADDTIFEGINLPGRWKPKMIGGIGGFLNVRQYEQIPIEICGHKILSTV
LVGSTPLNVIGRNVMSKIGCTLNF
>117075_3 500.0
PQITLWQRPLITVKVGGQLVEALMDTGADDTIFEGINLPGRWKPKMIGGIGGFLNVREYEQIPIEICGHKILSTV
LVGSTPLNVIGRNVMSKIGCTLNF
>117075_4 500.0
PQITLWQRPFITVKIGGQLVEALMDTGADDTIFEGINLPGRWKPKMIGGIGGFLNVRQYEQIPIEICGHKILSTVL
VGSTPLNVIGRNVMSKIGCTLNF
>117075_5 500.0
PQITLWQRPFITVKIGGQLVEALMDTGADDTIFEGINLPGRWKPKMIGGIGGFLNVREYEQIPIEICGHKILSTVL
VGSTPLNVIGRNVMSKIGCTLNF
>117075_6 500.0
PQITLWQRPFITVKVGGQLVEALMDTGADDTIFEGINLPGRWKPKMIGGIGGFLNVRQYEQIPIEICGHKILSTV
LVGSTPLNVIGRNVMSKIGCTLNF
>117075_7 500.0
PQITLWQRPFITVKVGGQLVEALMDTGADDTIFEGINLPGRWKPKMIGGIGGFLNVREYEQIPIEICGHKILSTV
LVGSTPLNVIGRNVMSKIGCTLNF
>117075_8 500.0
PQITLWQRPVITVKIGGQLVEALMDTGADDTIFEGINLPGRWKPKMIGGIGGFLNVRQYEQIPIEICGHKILSTVL
VGSTPLNVIGRNVMSKIGCTLNF
>117075_9 500.0
PQITLWQRPVITVKIGGQLVEALMDTGADDTIFEGINLPGRWKPKMIGGIGGFLNVREYEQIPIEICGHKILSTVL
VGSTPLNVIGRNVMSKIGCTLNF
>117075_10 500.0
PQITLWQRPVITVKVGGQLVEALMDTGADDTIFEGINLPGRWKPKMIGGIGGFLNVRQYEQIPIEICGHKILSTV
LVGSTPLNVIGRNVMSKIGCTLNF
>117075_11 500.0
PQITLWQRPVITVKVGGQLVEALMDTGADDTIFEGINLPGRWKPKMIGGIGGFLNVREYEQIPIEICGHKILSTV
LVGSTPLNVIGRNVMSKIGCTLNF
>103779_0 500.0
PQITLWQRPIIKVKIGGQLVEALLDTGADDTILEGINLPGRWKPKIVGGIGGFMKVKEYDQIPVEICGHKIISTVLV
GPTPVNIIGRNVMTQIGCTLNF
>103779_1 500.0
PQITLWQRPIIKVKIGGQLVEALLDTGADDTILEGINLPGRWKPKIVGGIGGFMKVKEYDQIPVEICGHKIISTVLV
GPTPVNVIGRNVMTQIGCTLNF
>103779_2 500.0
PQITLWQRPIIKVKIGGQLVEALLDTGADDTILEGINLPGRWKPKIVGGIGGFMKVKEYDQIPVEICGHKVISTVL
VGPTPVNIIGRNVMTQIGCTLNF
>103779_3 500.0
PQITLWQRPIIKVKIGGQLVEALLDTGADDTILEGINLPGRWKPKIVGGIGGFMKVKEYDQIPVEICGHKVISTVL
VGPTPVNVIGRNVMTQIGCTLNF
>103779_4 500.0
PQITLWQRPIIKVKIGGQLVEALLDTGADDTILEGIDLPGRWKPKIVGGIGGFMKVKEYDQIPVEICGHKIISTVLV
GPTPVNIIGRNVMTQIGCTLNF
>103779_5 500.0
PQITLWQRPIIKVKIGGQLVEALLDTGADDTILEGIDLPGRWKPKIVGGIGGFMKVKEYDQIPVEICGHKIISTVLV
GPTPVNVIGRNVMTQIGCTLNF
>103779_6 500.0
PQITLWQRPIIKVKIGGQLVEALLDTGADDTILEGIDLPGRWKPKIVGGIGGFMKVKEYDQIPVEICGHKVISTVL
VGPTPVNIIGRNVMTQIGCTLNF
>103779_7 500.0
PQITLWQRPIIKVKIGGQLVEALLDTGADDTILEGIDLPGRWKPKIVGGIGGFMKVKEYDQIPVEICGHKVISTVL
VGPTPVNVIGRNVMTQIGCTLNF

>103779_8 500.0
PQITLWQRPIIKVKIGGQLVEALLDTGADDTIFEGINLPGRWKPKIVGGIGGFMKVKEYDQIPVEICGHKIISTVL
VGPTPVNIIGRNVMTQIGCTLNF
>103779_9 500.0
PQITLWQRPIIKVKIGGQLVEALLDTGADDTIFEGINLPGRWKPKIVGGIGGFMKVKEYDQIPVEICGHKIISTVL
VGPTPVNVIGRNVMTQIGCTLNF
>103779_10 500.0
PQITLWQRPIIKVKIGGQLVEALLDTGADDTIFEGINLPGRWKPKIVGGIGGFMKVKEYDQIPVEICGHKVISTVL
VGPTPVNIIGRNVMTQIGCTLNF
>103779_11 500.0
PQITLWQRPIIKVKIGGQLVEALLDTGADDTIFEGINLPGRWKPKIVGGIGGFMKVKEYDQIPVEICGHKVISTVL
VGPTPVNVIGRNVMTQIGCTLNF
>103779_12 500.0
PQITLWQRPIIKVKIGGQLVEALLDTGADDTIFEGIDLPGRWKPKIVGGIGGFMKVKEYDQIPVEICGHKIISTVL
VGPTPVNIIGRNVMTQIGCTLNF
>103779_13 500.0
PQITLWQRPIIKVKIGGQLVEALLDTGADDTIFEGIDLPGRWKPKIVGGIGGFMKVKEYDQIPVEICGHKIISTVL
VGPTPVNVIGRNVMTQIGCTLNF
>103779_14 500.0
PQITLWQRPIIKVKIGGQLVEALLDTGADDTIFEGIDLPGRWKPKIVGGIGGFMKVKEYDQIPVEICGHKVISTVL
VGPTPVNIIGRNVMTQIGCTLNF
>103779_15 500.0
PQITLWQRPIIKVKIGGQLVEALLDTGADDTIFEGIDLPGRWKPKIVGGIGGFMKVKEYDQIPVEICGHKVISTVL
VGPTPVNVIGRNVMTQIGCTLNF
>147550_0 500.0
PQITLWQRPIIKVKIGGQLVEALLDTGADDTIFEGIDLPGRWKPKIVGGIGGFMKVKEYDQIPVEVCGHKVISTV
LVGPTPVNVIGRNVMTQIGCTLNF
>86742_0 500.0
PQITLWQRPFITIKIGGQLREALLDTGADDTIFEDIQLPGRWKPKIVGGIGGFLKVKEYEQVPIEICGHKVISTVL
TGPTPVNVIGRNVMTQLGCTLNF
>86742_1 500.0
PQITLWQRPFITVKIGGQLREALLDTGADDTIFEDIQLPGRWKPKIVGGIGGFLKVKEYEQVPIEICGHKVISTVL
TGPTPVNVIGRNVMTQLGCTLNF
>103067_0 500.0
PQITLWQRPFITVKIAGQLKEALLDTGADDTVFQEMNLPGRWIPKLIGGIAGFMKVRQYDQIPMEIYGHKVMAT
VLVGPTPVNVIGRNLMTQIGCTLNF
>103067_1 500.0
PQITLWQRPFITVKIAGQVKEALLDTGADDTVFQEMNLPGRWIPKLIGGIAGFMKVRQYDQIPMEIYGHKVMA
TVLVGPTPVNVIGRNLMTQIGCTLNF
>90022_35 491.0
PQITLWQRPFVAVKIGGQLREALLDTGADNTIFEDVTLPGRWQPKIVGGIGGLLKVKQYDQVPIEIWGHKVVG
TVLIGPTPVNVLGRDVMTQIGCTLNF
>90022_36 491.0
PQITLWQRPFVAVKIGGQLREALLDTGADNTIFEDVTLPGRWQPKIVGGIGGLLKVKQYDQVPIEVWGHKVVG
TVLIGPTPVNVIGRDLMTQIGCTLNF
>90022_37 491.0
PQITLWQRPFVAVKIGGQLREALLDTGADNTIFEDVTLPGRWQPKIVGGIGGLLKVKQYDQVPIEVWGHKVVG
TVLIGPTPVNVIGRDVMTQIGCTLNF
>90022_38 491.0
PQITLWQRPFVAVKIGGQLREALLDTGADNTIFEDVTLPGRWQPKIVGGIGGLLKVKQYDQVPIEVWGHKVVG
TVLIGPTPVNVLGRDLMTQIGCTLNF
>90022_39 491.0
PQITLWQRPFVAVKIGGQLREALLDTGADNTIFEDVTLPGRWQPKIVGGIGGLLKVKQYDQVPIEVWGHKVVG
TVLIGPTPVNVLGRDVMTQIGCTLNF
>90022_40 491.0
PQITLWQRPFVAVKIGGQLREALLDTGADNTIFEDVTLPGRWQPKIVGGIGGLLRVKQYDQVPIEIWGHKVVG
TVLIGPTPVNVIGRDLMTQIGCTLNF
>90022_41 491.0
PQITLWQRPFVAVKIGGQLREALLDTGADNTIFEDVTLPGRWQPKIVGGIGGLLRVKQYDQVPIEIWGHKVVG
TVLIGPTPVNVIGRDVMTQIGCTLNF
>90022_42 491.0
PQITLWQRPFVAVKIGGQLREALLDTGADNTIFEDVTLPGRWQPKIVGGIGGLLRVKQYDQVPIEIWGHKVVG
TVLIGPTPVNVLGRDLMTQIGCTLNF

>90022_43 491.0
PQITLWQRPFVAVKIGGQLREALLDTGADNTIFEDVTLPGRWQPKIVGGIGGLLRVKQYDQVPIEIWGHKVVG
TVLIGPTPVNVLGRDVMTQIGCTLNF
>90022_44 491.0
PQITLWQRPFVAVKIGGQLREALLDTGADNTIFEDVTLPGRWQPKIVGGIGGLLRVKQYDQVPIEVWGHKVVG
TVLIGPTPVNVIGRDLMTQIGCTLNF
>90022_45 491.0
PQITLWQRPFVAVKIGGQLREALLDTGADNTIFEDVTLPGRWQPKIVGGIGGLLRVKQYDQVPIEVWGHKVVG
TVLIGPTPVNVIGRDVMTQIGCTLNF
>90022_46 491.0
PQITLWQRPFVAVKIGGQLREALLDTGADNTIFEDVTLPGRWQPKIVGGIGGLLRVKQYDQVPIEVWGHKVVG
TVLIGPTPVNVLGRDLMTQIGCTLNF
>90022_47 491.0
PQITLWQRPFVAVKIGGQLREALLDTGADNTIFEDVTLPGRWQPKIVGGIGGLLRVKQYDQVPIEVWGHKVVG
TVLIGPTPVNVLGRDVMTQIGCTLNF
>90022_48 491.0
PQITLWQRPFVAVRIGGQLREALLDTGADNTIFEDVTLPGRWQPKIVGGIGGLLKVKQYDQVPIEIWGHKVVG
TVLIGPTPVNVIGRDLMTQIGCTLNF
>90022_49 491.0
PQITLWQRPFVAVRIGGQLREALLDTGADNTIFEDVTLPGRWQPKIVGGIGGLLKVKQYDQVPIEIWGHKVVG
TVLIGPTPVNVIGRDVMTQIGCTLNF
>90022_50 491.0
PQITLWQRPFVAVRIGGQLREALLDTGADNTIFEDVTLPGRWQPKIVGGIGGLLKVKQYDQVPIEIWGHKVVG
TVLIGPTPVNVLGRDLMTQIGCTLNF
>90022_51 491.0
PQITLWQRPFVAVRIGGQLREALLDTGADNTIFEDVTLPGRWQPKIVGGIGGLLKVKQYDQVPIEIWGHKVVG
TVLIGPTPVNVLGRDVMTQIGCTLNF
>90022_52 491.0
PQITLWQRPFVAVRIGGQLREALLDTGADNTIFEDVTLPGRWQPKIVGGIGGLLKVKQYDQVPIEVWGHKVVG
TVLIGPTPVNVIGRDLMTQIGCTLNF
>90022_53 491.0
PQITLWQRPFVAVRIGGQLREALLDTGADNTIFEDVTLPGRWQPKIVGGIGGLLKVKQYDQVPIEVWGHKVVG
TVLIGPTPVNVIGRDVMTQIGCTLNF
>90022_54 491.0
PQITLWQRPFVAVRIGGQLREALLDTGADNTIFEDVTLPGRWQPKIVGGIGGLLKVKQYDQVPIEVWGHKVVG
TVLIGPTPVNVLGRDLMTQIGCTLNF
>90022_55 491.0
PQITLWQRPFVAVRIGGQLREALLDTGADNTIFEDVTLPGRWQPKIVGGIGGLLKVKQYDQVPIEVWGHKVVG
TVLIGPTPVNVLGRDVMTQIGCTLNF
>90022_56 491.0
PQITLWQRPFVAVRIGGQLREALLDTGADNTIFEDVTLPGRWQPKIVGGIGGLLRVKQYDQVPIEIWGHKVVG
TVLIGPTPVNVIGRDLMTQIGCTLNF
>90022_57 491.0
PQITLWQRPFVAVRIGGQLREALLDTGADNTIFEDVTLPGRWQPKIVGGIGGLLRVKQYDQVPIEIWGHKVVG
TVLIGPTPVNVIGRDVMTQIGCTLNF
>90022_58 491.0
PQITLWQRPFVAVRIGGQLREALLDTGADNTIFEDVTLPGRWQPKIVGGIGGLLRVKQYDQVPIEIWGHKVVG
TVLIGPTPVNVLGRDLMTQIGCTLNF
>90022_59 491.0
PQITLWQRPFVAVRIGGQLREALLDTGADNTIFEDVTLPGRWQPKIVGGIGGLLRVKQYDQVPIEIWGHKVVG
TVLIGPTPVNVLGRDVMTQIGCTLNF
>90022_60 491.0
PQITLWQRPFVAVRIGGQLREALLDTGADNTIFEDVTLPGRWQPKIVGGIGGLLRVKQYDQVPIEVWGHKVVG
TVLIGPTPVNVIGRDLMTQIGCTLNF
>90022_61 491.0
PQITLWQRPFVAVRIGGQLREALLDTGADNTIFEDVTLPGRWQPKIVGGIGGLLRVKQYDQVPIEVWGHKVVG
TVLIGPTPVNVIGRDVMTQIGCTLNF
>90022_62 491.0
PQITLWQRPFVAVRIGGQLREALLDTGADNTIFEDVTLPGRWQPKIVGGIGGLLRVKQYDQVPIEVWGHKVVG
TVLIGPTPVNVLGRDLMTQIGCTLNF
>90022_96 491.0
PQITLWQRPFIAVKIGGQLREALLDTGADNTIFEDVTLPGRWQPKIVGGIGGLLKVKQYDQVPIEIWGHKVVGT
VLIGPTPVNVIGRDLMTQIGCTLNF

>90022_97 491.0
PQITLWQRPFIAVKIGGQLREALLDTGADNTIFEDVTLPGRWQPKIVGGIGGLLKVKQYDQVPIEIWGHKVVGT
VLIGPTPVNVIGRDVMTQIGCTLNF
>90022_98 491.0
PQITLWQRPFIAVKIGGQLREALLDTGADNTIFEDVTLPGRWQPKIVGGIGGLLKVKQYDQVPIEIWGHKVVGT
VLIGPTPVNVLGRDLMTQIGCTLNF
>90022_99 491.0
PQITLWQRPFIAVKIGGQLREALLDTGADNTIFEDVTLPGRWQPKIVGGIGGLLKVKQYDQVPIEIWGHKVVGT
VLIGPTPVNVLGRDVMTQIGCTLNF
>90022_100 491.0
PQITLWQRPFIAVKIGGQLREALLDTGADNTIFEDVTLPGRWQPKIVGGIGGLLKVKQYDQVPIEVWGHKVVG
TVLIGPTPVNVIGRDLMTQIGCTLNF
>90022_101 491.0
PQITLWQRPFIAVKIGGQLREALLDTGADNTIFEDVTLPGRWQPKIVGGIGGLLKVKQYDQVPIEVWGHKVVG
TVLIGPTPVNVIGRDVMTQIGCTLNF
>90022_102 491.0
PQITLWQRPFIAVKIGGQLREALLDTGADNTIFEDVTLPGRWQPKIVGGIGGLLKVKQYDQVPIEVWGHKVVG
TVLIGPTPVNVLGRDLMTQIGCTLNF
>90022_103 491.0
PQITLWQRPFIAVKIGGQLREALLDTGADNTIFEDVTLPGRWQPKIVGGIGGLLKVKQYDQVPIEVWGHKVVG
TVLIGPTPVNVLGRDVMTQIGCTLNF
>90022_104 491.0
PQITLWQRPFIAVKIGGQLREALLDTGADNTIFEDVTLPGRWQPKIVGGIGGLLRVKQYDQVPIEIWGHKVVGT
VLIGPTPVNVIGRDLMTQIGCTLNF
>90022_105 491.0
PQITLWQRPFIAVKIGGQLREALLDTGADNTIFEDVTLPGRWQPKIVGGIGGLLRVKQYDQVPIEIWGHKVVGT
VLIGPTPVNVIGRDVMTQIGCTLNF
>90022_106 491.0
PQITLWQRPFIAVKIGGQLREALLDTGADNTIFEDVTLPGRWQPKIVGGIGGLLRVKQYDQVPIEIWGHKVVGT
VLIGPTPVNVLGRDLMTQIGCTLNF
>90022_107 491.0
PQITLWQRPFIAVKIGGQLREALLDTGADNTIFEDVTLPGRWQPKIVGGIGGLLRVKQYDQVPIEIWGHKVVGT
VLIGPTPVNVLGRDVMTQIGCTLNF
>90022_108 491.0
PQITLWQRPFIAVKIGGQLREALLDTGADNTIFEDVTLPGRWQPKIVGGIGGLLRVKQYDQVPIEVWGHKVVG
TVLIGPTPVNVIGRDLMTQIGCTLNF
>90022_109 491.0
PQITLWQRPFIAVKIGGQLREALLDTGADNTIFEDVTLPGRWQPKIVGGIGGLLRVKQYDQVPIEVWGHKVVG
TVLIGPTPVNVIGRDVMTQIGCTLNF
>90022_110 491.0
PQITLWQRPFIAVKIGGQLREALLDTGADNTIFEDVTLPGRWQPKIVGGIGGLLRVKQYDQVPIEVWGHKVVG
TVLIGPTPVNVLGRDLMTQIGCTLNF
>90022_111 491.0
PQITLWQRPFIAVKIGGQLREALLDTGADNTIFEDVTLPGRWQPKIVGGIGGLLRVKQYDQVPIEVWGHKVVG
TVLIGPTPVNVLGRDVMTQIGCTLNF
>90022_112 491.0
PQITLWQRPFIAVRIGGQLREALLDTGADNTIFEDVTLPGRWQPKIVGGIGGLLKVKQYDQVPIEIWGHKVVGT
VLIGPTPVNVIGRDLMTQIGCTLNF
>90022_113 491.0
PQITLWQRPFIAVRIGGQLREALLDTGADNTIFEDVTLPGRWQPKIVGGIGGLLKVKQYDQVPIEIWGHKVVGT
VLIGPTPVNVIGRDVMTQIGCTLNF
>90022_114 491.0
PQITLWQRPFIAVRIGGQLREALLDTGADNTIFEDVTLPGRWQPKIVGGIGGLLKVKQYDQVPIEIWGHKVVGT
VLIGPTPVNVLGRDLMTQIGCTLNF
>90022_115 491.0
PQITLWQRPFIAVRIGGQLREALLDTGADNTIFEDVTLPGRWQPKIVGGIGGLLKVKQYDQVPIEIWGHKVVGT
VLIGPTPVNVLGRDVMTQIGCTLNF
>90022_116 491.0
PQITLWQRPFIAVRIGGQLREALLDTGADNTIFEDVTLPGRWQPKIVGGIGGLLKVKQYDQVPIEVWGHKVVG
TVLIGPTPVNVIGRDLMTQIGCTLNF
>90022_117 491.0
PQITLWQRPFIAVRIGGQLREALLDTGADNTIFEDVTLPGRWQPKIVGGIGGLLKVKQYDQVPIEVWGHKVVG
TVLIGPTPVNVIGRDVMTQIGCTLNF

>90022_118 491.0
PQITLWQRPFIAVRIGGQLREALLDTGADNTIFEDVTLPGRWQPKIVGGIGGLLKVKQYDQVPIEVWGHKVVG
TVLIGPTPVNVLGRDLMTQIGCTLNF
>90022_119 491.0
PQITLWQRPFIAVRIGGQLREALLDTGADNTIFEDVTLPGRWQPKIVGGIGGLLKVKQYDQVPIEVWGHKVVG
TVLIGPTPVNVLGRDVMTQIGCTLNF
>90022_120 491.0
PQITLWQRPFIAVRIGGQLREALLDTGADNTIFEDVTLPGRWQPKIVGGIGGLLRVKQYDQVPIEIWGHKVVGT
VLIGPTPVNVIGRDLMTQIGCTLNF
>117069_0 500.0
PQITLWQRPILTVKIAGQLTEALLDTGADDTIFQDINLPGRWTPKIVGGIGGLLRVRQYEQVPIEVCGHKIITTVLI
GPTPVNVLGRNLMTQLGCTLNF
>117069_1 500.0
PQITLWQRPILTVKVAGQLTEALLDTGADDTIFQDINLPGRWTPKIVGGIGGLLRVRQYEQVPIEVCGHKIITTVL
IGPTPVNVLGRNLMTQLGCTLNF
>90022_12 491.0
PQITLWQRPFVTVKIGGQLREALLDTGADNTIFEDVTLPGRWQPKIVGGIGGLLRVKQYDQVPIEVWGHKVV
GTVLIGPTPVNVIGRDLMTQIGCTLNF
>90022_22 491.0
PQITLWQRPFVTVRIGGQLREALLDTGADNTIFEDVTLPGRWQPKIVGGIGGLLKVKQYDQVPIEVWGHKVV
GTVLIGPTPVNVLGRDLMTQIGCTLNF
>90022_23 491.0
PQITLWQRPFVTVRIGGQLREALLDTGADNTIFEDVTLPGRWQPKIVGGIGGLLKVKQYDQVPIEVWGHKVV
GTVLIGPTPVNVLGRDVMTQIGCTLNF
>90022_65 491.0
PQITLWQRPFITVKIGGQLREALLDTGADNTIFEDVTLPGRWQPKIVGGIGGLLKVKQYDQVPIEIWGHKVVGT
VLIGPTPVNVIGRDVMTQIGCTLNF

Sequences for the DRV-susceptible ensemble
>216607_0 0.2
PQITLWQRPLVTIKIGGQLKEALLDTGADDTVLEEMNLPGRWKPRMIGGIGGFIKVGQYDQMPIEICGHKTEG
TVLVGPTPVNIIGRNLLTQIGCTLNF
>216607_1 0.2
PQITLWQRPLVTIKIGGQLKEALLDTGADDTVLEEMNLPGRWKPRMIGGIGGFIKVGQYDQMPIEICGHKTEG
TVLVGPTPVNIIGRNLLTQMGCTLNF
>216607_2 0.2
PQITLWQRPLVTIKIGGQLKEALLDTGADDTVLEEINLPGRWKPRMIGGIGGFIKVGQYDQMPIEICGHKTEGT
VLVGPTPVNIIGRNLLTQIGCTLNF
>216607_3 0.2
PQITLWQRPLVTIKIGGQLKEALLDTGADDTVLEEINLPGRWKPRMIGGIGGFIKVGQYDQMPIEICGHKTEGT
VLVGPTPVNIIGRNLLTQMGCTLNF
>116920_3 0.2
PQITLWQRPLVTIKIGGQLKEALLDTGADDTVLEEMNLPGRWKPRMIGGLGGFIKVRQYDQVPIEICGHKTVS
TVLIGPTPANIIGRNLLTQLGCTLNF
>116920_1 0.2
PQITLWQRPLVTIKIGGQLKEALLDTGADDTVLEEMNLPGRWKPRMIGGLGGFIKVRQYDQVPIEICGHKIVST
VLIGPTPANIIGRNLLTQLGCTLNF
>258503_0 0.3
PQITLWQRPLVTIKIGGQLKEAILDTGADDTVLEEMNLPGRWKPKMIGGIGGFIKVKQYDQIPIEICGYKVIGTVL
VGPTPVNIIGRSLLTRLGCTLNF
>116920_2 0.2
PQITLWQRPLVTIKIGGQLKEALLDTGADDTVLEEMNLPGRWKPRMIGGLGGFIKVRQYDQVPIEICGHKTVG
TVLIGPTPANIIGRNLLTQLGCTLNF
>87104_0 0.4
PQITLWQRPLVAIKIGGQLKEALLDTGADDTVLEEINLPGRWKPKMIGGIGGFIKVKQYDQVPIEICGHKAIGTV
LVGPTPVNIIGRNLLTQLGCTLNF
>206413_0 0.4
PQITLWQRPLVTIKVGGQLKEALLDTGADDTVLEDMNLPGRWKPKMIGGIGGFIKVGQYDQILIEICGHKAIGT
VLVGPTPVNIIGRNLLTQIGCTLNF
>216593_0 0.3
PQITLWQRPLVTIKIGGQLKEALLDTGADDTVLEDMNLPGRWKPKMIGGIGGFIKVGQYDHILIEICGHKAIGTV
LLGPTPVNIIGRNLLTQLGCTLNF
>216593_1 0.3

PQITLWQRPLVTIKIGGQLKEALLDTGADDTVLEDMNLPGRWKPKMIGGIGGFIKVGQYDHILVEICGHKAIGT
VLLGPTPVNIIGRNLLTQLGCTLNF
>216593_2 0.3
PQITLWQRPLVTIKIGGQLKEALLDTGADDTVLEDMNLPGKWKPKMIGGIGGFIKVGQYDHILIEICGHKAIGTV
LLGPTPVNIIGRNLLTQLGCTLNF
>216593_3 0.3
PQITLWQRPLVTIKIGGQLKEALLDTGADDTVLEDMNLPGKWKPKMIGGIGGFIKVGQYDHILVEICGHKAIGTV
LLGPTPVNIIGRNLLTQLGCTLNF
>216597_0 0.4
PQITLWQRPLVTIKIGGQLKEALLDTGADDTVLEEMDIPGRWKPKMIGGIGGFIKVGQYDQVLIEICGHKAIGTV
LVGPTPVNIIGRNLLTQIGCTLNF
>216597_1 0.4
PQITLWQRPLVTIKIGGQLKEALLDTGADDTVLEEMDIPGRWKPKMIGGIGGFIKVGQYDQVLIEICGHKAIGTV
LIGPTPVNIIGRNLLTQIGCTLNF
>216597_2 0.4
PQITLWQRPLVTIKIGGQLKEALLDTGADDTVLEEMDIPGRWKPKMIGGIGGFIKVGQYDQVLIEICGHKAMGT
VLVGPTPVNIIGRNLLTQIGCTLNF
>216597_3 0.4
PQITLWQRPLVTIKIGGQLKEALLDTGADDTVLEEMDIPGRWKPKMIGGIGGFIKVGQYDQVLIEICGHKAMGT
VLIGPTPVNIIGRNLLTQIGCTLNF
>216597_4 0.4
PQITLWQRPLVTIKIGGQLKEALLDTGADDTVLEEMDIPGRWKPKMIGGIGGFIKVGQYDQVLIEICGHKAVGT
VLVGPTPVNIIGRNLLTQIGCTLNF
>216597_5 0.4
PQITLWQRPLVTIKIGGQLKEALLDTGADDTVLEEMDIPGRWKPKMIGGIGGFIKVGQYDQVLIEICGHKAVGT
VLIGPTPVNIIGRNLLTQIGCTLNF
>216597_12 0.4
PQITLWQRPLVTIKIGGQLKEALLDTGADDTVLEEMDIPGRWKPKMIGGIGGFIKVGQYDQVLVEICGHKAIGT
VLVGPTPVNIIGRNLLTQIGCTLNF
>216597_13 0.4
PQITLWQRPLVTIKIGGQLKEALLDTGADDTVLEEMDIPGRWKPKMIGGIGGFIKVGQYDQVLVEICGHKAIGT
VLIGPTPVNIIGRNLLTQIGCTLNF
>216597_14 0.4
PQITLWQRPLVTIKIGGQLKEALLDTGADDTVLEEMDIPGRWKPKMIGGIGGFIKVGQYDQVLVEICGHKAMG
TVLVGPTPVNIIGRNLLTQIGCTLNF
>216597_15 0.4
PQITLWQRPLVTIKIGGQLKEALLDTGADDTVLEEMDIPGRWKPKMIGGIGGFIKVGQYDQVLVEICGHKAMG
TVLIGPTPVNIIGRNLLTQIGCTLNF
>216597_16 0.4
PQITLWQRPLVTIKIGGQLKEALLDTGADDTVLEEMDIPGRWKPKMIGGIGGFIKVGQYDQVLVEICGHKAVGT
VLVGPTPVNIIGRNLLTQIGCTLNF
>216597_17 0.4
PQITLWQRPLVTIKIGGQLKEALLDTGADDTVLEEMDIPGRWKPKMIGGIGGFIKVGQYDQVLVEICGHKAVGT
VLIGPTPVNIIGRNLLTQIGCTLNF
>216597_24 0.4
PQITLWQRPLVTIKIGGQLKEALLDTGADDTVLEEMDIPGKWKPKMIGGIGGFIKVGQYDQVLIEICGHKAIGTV
LVGPTPVNIIGRNLLTQIGCTLNF
>216597_25 0.4
PQITLWQRPLVTIKIGGQLKEALLDTGADDTVLEEMDIPGKWKPKMIGGIGGFIKVGQYDQVLIEICGHKAIGTV
LIGPTPVNIIGRNLLTQIGCTLNF
>216597_26 0.4
PQITLWQRPLVTIKIGGQLKEALLDTGADDTVLEEMDIPGKWKPKMIGGIGGFIKVGQYDQVLIEICGHKAMGT
VLVGPTPVNIIGRNLLTQIGCTLNF
>216597_27 0.4
PQITLWQRPLVTIKIGGQLKEALLDTGADDTVLEEMDIPGKWKPKMIGGIGGFIKVGQYDQVLIEICGHKAMGT
VLIGPTPVNIIGRNLLTQIGCTLNF
>216597_28 0.4
PQITLWQRPLVTIKIGGQLKEALLDTGADDTVLEEMDIPGKWKPKMIGGIGGFIKVGQYDQVLIEICGHKAVGT
VLVGPTPVNIIGRNLLTQIGCTLNF
>216597_29 0.4
PQITLWQRPLVTIKIGGQLKEALLDTGADDTVLEEMDIPGKWKPKMIGGIGGFIKVGQYDQVLIEICGHKAVGT
VLIGPTPVNIIGRNLLTQIGCTLNF
>216597_36 0.4

PQITLWQRPLVTIKIGGQLKEALLDTGADDTVLEEMDIPGKWKPKMIGGIGGFIKVGQYDQVLVEICGHKAIGT
VLVGPTPVNIIGRNLLTQIGCTLNF
>216597_37 0.4
PQITLWQRPLVTIKIGGQLKEALLDTGADDTVLEEMDIPGKWKPKMIGGIGGFIKVGQYDQVLVEICGHKAIGT
VLIGPTPVNIIGRNLLTQIGCTLNF
>216597_38 0.4
PQITLWQRPLVTIKIGGQLKEALLDTGADDTVLEEMDIPGKWKPKMIGGIGGFIKVGQYDQVLVEICGHKAMGT
VLVGPTPVNIIGRNLLTQIGCTLNF
>216597_39 0.4
PQITLWQRPLVTIKIGGQLKEALLDTGADDTVLEEMDIPGKWKPKMIGGIGGFIKVGQYDQVLVEICGHKAMGT
VLIGPTPVNIIGRNLLTQIGCTLNF
>216597_60 0.4
PQITLWQRPLVTIKIGGQLKEALLDTGADDTVLEEMDMPGRWKPKMIGGIGGFIKVGQYDQVLVEICGHKAIG
TVLVGPTPVNIIGRNLLTQIGCTLNF
>216597_326 0.4
PQITLWQRPLVTIKIGGQLKEALLDTGADDTVLEEMRIPGKWKPKMIGGIGGFIKVGQYDQVLVEICGHKAMGT
VLVGPTPVNIIGRNLLTQIGCTLNF
>216597_327 0.4
PQITLWQRPLVTIKIGGQLKEALLDTGADDTVLEEMRIPGKWKPKMIGGIGGFIKVGQYDQVLVEICGHKAMGT
VLIGPTPVNIIGRNLLTQIGCTLNF
>216597_328 0.4
PQITLWQRPLVTIKIGGQLKEALLDTGADDTVLEEMRIPGKWKPKMIGGIGGFIKVGQYDQVLVEICGHKAVGT
VLVGPTPVNIIGRNLLTQIGCTLNF
>216597_329 0.4
PQITLWQRPLVTIKIGGQLKEALLDTGADDTVLEEMRIPGKWKPKMIGGIGGFIKVGQYDQVLVEICGHKAVGT
VLIGPTPVNIIGRNLLTQIGCTLNF
>216597_336 0.4
PQITLWQRPLVTIKIGGQLKEALLDTGADDTVLEEMRMPGRWKPKMIGGIGGFIKVGQYDQVLIEICGHKAIGT
VLVGPTPVNIIGRNLLTQIGCTLNF
>216597_337 0.4
PQITLWQRPLVTIKIGGQLKEALLDTGADDTVLEEMRMPGRWKPKMIGGIGGFIKVGQYDQVLIEICGHKAIGT
VLIGPTPVNIIGRNLLTQIGCTLNF
>216597_338 0.4
PQITLWQRPLVTIKIGGQLKEALLDTGADDTVLEEMRMPGRWKPKMIGGIGGFIKVGQYDQVLIEICGHKAMG
TVLVGPTPVNIIGRNLLTQIGCTLNF
>216597_339 0.4
PQITLWQRPLVTIKIGGQLKEALLDTGADDTVLEEMRMPGRWKPKMIGGIGGFIKVGQYDQVLIEICGHKAMG
TVLIGPTPVNIIGRNLLTQIGCTLNF
>216597_340 0.4
PQITLWQRPLVTIKIGGQLKEALLDTGADDTVLEEMRMPGRWKPKMIGGIGGFIKVGQYDQVLIEICGHKAVG
TVLVGPTPVNIIGRNLLTQIGCTLNF
>216597_341 0.4
PQITLWQRPLVTIKIGGQLKEALLDTGADDTVLEEMRMPGRWKPKMIGGIGGFIKVGQYDQVLIEICGHKAVG
TVLIGPTPVNIIGRNLLTQIGCTLNF
>216597_348 0.4
PQITLWQRPLVTIKIGGQLKEALLDTGADDTVLEEMRMPGRWKPKMIGGIGGFIKVGQYDQVLVEICGHKAIG
TVLVGPTPVNIIGRNLLTQIGCTLNF
>216597_349 0.4
PQITLWQRPLVTIKIGGQLKEALLDTGADDTVLEEMRMPGRWKPKMIGGIGGFIKVGQYDQVLVEICGHKAIG
TVLIGPTPVNIIGRNLLTQIGCTLNF
>216597_350 0.4
PQITLWQRPLVTIKIGGQLKEALLDTGADDTVLEEMRMPGRWKPKMIGGIGGFIKVGQYDQVLVEICGHKAMG
TVLVGPTPVNIIGRNLLTQIGCTLNF
>216597_351 0.4
PQITLWQRPLVTIKIGGQLKEALLDTGADDTVLEEMRMPGRWKPKMIGGIGGFIKVGQYDQVLVEICGHKAMG
TVLIGPTPVNIIGRNLLTQIGCTLNF
>216597_352 0.4
PQITLWQRPLVTIKIGGQLKEALLDTGADDTVLEEMRMPGRWKPKMIGGIGGFIKVGQYDQVLVEICGHKAVG
TVLVGPTPVNIIGRNLLTQIGCTLNF
>216597_400 0.4
PQITLWQRPLVTIKVGGQLKEALLDTGADDTVLEEMDIPGRWKPKMIGGIGGFIKVGQYDQVLVEICGHKAVG
TVLVGPTPVNIIGRNLLTQIGCTLNF
>216597_401 0.4

PQITLWQRPLVTIKVGGQLKEALLDTGADDTVLEEMDIPGRWKPKMIGGIGGFIKVGQYDQVLVEICGHKAVG
TVLIGPTPVNIIGRNLLTQIGCTLNF
>216597_408 0.4
PQITLWQRPLVTIKVGGQLKEALLDTGADDTVLEEMDIPGKWKPKMIGGIGGFIKVGQYDQVLIEICGHKAIGT
VLVGPTPVNIIGRNLLTQIGCTLNF
>216597_409 0.4
PQITLWQRPLVTIKVGGQLKEALLDTGADDTVLEEMDIPGKWKPKMIGGIGGFIKVGQYDQVLIEICGHKAIGT
VLIGPTPVNIIGRNLLTQIGCTLNF
>259193_0 0.3
PQITLWQRPLVTIKIGGQIKEALLDTGADDTVLEEIDLPGKWKPKMIGGIGGFIKVGQYDEILVEICGHKAIGTVL
VGPTPVNIIGRNLLTQIGCTLNF
>259193_1 0.3
PQITLWQRPLVTIKIGGQIKEALLDTGADDTVLEEIDLPGKWKPKMIGGIGGFIKVGQYDEILVEIWGHKAIGTVL
VGPTPVNIIGRNLLTQIGCTLNF
>259193_2 0.3
PQITLWQRPLVTIKIGGQIKEALLDTGADDTVLEEIDLPGKWKPKMIGGIGGFIKVGQYDEILVDICGHKAIGTVL
VGPTPVNIIGRNLLTQIGCTLNF
>259193_3 0.3
PQITLWQRPLVTIKIGGQIKEALLDTGADDTVLEEIDLPGKWKPKMIGGIGGFIKVGQYDEILVDIWGHKAIGTVL
VGPTPVNIIGRNLLTQIGCTLNF
>109484_0 0.3
PQITLWQRPLVTIKIGGQLKEALLDTGADDTVLEEMNLPGRWKPKMIGGIGGFIKVRQYDQIPIEICGHKATGT
VLIGPTPVNIIGRNLLTQLGCTLNF
>116505_0 0.3
PQITLWQRPLVTVKIGGQLKEALLDTGADDTVLEEMNLPGRWKPKMIGGIGGFIKVRQYDQIPIEICGHKAEGT
VLVGPTPVNIIGRNLLTQIGCTLNF
>116505_1 0.3
PQITLWQRPLVTVKIGGQLKEALLDTGADDTVLEEMSLPGRWKPKMIGGIGGFIKVRQYDQIPIEICGHKAEGT
VLVGPTPVNIIGRNLLTQIGCTLNF
>145683_0 0.3
PQITLWQRPIVTIKIGGQLKEALLDTGADDTVLEEMNLPGRWKPKMIGGIGGFIKVRQYDQIAIEICGHKAIGTV
LVGPTPVNIIGRNLLTQLGCTLNF
>145683_1 0.3
PQITLWQRPIVTIKIGGQLKEALLDTGADDTVLEEINLPGRWKPKMIGGIGGFIKVRQYDQIAIEICGHKAIGTVL
VGPTPVNIIGRNLLTQLGCTLNF
>198527_0 0.3
PQITLWQRPLVTIKIGGQLKEALLDTGADDTVLEEMNLPGRWKPKMIGGIGGFIKVRQYDEVTIDICGHKAIGT
VLIGPTPVNIIGRNLLTQIGCTLNF
>116920_0 0.2
PQITLWQRPLVTIKIGGQLKEALLDTGADDTVLEEMNLPGRWKPRMIGGLGGFIKVRQYDQVPIEICGHKIVGT
VLIGPTPANIIGRNLLTQLGCTLNF
>257927_0 0.3
PQITLWQRPLVTIRIGGQLKEALLDTGADDTVLEEMNLPGRWKPKMIGGIGGFIKVGQYDQIPLEICGHKAVGT
VLIGPTPVNIIGRNLLTQIGCTLNF
>187173_0 0.2
PQITLWQRPLVTVKIGGQLKEALLDTGADDTVLEDINLPGKWKPKMIGGIGGFIKVRQYDQILIEICGKKAIGTVL
VGPTPVNIIGRNMLTQIGCTLNF
>187173_1 0.2
PQITLWQRPLVTVKIGGQLKEALLDTGADDTVLEDINLPGKWKPKMIGGIGGFIKVRQYDQILIEICGKKAIGTVL
IGPTPVNIIGRNMLTQIGCTLNF
>187173_2 0.2
PQITLWQRPLVTVKIGGQLKEALLDTGADDTVLEDINLPGNWKPKMIGGIGGFIKVRQYDQILIEICGKKAIGTV
LVGPTPVNIIGRNMLTQIGCTLNF
>187173_3 0.2
PQITLWQRPLVTVKIGGQLKEALLDTGADDTVLEDINLPGNWKPKMIGGIGGFIKVRQYDQILIEICGKKAIGTV
LIGPTPVNIIGRNMLTQIGCTLNF
>187173_4 0.2
PQITLWQRPLVTVKIGGQLTEALLDTGADDTVLEDINLPGKWKPKMIGGIGGFIKVRQYDQILIEICGKKAIGTVL
VGPTPVNIIGRNMLTQIGCTLNF
>187173_5 0.2
PQITLWQRPLVTVKIGGQLTEALLDTGADDTVLEDINLPGKWKPKMIGGIGGFIKVRQYDQILIEICGKKAIGTVL
IGPTPVNIIGRNMLTQIGCTLNF
>187173_6 0.2

PQITLWQRPLVTVKIGGQLTEALLDTGADDTVLEDINLPGNWKPKMIGGIGGFIKVRQYDQILIEICGKKAIGTVL
VGPTPVNIIGRNMLTQIGCTLNF
>187173_7 0.2
PQITLWQRPLVTVKIGGQLTEALLDTGADDTVLEDINLPGNWKPKMIGGIGGFIKVRQYDQILIEICGKKAIGTVL
IGPTPVNIIGRNMLTQIGCTLNF
>187173_8 0.2
PQITLWQRPLVTVRIGGQLKEALLDTGADDTVLEDINLPGKWKPKMIGGIGGFIKVRQYDQILIEICGKKAIGTV
LVGPTPVNIIGRNMLTQIGCTLNF
>187173_9 0.2
PQITLWQRPLVTVRIGGQLKEALLDTGADDTVLEDINLPGKWKPKMIGGIGGFIKVRQYDQILIEICGKKAIGTV
LIGPTPVNIIGRNMLTQIGCTLNF
>187173_10 0.2
PQITLWQRPLVTVRIGGQLKEALLDTGADDTVLEDINLPGNWKPKMIGGIGGFIKVRQYDQILIEICGKKAIGTV
LVGPTPVNIIGRNMLTQIGCTLNF
>187173_11 0.2
PQITLWQRPLVTVRIGGQLKEALLDTGADDTVLEDINLPGNWKPKMIGGIGGFIKVRQYDQILIEICGKKAIGTV
LIGPTPVNIIGRNMLTQIGCTLNF
>187173_12 0.2
PQITLWQRPLVTVRIGGQLTEALLDTGADDTVLEDINLPGKWKPKMIGGIGGFIKVRQYDQILIEICGKKAIGTVL
VGPTPVNIIGRNMLTQIGCTLNF
>187173_13 0.2
PQITLWQRPLVTVRIGGQLTEALLDTGADDTVLEDINLPGKWKPKMIGGIGGFIKVRQYDQILIEICGKKAIGTVL
IGPTPVNIIGRNMLTQIGCTLNF
>187173_14 0.2
PQITLWQRPLVTVRIGGQLTEALLDTGADDTVLEDINLPGNWKPKMIGGIGGFIKVRQYDQILIEICGKKAIGTVL
VGPTPVNIIGRNMLTQIGCTLNF
>187173_15 0.2
PQITLWQRPLVTVRIGGQLTEALLDTGADDTVLEDINLPGNWKPKMIGGIGGFIKVRQYDQILIEICGKKAIGTVL
IGPTPVNIIGRNMLTQIGCTLNF
>187173_16 0.2
PQITLWQRPIVTVKIGGQLKEALLDTGADDTVLEDINLPGKWKPKMIGGIGGFIKVRQYDQILIEICGKKAIGTVL
VGPTPVNIIGRNMLTQIGCTLNF
>187173_17 0.2
PQITLWQRPIVTVKIGGQLKEALLDTGADDTVLEDINLPGKWKPKMIGGIGGFIKVRQYDQILIEICGKKAIGTVL
IGPTPVNIIGRNMLTQIGCTLNF
>187173_18 0.2
PQITLWQRPIVTVKIGGQLKEALLDTGADDTVLEDINLPGNWKPKMIGGIGGFIKVRQYDQILIEICGKKAIGTVL
VGPTPVNIIGRNMLTQIGCTLNF
>187173_19 0.2
PQITLWQRPIVTVKIGGQLKEALLDTGADDTVLEDINLPGNWKPKMIGGIGGFIKVRQYDQILIEICGKKAIGTVL
IGPTPVNIIGRNMLTQIGCTLNF
>187173_20 0.2
PQITLWQRPIVTVKIGGQLTEALLDTGADDTVLEDINLPGKWKPKMIGGIGGFIKVRQYDQILIEICGKKAIGTVL
VGPTPVNIIGRNMLTQIGCTLNF
>187173_21 0.2
PQITLWQRPIVTVKIGGQLTEALLDTGADDTVLEDINLPGKWKPKMIGGIGGFIKVRQYDQILIEICGKKAIGTVLI
GPTPVNIIGRNMLTQIGCTLNF
>187173_22 0.2
PQITLWQRPIVTVKIGGQLTEALLDTGADDTVLEDINLPGNWKPKMIGGIGGFIKVRQYDQILIEICGKKAIGTVL
VGPTPVNIIGRNMLTQIGCTLNF
>187173_23 0.2
PQITLWQRPIVTVKIGGQLTEALLDTGADDTVLEDINLPGNWKPKMIGGIGGFIKVRQYDQILIEICGKKAIGTVLI
GPTPVNIIGRNMLTQIGCTLNF
>187173_24 0.2
PQITLWQRPIVTVRIGGQLKEALLDTGADDTVLEDINLPGKWKPKMIGGIGGFIKVRQYDQILIEICGKKAIGTVL
VGPTPVNIIGRNMLTQIGCTLNF
>187173_25 0.2
PQITLWQRPIVTVRIGGQLKEALLDTGADDTVLEDINLPGKWKPKMIGGIGGFIKVRQYDQILIEICGKKAIGTVL
IGPTPVNIIGRNMLTQIGCTLNF
>187173_26 0.2
PQITLWQRPIVTVRIGGQLKEALLDTGADDTVLEDINLPGNWKPKMIGGIGGFIKVRQYDQILIEICGKKAIGTVL
VGPTPVNIIGRNMLTQIGCTLNF
>187173_27 0.2

PQITLWQRPIVTVRIGGQLKEALLDTGADDTVLEDINLPGNWKPKMIGGIGGFIKVRQYDQILIEICGKKAIGTVL
IGPTPVNIIGRNMLTQIGCTLNF
>187173_28 0.2
PQITLWQRPIVTVRIGGQLTEALLDTGADDTVLEDINLPGKWKPKMIGGIGGFIKVRQYDQILIEICGKKAIGTVL
VGPTPVNIIGRNMLTQIGCTLNF
>187173_29 0.2
PQITLWQRPIVTVRIGGQLTEALLDTGADDTVLEDINLPGKWKPKMIGGIGGFIKVRQYDQILIEICGKKAIGTVLI
GPTPVNIIGRNMLTQIGCTLNF
>187173_30 0.2
PQITLWQRPIVTVRIGGQLTEALLDTGADDTVLEDINLPGNWKPKMIGGIGGFIKVRQYDQILIEICGKKAIGTVL
VGPTPVNIIGRNMLTQIGCTLNF
>187173_31 0.2
PQITLWQRPIVTVRIGGQLTEALLDTGADDTVLEDINLPGNWKPKMIGGIGGFIKVRQYDQILIEICGKKAIGTVL
IGPTPVNIIGRNMLTQIGCTLNF

Sequences for the FPV-resistant ensemble
>235731_150 200.0
PQITLWQRPFVTVTVGGQTTEALLDTGADDTILEDIPLPGRWIPKIIGGIGGLIKVGQYDQIPIEIFGHTVIGTVLV
GATPVNVIGRNLMTRIGCTLNF
>235731_152 200.0
PQITLWQRPFVTVTVGGQTTEALLDTGADDTILEDIPLPGRWIPKIIGGIGGLIKVGQYDQIPIEIFGQTVIGTVLV
GATPVNVIGRNLMTRIGCTLNF
>235731_153 200.0
PQITLWQRPFVTVTVGGQTTEALLDTGADDTILEDIPLPGRWIPKIIGGIGGLIRVGQYDQIPIEIFGHTVIGTVLV
GATPVNVIGRNLMTRIGCTLNF
>235685_1 200.0
PQITLWQRPFVTVKVGGQTTEALLDTGADDTIFEDINLPGRWIPKIIGGIGGLIKVGQYDQIPIEIFGLTVIGTVLV
GATPVNVIGRNLMTRIGCTLNF
>235685_4 200.0
PQITLWQRPFVTVKVGGQTTEALLDTGADDTIFEDINLPGRWIPKIIGGIGGLIRVGQYDQIPIEIFGLTVIGTVLV
GATPVNVIGRNLMTRIGCTLNF
>235685_13 200.0
PQITLWQRPFVTVKVGGQTTEALLDTGADDTIFEDIHLPGRWIPKIIGGIGGLIKVGQYDQIPIEIFGLTVIGTVLV
GATPVNVIGRNLMTRIGCTLNF
>235685_16 200.0
PQITLWQRPFVTVKVGGQTTEALLDTGADDTIFEDIHLPGRWIPKIIGGIGGLIRVGQYDQIPIEIFGLTVIGTVLV
GATPVNVIGRNLMTRIGCTLNF
>235685_25 200.0
PQITLWQRPFVTVTVGGQTTEALLDTGADDTIFEDINLPGRWIPKIIGGIGGLIKVGQYDQIPIEIFGLTVIGTVLV
GATPVNVIGRNLMTRIGCTLNF
>235685_28 200.0
PQITLWQRPFVTVTVGGQTTEALLDTGADDTIFEDINLPGRWIPKIIGGIGGLIRVGQYDQIPIEIFGLTVIGTVLV
GATPVNVIGRNLMTRIGCTLNF
>235685_37 200.0
PQITLWQRPFVTVTVGGQTTEALLDTGADDTIFEDIHLPGRWIPKIIGGIGGLIKVGQYDQIPIEIFGLTVIGTVLV
GATPVNVIGRNLMTRIGCTLNF
>235685_40 200.0
PQITLWQRPFVTVTVGGQTTEALLDTGADDTIFEDIHLPGRWIPKIIGGIGGLIRVGQYDQIPIEIFGLTVIGTVLV
GATPVNVIGRNLMTRIGCTLNF
>235731_1 200.0
PQITLWQRPFVTVKVGGQTTEALLDTGADDTILEDINLPGRWIPKIIGGIGGFIKVGQYDQIPIEIFGLTVIGTVLV
GATPVNVIGRNLMTRIGCTLNF
>235731_4 200.0
PQITLWQRPFVTVKVGGQTTEALLDTGADDTILEDINLPGRWIPKIIGGIGGFIRVGQYDQIPIEIFGLTVIGTVLV
GATPVNVIGRNLMTRIGCTLNF
>235731_7 200.0
PQITLWQRPFVTVKVGGQTTEALLDTGADDTILEDINLPGRWIPKIIGGIGGLIKVGQYDQIPIEIFGLTVIGTVLV
GATPVNVIGRNLMTRIGCTLNF
>235731_10 200.0
PQITLWQRPFVTVKVGGQTTEALLDTGADDTILEDINLPGRWIPKIIGGIGGLIRVGQYDQIPIEIFGLTVIGTVLV
GATPVNVIGRNLMTRIGCTLNF
>235731_25 200.0
PQITLWQRPFVTVKVGGQTTEALLDTGADDTILEDIHLPGRWIPKIIGGIGGFIKVGQYDQIPIEIFGLTVIGTVLV

GATPVNVIGRNLMTRIGCTLNF
>117039_1 400.0
PQITLWQRPLITVKIGGQLVEALMDTGADDTIFEGINLPGRWKPKMIGGIGGFLNVRQYEQIPIEIGGHKILSTVL
VGSTPVNVIGRNVMSKIGCTLNF
>117039_3 400.0
PQITLWQRPVITVKIGGQLVEALMDTGADDTIFEGINLPGRWKPKMIGGIGGFLNVRQYEQIPIEIGGHKILSTVL
VGSTPVNVIGRNVMSKIGCTLNF
>235731_177 200.0
PQITLWQRPFVTVTVGGQTTEALLDTGADDTILEDITLPGRWIPKIIGGIGGLIRVGQYDQIPIEIFGHTVIGTVLV
GATPVNVIGRNLMTRIGCTLNF
>235731_179 200.0
PQITLWQRPFVTVTVGGQTTEALLDTGADDTILEDITLPGRWIPKIIGGIGGLIRVGQYDQIPIEIFGQTVIGTVLV
GATPVNVIGRNLMTRIGCTLNF
>205693_0 200.0
PQITLWQRPFIPVKIGGQQMEALLDTGADDTILEEINLPGRWTPKIVGGIGGFMRVGQYDQIPIEICGQKIIGTVL
VGPTPANIIGRNVMTQIGCTLNF
>205693_1 200.0
PQITLWQRPFIPVKIGGQQMEALLDTGADDTILEEINLPGRWTPKIVGGIGGFMRVGQYDQIPIEIYGQKIIGTVL
VGPTPANIIGRNVMTQIGCTLNF
>235685_7 200.0
PQITLWQRPFVTVKVGGQTTEALLDTGADDTIFEDINLPGRWTPKIIGGIGGLIKVGQYDQIPIEIFGLTVIGTVLV
GATPVNVIGRNLMTRIGCTLNF
>235685_10 200.0
PQITLWQRPFVTVKVGGQTTEALLDTGADDTIFEDINLPGRWTPKIIGGIGGLIRVGQYDQIPIEIFGLTVIGTVLV
GATPVNVIGRNLMTRIGCTLNF
>235685_19 200.0
PQITLWQRPFVTVKVGGQTTEALLDTGADDTIFEDIHLPGRWTPKIIGGIGGLIKVGQYDQIPIEIFGLTVIGTVLV
GATPVNVIGRNLMTRIGCTLNF
>235685_22 200.0
PQITLWQRPFVTVKVGGQTTEALLDTGADDTIFEDIHLPGRWTPKIIGGIGGLIRVGQYDQIPIEIFGLTVIGTVLV
GATPVNVIGRNLMTRIGCTLNF
>235685_31 200.0
PQITLWQRPFVTVTVGGQTTEALLDTGADDTIFEDINLPGRWTPKIIGGIGGLIKVGQYDQIPIEIFGLTVIGTVLV
GATPVNVIGRNLMTRIGCTLNF
>235685_34 200.0
PQITLWQRPFVTVTVGGQTTEALLDTGADDTIFEDINLPGRWTPKIIGGIGGLIRVGQYDQIPIEIFGLTVIGTVLV
GATPVNVIGRNLMTRIGCTLNF
>235685_43 200.0
PQITLWQRPFVTVTVGGQTTEALLDTGADDTIFEDIHLPGRWTPKIIGGIGGLIKVGQYDQIPIEIFGLTVIGTVLV
GATPVNVIGRNLMTRIGCTLNF
>235685_46 200.0
PQITLWQRPFVTVTVGGQTTEALLDTGADDTIFEDIHLPGRWTPKIIGGIGGLIRVGQYDQIPIEIFGLTVIGTVLV
GATPVNVIGRNLMTRIGCTLNF
>235731_13 200.0
PQITLWQRPFVTVKVGGQTTEALLDTGADDTILEDINLPGRWTPKIIGGIGGFIKVGQYDQIPIEIFGLTVIGTVLV
GATPVNVIGRNLMTRIGCTLNF
>235731_16 200.0
PQITLWQRPFVTVKVGGQTTEALLDTGADDTILEDINLPGRWTPKIIGGIGGFIRVGQYDQIPIEIFGLTVIGTVLV
GATPVNVIGRNLMTRIGCTLNF
>235731_19 200.0
PQITLWQRPFVTVKVGGQTTEALLDTGADDTILEDINLPGRWTPKIIGGIGGLIKVGQYDQIPIEIFGLTVIGTVLV
GATPVNVIGRNLMTRIGCTLNF
>235731_85 200.0
PQITLWQRPFVTVKVGGQTTEALLDTGADDTILEDITLPGRWTPKIIGGIGGFIKVGQYDQIPIEIFGLTVIGTVLV
GATPVNVIGRNLMTRIGCTLNF
>117039_0 400.0
PQITLWQRPLITVKIGGQLVEALMDTGADDTIFEGINLPGRWKPKMIGGIGGFLNVRQYEQIPIEICGHKILSTVL
VGSTPVNVIGRNVMSKIGCTLNF
>117039_2 400.0
PQITLWQRPVITVKIGGQLVEALMDTGADDTIFEGINLPGRWKPKMIGGIGGFLNVRQYEQIPIEICGHKILSTVL
VGSTPVNVIGRNVMSKIGCTLNF
>117075_0 400.0
PQITLWQRPLITVKIGGQLVEALMDTGADDTIFEGINLPGRWKPKMIGGIGGFLNVRQYEQIPIEICGHKILSTVL

VGSTPLNVIGRNVMSKIGCTLNF
>117075_1 400.0
PQITLWQRPLITVKIGGQLVEALMDTGADDTIFEGINLPGRWKPKMIGGIGGFLNVREYEQIPIEICGHKILSTVL
VGSTPLNVIGRNVMSKIGCTLNF
>117075_2 400.0
PQITLWQRPLITVKVGGQLVEALMDTGADDTIFEGINLPGRWKPKMIGGIGGFLNVRQYEQIPIEICGHKILSTV
LVGSTPLNVIGRNVMSKIGCTLNF
>117075_3 400.0
PQITLWQRPLITVKVGGQLVEALMDTGADDTIFEGINLPGRWKPKMIGGIGGFLNVREYEQIPIEICGHKILSTV
LVGSTPLNVIGRNVMSKIGCTLNF
>117075_4 400.0
PQITLWQRPFITVKIGGQLVEALMDTGADDTIFEGINLPGRWKPKMIGGIGGFLNVRQYEQIPIEICGHKILSTVL
VGSTPLNVIGRNVMSKIGCTLNF
>117075_5 400.0
PQITLWQRPFITVKIGGQLVEALMDTGADDTIFEGINLPGRWKPKMIGGIGGFLNVREYEQIPIEICGHKILSTVL
VGSTPLNVIGRNVMSKIGCTLNF
>117075_6 400.0
PQITLWQRPFITVKVGGQLVEALMDTGADDTIFEGINLPGRWKPKMIGGIGGFLNVRQYEQIPIEICGHKILSTV
LVGSTPLNVIGRNVMSKIGCTLNF
>117075_7 400.0
PQITLWQRPFITVKVGGQLVEALMDTGADDTIFEGINLPGRWKPKMIGGIGGFLNVREYEQIPIEICGHKILSTV
LVGSTPLNVIGRNVMSKIGCTLNF
>117075_8 400.0
PQITLWQRPVITVKIGGQLVEALMDTGADDTIFEGINLPGRWKPKMIGGIGGFLNVRQYEQIPIEICGHKILSTVL
VGSTPLNVIGRNVMSKIGCTLNF
>117075_9 400.0
PQITLWQRPVITVKIGGQLVEALMDTGADDTIFEGINLPGRWKPKMIGGIGGFLNVREYEQIPIEICGHKILSTVL
VGSTPLNVIGRNVMSKIGCTLNF
>117075_10 400.0
PQITLWQRPVITVKVGGQLVEALMDTGADDTIFEGINLPGRWKPKMIGGIGGFLNVRQYEQIPIEICGHKILSTV
LVGSTPLNVIGRNVMSKIGCTLNF
>117075_11 400.0
PQITLWQRPVITVKVGGQLVEALMDTGADDTIFEGINLPGRWKPKMIGGIGGFLNVREYEQIPIEICGHKILSTV
LVGSTPLNVIGRNVMSKIGCTLNF
>54177_0 252.2
PQITLWQRPVVTIKIGGQLIEALLDTGADDTVLEEINLPGRWKPKIIGGIGGFIKVRQYDQILIEICGHKVISTVVV
GPTPVNAIGRNLLTQIGCTLNF
>235731_119 200.0
PQITLWQRPFVTVTVGGQTTEALLDTGADDTILEDINLPGRWTPKIIGGIGGLIRVGQYDQIPIEIFGQTVIGTVL
VGATPVNVIGRNLMTRIGCTLNF
>117133_0 400.0
PQITLWQRPVVVVKVGGQLMEALLDTGADDTIFEEMNLPGRWTPKIVGGIGGFMKVRQYENVPIEIYGKKILS
TVLIGPTPANIIGRNVMTQIGCTLNF
>117063_1 400.0
PQITLWQRPVVTVKVAGQLREALLDTGADDTIFEEINLPGRWTPKIVGGIGGFMKVRQYDQIPIEICGHKVISTV
LVGPTPTNIIGGNLMTQIGCTLNF
>98601_0 400.0
PQITLWQRPFVTIKIGGQLIEALLDTGADDTVLEEMNLPGRWKPKIIGGIGGFMKVRQYDQIPIEICGHKVITTVL
IGPTPVNAIGRNLLTQIGCTLNF
>103779_0 400.0
PQITLWQRPIIKVKIGGQLVEALLDTGADDTILEGINLPGRWKPKIVGGIGGFMKVKEYDQIPVEICGHKIISTVLV
GPTPVNIIGRNVMTQIGCTLNF
>103779_1 400.0
PQITLWQRPIIKVKIGGQLVEALLDTGADDTILEGINLPGRWKPKIVGGIGGFMKVKEYDQIPVEICGHKIISTVLV
GPTPVNVIGRNVMTQIGCTLNF
>103779_2 400.0
PQITLWQRPIIKVKIGGQLVEALLDTGADDTILEGINLPGRWKPKIVGGIGGFMKVKEYDQIPVEICGHKVISTVL
VGPTPVNIIGRNVMTQIGCTLNF
>103779_3 400.0
PQITLWQRPIIKVKIGGQLVEALLDTGADDTILEGINLPGRWKPKIVGGIGGFMKVKEYDQIPVEICGHKVISTVL
VGPTPVNVIGRNVMTQIGCTLNF
>103779_4 400.0
PQITLWQRPIIKVKIGGQLVEALLDTGADDTILEGIDLPGRWKPKIVGGIGGFMKVKEYDQIPVEICGHKIISTVLV

GPTPVNIIGRNVMTQIGCTLNF
>103779_5 400.0
PQITLWQRPIIKVKIGGQLVEALLDTGADDTILEGIDLPGRWKPKIVGGIGGFMKVKEYDQIPVEICGHKIISTVLV
GPTPVNVIGRNVMTQIGCTLNF
>103779_6 400.0
PQITLWQRPIIKVKIGGQLVEALLDTGADDTILEGIDLPGRWKPKIVGGIGGFMKVKEYDQIPVEICGHKVISTVL
VGPTPVNIIGRNVMTQIGCTLNF
>103779_7 400.0
PQITLWQRPIIKVKIGGQLVEALLDTGADDTILEGIDLPGRWKPKIVGGIGGFMKVKEYDQIPVEICGHKVISTVL
VGPTPVNVIGRNVMTQIGCTLNF
>103779_8 400.0
PQITLWQRPIIKVKIGGQLVEALLDTGADDTIFEGINLPGRWKPKIVGGIGGFMKVKEYDQIPVEICGHKIISTVL
VGPTPVNIIGRNVMTQIGCTLNF
>103779_9 400.0
PQITLWQRPIIKVKIGGQLVEALLDTGADDTIFEGINLPGRWKPKIVGGIGGFMKVKEYDQIPVEICGHKIISTVL
VGPTPVNVIGRNVMTQIGCTLNF
>103779_10 400.0
PQITLWQRPIIKVKIGGQLVEALLDTGADDTIFEGINLPGRWKPKIVGGIGGFMKVKEYDQIPVEICGHKVISTVL
VGPTPVNIIGRNVMTQIGCTLNF
>103779_11 400.0
PQITLWQRPIIKVKIGGQLVEALLDTGADDTIFEGINLPGRWKPKIVGGIGGFMKVKEYDQIPVEICGHKVISTVL
VGPTPVNVIGRNVMTQIGCTLNF
>103779_12 400.0
PQITLWQRPIIKVKIGGQLVEALLDTGADDTIFEGIDLPGRWKPKIVGGIGGFMKVKEYDQIPVEICGHKIISTVL
VGPTPVNIIGRNVMTQIGCTLNF
>103779_13 400.0
PQITLWQRPIIKVKIGGQLVEALLDTGADDTIFEGIDLPGRWKPKIVGGIGGFMKVKEYDQIPVEICGHKIISTVL
VGPTPVNVIGRNVMTQIGCTLNF
>103779_14 400.0
PQITLWQRPIIKVKIGGQLVEALLDTGADDTIFEGIDLPGRWKPKIVGGIGGFMKVKEYDQIPVEICGHKVISTVL
VGPTPVNIIGRNVMTQIGCTLNF
>103779_15 400.0
PQITLWQRPIIKVKIGGQLVEALLDTGADDTIFEGIDLPGRWKPKIVGGIGGFMKVKEYDQIPVEICGHKVISTVL
VGPTPVNVIGRNVMTQIGCTLNF
>147550_0 400.0
PQITLWQRPIIKVKIGGQLVEALLDTGADDTIFEGIDLPGRWKPKIVGGIGGFMKVKEYDQIPVEVCGHKVISTV
LVGPTPVNVIGRNVMTQIGCTLNF
>113050_0 400.0
PQITLWQRPFVTVKIGGQITEALLDTGADDTILENINLPGRWKPKIIQGIGGFLKVRQYDQIPIEIEGHKVIGSVV
VGPTPVNVIGRNVMSQLGCTLNF
>86742_0 400.0
PQITLWQRPFITIKIGGQLREALLDTGADDTIFEDIQLPGRWKPKIVGGIGGFLKVKEYEQVPIEICGHKVISTVL
TGPTPVNVIGRNVMTQLGCTLNF
>86742_1 400.0
PQITLWQRPFITVKIGGQLREALLDTGADDTIFEDIQLPGRWKPKIVGGIGGFLKVKEYEQVPIEICGHKVISTVL
TGPTPVNVIGRNVMTQLGCTLNF
>230072_0 400.0
PQITLWQRPVITVRVGGQLTEALLDTGADDTIFEEINLPGKWKPKLIGGIGGFLKVKQYEQIPIEICEHTIMSTVL
VGPTPVNVIGRNVMTQLGCTLNF
>66194_0 300.0
PQITLWQRPVVDVKVGGQLMEALLDTGADDTIFEEMNLPGRWTPKMVGGIGGFMKVRQYEDVPIEIYGKKIL
STVLIGPTPANIIGRNVMTQIGCTLNF
>66194_1 300.0
PQITLWQRPVVDVKVGGQLMEALLDTGADDTIFEEMNLPGRWTPKMVGGIGGFMKVRQYENVPIEIYGKKIL
STVLIGPTPANIIGRNVMTQIGCTLNF
>66194_2 300.0
PQITLWQRPVVDVKVGGQLMEALLDTGADDTIFEEMNLPGRWTPKIVGGIGGFMKVRQYEDVPIEIYGKKILS
TVLIGPTPANIIGRNVMTQIGCTLNF
>66194_3 300.0
PQITLWQRPVVDVKVGGQLMEALLDTGADDTIFEEMNLPGRWTPKIVGGIGGFMKVRQYENVPIEIYGKKILS
TVLIGPTPANIIGRNVMTQIGCTLNF
>66194_4 300.0
PQITLWQRPVVDVKVGGQLMEALLDTGADDTIFEELNLPGRWTPKMVGGIGGFMKVRQYEDVPIEIYGKKILS

TVLIGPTPANIIGRNVMTQIGCTLNF
>66194_5 300.0
PQITLWQRPVVDVKVGGQLMEALLDTGADDTIFEELNLPGRWTPKMVGGIGGFMKVRQYENVPIEIYGKKILS
TVLIGPTPANIIGRNVMTQIGCTLNF
>66194_6 300.0
PQITLWQRPVVDVKVGGQLMEALLDTGADDTIFEELNLPGRWTPKIVGGIGGFMKVRQYEDVPIEIYGKKILST
VLIGPTPANIIGRNVMTQIGCTLNF
>66194_7 300.0
PQITLWQRPVVDVKVGGQLMEALLDTGADDTIFEELNLPGRWTPKIVGGIGGFMKVRQYENVPIEIYGKKILST
VLIGPTPANIIGRNVMTQIGCTLNF
>66194_8 300.0
PQITLWQRPVVDVKVGGQLMEALLDTGADDTIFEDMNLPGRWTPKMVGGIGGFMKVRQYEDVPIEIYGKKIL
STVLIGPTPANIIGRNVMTQIGCTLNF
>66194_9 300.0
PQITLWQRPVVDVKVGGQLMEALLDTGADDTIFEDMNLPGRWTPKMVGGIGGFMKVRQYENVPIEIYGKKIL
STVLIGPTPANIIGRNVMTQIGCTLNF
>66194_10 300.0
PQITLWQRPVVDVKVGGQLMEALLDTGADDTIFEDMNLPGRWTPKIVGGIGGFMKVRQYEDVPIEIYGKKILS
TVLIGPTPANIIGRNVMTQIGCTLNF
>66194_11 300.0
PQITLWQRPVVDVKVGGQLMEALLDTGADDTIFEDMNLPGRWTPKIVGGIGGFMKVRQYENVPIEIYGKKILS
TVLIGPTPANIIGRNVMTQIGCTLNF
>66194_12 300.0
PQITLWQRPVVDVKVGGQLMEALLDTGADDTIFEDLNLPGRWTPKMVGGIGGFMKVRQYEDVPIEIYGKKILS
TVLIGPTPANIIGRNVMTQIGCTLNF
>66194_13 300.0
PQITLWQRPVVDVKVGGQLMEALLDTGADDTIFEDLNLPGRWTPKMVGGIGGFMKVRQYENVPIEIYGKKILS
TVLIGPTPANIIGRNVMTQIGCTLNF
>66194_14 300.0
PQITLWQRPVVDVKVGGQLMEALLDTGADDTIFEDLNLPGRWTPKIVGGIGGFMKVRQYEDVPIEIYGKKILS
TVLIGPTPANIIGRNVMTQIGCTLNF
>66194_15 300.0
PQITLWQRPVVDVKVGGQLMEALLDTGADDTIFEDLNLPGRWTPKIVGGIGGFMKVRQYENVPIEIYGKKILS
TVLIGPTPANIIGRNVMTQIGCTLNF
>81805_0 300.0
PQITLWQRPIITVKVGGQLREALLDTGADDTIFEDIDLPGRWTPKIIGGIGGLMKVKQYEQIPIEICGHELIGPVL
VGPTPANVIGRNVMTQIGCTLNF
>145212_1 400.0
PQITLWQRPIVTVKIAGQLKEALLDTGADDTIFEEMNLPGRWTPKLIGGIGGFLKVRQYDQVPIEICGHKVIGPV
LVGPTPANVIGRNLMSQIGCTLNF
>145212_3 400.0
PQITLWQRPIVTVRIAGQLKEALLDTGADDTIFEEMNLPGRWTPKLIGGIGGFLKVRQYDQVPIEICGHKVIGPV
LVGPTPANVIGRNLMSQIGCTLNF
>145212_5 400.0
PQITLWQRPIITVKIAGQLKEALLDTGADDTIFEEMNLPGRWTPKLIGGIGGFLKVRQYDQVPIEICGHKVIGPV
LVGPTPANVIGRNLMSQIGCTLNF
>145212_7 400.0
PQITLWQRPIITVRIAGQLKEALLDTGADDTIFEEMNLPGRWTPKLIGGIGGFLKVRQYDQVPIEICGHKVIGPV
LVGPTPANVIGRNLMSQIGCTLNF
>205630_0 400.0
PQITLWQRPIVTVKVGGQLTEALLDTGADDTIFEEMNLPGRWKPKMVGGIGGFIKVGQYDQIPIEFCGHKVIST
VLIGPTPVNIIGRNVMTQIGCTLNF
>205630_1 400.0
PQITLWQRPIVTVKVGGQLTEALLDTGADDTIFEKMNLPGRWKPKMVGGIGGFIKVGQYDQIPIEFCGHKVIST
VLIGPTPVNIIGRNVMTQIGCTLNF
>113060_0 400.0
PQITLWQRPFVTVKIGGQLREALLDTGADNTIFEEIDLPGRWKPKIIGGIGGFLKVRQYDQIPLEVCGHKTTGT
VLVGPTPVNVIGRDMMTQLGFTLNF
>117063_0 400.0
PQITLWQRPVVTVKVAGQLREALLDTGADDTIFEEINLPGRWTPKIVGGIGGFMKVRQYDQIPIEICGHKVISTV
LVGPTPTNIIGRNLMTQIGCTLNF
>98603_0 400.0
PQITLWQRPIVTIKIGGQLKEALLDTGADDTVFEEMNLPGRWKPKIIGGIGGFVKVRQYDQIPIEICGHKVIGTV

VVGPTPVNAIGRNLLTQIGCTLNF

Sequences for the FPV-susceptible ensemble
>102782_168 0.1
PQITLWQRPIVTIKVEGQLTEAPLDTGADDTVLEDVNLPGRWKPKMIGGLGGFIKVRQYDQIHIEICGHKAIGT
VLVGPTPVNIIGRNMLTQIGCTLNF
>102782_169 0.1
PQITLWQRPIVTIKVEGQLTEAPLDTGADDTVLEDVNLPGRWKPKMIGGLGGFIKVRQYDQIHIEICGHKAIGT
VLVGPTPVNIIGRSMLTQIGCTLNF
>102782_170 0.1
PQITLWQRPIVTIKVEGQLTEAPLDTGADDTVLEDVNLPGRWKPKMIGGLGGFIKVRQYDQIHIEICGHKAIGT
VLIGPTPVNIIGRNMLTQIGCTLNF
>102782_171 0.1
PQITLWQRPIVTIKVEGQLTEAPLDTGADDTVLEDVNLPGRWKPKMIGGLGGFIKVRQYDQIHIEICGHKAIGT
VLIGPTPVNIIGRSMLTQIGCTLNF
>102782_176 0.1
PQITLWQRPIVTIKVEGQLTEAPLDTGADDTVFEDMNLPGRWKPKMIGGLGGFIKVRQYDQIHIEICGHKAIGT
VLVGPTPVNIIGRNMLTQIGCTLNF
>102782_177 0.1
PQITLWQRPIVTIKVEGQLTEAPLDTGADDTVFEDMNLPGRWKPKMIGGLGGFIKVRQYDQIHIEICGHKAIGT
VLVGPTPVNIIGRSMLTQIGCTLNF
>102782_178 0.1
PQITLWQRPIVTIKVEGQLTEAPLDTGADDTVFEDMNLPGRWKPKMIGGLGGFIKVRQYDQIHIEICGHKAIGT
VLIGPTPVNIIGRNMLTQIGCTLNF
>102782_179 0.1
PQITLWQRPIVTIKVEGQLTEAPLDTGADDTVFEDMNLPGRWKPKMIGGLGGFIKVRQYDQIHIEICGHKAIGT
VLIGPTPVNIIGRSMLTQIGCTLNF
>102782_184 0.1
PQITLWQRPIVTIKVEGQLTEAPLDTGADDTVFEDVNLPGRWKPKMIGGLGGFIKVRQYDQIHIEICGHKAIGT
VLVGPTPVNIIGRNMLTQIGCTLNF
>102782_185 0.1
PQITLWQRPIVTIKVEGQLTEAPLDTGADDTVFEDVNLPGRWKPKMIGGLGGFIKVRQYDQIHIEICGHKAIGT
VLVGPTPVNIIGRSMLTQIGCTLNF
>102782_186 0.1
PQITLWQRPIVTIKVEGQLTEAPLDTGADDTVFEDVNLPGRWKPKMIGGLGGFIKVRQYDQIHIEICGHKAIGT
VLIGPTPVNIIGRNMLTQIGCTLNF
>102782_187 0.1
PQITLWQRPIVTIKVEGQLTEAPLDTGADDTVFEDVNLPGRWKPKMIGGLGGFIKVRQYDQIHIEICGHKAIGT
VLIGPTPVNIIGRSMLTQIGCTLNF
>102782_36 0.1
PQITLWQRPIVTIKVEGQLKEAPLDTGADDTVLEDMNLPGRWKPKMIGGLGGFIKVRQYDQIHIEICGHKVIGT
VLVGPTPVNIIGRNMLTQIGCTLNF
>102782_37 0.1
PQITLWQRPIVTIKVEGQLKEAPLDTGADDTVLEDMNLPGRWKPKMIGGLGGFIKVRQYDQIHIEICGHKVIGT
VLVGPTPVNIIGRSMLTQIGCTLNF
>102782_38 0.1
PQITLWQRPIVTIKVEGQLKEAPLDTGADDTVLEDMNLPGRWKPKMIGGLGGFIKVRQYDQIHIEICGHKVIGT
VLIGPTPVNIIGRNMLTQIGCTLNF
>102782_39 0.1
PQITLWQRPIVTIKVEGQLKEAPLDTGADDTVLEDMNLPGRWKPKMIGGLGGFIKVRQYDQIHIEICGHKVIGT
VLIGPTPVNIIGRSMLTQIGCTLNF
>102782_44 0.1
PQITLWQRPIVTIKVEGQLKEAPLDTGADDTVLEDVNLPGRWKPKMIGGLGGFIKVRQYDQIHIEICGHKVIGT
VLVGPTPVNIIGRNMLTQIGCTLNF
>102782_45 0.1
PQITLWQRPIVTIKVEGQLKEAPLDTGADDTVLEDVNLPGRWKPKMIGGLGGFIKVRQYDQIHIEICGHKVIGT
VLVGPTPVNIIGRSMLTQIGCTLNF
>102782_46 0.1
PQITLWQRPIVTIKVEGQLKEAPLDTGADDTVLEDVNLPGRWKPKMIGGLGGFIKVRQYDQIHIEICGHKVIGT
VLIGPTPVNIIGRNMLTQIGCTLNF
>102782_47 0.1
PQITLWQRPIVTIKVEGQLKEAPLDTGADDTVLEDVNLPGRWKPKMIGGLGGFIKVRQYDQIHIEICGHKVIGT
VLIGPTPVNIIGRSMLTQIGCTLNF

>102782_52 0.1
PQITLWQRPIVTIKVEGQLKEAPLDTGADDTVFEDMNLPGRWKPKMIGGLGGFIKVRQYDQIHIEICGHKVIGT
VLVGPTPVNIIGRNMLTQIGCTLNF
>102782_53 0.1
PQITLWQRPIVTIKVEGQLKEAPLDTGADDTVFEDMNLPGRWKPKMIGGLGGFIKVRQYDQIHIEICGHKVIGT
VLVGPTPVNIIGRSMLTQIGCTLNF
>102782_54 0.1
PQITLWQRPIVTIKVEGQLKEAPLDTGADDTVFEDMNLPGRWKPKMIGGLGGFIKVRQYDQIHIEICGHKVIGT
VLIGPTPVNIIGRNMLTQIGCTLNF
>102782_55 0.1
PQITLWQRPIVTIKVEGQLKEAPLDTGADDTVFEDMNLPGRWKPKMIGGLGGFIKVRQYDQIHIEICGHKVIGT
VLIGPTPVNIIGRSMLTQIGCTLNF
>102782_60 0.1
PQITLWQRPIVTIKVEGQLKEAPLDTGADDTVFEDVNLPGRWKPKMIGGLGGFIKVRQYDQIHIEICGHKVIGT
VLVGPTPVNIIGRNMLTQIGCTLNF
>102782_61 0.1
PQITLWQRPIVTIKVEGQLKEAPLDTGADDTVFEDVNLPGRWKPKMIGGLGGFIKVRQYDQIHIEICGHKVIGT
VLVGPTPVNIIGRSMLTQIGCTLNF
>102782_62 0.1
PQITLWQRPIVTIKVEGQLKEAPLDTGADDTVFEDVNLPGRWKPKMIGGLGGFIKVRQYDQIHIEICGHKVIGT
VLIGPTPVNIIGRNMLTQIGCTLNF
>102782_63 0.1
PQITLWQRPIVTIKVEGQLKEAPLDTGADDTVFEDVNLPGRWKPKMIGGLGGFIKVRQYDQIHIEICGHKVIGT
VLIGPTPVNIIGRSMLTQIGCTLNF
>102782_164 0.1
PQITLWQRPIVTIKVEGQLTEAPLDTGADDTVLEDMNLPGRWKPKMIGGLGGFIKVRQYDQIHIEICGHKVIGT
VLVGPTPVNIIGRNMLTQIGCTLNF
>102782_64 0.1
PQITLWQRPIVTIKVEGQLIEALLDTGADDTVLEDMNLPGRWKPKMIGGLGGFIKVRQYDQIHIEICGHKAIGTV
LVGPTPVNIIGRNMLTQIGCTLNF
>102782_65 0.1
PQITLWQRPIVTIKVEGQLIEALLDTGADDTVLEDMNLPGRWKPKMIGGLGGFIKVRQYDQIHIEICGHKAIGTV
LVGPTPVNIIGRSMLTQIGCTLNF
>102782_66 0.1
PQITLWQRPIVTIKVEGQLIEALLDTGADDTVLEDMNLPGRWKPKMIGGLGGFIKVRQYDQIHIEICGHKAIGTV
LIGPTPVNIIGRNMLTQIGCTLNF
>102782_67 0.1
PQITLWQRPIVTIKVEGQLIEALLDTGADDTVLEDMNLPGRWKPKMIGGLGGFIKVRQYDQIHIEICGHKAIGTV
LIGPTPVNIIGRSMLTQIGCTLNF
>102782_72 0.1
PQITLWQRPIVTIKVEGQLIEALLDTGADDTVLEDVNLPGRWKPKMIGGLGGFIKVRQYDQIHIEICGHKAIGTV
LVGPTPVNIIGRNMLTQIGCTLNF
>102782_73 0.1
PQITLWQRPIVTIKVEGQLIEALLDTGADDTVLEDVNLPGRWKPKMIGGLGGFIKVRQYDQIHIEICGHKAIGTV
LVGPTPVNIIGRSMLTQIGCTLNF
>102782_74 0.1
PQITLWQRPIVTIKVEGQLIEALLDTGADDTVLEDVNLPGRWKPKMIGGLGGFIKVRQYDQIHIEICGHKAIGTV
LIGPTPVNIIGRNMLTQIGCTLNF
>102782_75 0.1
PQITLWQRPIVTIKVEGQLIEALLDTGADDTVLEDVNLPGRWKPKMIGGLGGFIKVRQYDQIHIEICGHKAIGTV
LIGPTPVNIIGRSMLTQIGCTLNF
>102782_80 0.1
PQITLWQRPIVTIKVEGQLIEALLDTGADDTVFEDMNLPGRWKPKMIGGLGGFIKVRQYDQIHIEICGHKAIGTV
LVGPTPVNIIGRNMLTQIGCTLNF
>102782_81 0.1
PQITLWQRPIVTIKVEGQLIEALLDTGADDTVFEDMNLPGRWKPKMIGGLGGFIKVRQYDQIHIEICGHKAIGTV
LVGPTPVNIIGRSMLTQIGCTLNF
>102782_82 0.1
PQITLWQRPIVTIKVEGQLIEALLDTGADDTVFEDMNLPGRWKPKMIGGLGGFIKVRQYDQIHIEICGHKAIGTV
LIGPTPVNIIGRNMLTQIGCTLNF
>102782_83 0.1
PQITLWQRPIVTIKVEGQLIEALLDTGADDTVFEDMNLPGRWKPKMIGGLGGFIKVRQYDQIHIEICGHKAIGTV
LIGPTPVNIIGRSMLTQIGCTLNF

>102782_88 0.1
PQITLWQRPIVTIKVEGQLIEALLDTGADDTVFEDVNLPGRWKPKMIGGLGGFIKVRQYDQIHIEICGHKAIGTV
LVGPTPVNIIGRNMLTQIGCTLNF
>102782_89 0.1
PQITLWQRPIVTIKVEGQLIEALLDTGADDTVFEDVNLPGRWKPKMIGGLGGFIKVRQYDQIHIEICGHKAIGTV
LVGPTPVNIIGRSMLTQIGCTLNF
>102782_90 0.1
PQITLWQRPIVTIKVEGQLIEALLDTGADDTVFEDVNLPGRWKPKMIGGLGGFIKVRQYDQIHIEICGHKAIGTV
LIGPTPVNIIGRNMLTQIGCTLNF
>102782_91 0.1
PQITLWQRPIVTIKVEGQLIEALLDTGADDTVFEDVNLPGRWKPKMIGGLGGFIKVRQYDQIHIEICGHKAIGTV
LIGPTPVNIIGRSMLTQIGCTLNF
>102782_68 0.1
PQITLWQRPIVTIKVEGQLIEALLDTGADDTVLEDMNLPGRWKPKMIGGLGGFIKVRQYDQIHIEICGHKVIGTV
LVGPTPVNIIGRNMLTQIGCTLNF
>102782_69 0.1
PQITLWQRPIVTIKVEGQLIEALLDTGADDTVLEDMNLPGRWKPKMIGGLGGFIKVRQYDQIHIEICGHKVIGTV
LVGPTPVNIIGRSMLTQIGCTLNF
>102782_70 0.1
PQITLWQRPIVTIKVEGQLIEALLDTGADDTVLEDMNLPGRWKPKMIGGLGGFIKVRQYDQIHIEICGHKVIGTV
LIGPTPVNIIGRNMLTQIGCTLNF
>102782_71 0.1
PQITLWQRPIVTIKVEGQLIEALLDTGADDTVLEDMNLPGRWKPKMIGGLGGFIKVRQYDQIHIEICGHKVIGTV
LIGPTPVNIIGRSMLTQIGCTLNF
>102782_76 0.1
PQITLWQRPIVTIKVEGQLIEALLDTGADDTVLEDVNLPGRWKPKMIGGLGGFIKVRQYDQIHIEICGHKVIGTV
LVGPTPVNIIGRNMLTQIGCTLNF
>102782_77 0.1
PQITLWQRPIVTIKVEGQLIEALLDTGADDTVLEDVNLPGRWKPKMIGGLGGFIKVRQYDQIHIEICGHKVIGTV
LVGPTPVNIIGRSMLTQIGCTLNF
>102782_78 0.1
PQITLWQRPIVTIKVEGQLIEALLDTGADDTVLEDVNLPGRWKPKMIGGLGGFIKVRQYDQIHIEICGHKVIGTV
LIGPTPVNIIGRNMLTQIGCTLNF
>102782_79 0.1
PQITLWQRPIVTIKVEGQLIEALLDTGADDTVLEDVNLPGRWKPKMIGGLGGFIKVRQYDQIHIEICGHKVIGTV
LIGPTPVNIIGRSMLTQIGCTLNF
>102782_84 0.1
PQITLWQRPIVTIKVEGQLIEALLDTGADDTVFEDMNLPGRWKPKMIGGLGGFIKVRQYDQIHIEICGHKVIGTV
LVGPTPVNIIGRNMLTQIGCTLNF
>102782_85 0.1
PQITLWQRPIVTIKVEGQLIEALLDTGADDTVFEDMNLPGRWKPKMIGGLGGFIKVRQYDQIHIEICGHKVIGTV
LVGPTPVNIIGRSMLTQIGCTLNF
>102782_86 0.1
PQITLWQRPIVTIKVEGQLIEALLDTGADDTVFEDMNLPGRWKPKMIGGLGGFIKVRQYDQIHIEICGHKVIGTV
LIGPTPVNIIGRNMLTQIGCTLNF
>102782_87 0.1
PQITLWQRPIVTIKVEGQLIEALLDTGADDTVFEDMNLPGRWKPKMIGGLGGFIKVRQYDQIHIEICGHKVIGTV
LIGPTPVNIIGRSMLTQIGCTLNF
>102782_92 0.1
PQITLWQRPIVTIKVEGQLIEALLDTGADDTVFEDVNLPGRWKPKMIGGLGGFIKVRQYDQIHIEICGHKVIGTV
LVGPTPVNIIGRNMLTQIGCTLNF
>102782_93 0.1
PQITLWQRPIVTIKVEGQLIEALLDTGADDTVFEDVNLPGRWKPKMIGGLGGFIKVRQYDQIHIEICGHKVIGTV
LVGPTPVNIIGRSMLTQIGCTLNF
>102782_94 0.1
PQITLWQRPIVTIKVEGQLIEALLDTGADDTVFEDVNLPGRWKPKMIGGLGGFIKVRQYDQIHIEICGHKVIGTV
LIGPTPVNIIGRNMLTQIGCTLNF
>102782_95 0.1
PQITLWQRPIVTIKVEGQLIEALLDTGADDTVFEDVNLPGRWKPKMIGGLGGFIKVRQYDQIHIEICGHKVIGTV
LIGPTPVNIIGRSMLTQIGCTLNF
>102782_4 0.1
PQITLWQRPIVTIKVEGQLKEALLDTGADDTVLEDMNLPGRWKPKMIGGLGGFIKVRQYDQIHIEICGHKVIGT
VLVGPTPVNIIGRNMLTQIGCTLNF

>102782_5 0.1
PQITLWQRPIVTIKVEGQLKEALLDTGADDTVLEDMNLPGRWKPKMIGGLGGFIKVRQYDQIHIEICGHKVIGT
VLVGPTPVNIIGRSMLTQIGCTLNF
>102782_6 0.1
PQITLWQRPIVTIKVEGQLKEALLDTGADDTVLEDMNLPGRWKPKMIGGLGGFIKVRQYDQIHIEICGHKVIGT
VLIGPTPVNIIGRNMLTQIGCTLNF
>102782_7 0.1
PQITLWQRPIVTIKVEGQLKEALLDTGADDTVLEDMNLPGRWKPKMIGGLGGFIKVRQYDQIHIEICGHKVIGT
VLIGPTPVNIIGRSMLTQIGCTLNF
>102782_12 0.1
PQITLWQRPIVTIKVEGQLKEALLDTGADDTVLEDVNLPGRWKPKMIGGLGGFIKVRQYDQIHIEICGHKVIGT
VLVGPTPVNIIGRNMLTQIGCTLNF
>102782_13 0.1
PQITLWQRPIVTIKVEGQLKEALLDTGADDTVLEDVNLPGRWKPKMIGGLGGFIKVRQYDQIHIEICGHKVIGT
VLVGPTPVNIIGRSMLTQIGCTLNF
>102782_14 0.1
PQITLWQRPIVTIKVEGQLKEALLDTGADDTVLEDVNLPGRWKPKMIGGLGGFIKVRQYDQIHIEICGHKVIGT
VLIGPTPVNIIGRNMLTQIGCTLNF
>102782_15 0.1
PQITLWQRPIVTIKVEGQLKEALLDTGADDTVLEDVNLPGRWKPKMIGGLGGFIKVRQYDQIHIEICGHKVIGT
VLIGPTPVNIIGRSMLTQIGCTLNF
>102782_20 0.1
PQITLWQRPIVTIKVEGQLKEALLDTGADDTVFEDMNLPGRWKPKMIGGLGGFIKVRQYDQIHIEICGHKVIGT
VLVGPTPVNIIGRNMLTQIGCTLNF
>102782_21 0.1
PQITLWQRPIVTIKVEGQLKEALLDTGADDTVFEDMNLPGRWKPKMIGGLGGFIKVRQYDQIHIEICGHKVIGT
VLVGPTPVNIIGRSMLTQIGCTLNF
>102782_22 0.1
PQITLWQRPIVTIKVEGQLKEALLDTGADDTVFEDMNLPGRWKPKMIGGLGGFIKVRQYDQIHIEICGHKVIGT
VLIGPTPVNIIGRNMLTQIGCTLNF
>102782_23 0.1
PQITLWQRPIVTIKVEGQLKEALLDTGADDTVFEDMNLPGRWKPKMIGGLGGFIKVRQYDQIHIEICGHKVIGT
VLIGPTPVNIIGRSMLTQIGCTLNF
>102782_30 0.1
PQITLWQRPIVTIKVEGQLKEALLDTGADDTVFEDVNLPGRWKPKMIGGLGGFIKVRQYDQIHIEICGHKVIGT
VLIGPTPVNIIGRNMLTQIGCTLNF
>102782_31 0.1
PQITLWQRPIVTIKVEGQLKEALLDTGADDTVFEDVNLPGRWKPKMIGGLGGFIKVRQYDQIHIEICGHKVIGT
VLIGPTPVNIIGRSMLTQIGCTLNF
>102782_132 0.1
PQITLWQRPIVTIKVEGQLTEALLDTGADDTVLEDMNLPGRWKPKMIGGLGGFIKVRQYDQIHIEICGHKVIGT
VLVGPTPVNIIGRNMLTQIGCTLNF
>102782_133 0.1
PQITLWQRPIVTIKVEGQLTEALLDTGADDTVLEDMNLPGRWKPKMIGGLGGFIKVRQYDQIHIEICGHKVIGT
VLVGPTPVNIIGRSMLTQIGCTLNF
>102782_134 0.1
PQITLWQRPIVTIKVEGQLTEALLDTGADDTVLEDMNLPGRWKPKMIGGLGGFIKVRQYDQIHIEICGHKVIGT
VLIGPTPVNIIGRNMLTQIGCTLNF
>102782_135 0.1
PQITLWQRPIVTIKVEGQLTEALLDTGADDTVLEDMNLPGRWKPKMIGGLGGFIKVRQYDQIHIEICGHKVIGT
VLIGPTPVNIIGRSMLTQIGCTLNF
>102782_140 0.1
PQITLWQRPIVTIKVEGQLTEALLDTGADDTVLEDVNLPGRWKPKMIGGLGGFIKVRQYDQIHIEICGHKVIGTV
LVGPTPVNIIGRNMLTQIGCTLNF
>102782_141 0.1
PQITLWQRPIVTIKVEGQLTEALLDTGADDTVLEDVNLPGRWKPKMIGGLGGFIKVRQYDQIHIEICGHKVIGTV
LVGPTPVNIIGRSMLTQIGCTLNF
>102782_142 0.1
PQITLWQRPIVTIKVEGQLTEALLDTGADDTVLEDVNLPGRWKPKMIGGLGGFIKVRQYDQIHIEICGHKVIGTV
LIGPTPVNIIGRNMLTQIGCTLNF
>102782_143 0.1
PQITLWQRPIVTIKVEGQLTEALLDTGADDTVLEDVNLPGRWKPKMIGGLGGFIKVRQYDQIHIEICGHKVIGTV
LIGPTPVNIIGRSMLTQIGCTLNF

>102782_148 0.1
PQITLWQRPIVTIKVEGQLTEALLDTGADDTVFEDMNLPGRWKPKMIGGLGGFIKVRQYDQIHIEICGHKVIGT
VLVGPTPVNIIGRNMLTQIGCTLNF
>102782_149 0.1
PQITLWQRPIVTIKVEGQLTEALLDTGADDTVFEDMNLPGRWKPKMIGGLGGFIKVRQYDQIHIEICGHKVIGT
VLVGPTPVNIIGRSMLTQIGCTLNF
>102782_150 0.1
PQITLWQRPIVTIKVEGQLTEALLDTGADDTVFEDMNLPGRWKPKMIGGLGGFIKVRQYDQIHIEICGHKVIGT
VLIGPTPVNIIGRNMLTQIGCTLNF
>102782_151 0.1
PQITLWQRPIVTIKVEGQLTEALLDTGADDTVFEDMNLPGRWKPKMIGGLGGFIKVRQYDQIHIEICGHKVIGT
VLIGPTPVNIIGRSMLTQIGCTLNF
>102782_2 0.1
PQITLWQRPIVTIKVEGQLKEALLDTGADDTVLEDMNLPGRWKPKMIGGLGGFIKVRQYDQIHIEICGHKAIGT
VLIGPTPVNIIGRNMLTQIGCTLNF
>102782_3 0.1
PQITLWQRPIVTIKVEGQLKEALLDTGADDTVLEDMNLPGRWKPKMIGGLGGFIKVRQYDQIHIEICGHKAIGT
VLIGPTPVNIIGRSMLTQIGCTLNF
>102782_8 0.1
PQITLWQRPIVTIKVEGQLKEALLDTGADDTVLEDVNLPGRWKPKMIGGLGGFIKVRQYDQIHIEICGHKAIGT
VLVGPTPVNIIGRNMLTQIGCTLNF
>102782_10 0.1
PQITLWQRPIVTIKVEGQLKEALLDTGADDTVLEDVNLPGRWKPKMIGGLGGFIKVRQYDQIHIEICGHKAIGT
VLIGPTPVNIIGRNMLTQIGCTLNF
>102782_11 0.1
PQITLWQRPIVTIKVEGQLKEALLDTGADDTVLEDVNLPGRWKPKMIGGLGGFIKVRQYDQIHIEICGHKAIGT
VLIGPTPVNIIGRSMLTQIGCTLNF
>102782_16 0.1
PQITLWQRPIVTIKVEGQLKEALLDTGADDTVFEDMNLPGRWKPKMIGGLGGFIKVRQYDQIHIEICGHKAIGT
VLVGPTPVNIIGRNMLTQIGCTLNF
>102782_17 0.1
PQITLWQRPIVTIKVEGQLKEALLDTGADDTVFEDMNLPGRWKPKMIGGLGGFIKVRQYDQIHIEICGHKAIGT
VLVGPTPVNIIGRSMLTQIGCTLNF
>102782_18 0.1
PQITLWQRPIVTIKVEGQLKEALLDTGADDTVFEDMNLPGRWKPKMIGGLGGFIKVRQYDQIHIEICGHKAIGT
VLIGPTPVNIIGRNMLTQIGCTLNF
>102782_19 0.1
PQITLWQRPIVTIKVEGQLKEALLDTGADDTVFEDMNLPGRWKPKMIGGLGGFIKVRQYDQIHIEICGHKAIGT
VLIGPTPVNIIGRSMLTQIGCTLNF
>102782_24 0.1
PQITLWQRPIVTIKVEGQLKEALLDTGADDTVFEDVNLPGRWKPKMIGGLGGFIKVRQYDQIHIEICGHKAIGT
VLVGPTPVNIIGRNMLTQIGCTLNF
>102782_25 0.1
PQITLWQRPIVTIKVEGQLKEALLDTGADDTVFEDVNLPGRWKPKMIGGLGGFIKVRQYDQIHIEICGHKAIGT
VLVGPTPVNIIGRSMLTQIGCTLNF
>102782_26 0.1
PQITLWQRPIVTIKVEGQLKEALLDTGADDTVFEDVNLPGRWKPKMIGGLGGFIKVRQYDQIHIEICGHKAIGT
VLIGPTPVNIIGRNMLTQIGCTLNF
>13255_0 0.1
PQITLWQRPLVTIKIGGQLKEALLDTGADDTVLEEMNLPGRWKPKMIGGIGGFIKVGQYDQILIEICGHKAIGTV
LVGPTPVNIIGRSLLTQIGCTLNF

Sequences for the IDV-resistant ensemble
>144045_0 500.0
PQITLWQRPFIPVKVGGQPIEALLDTGADDTVLEGINLPGRWKPKIIGGIGGFVKVRQYDQIPIEICGHKVLSTV
LVGPTPVNVIGRNLMTQLGCTLNF
>102993_0 500.0
PQIALWQRPIVTVKVGGQLIEALLDTGADDTVLEDINLPGRWKPKIIGGIGGFVKVKEYENVPIEICGHKATCSV
LVGPTPFNIIGRNLMTKIGCTLNF
>102993_1 500.0
PQIALWQRPIVTVKVGGQLIEALLDTGADDTVLENINLPGRWKPKIIGGIGGFVKVKEYENVPIEICGHKATCSV
LVGPTPFNIIGRNLMTKIGCTLNF
>116523_0 500.0

PQITLWQRPVVTIKIGGQLKEALLDTGADDTVLEEMDLPGRWKPKMILGIGGFSKVRQYDQVPIEICGYKVIGS
VLIGPTPANIIGRNLLTQLGCTLNF
>116523_1 500.0
PQITLWQRPVVTIKIGGQLKEALLDTGADDTVLEEMDLPGRWKPKMILGIGGYSKVRQYDQVPIEICGYKVIGS
VLIGPTPANIIGRNLLTQLGCTLNF
>116523_2 500.0
PQITLWQRPVVTIKIGGQLKEALLDTGADDTVLEEMDLPGRWKPKMIVGIGGFSKVRQYDQVPIEICGYKVIGS
VLIGPTPANIIGRNLLTQLGCTLNF
>116523_3 500.0
PQITLWQRPVVTIKIGGQLKEALLDTGADDTVLEEMDLPGRWKPKMIVGIGGYSKVRQYDQVPIEICGYKVIGS
VLIGPTPANIIGRNLLTQLGCTLNF
>116523_4 500.0
PQITLWQRPVVTIKIGGQLKEALLDTGADDTVLEEMDLPGRWKPKIILGIGGFSKVRQYDQVPIEICGYKVIGSV
LIGPTPANIIGRNLLTQLGCTLNF
>116523_5 500.0
PQITLWQRPVVTIKIGGQLKEALLDTGADDTVLEEMDLPGRWKPKIILGIGGYSKVRQYDQVPIEICGYKVIGSV
LIGPTPANIIGRNLLTQLGCTLNF
>116523_6 500.0
PQITLWQRPVVTIKIGGQLKEALLDTGADDTVLEEMDLPGRWKPKIIVGIGGFSKVRQYDQVPIEICGYKVIGS
VLIGPTPANIIGRNLLTQLGCTLNF
>116523_7 500.0
PQITLWQRPVVTIKIGGQLKEALLDTGADDTVLEEMDLPGRWKPKIIVGIGGYSKVRQYDQVPIEICGYKVIGS
VLIGPTPANIIGRNLLTQLGCTLNF
>116523_8 500.0
PQITLWQRPVVTIKIGGQLKEALLDTGADDTVLEEMDLPGRWTPKMILGIGGFSKVRQYDQVPIEICGYKVIGS
VLIGPTPANIIGRNLLTQLGCTLNF
>116523_9 500.0
PQITLWQRPVVTIKIGGQLKEALLDTGADDTVLEEMDLPGRWTPKMILGIGGYSKVRQYDQVPIEICGYKVIGS
VLIGPTPANIIGRNLLTQLGCTLNF
>116523_10 500.0
PQITLWQRPVVTIKIGGQLKEALLDTGADDTVLEEMDLPGRWTPKMIVGIGGFSKVRQYDQVPIEICGYKVIGS
VLIGPTPANIIGRNLLTQLGCTLNF
>116523_11 500.0
PQITLWQRPVVTIKIGGQLKEALLDTGADDTVLEEMDLPGRWTPKMIVGIGGYSKVRQYDQVPIEICGYKVIGS
VLIGPTPANIIGRNLLTQLGCTLNF
>116523_12 500.0
PQITLWQRPVVTIKIGGQLKEALLDTGADDTVLEEMDLPGRWTPKIILGIGGFSKVRQYDQVPIEICGYKVIGSV
LIGPTPANIIGRNLLTQLGCTLNF
>116523_13 500.0
PQITLWQRPVVTIKIGGQLKEALLDTGADDTVLEEMDLPGRWTPKIILGIGGYSKVRQYDQVPIEICGYKVIGSV
LIGPTPANIIGRNLLTQLGCTLNF
>116523_14 500.0
PQITLWQRPVVTIKIGGQLKEALLDTGADDTVLEEMDLPGRWTPKIIVGIGGFSKVRQYDQVPIEICGYKVIGSV
LIGPTPANIIGRNLLTQLGCTLNF
>116523_15 500.0
PQITLWQRPVVTIKIGGQLKEALLDTGADDTVLEEMDLPGRWTPKIIVGIGGYSKVRQYDQVPIEICGYKVIGS
VLIGPTPANIIGRNLLTQLGCTLNF
>81917_0 500.0
PQITLWQRPIVTIKIGGQLKEALLDTGADDTVLEEMNLPGRWTPKMIVGIGGFTKVRQYDQILVEICGHKVIGSV
LIGPTPANIIGRNLLTQIGCTLNF
>81917_1 500.0
PQITLWQRPIVTIKIGGQLKEALLDTGADDTVLEEMNLPGRWTPKMIVGIGGFTKVRQYDQVLVEICGHKVIGS
VLIGPTPANIIGRNLLTQIGCTLNF
>54177_0 400.0
PQITLWQRPVVTIKIGGQLIEALLDTGADDTVLEEINLPGRWKPKIIGGIGGFIKVRQYDQILIEICGHKVISTVVV
GPTPVNAIGRNLLTQIGCTLNF
>103057_1 500.0
PQITLWQRPIVTVKIGGQLREALLDTGADDTVFEDIELPGRWTPKIIGGIGGFVKVKQYEEVPIEICEHKTTTTVL
VGPTPFNIIGRNLMTQLGCTLNF
>103057_3 500.0
PQITLWQRPIVTVKIGGQLREALLDTGADDTVFEDIELPGRWTPKIIGGIGGFVKVKQYEEVPVEICEHKTTTTV
LVGPTPFNIIGRNLMTQLGCTLNF
>78924_3 500.0

PQITLWQRPVVTIKIGGQIKEALLDTGADDTVLEEMNLPGRWKPKLIVGIGGYAKVREYEEVPIEICGHKVVGS
VLIGPTPTNIIGRNLMTQLGCTLNF
>78924_7 500.0
PQITLWQRPVVKIKIGGQIKEALLDTGADDTVLEEMNLPGRWKPKLIVGIGGYAKVREYEEVPIEICGHKVVGS
VLIGPTPTNIIGRNLMTQLGCTLNF
>81911_4 500.0
PQITLWQRPIVPVKIGGQQIEALLDTGADDTVLEDINLPGRWKPKIIGGIGGFVKVRQYDQVPVEICGHKVVGT
VVVGPTPFNIIGRNLMTQIGCTLNF
>81911_5 500.0
PQITLWQRPIVPVKIGGQQIEALLDTGADDTVLEDINLPGRWKPKIIGGIGGFVKVKQYDQVPVEICGHKVVGT
VVVGPTPFNIIGRNLMTQIGCTLNF
>81911_6 500.0
PQITLWQRPIVPVKVGGQQIEALLDTGADDTVLEDINLPGRWKPKIIGGIGGFVKVRQYDQVPVEICGHKVVG
TVVVGPTPFNIIGRNLMTQIGCTLNF
>81911_7 500.0
PQITLWQRPIVPVKVGGQQIEALLDTGADDTVLEDINLPGRWKPKIIGGIGGFVKVKQYDQVPVEICGHKVVGT
VVVGPTPFNIIGRNLMTQIGCTLNF
>61115_0 400.0
PQITLWQRPIITVKIGGQTREVLLDTGADDTVIEDIDLPGRWKPKLIGGIGGFVKVREYNQVPIEICGHKVLSTVL
VGPTPVNVIGRNLMTQIGCTLNF
>61115_1 400.0
PQITLWQRPIITVKIGGQTREVLLDTGADDTVIEDIDLPGRWKPKLIGGIGGFVKVREYNQVPIEICGHKVVSTV
LVGPTPVNVIGRNLMTQIGCTLNF
>61115_2 400.0
PQITLWQRPIITVKIGGQTREVLLDTGADDTVIEDIDLPGRWKPKLIGGIGGFVKVREYNQVPIEICGRKVLSTVL
VGPTPVNVIGRNLMTQIGCTLNF
>61115_3 400.0
PQITLWQRPIITVKIGGQTREVLLDTGADDTVIEDIDLPGRWKPKLIGGIGGFVKVREYNQVPIEICGRKVVSTV
LVGPTPVNVIGRNLMTQIGCTLNF
>78924_2 500.0
PQITLWQRPVVTIKIGGQIKEALLDTGADDTVLEEMNLPGRWKPKLIVGIGGYAKVREYEEVPIEICGHKVVGS
VLIGPTPANIIGRNLMTQLGCTLNF
>78924_6 500.0
PQITLWQRPVVKIKIGGQIKEALLDTGADDTVLEEMNLPGRWKPKLIVGIGGYAKVREYEEVPIEICGHKVVGS
VLIGPTPANIIGRNLMTQLGCTLNF
>117023_0 500.0
PQITLWQRPIVTIKIGGQLREALLDTGADDTVLEDIELPGRWKPKMIVGVGGFAKVRQYDQVPIEICGHKVVGT
VLVGPTPANIIGRNLMTQLGCTLNF
>117023_1 500.0
PQITLWQRPIVTIKIGGQLREALLDTGADDTVLEDIELPGRWKPKMIVGVGGFAKVKQYDQVPIEICGHKVVGT
VLVGPTPANIIGRNLMTQLGCTLNF
>86640_0 500.0
PQITLWQRPFVTMKIGGQLIEALLDTGADDTVLEEIDLPGRWKPKIIGGIGGFVKVRQYDQVPIEIFGHKTTTTV
LVGPTPVNVIGRNLMTQLGCTLNF
>86640_1 500.0
PQITLWQRPFVTMKIGGQLIEALLDTGADDTVLEEIDLPGRWKPKIIGGIGGFVKVRQYDQVPIEVFGHKTTTT
VLVGPTPVNVIGRNLMTQLGCTLNF
>86890_0 500.0
PQITLWQRPIVTIKVGGQLKEALLDTGADDTVFQELDLPGRWKPKLIVGIGGFTKVRQYDQVPIEICGHKVVGD
ILVGPTPANIIGRNLMTQLGCTLNF
>86890_1 500.0
PQITLWQRPIVTIKVGGQLKEALLDTGADDTVFQELDLPGKWKPKLIVGIGGFTKVRQYDQVPIEICGHKVVGD
ILVGPTPANIIGRNLMTQLGCTLNF
>86890_2 500.0
PQITLWQRPIVTVKVGGQLKEALLDTGADDTVFQELDLPGRWKPKLIVGIGGFTKVRQYDQVPIEICGHKVVG
DILVGPTPANIIGRNLMTQLGCTLNF
>86890_3 500.0
PQITLWQRPIVTVKVGGQLKEALLDTGADDTVFQELDLPGKWKPKLIVGIGGFTKVRQYDQVPIEICGHKVVG
DILVGPTPANIIGRNLMTQLGCTLNF
>86890_4 500.0
PQITLWQRPVVTIKVGGQLKEALLDTGADDTVFQELDLPGRWKPKLIVGIGGFTKVRQYDQVPIEICGHKVVG
DILVGPTPANIIGRNLMTQLGCTLNF
>86890_5 500.0

PQITLWQRPVVTIKVGGQLKEALLDTGADDTVFQELDLPGKWKPKLIVGIGGFTKVRQYDQVPIEICGHKVVG
DILVGPTPANIIGRNLMTQLGCTLNF
>86890_6 500.0
PQITLWQRPVVTVKVGGQLKEALLDTGADDTVFQELDLPGRWKPKLIVGIGGFTKVRQYDQVPIEICGHKVV
GDILVGPTPANIIGRNLMTQLGCTLNF
>86890_7 500.0
PQITLWQRPVVTVKVGGQLKEALLDTGADDTVFQELDLPGKWKPKLIVGIGGFTKVRQYDQVPIEICGHKVV
GDILVGPTPANIIGRNLMTQLGCTLNF
>117033_1 500.0
PQITLWQRPIVTIKIGGQIREALLDTGADDTVLEDINLPGRWKPKLIVGIGGFSKVKEYDQVPIEICGHKVVGTVL
VGPTPANIIGRNLLTQLGCTLNF
>117033_2 500.0
PQITLWQRPIVTIKIGGQIREALLDTGADDTVLEDINLPGRWKPKLIVGIGGFTKVKEYDQVPIEICGHKVVGTVL
VGPTPANIIGRNLLTQLGCTLNF
>117033_4 500.0
PQITLWQRPIVTIKIGGQIREALLDTGADDTVLEDINLPGRWEPKLIVGIGGFSKVKEYDQVPIEICGHKVVGTVL
VGPTPANIIGRNLLTQLGCTLNF
>117033_5 500.0
PQITLWQRPIVTIKIGGQIREALLDTGADDTVLEDINLPGRWEPKLIVGIGGFTKVKEYDQVPIEICGHKVVGTVL
VGPTPANIIGRNLLTQLGCTLNF
>147553_0 500.0
PQITLWQRPIVTIKIGGQLREALLDTGADDTVFTDIDLPGRWTPKIIVGVGGFSKVKQYDQVPIEICGHKVVGTV
LIGPTPANIVGRNLLTQLGCTLNF
>103057_0 500.0
PQITLWQRPIVTVKIGGQLREALLDTGADDTVFEDIELPGRWTPKIIGGIGGFVKVKQYEEVPIEICEHKTTATVL
VGPTPFNIIGRNLMTQLGCTLNF
>103057_2 500.0
PQITLWQRPIVTVKIGGQLREALLDTGADDTVFEDIELPGRWTPKIIGGIGGFVKVKQYEEVPVEICEHKTTATV
LVGPTPFNIIGRNLMTQLGCTLNF
>123049_0 500.0
PQITLWQRPIITIKIGGQLKDVLMDTGADDTVLEDIDLPGRWKPKLIGGIGGFVKVRQYDHVPVEVCGHKVTTT
VLVGPTPVDVIGRNLLTQIGCTLNF
>123049_1 500.0
PQITLWQRPIITIKIGGQLKDVLMDTGADDTVLEDIDLPGRWKPKLIGGIGGFVKVRQYDNVPVEVCGHKVTTT
VLVGPTPVDVIGRNLLTQIGCTLNF
>123049_2 500.0
PQITLWQRPIITIKIGGQLKDVLMDTGADDTVLEDIDLPGRWKPKLIGGIGGFVKVKQYDHVPVEVCGHKVTTT
VLVGPTPVDVIGRNLLTQIGCTLNF
>123049_3 500.0
PQITLWQRPIITIKIGGQLKDVLMDTGADDTVLEDIDLPGRWKPKLIGGIGGFVKVKQYDNVPVEVCGHKVTTT
VLVGPTPVDVIGRNLLTQIGCTLNF
>123049_4 500.0
PQITLWQRPIITIKIGGQLKDVLMDTGADDTVLEDIDLPGKWKPKLIGGIGGFVKVRQYDHVPVEVCGHKVTTT
VLVGPTPVDVIGRNLLTQIGCTLNF
>123049_5 500.0
PQITLWQRPIITIKIGGQLKDVLMDTGADDTVLEDIDLPGKWKPKLIGGIGGFVKVRQYDNVPVEVCGHKVTTT
VLVGPTPVDVIGRNLLTQIGCTLNF
>123049_6 500.0
PQITLWQRPIITIKIGGQLKDVLMDTGADDTVLEDIDLPGKWKPKLIGGIGGFVKVKQYDHVPVEVCGHKVTTT
VLVGPTPVDVIGRNLLTQIGCTLNF
>123049_7 500.0
PQITLWQRPIITIKIGGQLKDVLMDTGADDTVLEDIDLPGKWKPKLIGGIGGFVKVKQYDNVPVEVCGHKVTTT
VLVGPTPVDVIGRNLLTQIGCTLNF
>123049_8 500.0
PQITLWQRPIITIKIGGQLKDVLMDTGADDTVLENIDLPGRWKPKLIGGIGGFVKVRQYDHVPVEVCGHKVTTT
VLVGPTPVDVIGRNLLTQIGCTLNF
>123049_9 500.0
PQITLWQRPIITIKIGGQLKDVLMDTGADDTVLENIDLPGRWKPKLIGGIGGFVKVRQYDNVPVEVCGHKVTTT
VLVGPTPVDVIGRNLLTQIGCTLNF
>123049_10 500.0
PQITLWQRPIITIKIGGQLKDVLMDTGADDTVLENIDLPGRWKPKLIGGIGGFVKVKQYDHVPVEVCGHKVTTT
VLVGPTPVDVIGRNLLTQIGCTLNF
>123049_11 500.0

PQITLWQRPIITIKIGGQLKDVLMDTGADDTVLENIDLPGRWKPKLIGGIGGFVKVKQYDNVPVEVCGHKVTTT
VLVGPTPVDVIGRNLLTQIGCTLNF
>123049_12 500.0
PQITLWQRPIITIKIGGQLKDVLMDTGADDTVLENIDLPGKWKPKLIGGIGGFVKVRQYDHVPVEVCGHKVTTT
VLVGPTPVDVIGRNLLTQIGCTLNF
>123049_13 500.0
PQITLWQRPIITIKIGGQLKDVLMDTGADDTVLENIDLPGKWKPKLIGGIGGFVKVRQYDNVPVEVCGHKVTTT
VLVGPTPVDVIGRNLLTQIGCTLNF
>123049_14 500.0
PQITLWQRPIITIKIGGQLKDVLMDTGADDTVLENIDLPGKWKPKLIGGIGGFVKVKQYDHVPVEVCGHKVTTT
VLVGPTPVDVIGRNLLTQIGCTLNF
>123049_15 500.0
PQITLWQRPIITIKIGGQLKDVLMDTGADDTVLENIDLPGKWKPKLIGGIGGFVKVKQYDNVPVEVCGHKVTTT
VLVGPTPVDVIGRNLLTQIGCTLNF
>98601_0 400.0
PQITLWQRPFVTIKIGGQLIEALLDTGADDTVLEEMNLPGRWKPKIIGGIGGFMKVRQYDQIPIEICGHKVITTVL
IGPTPVNAIGRNLLTQIGCTLNF
>113192_1 500.0
PQITLWQRPFVTMKIGGQLIEALLDTGADDTVLEEISLPGRWKPKIIGGIGGFVKVRQYDQVPIEVFGHKATTT
VLVGPTPVNVIGRNLMTQLGCTLNF
>113192_3 500.0
PQITLWQRPFVTMKIGGQLIEALLDTGADDTVLEEISLPGRWKPKIIGGIGGFVKVRQYDQVPIEVFGQKATTT
VLVGPTPVNVIGRNLMTQLGCTLNF
>113192_5 500.0
PQITLWQRPFVTMKIGGQLIEALLDTGADDTVLEEISLPGRWKPKIIGGIGGFVKVRQYDQVPVEVFGHKATTT
VLVGPTPVNVIGRNLMTQLGCTLNF
>113192_7 500.0
PQITLWQRPFVTMKIGGQLIEALLDTGADDTVLEEISLPGRWKPKIIGGIGGFVKVRQYDQVPVEVFGQKATTT
VLVGPTPVNVIGRNLMTQLGCTLNF
>113192_9 500.0
PQITLWQRPFVTMKIGGQIIEALLDTGADDTVLEEISLPGRWKPKIIGGIGGFVKVRQYDQVPIEVFGHKATTTV
LVGPTPVNVIGRNLMTQLGCTLNF
>113192_11 500.0
PQITLWQRPFVTMKIGGQIIEALLDTGADDTVLEEISLPGRWKPKIIGGIGGFVKVRQYDQVPIEVFGQKATTTV
LVGPTPVNVIGRNLMTQLGCTLNF
>113192_13 500.0
PQITLWQRPFVTMKIGGQIIEALLDTGADDTVLEEISLPGRWKPKIIGGIGGFVKVRQYDQVPVEVFGHKATTT
VLVGPTPVNVIGRNLMTQLGCTLNF
>113192_15 500.0
PQITLWQRPFVTMKIGGQIIEALLDTGADDTVLEEISLPGRWKPKIIGGIGGFVKVRQYDQVPVEVFGQKATTT
VLVGPTPVNVIGRNLMTQLGCTLNF
>56150_0 400.0
PQITLWRRPVVTIKVGGQLTEALLDTGADDTVFEDINLPGRWTPKMIMGIGGLVKVRQYDQVPIEFCGHKVLS
TVLVGPTPANIIGRNLMTQIGCTLNF
>56150_1 400.0
PQITLWRRPVVTIKVGGQLTEALLDTGADDTVFEDINLPGRWTPKMIMGIGGLVKVRQYDQVPIEFCGRKVLS
TVLVGPTPANIIGRNLMTQIGCTLNF
>56150_2 400.0
PQITLWRRPVVTIKVGGQLTEALLDTGADDTVFEDINLPGRWTPKMIMGIGGLVKVKQYDQVPIEFCGHKVLS
TVLVGPTPANIIGRNLMTQIGCTLNF
>73552_0 400.0
PQITLWQRPIVTIKIGGQLREALLDTGADDTILEDLDLPGRWKPKIVGGIGGFIKVRQYDQIPMEIEGHKVVGTV
LVGPTPTNIIGRNVMTQLGCTLNF
>73552_3 400.0
PQITLWQRPIVTIKIGGQLREALLDTGADDTILEDLDLPGRWKPKIVGGIGGFIKVKQYDQIPMEIEGHKVVGTV
LVGPTPTNIVGRNVMTQLGCTLNF
>81911_0 500.0
PQITLWQRPIVTVKIGGQQIEALLDTGADDTVLEDINLPGRWKPKIIGGIGGFVKVRQYDQVPVEICGHKVVGT
VVVGPTPFNIIGRNLMTQIGCTLNF
>81911_1 500.0
PQITLWQRPIVTVKIGGQQIEALLDTGADDTVLEDINLPGRWKPKIIGGIGGFVKVKQYDQVPVEICGHKVVGT
VVVGPTPFNIIGRNLMTQIGCTLNF
>81911_2 500.0

PQITLWQRPIVTVKVGGQQIEALLDTGADDTVLEDINLPGRWKPKIIGGIGGFVKVRQYDQVPVEICGHKVVGT
VVVGPTPFNIIGRNLMTQIGCTLNF
>81911_3 500.0
PQITLWQRPIVTVKVGGQQIEALLDTGADDTVLEDINLPGRWKPKIIGGIGGFVKVKQYDQVPVEICGHKVVGT
VVVGPTPFNIIGRNLMTQIGCTLNF
>115081_0 500.0
PQITLWQRPIVTIKIGGQLREALLDTGADDTVLEDIDLPGRWKPKLIVGIGGFVKVRQYEQVPIEICGHKVVGTV
LIGPTPSNIIGRNLMTQLGCTLNF
>117033_0 500.0
PQITLWQRPIVTIKIGGQIREALLDTGADDTVLEDINLPGRWKPKLIVGIGGFCKVKEYDQVPIEICGHKVVGTV
LVGPTPANIIGRNLLTQLGCTLNF
>117033_3 500.0
PQITLWQRPIVTIKIGGQIREALLDTGADDTVLEDINLPGRWEPKLIVGIGGFCKVKEYDQVPIEICGHKVVGTV
LVGPTPANIIGRNLLTQLGCTLNF
>113192_0 500.0
PQITLWQRPFVTMKIGGQLIEALLDTGADDTVLEEISLPGRWKPKIIGGIGGFVKVRQYDQVPIEVFGHKAITTV
LVGPTPVNVIGRNLMTQLGCTLNF
>113192_2 500.0
PQITLWQRPFVTMKIGGQLIEALLDTGADDTVLEEISLPGRWKPKIIGGIGGFVKVRQYDQVPIEVFGQKAITTV
LVGPTPVNVIGRNLMTQLGCTLNF
>113192_6 500.0
PQITLWQRPFVTMKIGGQLIEALLDTGADDTVLEEISLPGRWKPKIIGGIGGFVKVRQYDQVPVEVFGQKAITT
VLVGPTPVNVIGRNLMTQLGCTLNF
>113192_8 500.0
PQITLWQRPFVTMKIGGQIIEALLDTGADDTVLEEISLPGRWKPKIIGGIGGFVKVRQYDQVPIEVFGHKAITTV
LVGPTPVNVIGRNLMTQLGCTLNF
>113192_10 500.0
PQITLWQRPFVTMKIGGQIIEALLDTGADDTVLEEISLPGRWKPKIIGGIGGFVKVRQYDQVPIEVFGQKAITTV
LVGPTPVNVIGRNLMTQLGCTLNF
>113192_12 500.0
PQITLWQRPFVTMKIGGQIIEALLDTGADDTVLEEISLPGRWKPKIIGGIGGFVKVRQYDQVPVEVFGHKAITTV
LVGPTPVNVIGRNLMTQLGCTLNF
>113192_14 500.0
PQITLWQRPFVTMKIGGQIIEALLDTGADDTVLEEISLPGRWKPKIIGGIGGFVKVRQYDQVPVEVFGQKAITTV
LVGPTPVNVIGRNLMTQLGCTLNF
>98603_0 400.0
PQITLWQRPIVTIKIGGQLKEALLDTGADDTVFEEMNLPGRWKPKIIGGIGGFVKVRQYDQIPIEICGHKVIGTV
VVGPTPVNAIGRNLLTQIGCTLNF

Sequences for the IDV-susceptible ensemble
>102782_114 0.3
PQITLWQRPIVTIKVEGQLIEAPLDTGADDTVFEDMNLPGRWKPKMIGGLGGFIKVRQYDQIHIEICGHKAIGTV
LIGPTPVNIIGRNMLTQIGCTLNF
>102782_115 0.3
PQITLWQRPIVTIKVEGQLIEAPLDTGADDTVFEDMNLPGRWKPKMIGGLGGFIKVRQYDQIHIEICGHKAIGTV
LIGPTPVNIIGRSMLTQIGCTLNF
>102782_120 0.3
PQITLWQRPIVTIKVEGQLIEAPLDTGADDTVFEDVNLPGRWKPKMIGGLGGFIKVRQYDQIHIEICGHKAIGTV
LVGPTPVNIIGRNMLTQIGCTLNF
>102782_121 0.3
PQITLWQRPIVTIKVEGQLIEAPLDTGADDTVFEDVNLPGRWKPKMIGGLGGFIKVRQYDQIHIEICGHKAIGTV
LVGPTPVNIIGRSMLTQIGCTLNF
>102782_122 0.3
PQITLWQRPIVTIKVEGQLIEAPLDTGADDTVFEDVNLPGRWKPKMIGGLGGFIKVRQYDQIHIEICGHKAIGTV
LIGPTPVNIIGRNMLTQIGCTLNF
>102782_123 0.3
PQITLWQRPIVTIKVEGQLIEAPLDTGADDTVFEDVNLPGRWKPKMIGGLGGFIKVRQYDQIHIEICGHKAIGTV
LIGPTPVNIIGRSMLTQIGCTLNF
>102782_100 0.3
PQITLWQRPIVTIKVEGQLIEAPLDTGADDTVLEDMNLPGRWKPKMIGGLGGFIKVRQYDQIHIEICGHKVIGTV
LVGPTPVNIIGRNMLTQIGCTLNF
>102782_101 0.3
PQITLWQRPIVTIKVEGQLIEAPLDTGADDTVLEDMNLPGRWKPKMIGGLGGFIKVRQYDQIHIEICGHKVIGTV

LVGPTPVNIIGRSMLTQIGCTLNF
>102782_102 0.3
PQITLWQRPIVTIKVEGQLIEAPLDTGADDTVLEDMNLPGRWKPKMIGGLGGFIKVRQYDQIHIEICGHKVIGTV
LIGPTPVNIIGRNMLTQIGCTLNF
>102782_103 0.3
PQITLWQRPIVTIKVEGQLIEAPLDTGADDTVLEDMNLPGRWKPKMIGGLGGFIKVRQYDQIHIEICGHKVIGTV
LIGPTPVNIIGRSMLTQIGCTLNF
>102782_108 0.3
PQITLWQRPIVTIKVEGQLIEAPLDTGADDTVLEDVNLPGRWKPKMIGGLGGFIKVRQYDQIHIEICGHKVIGTV
LVGPTPVNIIGRNMLTQIGCTLNF
>102782_109 0.3
PQITLWQRPIVTIKVEGQLIEAPLDTGADDTVLEDVNLPGRWKPKMIGGLGGFIKVRQYDQIHIEICGHKVIGTV
LVGPTPVNIIGRSMLTQIGCTLNF
>102782_110 0.3
PQITLWQRPIVTIKVEGQLIEAPLDTGADDTVLEDVNLPGRWKPKMIGGLGGFIKVRQYDQIHIEICGHKVIGTV
LIGPTPVNIIGRNMLTQIGCTLNF
>102782_111 0.3
PQITLWQRPIVTIKVEGQLIEAPLDTGADDTVLEDVNLPGRWKPKMIGGLGGFIKVRQYDQIHIEICGHKVIGTV
LIGPTPVNIIGRSMLTQIGCTLNF
>102782_116 0.3
PQITLWQRPIVTIKVEGQLIEAPLDTGADDTVFEDMNLPGRWKPKMIGGLGGFIKVRQYDQIHIEICGHKVIGTV
LVGPTPVNIIGRNMLTQIGCTLNF
>102782_117 0.3
PQITLWQRPIVTIKVEGQLIEAPLDTGADDTVFEDMNLPGRWKPKMIGGLGGFIKVRQYDQIHIEICGHKVIGTV
LVGPTPVNIIGRSMLTQIGCTLNF
>102782_118 0.3
PQITLWQRPIVTIKVEGQLIEAPLDTGADDTVFEDMNLPGRWKPKMIGGLGGFIKVRQYDQIHIEICGHKVIGTV
LIGPTPVNIIGRNMLTQIGCTLNF
>102782_119 0.3
PQITLWQRPIVTIKVEGQLIEAPLDTGADDTVFEDMNLPGRWKPKMIGGLGGFIKVRQYDQIHIEICGHKVIGTV
LIGPTPVNIIGRSMLTQIGCTLNF
>102782_124 0.3
PQITLWQRPIVTIKVEGQLIEAPLDTGADDTVFEDVNLPGRWKPKMIGGLGGFIKVRQYDQIHIEICGHKVIGTV
LVGPTPVNIIGRNMLTQIGCTLNF
>102782_125 0.3
PQITLWQRPIVTIKVEGQLIEAPLDTGADDTVFEDVNLPGRWKPKMIGGLGGFIKVRQYDQIHIEICGHKVIGTV
LVGPTPVNIIGRSMLTQIGCTLNF
>102782_126 0.3
PQITLWQRPIVTIKVEGQLIEAPLDTGADDTVFEDVNLPGRWKPKMIGGLGGFIKVRQYDQIHIEICGHKVIGTV
LIGPTPVNIIGRNMLTQIGCTLNF
>102782_127 0.3
PQITLWQRPIVTIKVEGQLIEAPLDTGADDTVFEDVNLPGRWKPKMIGGLGGFIKVRQYDQIHIEICGHKVIGTV
LIGPTPVNIIGRSMLTQIGCTLNF
>102782_42 0.3
PQITLWQRPIVTIKVEGQLKEAPLDTGADDTVLEDVNLPGRWKPKMIGGLGGFIKVRQYDQIHIEICGHKAIGT
VLIGPTPVNIIGRNMLTQIGCTLNF
>102782_43 0.3
PQITLWQRPIVTIKVEGQLKEAPLDTGADDTVLEDVNLPGRWKPKMIGGLGGFIKVRQYDQIHIEICGHKAIGT
VLIGPTPVNIIGRSMLTQIGCTLNF
>102782_48 0.3
PQITLWQRPIVTIKVEGQLKEAPLDTGADDTVFEDMNLPGRWKPKMIGGLGGFIKVRQYDQIHIEICGHKAIGT
VLVGPTPVNIIGRNMLTQIGCTLNF
>102782_49 0.3
PQITLWQRPIVTIKVEGQLKEAPLDTGADDTVFEDMNLPGRWKPKMIGGLGGFIKVRQYDQIHIEICGHKAIGT
VLVGPTPVNIIGRSMLTQIGCTLNF
>102782_50 0.3
PQITLWQRPIVTIKVEGQLKEAPLDTGADDTVFEDMNLPGRWKPKMIGGLGGFIKVRQYDQIHIEICGHKAIGT
VLIGPTPVNIIGRNMLTQIGCTLNF
>102782_51 0.3
PQITLWQRPIVTIKVEGQLKEAPLDTGADDTVFEDMNLPGRWKPKMIGGLGGFIKVRQYDQIHIEICGHKAIGT
VLIGPTPVNIIGRSMLTQIGCTLNF
>102782_56 0.3
PQITLWQRPIVTIKVEGQLKEAPLDTGADDTVFEDVNLPGRWKPKMIGGLGGFIKVRQYDQIHIEICGHKAIGT

VLVGPTPVNIIGRNMLTQIGCTLNF
>102782_57 0.3
PQITLWQRPIVTIKVEGQLKEAPLDTGADDTVFEDVNLPGRWKPKMIGGLGGFIKVRQYDQIHIEICGHKAIGT
VLVGPTPVNIIGRSMLTQIGCTLNF
>102782_58 0.3
PQITLWQRPIVTIKVEGQLKEAPLDTGADDTVFEDVNLPGRWKPKMIGGLGGFIKVRQYDQIHIEICGHKAIGT
VLIGPTPVNIIGRNMLTQIGCTLNF
>102782_59 0.3
PQITLWQRPIVTIKVEGQLKEAPLDTGADDTVFEDVNLPGRWKPKMIGGLGGFIKVRQYDQIHIEICGHKAIGT
VLIGPTPVNIIGRSMLTQIGCTLNF
>102782_160 0.3
PQITLWQRPIVTIKVEGQLTEAPLDTGADDTVLEDMNLPGRWKPKMIGGLGGFIKVRQYDQIHIEICGHKAIGT
VLVGPTPVNIIGRNMLTQIGCTLNF
>102782_161 0.3
PQITLWQRPIVTIKVEGQLTEAPLDTGADDTVLEDMNLPGRWKPKMIGGLGGFIKVRQYDQIHIEICGHKAIGT
VLVGPTPVNIIGRSMLTQIGCTLNF
>102782_162 0.3
PQITLWQRPIVTIKVEGQLTEAPLDTGADDTVLEDMNLPGRWKPKMIGGLGGFIKVRQYDQIHIEICGHKAIGT
VLIGPTPVNIIGRNMLTQIGCTLNF
>102782_169 0.3
PQITLWQRPIVTIKVEGQLTEAPLDTGADDTVLEDVNLPGRWKPKMIGGLGGFIKVRQYDQIHIEICGHKAIGT
VLVGPTPVNIIGRSMLTQIGCTLNF
>102782_171 0.3
PQITLWQRPIVTIKVEGQLTEAPLDTGADDTVLEDVNLPGRWKPKMIGGLGGFIKVRQYDQIHIEICGHKAIGT
VLIGPTPVNIIGRSMLTQIGCTLNF
>102782_176 0.3
PQITLWQRPIVTIKVEGQLTEAPLDTGADDTVFEDMNLPGRWKPKMIGGLGGFIKVRQYDQIHIEICGHKAIGT
VLVGPTPVNIIGRNMLTQIGCTLNF
>102782_47 0.3
PQITLWQRPIVTIKVEGQLKEAPLDTGADDTVLEDVNLPGRWKPKMIGGLGGFIKVRQYDQIHIEICGHKVIGT
VLIGPTPVNIIGRSMLTQIGCTLNF
>102782_52 0.3
PQITLWQRPIVTIKVEGQLKEAPLDTGADDTVFEDMNLPGRWKPKMIGGLGGFIKVRQYDQIHIEICGHKVIGT
VLVGPTPVNIIGRNMLTQIGCTLNF
>102782_53 0.3
PQITLWQRPIVTIKVEGQLKEAPLDTGADDTVFEDMNLPGRWKPKMIGGLGGFIKVRQYDQIHIEICGHKVIGT
VLVGPTPVNIIGRSMLTQIGCTLNF
>102782_54 0.3
PQITLWQRPIVTIKVEGQLKEAPLDTGADDTVFEDMNLPGRWKPKMIGGLGGFIKVRQYDQIHIEICGHKVIGT
VLIGPTPVNIIGRNMLTQIGCTLNF
>102782_55 0.3
PQITLWQRPIVTIKVEGQLKEAPLDTGADDTVFEDMNLPGRWKPKMIGGLGGFIKVRQYDQIHIEICGHKVIGT
VLIGPTPVNIIGRSMLTQIGCTLNF
>102782_60 0.3
PQITLWQRPIVTIKVEGQLKEAPLDTGADDTVFEDVNLPGRWKPKMIGGLGGFIKVRQYDQIHIEICGHKVIGT
VLVGPTPVNIIGRNMLTQIGCTLNF
>102782_61 0.3
PQITLWQRPIVTIKVEGQLKEAPLDTGADDTVFEDVNLPGRWKPKMIGGLGGFIKVRQYDQIHIEICGHKVIGT
VLVGPTPVNIIGRSMLTQIGCTLNF
>102782_62 0.3
PQITLWQRPIVTIKVEGQLKEAPLDTGADDTVFEDVNLPGRWKPKMIGGLGGFIKVRQYDQIHIEICGHKVIGT
VLIGPTPVNIIGRNMLTQIGCTLNF
>102782_63 0.3
PQITLWQRPIVTIKVEGQLKEAPLDTGADDTVFEDVNLPGRWKPKMIGGLGGFIKVRQYDQIHIEICGHKVIGT
VLIGPTPVNIIGRSMLTQIGCTLNF
>102782_164 0.3
PQITLWQRPIVTIKVEGQLTEAPLDTGADDTVLEDMNLPGRWKPKMIGGLGGFIKVRQYDQIHIEICGHKVIGT
VLVGPTPVNIIGRNMLTQIGCTLNF
>102782_165 0.3
PQITLWQRPIVTIKVEGQLTEAPLDTGADDTVLEDMNLPGRWKPKMIGGLGGFIKVRQYDQIHIEICGHKVIGT
VLVGPTPVNIIGRSMLTQIGCTLNF
>102782_166 0.3
PQITLWQRPIVTIKVEGQLTEAPLDTGADDTVLEDMNLPGRWKPKMIGGLGGFIKVRQYDQIHIEICGHKVIGT

VLIGPTPVNIIGRNMLTQIGCTLNF
>102782_167 0.3
PQITLWQRPIVTIKVEGQLTEAPLDTGADDTVLEDMNLPGRWKPKMIGGLGGFIKVRQYDQIHIEICGHKVIGT
VLIGPTPVNIIGRSMLTQIGCTLNF
>102782_172 0.3
PQITLWQRPIVTIKVEGQLTEAPLDTGADDTVLEDVNLPGRWKPKMIGGLGGFIKVRQYDQIHIEICGHKVIGT
VLVGPTPVNIIGRNMLTQIGCTLNF
>102782_173 0.3
PQITLWQRPIVTIKVEGQLTEAPLDTGADDTVLEDVNLPGRWKPKMIGGLGGFIKVRQYDQIHIEICGHKVIGT
VLVGPTPVNIIGRSMLTQIGCTLNF
>102782_174 0.3
PQITLWQRPIVTIKVEGQLTEAPLDTGADDTVLEDVNLPGRWKPKMIGGLGGFIKVRQYDQIHIEICGHKVIGT
VLIGPTPVNIIGRNMLTQIGCTLNF
>102782_175 0.3
PQITLWQRPIVTIKVEGQLTEAPLDTGADDTVLEDVNLPGRWKPKMIGGLGGFIKVRQYDQIHIEICGHKVIGT
VLIGPTPVNIIGRSMLTQIGCTLNF
>102782_180 0.3
PQITLWQRPIVTIKVEGQLTEAPLDTGADDTVFEDMNLPGRWKPKMIGGLGGFIKVRQYDQIHIEICGHKVIGT
VLVGPTPVNIIGRNMLTQIGCTLNF
>102782_181 0.3
PQITLWQRPIVTIKVEGQLTEAPLDTGADDTVFEDMNLPGRWKPKMIGGLGGFIKVRQYDQIHIEICGHKVIGT
VLVGPTPVNIIGRSMLTQIGCTLNF
>102782_182 0.3
PQITLWQRPIVTIKVEGQLTEAPLDTGADDTVFEDMNLPGRWKPKMIGGLGGFIKVRQYDQIHIEICGHKVIGT
VLIGPTPVNIIGRNMLTQIGCTLNF
>102782_183 0.3
PQITLWQRPIVTIKVEGQLTEAPLDTGADDTVFEDMNLPGRWKPKMIGGLGGFIKVRQYDQIHIEICGHKVIGT
VLIGPTPVNIIGRSMLTQIGCTLNF
>102782_188 0.3
PQITLWQRPIVTIKVEGQLTEAPLDTGADDTVFEDVNLPGRWKPKMIGGLGGFIKVRQYDQIHIEICGHKVIGT
VLVGPTPVNIIGRNMLTQIGCTLNF
>102782_189 0.3
PQITLWQRPIVTIKVEGQLTEAPLDTGADDTVFEDVNLPGRWKPKMIGGLGGFIKVRQYDQIHIEICGHKVIGT
VLVGPTPVNIIGRSMLTQIGCTLNF
>102782_190 0.3
PQITLWQRPIVTIKVEGQLTEAPLDTGADDTVFEDVNLPGRWKPKMIGGLGGFIKVRQYDQIHIEICGHKVIGT
VLIGPTPVNIIGRNMLTQIGCTLNF
>102782_191 0.3
PQITLWQRPIVTIKVEGQLTEAPLDTGADDTVFEDVNLPGRWKPKMIGGLGGFIKVRQYDQIHIEICGHKVIGT
VLIGPTPVNIIGRSMLTQIGCTLNF
>102782_76 0.3
PQITLWQRPIVTIKVEGQLIEALLDTGADDTVLEDVNLPGRWKPKMIGGLGGFIKVRQYDQIHIEICGHKVIGTV
LVGPTPVNIIGRNMLTQIGCTLNF
>102782_78 0.3
PQITLWQRPIVTIKVEGQLIEALLDTGADDTVLEDVNLPGRWKPKMIGGLGGFIKVRQYDQIHIEICGHKVIGTV
LIGPTPVNIIGRNMLTQIGCTLNF
>102782_79 0.3
PQITLWQRPIVTIKVEGQLIEALLDTGADDTVLEDVNLPGRWKPKMIGGLGGFIKVRQYDQIHIEICGHKVIGTV
LIGPTPVNIIGRSMLTQIGCTLNF
>102782_84 0.3
PQITLWQRPIVTIKVEGQLIEALLDTGADDTVFEDMNLPGRWKPKMIGGLGGFIKVRQYDQIHIEICGHKVIGTV
LVGPTPVNIIGRNMLTQIGCTLNF
>102782_85 0.3
PQITLWQRPIVTIKVEGQLIEALLDTGADDTVFEDMNLPGRWKPKMIGGLGGFIKVRQYDQIHIEICGHKVIGTV
LVGPTPVNIIGRSMLTQIGCTLNF
>102782_86 0.3
PQITLWQRPIVTIKVEGQLIEALLDTGADDTVFEDMNLPGRWKPKMIGGLGGFIKVRQYDQIHIEICGHKVIGTV
LIGPTPVNIIGRNMLTQIGCTLNF
>102782_87 0.3
PQITLWQRPIVTIKVEGQLIEALLDTGADDTVFEDMNLPGRWKPKMIGGLGGFIKVRQYDQIHIEICGHKVIGTV
LIGPTPVNIIGRSMLTQIGCTLNF
>102782_92 0.3
PQITLWQRPIVTIKVEGQLIEALLDTGADDTVFEDVNLPGRWKPKMIGGLGGFIKVRQYDQIHIEICGHKVIGTV

LVGPTPVNIIGRNMLTQIGCTLNF
>102782_93 0.3
PQITLWQRPIVTIKVEGQLIEALLDTGADDTVFEDVNLPGRWKPKMIGGLGGFIKVRQYDQIHIEICGHKVIGTV
LVGPTPVNIIGRSMLTQIGCTLNF
>102782_94 0.3
PQITLWQRPIVTIKVEGQLIEALLDTGADDTVFEDVNLPGRWKPKMIGGLGGFIKVRQYDQIHIEICGHKVIGTV
LIGPTPVNIIGRNMLTQIGCTLNF
>102782_95 0.3
PQITLWQRPIVTIKVEGQLIEALLDTGADDTVFEDVNLPGRWKPKMIGGLGGFIKVRQYDQIHIEICGHKVIGTV
LIGPTPVNIIGRSMLTQIGCTLNF
>102793_0 0.2
PQITLWQRPLVTIKIGGQLKEALLDTGADDTVFEDIDLPGRWKPIMIGGLGGFIKVRQYDQITIEICGHKAIGTVL
VGPTPVNIIGRNLLTQIGCTLNF
>102782_19 0.3
PQITLWQRPIVTIKVEGQLKEALLDTGADDTVFEDMNLPGRWKPKMIGGLGGFIKVRQYDQIHIEICGHKAIGT
VLIGPTPVNIIGRSMLTQIGCTLNF
>102782_24 0.3
PQITLWQRPIVTIKVEGQLKEALLDTGADDTVFEDVNLPGRWKPKMIGGLGGFIKVRQYDQIHIEICGHKAIGT
VLVGPTPVNIIGRNMLTQIGCTLNF
>102782_25 0.3
PQITLWQRPIVTIKVEGQLKEALLDTGADDTVFEDVNLPGRWKPKMIGGLGGFIKVRQYDQIHIEICGHKAIGT
VLVGPTPVNIIGRSMLTQIGCTLNF
>102782_26 0.3
PQITLWQRPIVTIKVEGQLKEALLDTGADDTVFEDVNLPGRWKPKMIGGLGGFIKVRQYDQIHIEICGHKAIGT
VLIGPTPVNIIGRNMLTQIGCTLNF
>102782_27 0.3
PQITLWQRPIVTIKVEGQLKEALLDTGADDTVFEDVNLPGRWKPKMIGGLGGFIKVRQYDQIHIEICGHKAIGT
VLIGPTPVNIIGRSMLTQIGCTLNF
>102782_128 0.3
PQITLWQRPIVTIKVEGQLTEALLDTGADDTVLEDMNLPGRWKPKMIGGLGGFIKVRQYDQIHIEICGHKAIGT
VLVGPTPVNIIGRNMLTQIGCTLNF
>102782_129 0.3
PQITLWQRPIVTIKVEGQLTEALLDTGADDTVLEDMNLPGRWKPKMIGGLGGFIKVRQYDQIHIEICGHKAIGT
VLVGPTPVNIIGRSMLTQIGCTLNF
>102782_130 0.3
PQITLWQRPIVTIKVEGQLTEALLDTGADDTVLEDMNLPGRWKPKMIGGLGGFIKVRQYDQIHIEICGHKAIGT
VLIGPTPVNIIGRNMLTQIGCTLNF
>102782_131 0.3
PQITLWQRPIVTIKVEGQLTEALLDTGADDTVLEDMNLPGRWKPKMIGGLGGFIKVRQYDQIHIEICGHKAIGT
VLIGPTPVNIIGRSMLTQIGCTLNF
>102782_136 0.3
PQITLWQRPIVTIKVEGQLTEALLDTGADDTVLEDVNLPGRWKPKMIGGLGGFIKVRQYDQIHIEICGHKAIGTV
LVGPTPVNIIGRNMLTQIGCTLNF
>102782_137 0.3
PQITLWQRPIVTIKVEGQLTEALLDTGADDTVLEDVNLPGRWKPKMIGGLGGFIKVRQYDQIHIEICGHKAIGTV
LVGPTPVNIIGRSMLTQIGCTLNF
>102782_138 0.3
PQITLWQRPIVTIKVEGQLTEALLDTGADDTVLEDVNLPGRWKPKMIGGLGGFIKVRQYDQIHIEICGHKAIGTV
LIGPTPVNIIGRNMLTQIGCTLNF
>102782_139 0.3
PQITLWQRPIVTIKVEGQLTEALLDTGADDTVLEDVNLPGRWKPKMIGGLGGFIKVRQYDQIHIEICGHKAIGTV
LIGPTPVNIIGRSMLTQIGCTLNF
>102782_144 0.3
PQITLWQRPIVTIKVEGQLTEALLDTGADDTVFEDMNLPGRWKPKMIGGLGGFIKVRQYDQIHIEICGHKAIGT
VLVGPTPVNIIGRNMLTQIGCTLNF
>102782_145 0.3
PQITLWQRPIVTIKVEGQLTEALLDTGADDTVFEDMNLPGRWKPKMIGGLGGFIKVRQYDQIHIEICGHKAIGT
VLVGPTPVNIIGRSMLTQIGCTLNF
>102782_146 0.3
PQITLWQRPIVTIKVEGQLTEALLDTGADDTVFEDMNLPGRWKPKMIGGLGGFIKVRQYDQIHIEICGHKAIGT
VLIGPTPVNIIGRNMLTQIGCTLNF
>102782_147 0.3
PQITLWQRPIVTIKVEGQLTEALLDTGADDTVFEDMNLPGRWKPKMIGGLGGFIKVRQYDQIHIEICGHKAIGT

VLIGPTPVNIIGRSMLTQIGCTLNF
>102782_152 0.3
PQITLWQRPIVTIKVEGQLTEALLDTGADDTVFEDVNLPGRWKPKMIGGLGGFIKVRQYDQIHIEICGHKAIGTV
LVGPTPVNIIGRNMLTQIGCTLNF
>102782_153 0.3
PQITLWQRPIVTIKVEGQLTEALLDTGADDTVFEDVNLPGRWKPKMIGGLGGFIKVRQYDQIHIEICGHKAIGTV
LVGPTPVNIIGRSMLTQIGCTLNF
>102782_154 0.3
PQITLWQRPIVTIKVEGQLTEALLDTGADDTVFEDVNLPGRWKPKMIGGLGGFIKVRQYDQIHIEICGHKAIGTV
LIGPTPVNIIGRNMLTQIGCTLNF
>102782_155 0.3
PQITLWQRPIVTIKVEGQLTEALLDTGADDTVFEDVNLPGRWKPKMIGGLGGFIKVRQYDQIHIEICGHKAIGTV
LIGPTPVNIIGRSMLTQIGCTLNF
>102782_7 0.3
PQITLWQRPIVTIKVEGQLKEALLDTGADDTVLEDMNLPGRWKPKMIGGLGGFIKVRQYDQIHIEICGHKVIGT
VLIGPTPVNIIGRSMLTQIGCTLNF
>102782_158 0.3
PQITLWQRPIVTIKVEGQLTEALLDTGADDTVFEDVNLPGRWKPKMIGGLGGFIKVRQYDQIHIEICGHKVIGTV
LIGPTPVNIIGRNMLTQIGCTLNF
>102782_159 0.3
PQITLWQRPIVTIKVEGQLTEALLDTGADDTVFEDVNLPGRWKPKMIGGLGGFIKVRQYDQIHIEICGHKVIGTV
LIGPTPVNIIGRSMLTQIGCTLNF
>257929_0 0.3
PQITLWQRPLVTVKVGGQLKEALLDTGADDTVLEEMNLQGRWKPKMIGGIGGFIKVKQYDQIPVEICGHKAIG
TVLVGPTPVNIIGRNLLTQIGCTLNF

Sequences for the LPV-resistant ensemble
>187119_0 500.0
PQITLWQRPIIPVRIGGQLKEALLDTGADDTIFEEMTLPGRWQPKIVGGIGGFLKVRQYDQIPIEFCGYKVMCT
VLIGSTPINVIGRDTLTKIGCTLNF
>154816_0 500.0
PQITLWQRPFITVKIGGQQMEALLDTGADDTIVEAINLPGRWKPKIVGGIGGFMKVKQYDQVPVEICGHKAITA
VLVGPTPVNVIGRNVMTQIGCTLNF
>117033_2 500.0
PQITLWQRPIVTIKIGGQIREALLDTGADDTVLEDINLPGRWKPKLIVGIGGFTKVKEYDQVPIEICGHKVVGTVL
VGPTPANIIGRNLLTQLGCTLNF
>117033_4 500.0
PQITLWQRPIVTIKIGGQIREALLDTGADDTVLEDINLPGRWEPKLIVGIGGFSKVKEYDQVPIEICGHKVVGTVL
VGPTPANIIGRNLLTQLGCTLNF
>117033_5 500.0
PQITLWQRPIVTIKIGGQIREALLDTGADDTVLEDINLPGRWEPKLIVGIGGFTKVKEYDQVPIEICGHKVVGTVL
VGPTPANIIGRNLLTQLGCTLNF
>147553_0 500.0
PQITLWQRPIVTIKIGGQLREALLDTGADDTVFTDIDLPGRWTPKIIVGVGGFSKVKQYDQVPIEICGHKVVGTV
LIGPTPANIVGRNLLTQLGCTLNF
>81925_0 500.0
PQITLWQRPFVTVKIAGQIKEALLDTGADDTVFQEMDLPGRWKPKLIGGIGGFVKVRQYDQVLIEFCGHKTTC
TVLVGPTPVNVIGRNLMSQLGCTLNF
>81925_2 500.0
PQITLWQRPFVTVKIAGQVKEALLDTGADDTVFQEMDLPGRWKPKLIGGIGGFVKVRQYDQVLIEFCGHKTT
CTVLVGPTPVNVIGRNLMSQLGCTLNF
>50921_0 500.0
PQITLWQRPIVTIKIAGQQKEALLDTGADDTILEEMSLPGKWKPRIAGGIGGFIKVRQYDQIPIEICGHKVVGTVL
VGPTPANIIGRNLMTKLGCTLNF
>117063_1 500.0
PQITLWQRPVVTVKVAGQLREALLDTGADDTIFEEINLPGRWTPKIVGGIGGFMKVRQYDQIPIEICGHKVISTV
LVGPTPTNIIGGNLMTQIGCTLNF
>103779_0 500.0
PQITLWQRPIIKVKIGGQLVEALLDTGADDTILEGINLPGRWKPKIVGGIGGFMKVKEYDQIPVEICGHKIISTVLV
GPTPVNIIGRNVMTQIGCTLNF
>103779_1 500.0
PQITLWQRPIIKVKIGGQLVEALLDTGADDTILEGINLPGRWKPKIVGGIGGFMKVKEYDQIPVEICGHKIISTVLV
GPTPVNVIGRNVMTQIGCTLNF

>103779_2 500.0
PQITLWQRPIIKVKIGGQLVEALLDTGADDTILEGINLPGRWKPKIVGGIGGFMKVKEYDQIPVEICGHKVISTVL
VGPTPVNIIGRNVMTQIGCTLNF
>103779_3 500.0
PQITLWQRPIIKVKIGGQLVEALLDTGADDTILEGINLPGRWKPKIVGGIGGFMKVKEYDQIPVEICGHKVISTVL
VGPTPVNVIGRNVMTQIGCTLNF
>103779_4 500.0
PQITLWQRPIIKVKIGGQLVEALLDTGADDTILEGIDLPGRWKPKIVGGIGGFMKVKEYDQIPVEICGHKIISTVLV
GPTPVNIIGRNVMTQIGCTLNF
>103779_5 500.0
PQITLWQRPIIKVKIGGQLVEALLDTGADDTILEGIDLPGRWKPKIVGGIGGFMKVKEYDQIPVEICGHKIISTVLV
GPTPVNVIGRNVMTQIGCTLNF
>103779_6 500.0
PQITLWQRPIIKVKIGGQLVEALLDTGADDTILEGIDLPGRWKPKIVGGIGGFMKVKEYDQIPVEICGHKVISTVL
VGPTPVNIIGRNVMTQIGCTLNF
>103779_7 500.0
PQITLWQRPIIKVKIGGQLVEALLDTGADDTILEGIDLPGRWKPKIVGGIGGFMKVKEYDQIPVEICGHKVISTVL
VGPTPVNVIGRNVMTQIGCTLNF
>103779_8 500.0
PQITLWQRPIIKVKIGGQLVEALLDTGADDTIFEGINLPGRWKPKIVGGIGGFMKVKEYDQIPVEICGHKIISTVL
VGPTPVNIIGRNVMTQIGCTLNF
>103779_9 500.0
PQITLWQRPIIKVKIGGQLVEALLDTGADDTIFEGINLPGRWKPKIVGGIGGFMKVKEYDQIPVEICGHKIISTVL
VGPTPVNVIGRNVMTQIGCTLNF
>103779_10 500.0
PQITLWQRPIIKVKIGGQLVEALLDTGADDTIFEGINLPGRWKPKIVGGIGGFMKVKEYDQIPVEICGHKVISTVL
VGPTPVNIIGRNVMTQIGCTLNF
>103779_11 500.0
PQITLWQRPIIKVKIGGQLVEALLDTGADDTIFEGINLPGRWKPKIVGGIGGFMKVKEYDQIPVEICGHKVISTVL
VGPTPVNVIGRNVMTQIGCTLNF
>103779_12 500.0
PQITLWQRPIIKVKIGGQLVEALLDTGADDTIFEGIDLPGRWKPKIVGGIGGFMKVKEYDQIPVEICGHKIISTVL
VGPTPVNIIGRNVMTQIGCTLNF
>103779_13 500.0
PQITLWQRPIIKVKIGGQLVEALLDTGADDTIFEGIDLPGRWKPKIVGGIGGFMKVKEYDQIPVEICGHKIISTVL
VGPTPVNVIGRNVMTQIGCTLNF
>103779_14 500.0
PQITLWQRPIIKVKIGGQLVEALLDTGADDTIFEGIDLPGRWKPKIVGGIGGFMKVKEYDQIPVEICGHKVISTVL
VGPTPVNIIGRNVMTQIGCTLNF
>103779_15 500.0
PQITLWQRPIIKVKIGGQLVEALLDTGADDTIFEGIDLPGRWKPKIVGGIGGFMKVKEYDQIPVEICGHKVISTVL
VGPTPVNVIGRNVMTQIGCTLNF
>147550_0 500.0
PQITLWQRPIIKVKIGGQLVEALLDTGADDTIFEGIDLPGRWKPKIVGGIGGFMKVKEYDQIPVEVCGHKVISTV
LVGPTPVNVIGRNVMTQIGCTLNF
>103057_2 500.0
PQITLWQRPIVTVKIGGQLREALLDTGADDTVFEDIELPGRWTPKIIGGIGGFVKVKQYEEVPVEICEHKTTATV
LVGPTPFNIIGRNLMTQLGCTLNF
>117027_0 500.0
PQITLWQRPFVTVKIGGQPKEALLDTGADNTVFEDINLPGRWTPKIIGGIGGFVRVKQYDQVPIEICGHKTIGTI
LVGPTPANIIGRDVLTKIGCTLNF
>117027_1 500.0
PQITLWQRPFVTVKIGGQPKEALLDTGADNTVFEDINLPGRWTPKIIGGIGGFVRVKQYDQVPIEICGHKTMGT
ILVGPTPANIIGRDVLTKIGCTLNF
>143473_1 500.0
PQITLWQRPIVTVKIGGQLREALLDTGADDTVFEDIDLPGRWTPKLIGGIGGFVKVRQYDQVPIEICGHKITSTV
LVGPTPANIIGRNLMTQLGCTLNF
>143473_3 500.0
PQITLWQRPIVTVKIGGQLREALLDTGADDTVFEDIDLPGRWTPKLIGGIGGFVKVRQYDQVPIEICGHKVTST
VLVGPTPANIIGRNLMTQLGCTLNF
>143473_5 500.0
PQITLWQRPIVTVKIGGQLREALLDTGADDTVFEDIDLPGRWTPKLIGGIGGFVRVRQYDQVPIEICGHKITSTV
LVGPTPANIIGRNLMTQLGCTLNF

>143473_7 500.0
PQITLWQRPIVTVKIGGQLREALLDTGADDTVFEDIDLPGRWTPKLIGGIGGFVRVRQYDQVPIEICGHKVTST
VLVGPTPANIIGRNLMTQLGCTLNF
>143473_9 500.0
PQITLWQRPIVTVKIGGQIREALLDTGADDTVFEDIDLPGRWTPKLIGGIGGFVKVRQYDQVPIEICGHKITSTV
LVGPTPANIIGRNLMTQLGCTLNF
>143473_11 500.0
PQITLWQRPIVTVKIGGQIREALLDTGADDTVFEDIDLPGRWTPKLIGGIGGFVKVRQYDQVPIEICGHKVTSTV
LVGPTPANIIGRNLMTQLGCTLNF
>143473_13 500.0
PQITLWQRPIVTVKIGGQIREALLDTGADDTVFEDIDLPGRWTPKLIGGIGGFVRVRQYDQVPIEICGHKITSTV
LVGPTPANIIGRNLMTQLGCTLNF
>143473_15 500.0
PQITLWQRPIVTVKIGGQIREALLDTGADDTVFEDIDLPGRWTPKLIGGIGGFVRVRQYDQVPIEICGHKVTSTV
LVGPTPANIIGRNLMTQLGCTLNF
>115081_0 500.0
PQITLWQRPIVTIKIGGQLREALLDTGADDTVLEDIDLPGRWKPKLIVGIGGFVKVRQYEQVPIEICGHKVVGTV
LIGPTPSNIIGRNLMTQLGCTLNF
>86742_0 500.0
PQITLWQRPFITIKIGGQLREALLDTGADDTIFEDIQLPGRWKPKIVGGIGGFLKVKEYEQVPIEICGHKVISTVL
TGPTPVNVIGRNVMTQLGCTLNF
>86742_1 500.0
PQITLWQRPFITVKIGGQLREALLDTGADDTIFEDIQLPGRWKPKIVGGIGGFLKVKEYEQVPIEICGHKVISTVL
TGPTPVNVIGRNVMTQLGCTLNF
>82251_0 500.0
PQITLWQRPIVTVKIGGQLKEALLDTGADDTIFEEINLPGRWKPKMAGGIGGFIKVRQYDQIPIEICGHEVIGTVL
VGPTPANIIGRNLMTQLGCTLNF
>82251_1 500.0
PQITLWQRPIVTVKIGGQLKEALLDTGADDTIFEEINLPGRWKPKIAGGIGGFIKVRQYDQIPIEICGHEVIGTVL
VGPTPANIIGRNLMTQLGCTLNF
>82251_3 500.0
PQITLWQRPIVTVKIGGQLKEALLDTGADDTIFEEINLPGKWKPKMAGGIGGFIKVRQYDQIPIEICGHEVIGTVL
VGPTPANIIGRNLMTQLGCTLNF
>82251_4 500.0
PQITLWQRPIVTVKIGGQLKEALLDTGADDTIFEEINLPGKWKPKIAGGIGGFIKVRQYDQIPIEICGHEVIGTVLV
GPTPANIIGRNLMTQLGCTLNF
>82251_5 500.0
PQITLWQRPIVTVKIGGQLKEALLDTGADDTIFEEINLPGKWKPKLAGGIGGFIKVRQYDQIPIEICGHEVIGTVL
VGPTPANIIGRNLMTQLGCTLNF
>116999_0 500.0
PQITLWQRPVVTIKVGGQIKEALLDTGADDTILEEMNLPGRWKPKIAGGIGGFIKVRQYDQIPIEICGHKVIGTV
LIGPTPANIIGRNLLTQLGCTLNF
>116999_1 500.0
PQITLWQRPVVTIKVGGQIKEALLDTGADDTILEEMNLPGRWKPKIAGGIGGFIKVRQYDQIPIEICGHEVIGTV
LIGPTPANIIGRNLLTQLGCTLNF
>116999_2 500.0
PQITLWQRPVVTIKVGGQIKEALLDTGADDTILEEMNLPGRWKPKIAGGIGGFIKVREYDQIPIEICGHKVIGTVL
IGPTPANIIGRNLLTQLGCTLNF
>116999_3 500.0
PQITLWQRPVVTIKVGGQIKEALLDTGADDTILEEMNLPGRWKPKIAGGIGGFIKVREYDQIPIEICGHEVIGTVL
IGPTPANIIGRNLLTQLGCTLNF
>116999_4 500.0
PQITLWQRPVVTIKVGGQIKEALLDTGADDTILEEMNLPGRWKPKIAGGIGGFINVRQYDQIPIEICGHKVIGTV
LIGPTPANIIGRNLLTQLGCTLNF
>116999_5 500.0
PQITLWQRPVVTIKVGGQIKEALLDTGADDTILEEMNLPGRWKPKIAGGIGGFINVRQYDQIPIEICGHEVIGTV
LIGPTPANIIGRNLLTQLGCTLNF
>116999_6 500.0
PQITLWQRPVVTIKVGGQIKEALLDTGADDTILEEMNLPGRWKPKIAGGIGGFINVREYDQIPIEICGHKVIGTVL
IGPTPANIIGRNLLTQLGCTLNF
>116999_7 500.0
PQITLWQRPVVTIKVGGQIKEALLDTGADDTILEEMNLPGRWKPKIAGGIGGFINVREYDQIPIEICGHEVIGTVL
IGPTPANIIGRNLLTQLGCTLNF

>116999_8 500.0
PQITLWQRPVVTIKVGGQIKEALLDTGADDTIFEEMNLPGRWKPKIAGGIGGFIKVRQYDQIPIEICGHKVIGTV
LIGPTPANIIGRNLLTQLGCTLNF
>116999_9 500.0
PQITLWQRPVVTIKVGGQIKEALLDTGADDTIFEEMNLPGRWKPKIAGGIGGFIKVRQYDQIPIEICGHEVIGTV
LIGPTPANIIGRNLLTQLGCTLNF
>116999_10 500.0
PQITLWQRPVVTIKVGGQIKEALLDTGADDTIFEEMNLPGRWKPKIAGGIGGFIKVREYDQIPIEICGHKVIGTVL
IGPTPANIIGRNLLTQLGCTLNF
>116999_11 500.0
PQITLWQRPVVTIKVGGQIKEALLDTGADDTIFEEMNLPGRWKPKIAGGIGGFIKVREYDQIPIEICGHEVIGTVL
IGPTPANIIGRNLLTQLGCTLNF
>116999_12 500.0
PQITLWQRPVVTIKVGGQIKEALLDTGADDTIFEEMNLPGRWKPKIAGGIGGFINVRQYDQIPIEICGHKVIGTV
LIGPTPANIIGRNLLTQLGCTLNF
>116999_13 500.0
PQITLWQRPVVTIKVGGQIKEALLDTGADDTIFEEMNLPGRWKPKIAGGIGGFINVRQYDQIPIEICGHEVIGTV
LIGPTPANIIGRNLLTQLGCTLNF
>116999_14 500.0
PQITLWQRPVVTIKVGGQIKEALLDTGADDTIFEEMNLPGRWKPKIAGGIGGFINVREYDQIPIEICGHKVIGTV
LIGPTPANIIGRNLLTQLGCTLNF
>116999_15 500.0
PQITLWQRPVVTIKVGGQIKEALLDTGADDTIFEEMNLPGRWKPKIAGGIGGFINVREYDQIPIEICGHEVIGTV
LIGPTPANIIGRNLLTQLGCTLNF
>116999_16 500.0
PQITLWQRPVVTVKVGGQIKEALLDTGADDTILEEMNLPGRWKPKIAGGIGGFIKVRQYDQIPIEICGHKVIGTV
LIGPTPANIIGRNLLTQLGCTLNF
>117063_0 500.0
PQITLWQRPVVTVKVAGQLREALLDTGADDTIFEEINLPGRWTPKIVGGIGGFMKVRQYDQIPIEICGHKVISTV
LVGPTPTNIIGRNLMTQIGCTLNF
>115739_2 500.0
PQITLWQRPVVTVKVGGQLREALLDTGADDTIFEDISLPGKWTPKIVGGIGGLIKVKQYDQIPIEICGHKVISTVL
VGPTPANIIGRNLMTQLGCTLNF
>115739_3 500.0
PQITLWQRPVVTVKVGGQLREALLDTGADDTIFEDISLPGKWTPKIVGGIGGLIKVKQYDQIPIEICGHKVISTVL
VGPTPANIIGRNLMTQLGFTLNF
>143473_0 500.0
PQITLWQRPIVTVKIGGQLREALLDTGADDTVFEDIDLPGRWTPKLIGGIGGFVKVRQYDQVPIEICGHKIISTV
LVGPTPANIIGRNLMTQLGCTLNF
>143473_2 500.0
PQITLWQRPIVTVKIGGQLREALLDTGADDTVFEDIDLPGRWTPKLIGGIGGFVKVRQYDQVPIEICGHKVISTV
LVGPTPANIIGRNLMTQLGCTLNF
>143473_4 500.0
PQITLWQRPIVTVKIGGQLREALLDTGADDTVFEDIDLPGRWTPKLIGGIGGFVRVRQYDQVPIEICGHKIISTV
LVGPTPANIIGRNLMTQLGCTLNF
>143473_6 500.0
PQITLWQRPIVTVKIGGQLREALLDTGADDTVFEDIDLPGRWTPKLIGGIGGFVRVRQYDQVPIEICGHKVISTV
LVGPTPANIIGRNLMTQLGCTLNF
>143473_8 500.0
PQITLWQRPIVTVKIGGQIREALLDTGADDTVFEDIDLPGRWTPKLIGGIGGFVKVRQYDQVPIEICGHKIISTVL
VGPTPANIIGRNLMTQLGCTLNF
>143473_10 500.0
PQITLWQRPIVTVKIGGQIREALLDTGADDTVFEDIDLPGRWTPKLIGGIGGFVKVRQYDQVPIEICGHKVISTV
LVGPTPANIIGRNLMTQLGCTLNF
>143473_12 500.0
PQITLWQRPIVTVKIGGQIREALLDTGADDTVFEDIDLPGRWTPKLIGGIGGFVRVRQYDQVPIEICGHKIISTVL
VGPTPANIIGRNLMTQLGCTLNF
>143473_14 500.0
PQITLWQRPIVTVKIGGQIREALLDTGADDTVFEDIDLPGRWTPKLIGGIGGFVRVRQYDQVPIEICGHKVISTV
LVGPTPANIIGRNLMTQLGCTLNF
>103067_0 500.0
PQITLWQRPFITVKIAGQLKEALLDTGADDTVFQEMNLPGRWIPKLIGGIAGFMKVRQYDQIPMEIYGHKVMAT
VLVGPTPVNVIGRNLMTQIGCTLNF

>103067_1 500.0
PQITLWQRPFITVKIAGQVKEALLDTGADDTVFQEMNLPGRWIPKLIGGIAGFMKVRQYDQIPMEIYGHKVMA
TVLVGPTPVNVIGRNLMTQIGCTLNF
>145212_0 500.0
PQITLWQRPIVTVKIAGQLKEALLDTGADDTIFEEMNLPGRWTPKLIGGIGGFLKVRQYDQVPIEICGHKVIGPV
LVGPTPVNVIGRNLMSQIGCTLNF
>107977_0 500.0
PQITLWQRPIVTIKIGGQLREALIDTGADDTVLEDMDLPGRWKPKIIGGIGGFVKVRQYDHVPVEICGHKVVGS
VVVGPTPTNIIGRNLLTQLGCTLNF
>107977_1 500.0
PQITLWQRPIVTIKIGGQLREALIDTGADDTVLEDMDLPGRWKPKIIGGIGGFVKVKQYDHVPVEICGHKVVGS
VVVGPTPTNIIGRNLLTQLGCTLNF
>107977_2 500.0
PQITLWQRPIVTIKIGGQLREALIDTGADDTVLEDMELPGRWKPKIIGGIGGFVKVRQYDHVPVEICGHKVVGS
VVVGPTPTNIIGRNLLTQLGCTLNF
>107977_3 500.0
PQITLWQRPIVTIKIGGQLREALIDTGADDTVLEDMELPGRWKPKIIGGIGGFVKVKQYDHVPVEICGHKVVGS
VVVGPTPTNIIGRNLLTQLGCTLNF
>117069_0 500.0
PQITLWQRPILTVKIAGQLTEALLDTGADDTIFQDINLPGRWTPKIVGGIGGLLRVRQYEQVPIEVCGHKIITTVLI
GPTPVNVLGRNLMTQLGCTLNF
>117069_1 500.0
PQITLWQRPILTVKVAGQLTEALLDTGADDTIFQDINLPGRWTPKIVGGIGGLLRVRQYEQVPIEVCGHKIITTVL
IGPTPVNVLGRNLMTQLGCTLNF
>117053_0 500.0
PQITLWQRPIVTIKIGGQLREALLDTGADDTVLEDINLPGRWKPKLIGGIGGFVKVREYEEIPVEICGHKVIGTVL
VGPTPFNVVGRNLLTQLGCTLNF
>117053_1 500.0
PQITLWQRPIVTIKIGGQLREALLDTGADDTVLEDINLPGRWKPKLIGGIGGFVKVREYEEIPVEVCGHKVIGTV
LVGPTPFNVVGRNLLTQLGCTLNF
>117053_2 500.0
PQITLWQRPIVTIKIGGQLREALLDTGADDTVLEDINLPGRWKPKLIGGIGGFVKVREYEEIPVDICGHKVIGTVL
VGPTPFNVVGRNLLTQLGCTLNF
>117053_3 500.0
PQITLWQRPIVTIKIGGQLREALLDTGADDTVLEDINLPGRWKPKLIGGIGGFVKVREYEEIPVDVCGHKVIGTV
LVGPTPFNVVGRNLLTQLGCTLNF
>117053_4 500.0
PQITLWQRPIVTIKIGGQLREALLDTGADDTVLEDINLPGRWKPKLIGGIGGFVKVREYEEVPVEICGHKVIGTV
LVGPTPFNVVGRNLLTQLGCTLNF
>117053_5 500.0
PQITLWQRPIVTIKIGGQLREALLDTGADDTVLEDINLPGRWKPKLIGGIGGFVKVREYEEVPVEVCGHKVIGT
VLVGPTPFNVVGRNLLTQLGCTLNF
>117053_6 500.0
PQITLWQRPIVTIKIGGQLREALLDTGADDTVLEDINLPGRWKPKLIGGIGGFVKVREYEEVPVDICGHKVIGTV
LVGPTPFNVVGRNLLTQLGCTLNF
>117053_7 500.0
PQITLWQRPIVTIKIGGQLREALLDTGADDTVLEDINLPGRWKPKLIGGIGGFVKVREYEEVPVDVCGHKVIGT
VLVGPTPFNVVGRNLLTQLGCTLNF
>117053_8 500.0
PQITLWQRPIVTIKVGGQLREALLDTGADDTVLEDINLPGRWKPKLIGGIGGFVKVREYEEIPVEICGHKVIGTV
LVGPTPFNVVGRNLLTQLGCTLNF
>117053_9 500.0
PQITLWQRPIVTIKVGGQLREALLDTGADDTVLEDINLPGRWKPKLIGGIGGFVKVREYEEIPVEVCGHKVIGT
VLVGPTPFNVVGRNLLTQLGCTLNF
>117053_10 500.0
PQITLWQRPIVTIKVGGQLREALLDTGADDTVLEDINLPGRWKPKLIGGIGGFVKVREYEEIPVDICGHKVIGTV
LVGPTPFNVVGRNLLTQLGCTLNF
>117053_11 500.0
PQITLWQRPIVTIKVGGQLREALLDTGADDTVLEDINLPGRWKPKLIGGIGGFVKVREYEEIPVDVCGHKVIGT
VLVGPTPFNVVGRNLLTQLGCTLNF
>117053_12 500.0
PQITLWQRPIVTIKVGGQLREALLDTGADDTVLEDINLPGRWKPKLIGGIGGFVKVREYEEVPVEICGHKVIGT
VLVGPTPFNVVGRNLLTQLGCTLNF

>117053_13 500.0
PQITLWQRPIVTIKVGGQLREALLDTGADDTVLEDINLPGRWKPKLIGGIGGFVKVREYEEVPVEVCGHKVIGT
VLVGPTPFNVVGRNLLTQLGCTLNF
>117053_14 500.0
PQITLWQRPIVTIKVGGQLREALLDTGADDTVLEDINLPGRWKPKLIGGIGGFVKVREYEEVPVDICGHKVIGT
VLVGPTPFNVVGRNLLTQLGCTLNF
>117053_15 500.0
PQITLWQRPIVTIKVGGQLREALLDTGADDTVLEDINLPGRWKPKLIGGIGGFVKVREYEEVPVDVCGHKVIGT
VLVGPTPFNVVGRNLLTQLGCTLNF

Sequences for the LPV-susceptible ensemble
>81851_0 0.3
PQITLWQRPLVPIRIEGQLKEALLDTGADDTVIEEMSLSGRWKPKMIGGIGGFIKVKQYEQIPIEICGHKAIGTVL
VGPTPVNIIGRNLLTQLGCTLNF
>102783_3 0.3
PQITLWQRPITTIKIGGQLKEALLDTGADDTVLEDMNLPGRWKPKMIGGIGGFIKVRQYDEVPLEICGHKVTGT
VLIGSTPVNIIGRNLLTQLGCTLNF
>102783_2 0.3
PQITLWQRPITTIKIGGQLKEALLDTGADDTVLEDMNLPGRWKPKMIGGIGGFIKVRQYDEVPLEICGHKVTGT
VLIGATPVNIIGRNLLTQLGCTLNF
>102782_36 0.3
PQITLWQRPIVTIKVEGQLKEAPLDTGADDTVLEDMNLPGRWKPKMIGGLGGFIKVRQYDQIHIEICGHKVIGT
VLVGPTPVNIIGRNMLTQIGCTLNF
>102782_173 0.3
PQITLWQRPIVTIKVEGQLTEAPLDTGADDTVLEDVNLPGRWKPKMIGGLGGFIKVRQYDQIHIEICGHKVIGT
VLVGPTPVNIIGRSMLTQIGCTLNF
>102782_174 0.3
PQITLWQRPIVTIKVEGQLTEAPLDTGADDTVLEDVNLPGRWKPKMIGGLGGFIKVRQYDQIHIEICGHKVIGT
VLIGPTPVNIIGRNMLTQIGCTLNF
>102782_175 0.3
PQITLWQRPIVTIKVEGQLTEAPLDTGADDTVLEDVNLPGRWKPKMIGGLGGFIKVRQYDQIHIEICGHKVIGT
VLIGPTPVNIIGRSMLTQIGCTLNF
>102782_180 0.3
PQITLWQRPIVTIKVEGQLTEAPLDTGADDTVFEDMNLPGRWKPKMIGGLGGFIKVRQYDQIHIEICGHKVIGT
VLVGPTPVNIIGRNMLTQIGCTLNF
>102782_181 0.3
PQITLWQRPIVTIKVEGQLTEAPLDTGADDTVFEDMNLPGRWKPKMIGGLGGFIKVRQYDQIHIEICGHKVIGT
VLVGPTPVNIIGRSMLTQIGCTLNF
>102782_182 0.3
PQITLWQRPIVTIKVEGQLTEAPLDTGADDTVFEDMNLPGRWKPKMIGGLGGFIKVRQYDQIHIEICGHKVIGT
VLIGPTPVNIIGRNMLTQIGCTLNF
>102782_183 0.3
PQITLWQRPIVTIKVEGQLTEAPLDTGADDTVFEDMNLPGRWKPKMIGGLGGFIKVRQYDQIHIEICGHKVIGT
VLIGPTPVNIIGRSMLTQIGCTLNF
>102782_188 0.3
PQITLWQRPIVTIKVEGQLTEAPLDTGADDTVFEDVNLPGRWKPKMIGGLGGFIKVRQYDQIHIEICGHKVIGT
VLVGPTPVNIIGRNMLTQIGCTLNF
>102782_189 0.3
PQITLWQRPIVTIKVEGQLTEAPLDTGADDTVFEDVNLPGRWKPKMIGGLGGFIKVRQYDQIHIEICGHKVIGT
VLVGPTPVNIIGRSMLTQIGCTLNF
>102782_190 0.3
PQITLWQRPIVTIKVEGQLTEAPLDTGADDTVFEDVNLPGRWKPKMIGGLGGFIKVRQYDQIHIEICGHKVIGT
VLIGPTPVNIIGRNMLTQIGCTLNF
>102782_191 0.3
PQITLWQRPIVTIKVEGQLTEAPLDTGADDTVFEDVNLPGRWKPKMIGGLGGFIKVRQYDQIHIEICGHKVIGT
VLIGPTPVNIIGRSMLTQIGCTLNF
>102782_32 0.3
PQITLWQRPIVTIKVEGQLKEAPLDTGADDTVLEDMNLPGRWKPKMIGGLGGFIKVRQYDQIHIEICGHKAIGT
VLVGPTPVNIIGRNMLTQIGCTLNF
>102782_33 0.3
PQITLWQRPIVTIKVEGQLKEAPLDTGADDTVLEDMNLPGRWKPKMIGGLGGFIKVRQYDQIHIEICGHKAIGT
VLVGPTPVNIIGRSMLTQIGCTLNF
>102782_34 0.3

PQITLWQRPIVTIKVEGQLKEAPLDTGADDTVLEDMNLPGRWKPKMIGGLGGFIKVRQYDQIHIEICGHKAIGT
VLIGPTPVNIIGRNMLTQIGCTLNF
>102782_35 0.3
PQITLWQRPIVTIKVEGQLKEAPLDTGADDTVLEDMNLPGRWKPKMIGGLGGFIKVRQYDQIHIEICGHKAIGT
VLIGPTPVNIIGRSMLTQIGCTLNF
>102782_40 0.3
PQITLWQRPIVTIKVEGQLKEAPLDTGADDTVLEDVNLPGRWKPKMIGGLGGFIKVRQYDQIHIEICGHKAIGT
VLVGPTPVNIIGRNMLTQIGCTLNF
>102782_41 0.3
PQITLWQRPIVTIKVEGQLKEAPLDTGADDTVLEDVNLPGRWKPKMIGGLGGFIKVRQYDQIHIEICGHKAIGT
VLVGPTPVNIIGRSMLTQIGCTLNF
>102782_59 0.3
PQITLWQRPIVTIKVEGQLKEAPLDTGADDTVFEDVNLPGRWKPKMIGGLGGFIKVRQYDQIHIEICGHKAIGT
VLIGPTPVNIIGRSMLTQIGCTLNF
>102782_160 0.3
PQITLWQRPIVTIKVEGQLTEAPLDTGADDTVLEDMNLPGRWKPKMIGGLGGFIKVRQYDQIHIEICGHKAIGT
VLVGPTPVNIIGRNMLTQIGCTLNF
>102782_161 0.3
PQITLWQRPIVTIKVEGQLTEAPLDTGADDTVLEDMNLPGRWKPKMIGGLGGFIKVRQYDQIHIEICGHKAIGT
VLVGPTPVNIIGRSMLTQIGCTLNF
>102782_162 0.3
PQITLWQRPIVTIKVEGQLTEAPLDTGADDTVLEDMNLPGRWKPKMIGGLGGFIKVRQYDQIHIEICGHKAIGT
VLIGPTPVNIIGRNMLTQIGCTLNF
>102782_163 0.3
PQITLWQRPIVTIKVEGQLTEAPLDTGADDTVLEDMNLPGRWKPKMIGGLGGFIKVRQYDQIHIEICGHKAIGT
VLIGPTPVNIIGRSMLTQIGCTLNF
>102783_1 0.3
PQITLWQRPIITIKIGGQLKEALLDTGADDTVLEDMNLPGRWKPKMIGGIGGFIKVRQYDEVPLEICGHKVTGT
VLIGSTPVNIIGRNLLTQLGCTLNF
>102782_4 0.3
PQITLWQRPIVTIKVEGQLKEALLDTGADDTVLEDMNLPGRWKPKMIGGLGGFIKVRQYDQIHIEICGHKVIGT
VLVGPTPVNIIGRNMLTQIGCTLNF
>102782_5 0.3
PQITLWQRPIVTIKVEGQLKEALLDTGADDTVLEDMNLPGRWKPKMIGGLGGFIKVRQYDQIHIEICGHKVIGT
VLVGPTPVNIIGRSMLTQIGCTLNF
>102782_6 0.3
PQITLWQRPIVTIKVEGQLKEALLDTGADDTVLEDMNLPGRWKPKMIGGLGGFIKVRQYDQIHIEICGHKVIGT
VLIGPTPVNIIGRNMLTQIGCTLNF
>102782_0 0.3
PQITLWQRPIVTIKVEGQLKEALLDTGADDTVLEDMNLPGRWKPKMIGGLGGFIKVRQYDQIHIEICGHKAIGT
VLVGPTPVNIIGRNMLTQIGCTLNF
>102782_10 0.3
PQITLWQRPIVTIKVEGQLKEALLDTGADDTVLEDVNLPGRWKPKMIGGLGGFIKVRQYDQIHIEICGHKAIGT
VLIGPTPVNIIGRNMLTQIGCTLNF
>102782_11 0.3
PQITLWQRPIVTIKVEGQLKEALLDTGADDTVLEDVNLPGRWKPKMIGGLGGFIKVRQYDQIHIEICGHKAIGT
VLIGPTPVNIIGRSMLTQIGCTLNF
>102782_16 0.3
PQITLWQRPIVTIKVEGQLKEALLDTGADDTVFEDMNLPGRWKPKMIGGLGGFIKVRQYDQIHIEICGHKAIGT
VLVGPTPVNIIGRNMLTQIGCTLNF
>102782_17 0.3
PQITLWQRPIVTIKVEGQLKEALLDTGADDTVFEDMNLPGRWKPKMIGGLGGFIKVRQYDQIHIEICGHKAIGT
VLVGPTPVNIIGRSMLTQIGCTLNF
>102782_129 0.3
PQITLWQRPIVTIKVEGQLTEALLDTGADDTVLEDMNLPGRWKPKMIGGLGGFIKVRQYDQIHIEICGHKAIGT
VLVGPTPVNIIGRSMLTQIGCTLNF
>10574_1 0.3
PQITLLQRPLVTIKIGGQIKEALLDTGADDTVLEGIELPGRWKPKMIGGIGGFIKVRQYEQVPVEICGHKAIGTVL
VGPTPVDIIGRNLMTQLGCTLNF
>105308_14 0.3
PQITLWQRPIVTVKVGGQLKEALIDTGADDTVLEEIELPGRWKPKIIGGIGGFVKVRQYDQIPIEICGHKIIGTVL
VGPTPTNIIGRNLLTQIGCTLNF
>105308_27 0.3

PQITLWQRPIVTVRVGGQLKEALIDTGADDTVLEEIELPGRWKPKIIGGIGGFVKVRQYDQIPIEICGHKVIGTVL
VGPTPTNIIGRNLLTQLGCTLNF
>105308_30 0.3
PQITLWQRPIVTVRVGGQLKEALIDTGADDTVLEEIELPGRWKPKIIGGIGGFVKVRQYDQIPIEICGHKIIGTVL
VGPTPTNIIGRNLLTQIGCTLNF
>105308_31 0.3
PQITLWQRPIVTVRVGGQLKEALIDTGADDTVLEEIELPGRWKPKIIGGIGGFVKVRQYDQIPIEICGHKIIGTVL
VGPTPTNIIGRNLLTQLGCTLNF
>105308_34 0.3
PQITLWQRPVVTVKVGGQLKEALIDTGADDTVLEEMELPGRWKPKIIGGIGGFVKVRQYDQIPIEICGHKVIGT
VLVGPTPTNIIGRNLLTQIGCTLNF
>105308_35 0.3
PQITLWQRPVVTVKVGGQLKEALIDTGADDTVLEEMELPGRWKPKIIGGIGGFVKVRQYDQIPIEICGHKVIGT
VLVGPTPTNIIGRNLLTQLGCTLNF
>105308_38 0.3
PQITLWQRPVVTVKVGGQLKEALIDTGADDTVLEEMELPGRWKPKIIGGIGGFVKVRQYDQIPIEICGHKIIGTV
LVGPTPTNIIGRNLLTQIGCTLNF
>105308_39 0.3
PQITLWQRPVVTVKVGGQLKEALIDTGADDTVLEEMELPGRWKPKIIGGIGGFVKVRQYDQIPIEICGHKIIGTV
LVGPTPTNIIGRNLLTQLGCTLNF
>105308_42 0.3
PQITLWQRPVVTVKVGGQLKEALIDTGADDTVLEEIELPGRWKPKIIGGIGGFVKVRQYDQIPIEICGHKVIGTV
LVGPTPTNIIGRNLLTQIGCTLNF
>105308_43 0.3
PQITLWQRPVVTVKVGGQLKEALIDTGADDTVLEEIELPGRWKPKIIGGIGGFVKVRQYDQIPIEICGHKVIGTV
LVGPTPTNIIGRNLLTQLGCTLNF
>105308_46 0.3
PQITLWQRPVVTVKVGGQLKEALIDTGADDTVLEEIELPGRWKPKIIGGIGGFVKVRQYDQIPIEICGHKIIGTVL
VGPTPTNIIGRNLLTQIGCTLNF
>105308_47 0.3
PQITLWQRPVVTVKVGGQLKEALIDTGADDTVLEEIELPGRWKPKIIGGIGGFVKVRQYDQIPIEICGHKIIGTVL
VGPTPTNIIGRNLLTQLGCTLNF
>105308_50 0.3
PQITLWQRPVVTVRVGGQLKEALIDTGADDTVLEEMELPGRWKPKIIGGIGGFVKVRQYDQIPIEICGHKVIGT
VLVGPTPTNIIGRNLLTQIGCTLNF
>105308_51 0.3
PQITLWQRPVVTVRVGGQLKEALIDTGADDTVLEEMELPGRWKPKIIGGIGGFVKVRQYDQIPIEICGHKVIGT
VLVGPTPTNIIGRNLLTQLGCTLNF
>105308_54 0.3
PQITLWQRPVVTVRVGGQLKEALIDTGADDTVLEEMELPGRWKPKIIGGIGGFVKVRQYDQIPIEICGHKIIGTV
LVGPTPTNIIGRNLLTQIGCTLNF
>105308_55 0.3
PQITLWQRPVVTVRVGGQLKEALIDTGADDTVLEEMELPGRWKPKIIGGIGGFVKVRQYDQIPIEICGHKIIGTV
LVGPTPTNIIGRNLLTQLGCTLNF
>105308_58 0.3
PQITLWQRPVVTVRVGGQLKEALIDTGADDTVLEEIELPGRWKPKIIGGIGGFVKVRQYDQIPIEICGHKVIGTV
LVGPTPTNIIGRNLLTQIGCTLNF
>105308_59 0.3
PQITLWQRPVVTVRVGGQLKEALIDTGADDTVLEEIELPGRWKPKIIGGIGGFVKVRQYDQIPIEICGHKVIGTV
LVGPTPTNIIGRNLLTQLGCTLNF
>105308_62 0.3
PQITLWQRPVVTVRVGGQLKEALIDTGADDTVLEEIELPGRWKPKIIGGIGGFVKVRQYDQIPIEICGHKIIGTVL
VGPTPTNIIGRNLLTQIGCTLNF
>105308_63 0.3
PQITLWQRPVVTVRVGGQLKEALIDTGADDTVLEEIELPGRWKPKIIGGIGGFVKVRQYDQIPIEICGHKIIGTVL
VGPTPTNIIGRNLLTQLGCTLNF
>75054_0 0.3
PQITLWQRPLVTIKIGGQLKEALLDTGADDTVLEEMNLPGRWKPKMIGGIGGFIKVRQYDQITIEICGHKAIGTV
LVGPTPVNIIGRNLLTQIGCTLNF
>75054_2 0.3
PQITLWQRPLVTIKIGGQLKEALLDTGADDTVLEEMNLPGRWKPKMIGGIGGFIKVRQYDQITIDICGHKAIGTV
LVGPTPVNIIGRNLLTQIGCTLNF
>75054_4 0.3

PQITLWQRPLVTIKIGGQLKEALLDTGADDTVLEEINLPGRWKPKMIGGIGGFIKVRQYDQITIEICGHKAIGTVL
VGPTPVNIIGRNLLTQIGCTLNF
>75054_6 0.3
PQITLWQRPLVTIKIGGQLKEALLDTGADDTVLEEINLPGRWKPKMIGGIGGFIKVRQYDQITIDICGHKAIGTVL
VGPTPVNIIGRNLLTQIGCTLNF
>75054_8 0.3
PQITLWQRPIVTIKIGGQLKEALLDTGADDTVLEEMNLPGRWKPKMIGGIGGFIKVRQYDQITIEICGHKAIGTV
LVGPTPVNIIGRNLLTQIGCTLNF
>75054_10 0.3
PQITLWQRPIVTIKIGGQLKEALLDTGADDTVLEEMNLPGRWKPKMIGGIGGFIKVRQYDQITIDICGHKAIGTV
LVGPTPVNIIGRNLLTQIGCTLNF
>75054_12 0.3
PQITLWQRPIVTIKIGGQLKEALLDTGADDTVLEEINLPGRWKPKMIGGIGGFIKVRQYDQITIEICGHKAIGTVL
VGPTPVNIIGRNLLTQIGCTLNF
>75054_14 0.3
PQITLWQRPIVTIKIGGQLKEALLDTGADDTVLEEINLPGRWKPKMIGGIGGFIKVRQYDQITIDICGHKAIGTVL
VGPTPVNIIGRNLLTQIGCTLNF
>75114_0 0.3
PQITLWQRPLVTIKIGGQLKEALLDTGADDTVLEDMDLPGRWKPKMIGGIGGFIKVRQYDQISIEICGHKAIGTV
LVGPTPINIIGRNLLTQIGCTLNF
>148111_0 0.2
PQITLWQRPLVTVKIGGQLKEALLDTGADDTVLEEMNLPGRWKPRMIGGIGGFIKVRQYDQISMEICGHKAIG
TVLIGPTPVNIIGRNLLTQIGCTLNF
>148111_1 0.2
PQITLWQRPLVTVKIGGQLKEALLDTGADDTVLEEMNLPGRWKPRMIGGIGGFIKVRQYDQVSMEICGHKAIG
TVLIGPTPVNIIGRNLLTQIGCTLNF
>9706_0 0.3
PQITLWQRPLVTIKIGGQLKEALLDTGADDTVLEDMDLPGRWKPKMIGGIGGFIKVRQYDQILIEICGHKAIGTV
LVGPTPVNIIGRNLLTQIGCTLNF
>187173_0 0.2
PQITLWQRPLVTVKIGGQLKEALLDTGADDTVLEDINLPGKWKPKMIGGIGGFIKVRQYDQILIEICGKKAIGTVL
VGPTPVNIIGRNMLTQIGCTLNF
>187173_1 0.2
PQITLWQRPLVTVKIGGQLKEALLDTGADDTVLEDINLPGKWKPKMIGGIGGFIKVRQYDQILIEICGKKAIGTVL
IGPTPVNIIGRNMLTQIGCTLNF
>187173_2 0.2
PQITLWQRPLVTVKIGGQLKEALLDTGADDTVLEDINLPGNWKPKMIGGIGGFIKVRQYDQILIEICGKKAIGTV
LVGPTPVNIIGRNMLTQIGCTLNF
>187173_3 0.2
PQITLWQRPLVTVKIGGQLKEALLDTGADDTVLEDINLPGNWKPKMIGGIGGFIKVRQYDQILIEICGKKAIGTV
LIGPTPVNIIGRNMLTQIGCTLNF
>187173_4 0.2
PQITLWQRPLVTVKIGGQLTEALLDTGADDTVLEDINLPGKWKPKMIGGIGGFIKVRQYDQILIEICGKKAIGTVL
VGPTPVNIIGRNMLTQIGCTLNF
>187173_5 0.2
PQITLWQRPLVTVKIGGQLTEALLDTGADDTVLEDINLPGKWKPKMIGGIGGFIKVRQYDQILIEICGKKAIGTVL
IGPTPVNIIGRNMLTQIGCTLNF
>187173_6 0.2
PQITLWQRPLVTVKIGGQLTEALLDTGADDTVLEDINLPGNWKPKMIGGIGGFIKVRQYDQILIEICGKKAIGTVL
VGPTPVNIIGRNMLTQIGCTLNF
>187173_7 0.2
PQITLWQRPLVTVKIGGQLTEALLDTGADDTVLEDINLPGNWKPKMIGGIGGFIKVRQYDQILIEICGKKAIGTVL
IGPTPVNIIGRNMLTQIGCTLNF
>187173_8 0.2
PQITLWQRPLVTVRIGGQLKEALLDTGADDTVLEDINLPGKWKPKMIGGIGGFIKVRQYDQILIEICGKKAIGTV
LVGPTPVNIIGRNMLTQIGCTLNF
>187173_9 0.2
PQITLWQRPLVTVRIGGQLKEALLDTGADDTVLEDINLPGKWKPKMIGGIGGFIKVRQYDQILIEICGKKAIGTV
LIGPTPVNIIGRNMLTQIGCTLNF
>187173_10 0.2
PQITLWQRPLVTVRIGGQLKEALLDTGADDTVLEDINLPGNWKPKMIGGIGGFIKVRQYDQILIEICGKKAIGTV
LVGPTPVNIIGRNMLTQIGCTLNF
>187173_11 0.2

PQITLWQRPLVTVRIGGQLKEALLDTGADDTVLEDINLPGNWKPKMIGGIGGFIKVRQYDQILIEICGKKAIGTV
LIGPTPVNIIGRNMLTQIGCTLNF
>187173_12 0.2
PQITLWQRPLVTVRIGGQLTEALLDTGADDTVLEDINLPGKWKPKMIGGIGGFIKVRQYDQILIEICGKKAIGTVL
VGPTPVNIIGRNMLTQIGCTLNF
>187173_13 0.2
PQITLWQRPLVTVRIGGQLTEALLDTGADDTVLEDINLPGKWKPKMIGGIGGFIKVRQYDQILIEICGKKAIGTVL
IGPTPVNIIGRNMLTQIGCTLNF
>187173_14 0.2
PQITLWQRPLVTVRIGGQLTEALLDTGADDTVLEDINLPGNWKPKMIGGIGGFIKVRQYDQILIEICGKKAIGTVL
VGPTPVNIIGRNMLTQIGCTLNF
>187173_15 0.2
PQITLWQRPLVTVRIGGQLTEALLDTGADDTVLEDINLPGNWKPKMIGGIGGFIKVRQYDQILIEICGKKAIGTVL
IGPTPVNIIGRNMLTQIGCTLNF
>187173_16 0.2
PQITLWQRPIVTVKIGGQLKEALLDTGADDTVLEDINLPGKWKPKMIGGIGGFIKVRQYDQILIEICGKKAIGTVL
VGPTPVNIIGRNMLTQIGCTLNF
>187173_17 0.2
PQITLWQRPIVTVKIGGQLKEALLDTGADDTVLEDINLPGKWKPKMIGGIGGFIKVRQYDQILIEICGKKAIGTVL
IGPTPVNIIGRNMLTQIGCTLNF
>187173_18 0.2
PQITLWQRPIVTVKIGGQLKEALLDTGADDTVLEDINLPGNWKPKMIGGIGGFIKVRQYDQILIEICGKKAIGTVL
VGPTPVNIIGRNMLTQIGCTLNF
>187173_19 0.2
PQITLWQRPIVTVKIGGQLKEALLDTGADDTVLEDINLPGNWKPKMIGGIGGFIKVRQYDQILIEICGKKAIGTVL
IGPTPVNIIGRNMLTQIGCTLNF
>187173_20 0.2
PQITLWQRPIVTVKIGGQLTEALLDTGADDTVLEDINLPGKWKPKMIGGIGGFIKVRQYDQILIEICGKKAIGTVL
VGPTPVNIIGRNMLTQIGCTLNF
>187173_21 0.2
PQITLWQRPIVTVKIGGQLTEALLDTGADDTVLEDINLPGKWKPKMIGGIGGFIKVRQYDQILIEICGKKAIGTVLI
GPTPVNIIGRNMLTQIGCTLNF
>187173_22 0.2
PQITLWQRPIVTVKIGGQLTEALLDTGADDTVLEDINLPGNWKPKMIGGIGGFIKVRQYDQILIEICGKKAIGTVL
VGPTPVNIIGRNMLTQIGCTLNF
>187173_23 0.2
PQITLWQRPIVTVKIGGQLTEALLDTGADDTVLEDINLPGNWKPKMIGGIGGFIKVRQYDQILIEICGKKAIGTVLI
GPTPVNIIGRNMLTQIGCTLNF
>187173_24 0.2
PQITLWQRPIVTVRIGGQLKEALLDTGADDTVLEDINLPGKWKPKMIGGIGGFIKVRQYDQILIEICGKKAIGTVL
VGPTPVNIIGRNMLTQIGCTLNF
>187173_25 0.2
PQITLWQRPIVTVRIGGQLKEALLDTGADDTVLEDINLPGKWKPKMIGGIGGFIKVRQYDQILIEICGKKAIGTVL
IGPTPVNIIGRNMLTQIGCTLNF
>187173_26 0.2
PQITLWQRPIVTVRIGGQLKEALLDTGADDTVLEDINLPGNWKPKMIGGIGGFIKVRQYDQILIEICGKKAIGTVL
VGPTPVNIIGRNMLTQIGCTLNF
>187173_27 0.2
PQITLWQRPIVTVRIGGQLKEALLDTGADDTVLEDINLPGNWKPKMIGGIGGFIKVRQYDQILIEICGKKAIGTVL
IGPTPVNIIGRNMLTQIGCTLNF
>187173_28 0.2
PQITLWQRPIVTVRIGGQLTEALLDTGADDTVLEDINLPGKWKPKMIGGIGGFIKVRQYDQILIEICGKKAIGTVL
VGPTPVNIIGRNMLTQIGCTLNF
>187173_29 0.2
PQITLWQRPIVTVRIGGQLTEALLDTGADDTVLEDINLPGKWKPKMIGGIGGFIKVRQYDQILIEICGKKAIGTVLI
GPTPVNIIGRNMLTQIGCTLNF
>187173_30 0.2
PQITLWQRPIVTVRIGGQLTEALLDTGADDTVLEDINLPGNWKPKMIGGIGGFIKVRQYDQILIEICGKKAIGTVL
VGPTPVNIIGRNMLTQIGCTLNF
>187173_31 0.2
PQITLWQRPIVTVRIGGQLTEALLDTGADDTVLEDINLPGNWKPKMIGGIGGFIKVRQYDQILIEICGKKAIGTVL
IGPTPVNIIGRNMLTQIGCTLNF

Sequences for the NFV-resistant ensemble
>107985_112 600.0
PQITLWQRPLVAVKIGGQIIEALLDTGADNTVLENIELPGRWKPKMIGGIGGFIKVRQYEEIPIEICGHKVTGTVL
VGPTPVNIIGRDLMTQIGCTLNF
>107985_217 600.0
PQITLWQRPLIAIKIGGQIIEALLDTGADNTVLENIELPGRWKPKMIGGIGGLIKVRQYEEIPIEICGHKVTGTVLV
GPTPVDIIGRDLMTQIGCTLNF
>107985_219 600.0
PQITLWQRPLIAIKIGGQIIEALLDTGADNTVLENIELPGRWKPKMIGGIGGLIKVREYEEIPIEICGHKVTGTVLV
GPTPVDIIGRDLMTQIGCTLNF
>107985_220 600.0
PQITLWQRPLIAIKIGGQIIEALLDTGADNTVLENIELPGRWKPKMIGGIGGLVKVRQYEEIPIEICGHKVTGTVLV
GPTPVNIIGRDLMTQIGCTLNF
>107985_221 600.0
PQITLWQRPLIAIKIGGQIIEALLDTGADNTVLENIELPGRWKPKMIGGIGGLVKVRQYEEIPIEICGHKVTGTVLV
GPTPVDIIGRDLMTQIGCTLNF
>107985_222 600.0
PQITLWQRPLIAIKIGGQIIEALLDTGADNTVLENIELPGRWKPKMIGGIGGLVKVREYEEIPIEICGHKVTGTVLV
GPTPVNIIGRDLMTQIGCTLNF
>107985_223 600.0
PQITLWQRPLIAIKIGGQIIEALLDTGADNTVLENIELPGRWKPKMIGGIGGLVKVREYEEIPIEICGHKVTGTVLV
GPTPVDIIGRDLMTQIGCTLNF
>107985_224 600.0
PQITLWQRPLIAVKIGGQIIEALLDTGADNTVLENIDLPGRWKPKMIGGIGGFIKVRQYEEIPIEICGHKVTGTVLV
GPTPVNIIGRDLMTQIGCTLNF
>107985_225 600.0
PQITLWQRPLIAVKIGGQIIEALLDTGADNTVLENIDLPGRWKPKMIGGIGGFIKVRQYEEIPIEICGHKVTGTVLV
GPTPVDIIGRDLMTQIGCTLNF
>107985_226 600.0
PQITLWQRPLIAVKIGGQIIEALLDTGADNTVLENIDLPGRWKPKMIGGIGGFIKVREYEEIPIEICGHKVTGTVLV
GPTPVNIIGRDLMTQIGCTLNF
>107985_227 600.0
PQITLWQRPLIAVKIGGQIIEALLDTGADNTVLENIDLPGRWKPKMIGGIGGFIKVREYEEIPIEICGHKVTGTVLV
GPTPVDIIGRDLMTQIGCTLNF
>107985_228 600.0
PQITLWQRPLIAVKIGGQIIEALLDTGADNTVLENIDLPGRWKPKMIGGIGGFVKVRQYEEIPIEICGHKVTGTVL
VGPTPVNIIGRDLMTQIGCTLNF
>107985_229 600.0
PQITLWQRPLIAVKIGGQIIEALLDTGADNTVLENIDLPGRWKPKMIGGIGGFVKVRQYEEIPIEICGHKVTGTVL
VGPTPVDIIGRDLMTQIGCTLNF
>107985_230 600.0
PQITLWQRPLIAVKIGGQIIEALLDTGADNTVLENIDLPGRWKPKMIGGIGGFVKVREYEEIPIEICGHKVTGTVL
VGPTPVNIIGRDLMTQIGCTLNF
>107985_231 600.0
PQITLWQRPLIAVKIGGQIIEALLDTGADNTVLENIDLPGRWKPKMIGGIGGFVKVREYEEIPIEICGHKVTGTVL
VGPTPVDIIGRDLMTQIGCTLNF
>107985_232 600.0
PQITLWQRPLIAVKIGGQIIEALLDTGADNTVLENIDLPGRWKPKMIGGIGGLIKVRQYEEIPIEICGHKVTGTVLV
GPTPVNIIGRDLMTQIGCTLNF
>107985_233 600.0
PQITLWQRPLIAVKIGGQIIEALLDTGADNTVLENIDLPGRWKPKMIGGIGGLIKVRQYEEIPIEICGHKVTGTVLV
GPTPVDIIGRDLMTQIGCTLNF
>107985_234 600.0
PQITLWQRPLIAVKIGGQIIEALLDTGADNTVLENIDLPGRWKPKMIGGIGGLIKVREYEEIPIEICGHKVTGTVLV
GPTPVNIIGRDLMTQIGCTLNF
>107985_235 600.0
PQITLWQRPLIAVKIGGQIIEALLDTGADNTVLENIDLPGRWKPKMIGGIGGLIKVREYEEIPIEICGHKVTGTVLV
GPTPVDIIGRDLMTQIGCTLNF
>107985_236 600.0
PQITLWQRPLIAVKIGGQIIEALLDTGADNTVLENIDLPGRWKPKMIGGIGGLVKVRQYEEIPIEICGHKVTGTVL
VGPTPVNIIGRDLMTQIGCTLNF
>107985_237 600.0
PQITLWQRPLIAVKIGGQIIEALLDTGADNTVLENIDLPGRWKPKMIGGIGGLVKVRQYEEIPIEICGHKVTGTVL

VGPTPVDIIGRDLMTQIGCTLNF
>107985_238 600.0
PQITLWQRPLIAVKIGGQIIEALLDTGADNTVLENIDLPGRWKPKMIGGIGGLVKVREYEEIPIEICGHKVTGTVL
VGPTPVNIIGRDLMTQIGCTLNF
>107985_239 600.0
PQITLWQRPLIAVKIGGQIIEALLDTGADNTVLENIDLPGRWKPKMIGGIGGLVKVREYEEIPIEICGHKVTGTVL
VGPTPVDIIGRDLMTQIGCTLNF
>107985_240 600.0
PQITLWQRPLIAVKIGGQIIEALLDTGADNTVLENIELPGRWKPKMIGGIGGFIKVRQYEEIPIEICGHKVTGTVLV
GPTPVNIIGRDLMTQIGCTLNF
>107985_241 600.0
PQITLWQRPLIAVKIGGQIIEALLDTGADNTVLENIELPGRWKPKMIGGIGGFIKVRQYEEIPIEICGHKVTGTVLV
GPTPVDIIGRDLMTQIGCTLNF
>107985_242 600.0
PQITLWQRPLIAVKIGGQIIEALLDTGADNTVLENIELPGRWKPKMIGGIGGFIKVREYEEIPIEICGHKVTGTVLV
GPTPVNIIGRDLMTQIGCTLNF
>107985_243 600.0
PQITLWQRPLIAVKIGGQIIEALLDTGADNTVLENIELPGRWKPKMIGGIGGFIKVREYEEIPIEICGHKVTGTVLV
GPTPVDIIGRDLMTQIGCTLNF
>107985_244 600.0
PQITLWQRPLIAVKIGGQIIEALLDTGADNTVLENIELPGRWKPKMIGGIGGFVKVRQYEEIPIEICGHKVTGTVL
VGPTPVNIIGRDLMTQIGCTLNF
>107985_245 600.0
PQITLWQRPLIAVKIGGQIIEALLDTGADNTVLENIELPGRWKPKMIGGIGGFVKVRQYEEIPIEICGHKVTGTVL
VGPTPVDIIGRDLMTQIGCTLNF
>107985_246 600.0
PQITLWQRPLIAVKIGGQIIEALLDTGADNTVLENIELPGRWKPKMIGGIGGFVKVREYEEIPIEICGHKVTGTVL
VGPTPVNIIGRDLMTQIGCTLNF
>107985_247 600.0
PQITLWQRPLIAVKIGGQIIEALLDTGADNTVLENIELPGRWKPKMIGGIGGFVKVREYEEIPIEICGHKVTGTVL
VGPTPVDIIGRDLMTQIGCTLNF
>107985_248 600.0
PQITLWQRPLIAVKIGGQIIEALLDTGADNTVLENIELPGRWKPKMIGGIGGLIKVRQYEEIPIEICGHKVTGTVLV
GPTPVNIIGRDLMTQIGCTLNF
>107985_249 600.0
PQITLWQRPLIAVKIGGQIIEALLDTGADNTVLENIELPGRWKPKMIGGIGGLIKVRQYEEIPIEICGHKVTGTVLV
GPTPVDIIGRDLMTQIGCTLNF
>107985_250 600.0
PQITLWQRPLIAVKIGGQIIEALLDTGADNTVLENIELPGRWKPKMIGGIGGLIKVREYEEIPIEICGHKVTGTVLV
GPTPVNIIGRDLMTQIGCTLNF
>107985_251 600.0
PQITLWQRPLIAVKIGGQIIEALLDTGADNTVLENIELPGRWKPKMIGGIGGLIKVREYEEIPIEICGHKVTGTVLV
GPTPVDIIGRDLMTQIGCTLNF
>107985_252 600.0
PQITLWQRPLIAVKIGGQIIEALLDTGADNTVLENIELPGRWKPKMIGGIGGLVKVRQYEEIPIEICGHKVTGTVL
VGPTPVNIIGRDLMTQIGCTLNF
>107985_253 600.0
PQITLWQRPLIAVKIGGQIIEALLDTGADNTVLENIELPGRWKPKMIGGIGGLVKVRQYEEIPIEICGHKVTGTVL
VGPTPVDIIGRDLMTQIGCTLNF
>107985_254 600.0
PQITLWQRPLIAVKIGGQIIEALLDTGADNTVLENIELPGRWKPKMIGGIGGLVKVREYEEIPIEICGHKVTGTVL
VGPTPVNIIGRDLMTQIGCTLNF
>107985_255 600.0
PQITLWQRPLIAVKIGGQIIEALLDTGADNTVLENIELPGRWKPKMIGGIGGLVKVREYEEIPIEICGHKVTGTVL
VGPTPVDIIGRDLMTQIGCTLNF
>123049_0 600.0
PQITLWQRPIITIKIGGQLKDVLMDTGADDTVLEDIDLPGRWKPKLIGGIGGFVKVRQYDHVPVEVCGHKVTTT
VLVGPTPVDVIGRNLLTQIGCTLNF
>123049_1 600.0
PQITLWQRPIITIKIGGQLKDVLMDTGADDTVLEDIDLPGRWKPKLIGGIGGFVKVRQYDNVPVEVCGHKVTTT
VLVGPTPVDVIGRNLLTQIGCTLNF
>123049_2 600.0
PQITLWQRPIITIKIGGQLKDVLMDTGADDTVLEDIDLPGRWKPKLIGGIGGFVKVKQYDHVPVEVCGHKVTTT

VLVGPTPVDVIGRNLLTQIGCTLNF
>123049_3 600.0
PQITLWQRPIITIKIGGQLKDVLMDTGADDTVLEDIDLPGRWKPKLIGGIGGFVKVKQYDNVPVEVCGHKVTTT
VLVGPTPVDVIGRNLLTQIGCTLNF
>123049_4 600.0
PQITLWQRPIITIKIGGQLKDVLMDTGADDTVLEDIDLPGKWKPKLIGGIGGFVKVRQYDHVPVEVCGHKVTTT
VLVGPTPVDVIGRNLLTQIGCTLNF
>123049_5 600.0
PQITLWQRPIITIKIGGQLKDVLMDTGADDTVLEDIDLPGKWKPKLIGGIGGFVKVRQYDNVPVEVCGHKVTTT
VLVGPTPVDVIGRNLLTQIGCTLNF
>123049_6 600.0
PQITLWQRPIITIKIGGQLKDVLMDTGADDTVLEDIDLPGKWKPKLIGGIGGFVKVKQYDHVPVEVCGHKVTTT
VLVGPTPVDVIGRNLLTQIGCTLNF
>123049_7 600.0
PQITLWQRPIITIKIGGQLKDVLMDTGADDTVLEDIDLPGKWKPKLIGGIGGFVKVKQYDNVPVEVCGHKVTTT
VLVGPTPVDVIGRNLLTQIGCTLNF
>123049_8 600.0
PQITLWQRPIITIKIGGQLKDVLMDTGADDTVLENIDLPGRWKPKLIGGIGGFVKVRQYDHVPVEVCGHKVTTT
VLVGPTPVDVIGRNLLTQIGCTLNF
>123049_9 600.0
PQITLWQRPIITIKIGGQLKDVLMDTGADDTVLENIDLPGRWKPKLIGGIGGFVKVRQYDNVPVEVCGHKVTTT
VLVGPTPVDVIGRNLLTQIGCTLNF
>123049_10 600.0
PQITLWQRPIITIKIGGQLKDVLMDTGADDTVLENIDLPGRWKPKLIGGIGGFVKVKQYDHVPVEVCGHKVTTT
VLVGPTPVDVIGRNLLTQIGCTLNF
>123049_11 600.0
PQITLWQRPIITIKIGGQLKDVLMDTGADDTVLENIDLPGRWKPKLIGGIGGFVKVKQYDNVPVEVCGHKVTTT
VLVGPTPVDVIGRNLLTQIGCTLNF
>123049_12 600.0
PQITLWQRPIITIKIGGQLKDVLMDTGADDTVLENIDLPGKWKPKLIGGIGGFVKVRQYDHVPVEVCGHKVTTT
VLVGPTPVDVIGRNLLTQIGCTLNF
>123049_13 600.0
PQITLWQRPIITIKIGGQLKDVLMDTGADDTVLENIDLPGKWKPKLIGGIGGFVKVRQYDNVPVEVCGHKVTTT
VLVGPTPVDVIGRNLLTQIGCTLNF
>123049_14 600.0
PQITLWQRPIITIKIGGQLKDVLMDTGADDTVLENIDLPGKWKPKLIGGIGGFVKVKQYDHVPVEVCGHKVTTT
VLVGPTPVDVIGRNLLTQIGCTLNF
>123049_15 600.0
PQITLWQRPIITIKIGGQLKDVLMDTGADDTVLENIDLPGKWKPKLIGGIGGFVKVKQYDNVPVEVCGHKVTTT
VLVGPTPVDVIGRNLLTQIGCTLNF
>98601_0 600.0
PQITLWQRPFVTIKIGGQLIEALLDTGADDTVLEEMNLPGRWKPKIIGGIGGFMKVRQYDQIPIEICGHKVITTVL
IGPTPVNAIGRNLLTQIGCTLNF
>147975_1 600.0
PQITLWQRPILTVKVGGHLIEALLDTGADDTVLEGIDLPGRWKPKMIGGIGGFVKVRQYDQIPIEICGHKVLSTV
LVGPTPVNVIGRNLMTQIGCTLNF
>143332_24 600.0
PQITLWQRPIVTVKIGGHLVEVLLDTGADDTVLEEINLPGRWKPKMIGGIGGFVKVKQYDQVPIEIWGHKVLST
VLVGPTPVNVIGRNLMTQIGCTLNF
>143332_25 600.0
PQITLWQRPIVTVKIGGHLVEVLLDTGADDTVLEEINLPGRWKPKMIGGIGGFVKVKQYDQVPIEIWGHKVLST
VLVGPTPVNVVGRNLMTQIGCTLNF
>143332_81 600.0
PQITLWQRPIVTVKVGGHLVEVLLDTGADDTVIEEINLPGRWKPKMIGGIGGFVKVKQYDQVPIEIWGHKVLST
VLVGPTPVNVVGRNLMTQIGCTLNF
>143332_82 600.0
PQITLWQRPIVTVKVGGHLVEVLLDTGADDTVIEEINLPGRWKPKMIGGIGGFVRVKQYDQVPIEIWGHKVLST
VLVGPTPVNVIGRNLMTQIGCTLNF
>143332_83 600.0
PQITLWQRPIVTVKVGGHLVEVLLDTGADDTVIEEINLPGRWKPKMIGGIGGFVRVKQYDQVPIEIWGHKVLST
VLVGPTPVNVVGRNLMTQIGCTLNF
>143332_84 600.0
PQITLWQRPIVTVKVGGHLVEVLLDTGADDTVIEEINLPGRWKPKIIGGIGGFVKVKQYDQVPIEIWGHKVLSTV

LVGPTPVNVIGRNLMTQIGCTLNF
>143332_85 600.0
PQITLWQRPIVTVKVGGHLVEVLLDTGADDTVIEEINLPGRWKPKIIGGIGGFVKVKQYDQVPIEIWGHKVLSTV
LVGPTPVNVVGRNLMTQIGCTLNF
>143332_86 600.0
PQITLWQRPIVTVKVGGHLVEVLLDTGADDTVIEEINLPGRWKPKIIGGIGGFVRVKQYDQVPIEIWGHKVLSTV
LVGPTPVNVIGRNLMTQIGCTLNF
>143332_87 600.0
PQITLWQRPIVTVKVGGHLVEVLLDTGADDTVIEEINLPGRWKPKIIGGIGGFVRVKQYDQVPIEIWGHKVLSTV
LVGPTPVNVVGRNLMTQIGCTLNF
>143332_88 600.0
PQITLWQRPIVTVKVGGHLVEVLLDTGADDTVVEEINLPGRWKPKMIGGIGGFVKVKQYDQVPIEIWGHKVLS
TVLVGPTPVNVIGRNLMTQIGCTLNF
>143332_89 600.0
PQITLWQRPIVTVKVGGHLVEVLLDTGADDTVVEEINLPGRWKPKMIGGIGGFVKVKQYDQVPIEIWGHKVLS
TVLVGPTPVNVVGRNLMTQIGCTLNF
>143332_90 600.0
PQITLWQRPIVTVKVGGHLVEVLLDTGADDTVVEEINLPGRWKPKMIGGIGGFVRVKQYDQVPIEIWGHKVLS
TVLVGPTPVNVIGRNLMTQIGCTLNF
>143332_91 600.0
PQITLWQRPIVTVKVGGHLVEVLLDTGADDTVVEEINLPGRWKPKMIGGIGGFVRVKQYDQVPIEIWGHKVLS
TVLVGPTPVNVVGRNLMTQIGCTLNF
>143332_92 600.0
PQITLWQRPIVTVKVGGHLVEVLLDTGADDTVVEEINLPGRWKPKIIGGIGGFVKVKQYDQVPIEIWGHKVLST
VLVGPTPVNVIGRNLMTQIGCTLNF
>143332_93 600.0
PQITLWQRPIVTVKVGGHLVEVLLDTGADDTVVEEINLPGRWKPKIIGGIGGFVKVKQYDQVPIEIWGHKVLST
VLVGPTPVNVVGRNLMTQIGCTLNF
>143332_94 600.0
PQITLWQRPIVTVKVGGHLVEVLLDTGADDTVVEEINLPGRWKPKIIGGIGGFVRVKQYDQVPIEIWGHKVLST
VLVGPTPVNVIGRNLMTQIGCTLNF
>143332_95 600.0
PQITLWQRPIVTVKVGGHLVEVLLDTGADDTVVEEINLPGRWKPKIIGGIGGFVRVKQYDQVPIEIWGHKVLST
VLVGPTPVNVVGRNLMTQIGCTLNF
>103057_0 600.0
PQITLWQRPIVTVKIGGQLREALLDTGADDTVFEDIELPGRWTPKIIGGIGGFVKVKQYEEVPIEICEHKTTATVL
VGPTPFNIIGRNLMTQLGCTLNF
>103057_2 600.0
PQITLWQRPIVTVKIGGQLREALLDTGADDTVFEDIELPGRWTPKIIGGIGGFVKVKQYEEVPVEICEHKTTATV
LVGPTPFNIIGRNLMTQLGCTLNF
>56150_0 600.0
PQITLWRRPVVTIKVGGQLTEALLDTGADDTVFEDINLPGRWTPKMIMGIGGLVKVRQYDQVPIEFCGHKVLS
TVLVGPTPANIIGRNLMTQIGCTLNF
>56150_1 600.0
PQITLWRRPVVTIKVGGQLTEALLDTGADDTVFEDINLPGRWTPKMIMGIGGLVKVRQYDQVPIEFCGRKVLS
TVLVGPTPANIIGRNLMTQIGCTLNF
>56150_2 600.0
PQITLWRRPVVTIKVGGQLTEALLDTGADDTVFEDINLPGRWTPKMIMGIGGLVKVKQYDQVPIEFCGHKVLS
TVLVGPTPANIIGRNLMTQIGCTLNF
>56150_3 600.0
PQITLWRRPVVTIKVGGQLTEALLDTGADDTVFEDINLPGRWTPKMIMGIGGLVKVKQYDQVPIEFCGRKVLS
TVLVGPTPANIIGRNLMTQIGCTLNF
>143473_16 600.0
PQITLWQRPIVIVKIGGQLREALLDTGADDTVFEDIDLPGRWTPKLIGGIGGFVKVRQYDQVPIEICGHKIISTVL
VGPTPANIIGRNLMTQLGCTLNF
>143473_18 600.0
PQITLWQRPIVIVKIGGQLREALLDTGADDTVFEDIDLPGRWTPKLIGGIGGFVKVRQYDQVPIEICGHKVISTV
LVGPTPANIIGRNLMTQLGCTLNF
>143473_20 600.0
PQITLWQRPIVIVKIGGQLREALLDTGADDTVFEDIDLPGRWTPKLIGGIGGFVRVRQYDQVPIEICGHKIISTVL
VGPTPANIIGRNLMTQLGCTLNF
>143473_22 600.0
PQITLWQRPIVIVKIGGQLREALLDTGADDTVFEDIDLPGRWTPKLIGGIGGFVRVRQYDQVPIEICGHKVISTV

LVGPTPANIIGRNLMTQLGCTLNF
>143473_24 600.0
PQITLWQRPIVIVKIGGQIREALLDTGADDTVFEDIDLPGRWTPKLIGGIGGFVKVRQYDQVPIEICGHKIISTVL
VGPTPANIIGRNLMTQLGCTLNF
>143473_26 600.0
PQITLWQRPIVIVKIGGQIREALLDTGADDTVFEDIDLPGRWTPKLIGGIGGFVKVRQYDQVPIEICGHKVISTVL
VGPTPANIIGRNLMTQLGCTLNF
>143473_28 600.0
PQITLWQRPIVIVKIGGQIREALLDTGADDTVFEDIDLPGRWTPKLIGGIGGFVRVRQYDQVPIEICGHKIISTVL
VGPTPANIIGRNLMTQLGCTLNF
>143473_30 600.0
PQITLWQRPIVIVKIGGQIREALLDTGADDTVFEDIDLPGRWTPKLIGGIGGFVRVRQYDQVPIEICGHKVISTVL
VGPTPANIIGRNLMTQLGCTLNF
>148053_0 600.0
PQITLWQRPIVTVKIEGQLREALLDTGADNTVFEDITLPGRWKPRMIGGIGGFVKVKEYDQITIDICGHKAVGTV
LVGPTPANIIGRDLLTQIGCTLNF
>148053_1 600.0
PQITLWQRPIVTVKIEGQLREALLDTGADNTVFEDITLPGRWKPRMIGGIGGFVKVKEYDQVTIDICGHKAVGT
VLVGPTPANIIGRDLLTQIGCTLNF
>148053_2 600.0
PQITLWQRPVVTVKIEGQLREALLDTGADNTVFEDITLPGRWKPRMIGGIGGFVKVKEYDQITIDICGHKAVGT
VLVGPTPANIIGRDLLTQIGCTLNF
>5279_0 600.0
PQITLWQRPLVTVKIGGQLREALLDTGADNTVLEDISLPGRWKPKMIGGIGGFIKVKEYDQVPIEICGHKTIGTV
LVGPTPVNVIGRDLMTQIGCTLNF
>41212_0 600.0
PQITLWQRPLVTVKIGGQLKEALLDTGADNTVIEDINLPGRWKPKLIGGIGGFVKVKQYDQIPIEICGHKTIGTVL
VGPTPVNIIGRDLMTQLGCTLNF
>41212_1 600.0
PQITLWQRPLVTVKIGGQLKEALLDTGADNTVIEDINLPGRWNPKLIGGIGGFVKVKQYDQIPIEICGHKTIGTVL
VGPTPVNIIGRDLMTQLGCTLNF
>41212_2 600.0
PQITLWQRPLVTVKIGGQLKEALLDTGADNTVIEDINFPGRWKPKLIGGIGGFVKVKQYDQIPIEICGHKTIGTVL
VGPTPVNIIGRDLMTQLGCTLNF
>41212_3 600.0
PQITLWQRPLVTVKIGGQLKEALLDTGADNTVIEDINFPGRWNPKLIGGIGGFVKVKQYDQIPIEICGHKTIGTVL
VGPTPVNIIGRDLMTQLGCTLNF
>41212_8 600.0
PQITLWQRPLVTVKIGGQLKEALLDTGADNTVMEDINLPGRWKPKLIGGIGGFVKVKQYDQIPIEICGHKTIGTV
LVGPTPVNIIGRDLMTQLGCTLNF
>41212_9 600.0
PQITLWQRPLVTVKIGGQLKEALLDTGADNTVMEDINLPGRWNPKLIGGIGGFVKVKQYDQIPIEICGHKTIGTV
LVGPTPVNIIGRDLMTQLGCTLNF
>41212_10 600.0
PQITLWQRPLVTVKIGGQLKEALLDTGADNTVMEDINFPGRWKPKLIGGIGGFVKVKQYDQIPIEICGHKTIGTV
LVGPTPVNIIGRDLMTQLGCTLNF
>41212_11 600.0
PQITLWQRPLVTVKIGGQLKEALLDTGADNTVMEDINFPGRWNPKLIGGIGGFVKVKQYDQIPIEICGHKTIGT
VLVGPTPVNIIGRDLMTQLGCTLNF

Sequences for the NFV-susceptible ensemble
>81921_0 0.4
PQITLWQRPLVAIKIEGQLKEALLDTGADDTVLEEMNLPGRWKPKMIGGIGGFIKVRQYDQILIEICGHKAIGTV
LVGPTPVNIIGRNLLTQIGCTLNF
>81921_1 0.4
PQITLWQRPLVAIKIEGQLKEALLDTGADDTVLEEMNLPGRWKPKMIGGIGGFIKVRQYDQILIEICGHKAIGTV
LIGPTPVNIIGRNLLTQIGCTLNF
>81921_2 0.4
PQITLWQRPLVAIKVEGQLKEALLDTGADDTVLEEMNLPGRWKPKMIGGIGGFIKVRQYDQILIEICGHKAIGTV
LVGPTPVNIIGRNLLTQIGCTLNF
>81921_3 0.4
PQITLWQRPLVAIKVEGQLKEALLDTGADDTVLEEMNLPGRWKPKMIGGIGGFIKVRQYDQILIEICGHKAIGTV
LIGPTPVNIIGRNLLTQIGCTLNF

>81921_4 0.4
PQITLWQRPLVAIRIEGQLKEALLDTGADDTVLEEMNLPGRWKPKMIGGIGGFIKVRQYDQILIEICGHKAIGTV
LVGPTPVNIIGRNLLTQIGCTLNF
>81921_5 0.4
PQITLWQRPLVAIRIEGQLKEALLDTGADDTVLEEMNLPGRWKPKMIGGIGGFIKVRQYDQILIEICGHKAIGTV
LIGPTPVNIIGRNLLTQIGCTLNF
>81921_6 0.4
PQITLWQRPLVAIRVEGQLKEALLDTGADDTVLEEMNLPGRWKPKMIGGIGGFIKVRQYDQILIEICGHKAIGTV
LVGPTPVNIIGRNLLTQIGCTLNF
>81921_7 0.4
PQITLWQRPLVAIRVEGQLKEALLDTGADDTVLEEMNLPGRWKPKMIGGIGGFIKVRQYDQILIEICGHKAIGTV
LIGPTPVNIIGRNLLTQIGCTLNF
>81921_8 0.4
PQITLWQRPLVPIKIEGQLKEALLDTGADDTVLEEMNLPGRWKPKMIGGIGGFIKVRQYDQILIEICGHKAIGTV
LVGPTPVNIIGRNLLTQIGCTLNF
>81921_9 0.4
PQITLWQRPLVPIKIEGQLKEALLDTGADDTVLEEMNLPGRWKPKMIGGIGGFIKVRQYDQILIEICGHKAIGTV
LIGPTPVNIIGRNLLTQIGCTLNF
>81921_10 0.4
PQITLWQRPLVPIKVEGQLKEALLDTGADDTVLEEMNLPGRWKPKMIGGIGGFIKVRQYDQILIEICGHKAIGTV
LVGPTPVNIIGRNLLTQIGCTLNF
>81921_11 0.4
PQITLWQRPLVPIKVEGQLKEALLDTGADDTVLEEMNLPGRWKPKMIGGIGGFIKVRQYDQILIEICGHKAIGTV
LIGPTPVNIIGRNLLTQIGCTLNF
>81921_12 0.4
PQITLWQRPLVPIRIEGQLKEALLDTGADDTVLEEMNLPGRWKPKMIGGIGGFIKVRQYDQILIEICGHKAIGTV
LVGPTPVNIIGRNLLTQIGCTLNF
>81921_13 0.4
PQITLWQRPLVPIRIEGQLKEALLDTGADDTVLEEMNLPGRWKPKMIGGIGGFIKVRQYDQILIEICGHKAIGTV
LIGPTPVNIIGRNLLTQIGCTLNF
>81921_14 0.4
PQITLWQRPLVPIRVEGQLKEALLDTGADDTVLEEMNLPGRWKPKMIGGIGGFIKVRQYDQILIEICGHKAIGT
VLVGPTPVNIIGRNLLTQIGCTLNF
>81921_15 0.4
PQITLWQRPLVPIRVEGQLKEALLDTGADDTVLEEMNLPGRWKPKMIGGIGGFIKVRQYDQILIEICGHKAIGT
VLIGPTPVNIIGRNLLTQIGCTLNF
>206433_10 0.5
PQITLWQRPLVTAKIGGQLKEALLDTGADDTVLEEMSLPGRWKPKMIGGIGGFIKVGQYDQILIEICGHKAIGTV
LVGPTPVNIIGRNLLTQIGCTLNF
>61111_0 0.4
PQITLWQRPLVTIKIGGQTKEALLDTGADDTVLEEMNLPGRWKPKMIGGIGGFIKVRQYDQIPIEICGHKATGT
VLVGPTPVNIIGRNLLTQIGCTLNF
>61111_1 0.4
PQITLWQRPLVTIKIGGQTKEALLDTGADDTVLEEMNLPGRWKPKMIGGIGGFIKVKQYDQIPIEICGHKATGT
VLVGPTPVNIIGRNLLTQIGCTLNF
>44058_2 0.2
PQITLWQRPLVAIKIGGQLKEALLDTGADDTVLEEMNLPGKWKPKMIGGIGGFIKVRQYDQITIEICGHKAIGTV
LVGPTPVNIIGRNLLTQIGCTLNF
>44058_3 0.2
PQITLWQRPLVAIRIGGQLKEALLDTGADDTVLEEMNLPGKWKPKMIGGIGGFIKVRQYDQITIEICGHKAIGTV
LVGPTPVNIIGRNLLTQIGCTLNF
>45063_0 0.5
PQITLWQRPLVTIKIEGQLKEALLDTGADDTVLEDMNLPGRWKPKMIGGIGGFIKVRQYDQVSIEICGHRAIGT
VLVGPTPVNIIGRNLLTQIGCTLNF
>216605_0 0.5
PQITLWQRPLVSIKIGGQIKEALLDTGADDTVLEEMSLPGRWKPKMIGGIGGFIKVGQYDQIAIEICGHKAIGTV
LIGPTPVNIIGRNLLTQIGCTLNF
>109458_0 0.4
PQITLWQRPLVTIKIGGQLKEALLDTGADDTVLEDIDLPGRWKPKMIGGIGGFIKVRQYDQILVEICGYKAIGTVL
VGPTPVNIIGRNLLTQIGCTLNF
>109458_1 0.4
PQITLWQRPLVTIKIGGQLKEALLDTGADDTVLEDIDLPGKWKPKMIGGIGGFIKVRQYDQILVEICGYKAIGTVL
VGPTPVNIIGRNLLTQIGCTLNF

>148081_0 0.4
PQITLWQRPLVTIKIGGQTKEALLDTGADDTVLEEMNLPGKWKPKMIGGIGGFIKVRQYDQILIEICGHKAIGTV
LVGPTPVNIIGRNLLTQIGCTLNF
>148081_1 0.4
PQITLWQRPLVTIKIGGQTKEALLDTGADDTVLEEMNLPGKWKPKMIGGIGGFIKVRQYDQILVEICGHKAIGTV
LVGPTPVNIIGRNLLTQIGCTLNF
>148081_2 0.4
PQITLWQRPLVTIKVGGQTKEALLDTGADDTVLEEMNLPGKWKPKMIGGIGGFIKVRQYDQILIEICGHKAIGT
VLVGPTPVNIIGRNLLTQIGCTLNF
>148081_3 0.4
PQITLWQRPLVTIKVGGQTKEALLDTGADDTVLEEMNLPGKWKPKMIGGIGGFIKVRQYDQILVEICGHKAIGT
VLVGPTPVNIIGRNLLTQIGCTLNF
>148081_4 0.4
PQITLWQRPLVTIRIGGQTKEALLDTGADDTVLEEMNLPGKWKPKMIGGIGGFIKVRQYDQILIEICGHKAIGTV
LVGPTPVNIIGRNLLTQIGCTLNF
>148081_5 0.4
PQITLWQRPLVTIRIGGQTKEALLDTGADDTVLEEMNLPGKWKPKMIGGIGGFIKVRQYDQILVEICGHKAIGT
VLVGPTPVNIIGRNLLTQIGCTLNF
>148081_6 0.4
PQITLWQRPLVTIRVGGQTKEALLDTGADDTVLEEMNLPGKWKPKMIGGIGGFIKVRQYDQILIEICGHKAIGT
VLVGPTPVNIIGRNLLTQIGCTLNF
>148081_7 0.4
PQITLWQRPLVTIRVGGQTKEALLDTGADDTVLEEMNLPGKWKPKMIGGIGGFIKVRQYDQILVEICGHKAIGT
VLVGPTPVNIIGRNLLTQIGCTLNF
>75054_1 0.4
PQITLWQRPLVTIKIGGQLKEALLDTGADDTVLEEMNLPGRWKPKMIGGIGGFIKVRQYDQITIEICGHKATGTV
LVGPTPVNIIGRNLLTQIGCTLNF
>75054_3 0.4
PQITLWQRPLVTIKIGGQLKEALLDTGADDTVLEEMNLPGRWKPKMIGGIGGFIKVRQYDQITIDICGHKATGT
VLVGPTPVNIIGRNLLTQIGCTLNF
>75054_5 0.4
PQITLWQRPLVTIKIGGQLKEALLDTGADDTVLEEINLPGRWKPKMIGGIGGFIKVRQYDQITIEICGHKATGTVL
VGPTPVNIIGRNLLTQIGCTLNF
>75054_7 0.4
PQITLWQRPLVTIKIGGQLKEALLDTGADDTVLEEINLPGRWKPKMIGGIGGFIKVRQYDQITIDICGHKATGTVL
VGPTPVNIIGRNLLTQIGCTLNF
>75054_9 0.4
PQITLWQRPIVTIKIGGQLKEALLDTGADDTVLEEMNLPGRWKPKMIGGIGGFIKVRQYDQITIEICGHKATGTV
LVGPTPVNIIGRNLLTQIGCTLNF
>75054_11 0.4
PQITLWQRPIVTIKIGGQLKEALLDTGADDTVLEEMNLPGRWKPKMIGGIGGFIKVRQYDQITIDICGHKATGTV
LVGPTPVNIIGRNLLTQIGCTLNF
>75054_13 0.4
PQITLWQRPIVTIKIGGQLKEALLDTGADDTVLEEINLPGRWKPKMIGGIGGFIKVRQYDQITIEICGHKATGTVL
VGPTPVNIIGRNLLTQIGCTLNF
>75054_15 0.4
PQITLWQRPIVTIKIGGQLKEALLDTGADDTVLEEINLPGRWKPKMIGGIGGFIKVRQYDQITIDICGHKATGTVL
VGPTPVNIIGRNLLTQIGCTLNF
>13260_0 0.4
PQITLWQRPLVTIKIGGQLTEALLDTGADDTVLEEMNLPGRWKPKMIGGIGGFIKVGQYDQILIEICGHKAIGTV
LVGPTPVNIIGRNLLTQIGCTLNF
>206433_2 0.5
PQITLWQRPLVTIKIGGQLKEALLDTGADDTVLEEMSLPGRWKPKMIGGIGGFIKVGQYDQILIEICGHKAIGTV
LVGPTPVNIIGRNLLTQIGCTLNF
>206433_3 0.5
PQITLWQRPLVTIKIGGQLKEALLDTGADDTVLEEMSLPGRWKPKMIGGIGGFIKVGQYDQILIEICGHKAVGTV
LVGPTPVNIIGRNLLTQIGCTLNF
>206433_6 0.5
PQITLWQRPLVTIKVGGQLKEALLDTGADDTVLEEMSLPGRWKPKMIGGIGGFIKVGQYDQILIEICGHKAIGT
VLVGPTPVNIIGRNLLTQIGCTLNF
>206433_7 0.5
PQITLWQRPLVTIKVGGQLKEALLDTGADDTVLEEMSLPGRWKPKMIGGIGGFIKVGQYDQILIEICGHKAVGT
VLVGPTPVNIIGRNLLTQIGCTLNF

>148029_0 0.4
PQITLWQRPLVTVKIGGQVKEALLDTGADDTVLEEMNLPGKWKPKMIGGIGGFIKVRQYDNILVDICGHKAEG
TVLVGPTPVNIIGRNLLTQIGCTLNF
>148029_1 0.4
PQITLWQRPLVTVKIGGQVKEALLDTGADDTVLEEINLPGKWKPKMIGGIGGFIKVRQYDNILVDICGHKAEGT
VLVGPTPVNIIGRNLLTQIGCTLNF
>81788_0 0.5
PQITLWQRPLVTIKIGGQLKEALLDTGADDTVLEEMELPGRWKPKMIGGIGGFVKVRQYEQILVEICGHKAIGT
VLVGPTPANIIGRNLLTQIGCTLNF
>102774_3 0.4
PQITLWQRPLVTIKIGGQLKEALLDTGADDTVLEEMNLSGRWKPKMIGGLGGFIKVRQYDQIPIEICGHKAIGT
VLIGPTPVNIIGRNLLTQLGCTLNF
>102774_6 0.4
PQITLWQRPLVTIKIGGQLKEALLDTGADDTVLQEMNLSGRWKPKMIGGIGGFIKVRQYDQIPIEICGHKAIGTV
LIGPTPVNIIGRNLLTQLGCTLNF
>102774_7 0.4
PQITLWQRPLVTIKIGGQLKEALLDTGADDTVLQEMNLSGRWKPKMIGGLGGFIKVRQYDQIPIEICGHKAIGT
VLIGPTPVNIIGRNLLTQLGCTLNF
>102774_11 0.4
PQITLWQRPIVTIKIGGQLKEALLDTGADDTVLEEMNLSGRWKPKMIGGLGGFIKVRQYDQIPIEICGHKAIGTV
LIGPTPVNIIGRNLLTQLGCTLNF
>102774_14 0.4
PQITLWQRPIVTIKIGGQLKEALLDTGADDTVLQEMNLSGRWKPKMIGGIGGFIKVRQYDQIPIEICGHKAIGTV
LIGPTPVNIIGRNLLTQLGCTLNF
>102774_15 0.4
PQITLWQRPIVTIKIGGQLKEALLDTGADDTVLQEMNLSGRWKPKMIGGLGGFIKVRQYDQIPIEICGHKAIGTV
LIGPTPVNIIGRNLLTQLGCTLNF
>257929_0 0.3
PQITLWQRPLVTVKVGGQLKEALLDTGADDTVLEEMNLQGRWKPKMIGGIGGFIKVKQYDQIPVEICGHKAIG
TVLVGPTPVNIIGRNLLTQIGCTLNF
>206451_0 0.4
PQMTLWQRPLVTIRIGGQLKEALLDTGADDTVLEEMNLPGKWKPKMIGGIGGFIKVGQYDQIPIEICGHKAIGT
VLIGPTPVNIIGRNLLTQIGCTLNF
>45116_0 0.3
PQITLWQRPLVTIKIGGQLKEALLDTGADDTVLEEMNLPGRWKPKMIGGLGGFIKVRQYDQILIEICGHKVIGTV
LVGPTPVNIIGRNLLTQIGCTLNF
>75046_0 0.5
PQITLWQRPLVTIKIGGQLKEALLDTGADDTVLEEMNLPGRWKPKMIGGIGGFIKVRQYDQIAIEICGHKAIGTV
LVGPTPVNIIGRNLLTQIGCTLNF
>13251_0 0.4
PQITLWQRPLVTIRVGGQLKEALLDTGADDTVLEEMSLPGKWKPKMIGGIGGFIKVRQYDQVQIEICGHKAIGT
VLVGPTPVNIIGRNLLTQIGCTLNF
>13251_1 0.4
PQITLWQRPLVTIRVGGQLKEALLDTGADDTVLEEMSLPGKWKPKMIGGIGGFIKVRQYDQVQIEICGHKAIGT
VLVGPTPVNIIGRSLLTQIGCTLNF
>13251_2 0.4
PQITLWQRPLVTIRVGGQLKEALLDTGADDTVLEEMSLPGKWKPKMIGGIGGFIKVRQYDQVQIEICGHKAIGT
VLIGPTPVNIIGRNLLTQIGCTLNF
>13251_3 0.4
PQITLWQRPLVTIRVGGQLKEALLDTGADDTVLEEMSLPGKWKPKMIGGIGGFIKVRQYDQVQIEICGHKAIGT
VLIGPTPVNIIGRSLLTQIGCTLNF
>13251_4 0.4
PQITLWQRPLVTIRVGGQLKEALLDTGADDTVLEEISLPGKWKPKMIGGIGGFIKVRQYDQVQIEICGHKAIGT
VLVGPTPVNIIGRNLLTQIGCTLNF
>13251_5 0.4
PQITLWQRPLVTIRVGGQLKEALLDTGADDTVLEEISLPGKWKPKMIGGIGGFIKVRQYDQVQIEICGHKAIGT
VLVGPTPVNIIGRSLLTQIGCTLNF
>13251_6 0.4
PQITLWQRPLVTIRVGGQLKEALLDTGADDTVLEEISLPGKWKPKMIGGIGGFIKVRQYDQVQIEICGHKAIGT
VLIGPTPVNIIGRNLLTQIGCTLNF
>13251_7 0.4
PQITLWQRPLVTIRVGGQLKEALLDTGADDTVLEEISLPGKWKPKMIGGIGGFIKVRQYDQVQIEICGHKAIGT
VLIGPTPVNIIGRSLLTQIGCTLNF

>44058_0 0.2
PQITLWQRPLVTIKIGGQLKEALLDTGADDTVLEEMNLPGKWKPKMIGGIGGFIKVRQYDQITIEICGHKAIGTV
LVGPTPVNIIGRNLLTQIGCTLNF
>44058_1 0.2
PQITLWQRPLVTIRIGGQLKEALLDTGADDTVLEEMNLPGKWKPKMIGGIGGFIKVRQYDQITIEICGHKAIGTV
LVGPTPVNIIGRNLLTQIGCTLNF
>60639_0 0.4
PQITLWQRPLVNIKIGGQLKEALLDTGADDTVLEDMSLPGRWKPKMIGGIGGFIKVRQYDQIQVEICGHKAIGT
VLVGPTPVNIIGRNLLTQIGCTLNF
>75054_0 0.4
PQITLWQRPLVTIKIGGQLKEALLDTGADDTVLEEMNLPGRWKPKMIGGIGGFIKVRQYDQITIEICGHKAIGTV
LVGPTPVNIIGRNLLTQIGCTLNF
>75054_2 0.4
PQITLWQRPLVTIKIGGQLKEALLDTGADDTVLEEMNLPGRWKPKMIGGIGGFIKVRQYDQITIDICGHKAIGTV
LVGPTPVNIIGRNLLTQIGCTLNF
>75054_4 0.4
PQITLWQRPLVTIKIGGQLKEALLDTGADDTVLEEINLPGRWKPKMIGGIGGFIKVRQYDQITIEICGHKAIGTVL
VGPTPVNIIGRNLLTQIGCTLNF
>75054_6 0.4
PQITLWQRPLVTIKIGGQLKEALLDTGADDTVLEEINLPGRWKPKMIGGIGGFIKVRQYDQITIDICGHKAIGTVL
VGPTPVNIIGRNLLTQIGCTLNF
>75054_8 0.4
PQITLWQRPIVTIKIGGQLKEALLDTGADDTVLEEMNLPGRWKPKMIGGIGGFIKVRQYDQITIEICGHKAIGTV
LVGPTPVNIIGRNLLTQIGCTLNF
>75054_10 0.4
PQITLWQRPIVTIKIGGQLKEALLDTGADDTVLEEMNLPGRWKPKMIGGIGGFIKVRQYDQITIDICGHKAIGTV
LVGPTPVNIIGRNLLTQIGCTLNF
>75054_12 0.4
PQITLWQRPIVTIKIGGQLKEALLDTGADDTVLEEINLPGRWKPKMIGGIGGFIKVRQYDQITIEICGHKAIGTVL
VGPTPVNIIGRNLLTQIGCTLNF
>75054_14 0.4
PQITLWQRPIVTIKIGGQLKEALLDTGADDTVLEEINLPGRWKPKMIGGIGGFIKVRQYDQITIDICGHKAIGTVL
VGPTPVNIIGRNLLTQIGCTLNF
>75114_0 0.4
PQITLWQRPLVTIKIGGQLKEALLDTGADDTVLEDMDLPGRWKPKMIGGIGGFIKVRQYDQISIEICGHKAIGTV
LVGPTPINIIGRNLLTQIGCTLNF
>148059_0 0.5
PQITLWQRPLVTIKIGGQLKEALLDTGADDTVLEEMNLPGRWKPKMIGGIGGFIKVKQYDQIQIEICGHKAIGTV
LVGPTPVNIIGRNLLTQIGCTLNF
>148111_0 0.5
PQITLWQRPLVTVKIGGQLKEALLDTGADDTVLEEMNLPGRWKPRMIGGIGGFIKVRQYDQISMEICGHKAIG
TVLIGPTPVNIIGRNLLTQIGCTLNF
>148111_1 0.5
PQITLWQRPLVTVKIGGQLKEALLDTGADDTVLEEMNLPGRWKPRMIGGIGGFIKVRQYDQVSMEICGHKAIG
TVLIGPTPVNIIGRNLLTQIGCTLNF
>9706_0 0.4
PQITLWQRPLVTIKIGGQLKEALLDTGADDTVLEDMDLPGRWKPKMIGGIGGFIKVRQYDQILIEICGHKAIGTV
LVGPTPVNIIGRNLLTQIGCTLNF
>28226_0 0.4
PQITLWQRPLVTIKIGGQLKEALLDTGADDTVLEEMNLPGRWKPKMIGGIGGFIKVRQYDQILVEICGHKAIGTV
LVGPTPVNIIGRNLLTQMGCTLNF
>28226_1 0.4
PQITLWQRPLVTIKIGGQLKEALLDTGADDTVLEEMNLPGRWKPKMIGGIGGFIKVRQYDQILVEICGHKAIGTV
LVGPTPVNIIGRNLWTQMGCTLNF
>28226_2 0.4
PQITLWQRPLVTIKIGGQLKEALLDTGADDTVLEEMNLPGKWKPKMIGGIGGFIKVRQYDQILVEICGHKAIGTV
LVGPTPVNIIGRNLLTQMGCTLNF
>28226_3 0.4
PQITLWQRPLVTIKIGGQLKEALLDTGADDTVLEEMNLPGKWKPKMIGGIGGFIKVRQYDQILVEICGHKAIGTV
LVGPTPVNIIGRNLWTQMGCTLNF
>28226_4 0.4
PQITLWQRPLVTIKIGGQLKEALLDTGADDTVLEEMDLPGRWKPKMIGGIGGFIKVRQYDQILVEICGHKAIGTV
LVGPTPVNIIGRNLLTQMGCTLNF

>28226_5 0.4
PQITLWQRPLVTIKIGGQLKEALLDTGADDTVLEEMDLPGRWKPKMIGGIGGFIKVRQYDQILVEICGHKAIGTV
LVGPTPVNIIGRNLWTQMGCTLNF
>28226_6 0.4
PQITLWQRPLVTIKIGGQLKEALLDTGADDTVLEEMDLPGKWKPKMIGGIGGFIKVRQYDQILVEICGHKAIGTV
LVGPTPVNIIGRNLLTQMGCTLNF
>28226_7 0.4
PQITLWQRPLVTIKIGGQLKEALLDTGADDTVLEEMDLPGKWKPKMIGGIGGFIKVRQYDQILVEICGHKAIGTV
LVGPTPVNIIGRNLWTQMGCTLNF
>44074_0 0.4
PQITLWQRPLVTVKIGGQLKEALLDTGADDTVLEEMDLPGRWKPKMIGGIGGFIKVRQYDQILIEICGHRAIGT
VLVGPTPVNIIGRNLLTQIGCTLNF
>44074_1 0.4
PQITLWQRPLVTVRIGGQLKEALLDTGADDTVLEEMDLPGRWKPKMIGGIGGFIKVRQYDQILIEICGHRAIGT
VLVGPTPVNIIGRNLLTQIGCTLNF
>45114_0 0.2
PQITLWQRPLVTIKIGGQLKEALLDTGADDTVLEEMNLPGRWKPKMIGGLGGFIKVRQYDQILIEICGHKAIGTV
LVGPTPVNIIGRNLLTQIGCTLNF
>54452_0 0.4
PQITLWQRPLVTIKIGGQLTEALLDTGADDTVLEEMNLPGRWKPKMIGGIGGFIKVRQYDQILIEICGHKAIGTV
LVGPTPVNIIGRNLLTQIGCTLNF
>75126_0 0.5
PQITLWQRPLVTVRIGGQLKEALLDTGADDTVLEEMNLPGKWKPKMIGGIGGFIKVRQYDQILVEICGHKAIGT
VLVGPTPVNIIGRNLLTQIGCTLNF
>102796_0 0.2
PQITLWQRPLVTIKIGGQLKEALLDTGADDTVLEEIDLPGKWKPKMIGGLGGFIKVRQYDQILVEICGHKAIGTV
LVGPTPVNIIGRNLLTQIGCTLNF
>102796_1 0.2
PQITLWQRPLVTIKIGGQLKEALLDTGADDTVLKEIDLPGKWKPKMIGGLGGFIKVRQYDQILVEICGHKAIGTV
LVGPTPVNIIGRNLLTQIGCTLNF
>109438_0 0.5
PQITLWQRPLVTIKVGGQLKEALLDTGADDTVLEEMSLPGKWKPKMIGGIGGFIKVRQYDQILIEICGHKAIGTV
LVGPTPVNIIGRNLLTQIGCTLNF
>109438_1 0.5
PQITLWQRPLVTIKVGGQLKEALLDTGADDTVLEEMSLPGKWKPKMIGGIGGFIKVRQYDQILVEICGHKAIGT
VLVGPTPVNIIGRNLLTQIGCTLNF

Sequences for the SQV-resistant ensemble
>117051_0 1000.0
PQITLWQRPFIPVKIGGQLIEALMDTGADDTVLEDINLPGRWKPKIIGGIGGFIKVRQYDQVEIEICGHKITSPVL
VGATPVNAIGRNVMTKLGCTLNF
>98601_0 1000.0
PQITLWQRPFVTIKIGGQLIEALLDTGADDTVLEEMNLPGRWKPKIIGGIGGFMKVRQYDQIPIEICGHKVITTVL
IGPTPVNAIGRNLLTQIGCTLNF
>98604_0 1000.0
PQITLWQRPFVTIKIGGQLIEALLDTGADDTVLEEMNLPGRWKPKIIGGIGGFVKVRQYDQIPIEICGHKVISTVL
VGPTPVNAIGRNLLTQIGCTLNF
>98604_1 1000.0
PQITLWQRPFVTIKIGGQLIEALLDTGADDTVLEEINLPGRWKPKIIGGIGGFVKVRQYDQIPIEICGHKVISTVLV
GPTPVNAIGRNLLTQIGCTLNF
>123049_0 1000.0
PQITLWQRPIITIKIGGQLKDVLMDTGADDTVLEDIDLPGRWKPKLIGGIGGFVKVRQYDHVPVEVCGHKVTTT
VLVGPTPVDVIGRNLLTQIGCTLNF
>123049_1 1000.0
PQITLWQRPIITIKIGGQLKDVLMDTGADDTVLEDIDLPGRWKPKLIGGIGGFVKVRQYDNVPVEVCGHKVTTT
VLVGPTPVDVIGRNLLTQIGCTLNF
>123049_2 1000.0
PQITLWQRPIITIKIGGQLKDVLMDTGADDTVLEDIDLPGRWKPKLIGGIGGFVKVKQYDHVPVEVCGHKVTTT
VLVGPTPVDVIGRNLLTQIGCTLNF
>123049_3 1000.0
PQITLWQRPIITIKIGGQLKDVLMDTGADDTVLEDIDLPGRWKPKLIGGIGGFVKVKQYDNVPVEVCGHKVTTT
VLVGPTPVDVIGRNLLTQIGCTLNF
>123049_4 1000.0

PQITLWQRPIITIKIGGQLKDVLMDTGADDTVLEDIDLPGKWKPKLIGGIGGFVKVRQYDHVPVEVCGHKVTTT
VLVGPTPVDVIGRNLLTQIGCTLNF
>123049_5 1000.0
PQITLWQRPIITIKIGGQLKDVLMDTGADDTVLEDIDLPGKWKPKLIGGIGGFVKVRQYDNVPVEVCGHKVTTT
VLVGPTPVDVIGRNLLTQIGCTLNF
>123049_6 1000.0
PQITLWQRPIITIKIGGQLKDVLMDTGADDTVLEDIDLPGKWKPKLIGGIGGFVKVKQYDHVPVEVCGHKVTTT
VLVGPTPVDVIGRNLLTQIGCTLNF
>123049_7 1000.0
PQITLWQRPIITIKIGGQLKDVLMDTGADDTVLEDIDLPGKWKPKLIGGIGGFVKVKQYDNVPVEVCGHKVTTT
VLVGPTPVDVIGRNLLTQIGCTLNF
>123049_8 1000.0
PQITLWQRPIITIKIGGQLKDVLMDTGADDTVLENIDLPGRWKPKLIGGIGGFVKVRQYDHVPVEVCGHKVTTT
VLVGPTPVDVIGRNLLTQIGCTLNF
>123049_9 1000.0
PQITLWQRPIITIKIGGQLKDVLMDTGADDTVLENIDLPGRWKPKLIGGIGGFVKVRQYDNVPVEVCGHKVTTT
VLVGPTPVDVIGRNLLTQIGCTLNF
>123049_10 1000.0
PQITLWQRPIITIKIGGQLKDVLMDTGADDTVLENIDLPGRWKPKLIGGIGGFVKVKQYDHVPVEVCGHKVTTT
VLVGPTPVDVIGRNLLTQIGCTLNF
>123049_11 1000.0
PQITLWQRPIITIKIGGQLKDVLMDTGADDTVLENIDLPGRWKPKLIGGIGGFVKVKQYDNVPVEVCGHKVTTT
VLVGPTPVDVIGRNLLTQIGCTLNF
>123049_12 1000.0
PQITLWQRPIITIKIGGQLKDVLMDTGADDTVLENIDLPGKWKPKLIGGIGGFVKVRQYDHVPVEVCGHKVTTT
VLVGPTPVDVIGRNLLTQIGCTLNF
>123049_13 1000.0
PQITLWQRPIITIKIGGQLKDVLMDTGADDTVLENIDLPGKWKPKLIGGIGGFVKVRQYDNVPVEVCGHKVTTT
VLVGPTPVDVIGRNLLTQIGCTLNF
>123049_14 1000.0
PQITLWQRPIITIKIGGQLKDVLMDTGADDTVLENIDLPGKWKPKLIGGIGGFVKVKQYDHVPVEVCGHKVTTT
VLVGPTPVDVIGRNLLTQIGCTLNF
>123049_15 1000.0
PQITLWQRPIITIKIGGQLKDVLMDTGADDTVLENIDLPGKWKPKLIGGIGGFVKVKQYDNVPVEVCGHKVTTT
VLVGPTPVDVIGRNLLTQIGCTLNF
>147975_1 1000.0
PQITLWQRPILTVKVGGHLIEALLDTGADDTVLEGIDLPGRWKPKMIGGIGGFVKVRQYDQIPIEICGHKVLSTV
LVGPTPVNVIGRNLMTQIGCTLNF
>143332_24 1000.0
PQITLWQRPIVTVKIGGHLVEVLLDTGADDTVLEEINLPGRWKPKMIGGIGGFVKVKQYDQVPIEIWGHKVLST
VLVGPTPVNVIGRNLMTQIGCTLNF
>143332_25 1000.0
PQITLWQRPIVTVKIGGHLVEVLLDTGADDTVLEEINLPGRWKPKMIGGIGGFVKVKQYDQVPIEIWGHKVLST
VLVGPTPVNVVGRNLMTQIGCTLNF
>143332_26 1000.0
PQITLWQRPIVTVKIGGHLVEVLLDTGADDTVLEEINLPGRWKPKMIGGIGGFVRVKQYDQVPIEIWGHKVLST
VLVGPTPVNVIGRNLMTQIGCTLNF
>143332_27 1000.0
PQITLWQRPIVTVKIGGHLVEVLLDTGADDTVLEEINLPGRWKPKMIGGIGGFVRVKQYDQVPIEIWGHKVLST
VLVGPTPVNVVGRNLMTQIGCTLNF
>143332_28 1000.0
PQITLWQRPIVTVKIGGHLVEVLLDTGADDTVLEEINLPGRWKPKIIGGIGGFVKVKQYDQVPIEIWGHKVLSTV
LVGPTPVNVIGRNLMTQIGCTLNF
>143332_29 1000.0
PQITLWQRPIVTVKIGGHLVEVLLDTGADDTVLEEINLPGRWKPKIIGGIGGFVKVKQYDQVPIEIWGHKVLSTV
LVGPTPVNVVGRNLMTQIGCTLNF
>143332_30 1000.0
PQITLWQRPIVTVKIGGHLVEVLLDTGADDTVLEEINLPGRWKPKIIGGIGGFVRVKQYDQVPIEIWGHKVLSTV
LVGPTPVNVIGRNLMTQIGCTLNF
>143332_31 1000.0
PQITLWQRPIVTVKIGGHLVEVLLDTGADDTVLEEINLPGRWKPKIIGGIGGFVRVKQYDQVPIEIWGHKVLSTV
LVGPTPVNVVGRNLMTQIGCTLNF
>143332_32 1000.0

PQITLWQRPIVTVKIGGHLVEVLLDTGADDTVIEEINLPGRWKPKMIGGIGGFVKVKQYDQVPIEIWGHKVLST
VLVGPTPVNVIGRNLMTQIGCTLNF
>143332_33 1000.0
PQITLWQRPIVTVKIGGHLVEVLLDTGADDTVIEEINLPGRWKPKMIGGIGGFVKVKQYDQVPIEIWGHKVLST
VLVGPTPVNVVGRNLMTQIGCTLNF
>143332_34 1000.0
PQITLWQRPIVTVKIGGHLVEVLLDTGADDTVIEEINLPGRWKPKMIGGIGGFVRVKQYDQVPIEIWGHKVLST
VLVGPTPVNVIGRNLMTQIGCTLNF
>143332_35 1000.0
PQITLWQRPIVTVKIGGHLVEVLLDTGADDTVIEEINLPGRWKPKMIGGIGGFVRVKQYDQVPIEIWGHKVLST
VLVGPTPVNVVGRNLMTQIGCTLNF
>143332_36 1000.0
PQITLWQRPIVTVKIGGHLVEVLLDTGADDTVIEEINLPGRWKPKIIGGIGGFVKVKQYDQVPIEIWGHKVLSTV
LVGPTPVNVIGRNLMTQIGCTLNF
>143332_37 1000.0
PQITLWQRPIVTVKIGGHLVEVLLDTGADDTVIEEINLPGRWKPKIIGGIGGFVKVKQYDQVPIEIWGHKVLSTV
LVGPTPVNVVGRNLMTQIGCTLNF
>143332_38 1000.0
PQITLWQRPIVTVKIGGHLVEVLLDTGADDTVIEEINLPGRWKPKIIGGIGGFVRVKQYDQVPIEIWGHKVLSTV
LVGPTPVNVIGRNLMTQIGCTLNF
>143332_39 1000.0
PQITLWQRPIVTVKIGGHLVEVLLDTGADDTVIEEINLPGRWKPKIIGGIGGFVRVKQYDQVPIEIWGHKVLSTV
LVGPTPVNVVGRNLMTQIGCTLNF
>143332_40 1000.0
PQITLWQRPIVTVKIGGHLVEVLLDTGADDTVVEEINLPGRWKPKMIGGIGGFVKVKQYDQVPIEIWGHKVLST
VLVGPTPVNVIGRNLMTQIGCTLNF
>143332_41 1000.0
PQITLWQRPIVTVKIGGHLVEVLLDTGADDTVVEEINLPGRWKPKMIGGIGGFVKVKQYDQVPIEIWGHKVLST
VLVGPTPVNVVGRNLMTQIGCTLNF
>143332_42 1000.0
PQITLWQRPIVTVKIGGHLVEVLLDTGADDTVVEEINLPGRWKPKMIGGIGGFVRVKQYDQVPIEIWGHKVLST
VLVGPTPVNVIGRNLMTQIGCTLNF
>143332_43 1000.0
PQITLWQRPIVTVKIGGHLVEVLLDTGADDTVVEEINLPGRWKPKMIGGIGGFVRVKQYDQVPIEIWGHKVLST
VLVGPTPVNVVGRNLMTQIGCTLNF
>143332_44 1000.0
PQITLWQRPIVTVKIGGHLVEVLLDTGADDTVVEEINLPGRWKPKIIGGIGGFVKVKQYDQVPIEIWGHKVLSTV
LVGPTPVNVIGRNLMTQIGCTLNF
>143332_45 1000.0
PQITLWQRPIVTVKIGGHLVEVLLDTGADDTVVEEINLPGRWKPKIIGGIGGFVKVKQYDQVPIEIWGHKVLSTV
LVGPTPVNVVGRNLMTQIGCTLNF
>143332_46 1000.0
PQITLWQRPIVTVKIGGHLVEVLLDTGADDTVVEEINLPGRWKPKIIGGIGGFVRVKQYDQVPIEIWGHKVLSTV
LVGPTPVNVIGRNLMTQIGCTLNF
>143332_47 1000.0
PQITLWQRPIVTVKIGGHLVEVLLDTGADDTVVEEINLPGRWKPKIIGGIGGFVRVKQYDQVPIEIWGHKVLSTV
LVGPTPVNVVGRNLMTQIGCTLNF
>143332_72 1000.0
PQITLWQRPIVTVKVGGHLVEVLLDTGADDTVLEEINLPGRWKPKMIGGIGGFVKVKQYDQVPIEIWGHKVLS
TVLVGPTPVNVIGRNLMTQIGCTLNF
>143473_26 1000.0
PQITLWQRPIVIVKIGGQIREALLDTGADDTVFEDIDLPGRWTPKLIGGIGGFVKVRQYDQVPIEICGHKVISTVL
VGPTPANIIGRNLMTQLGCTLNF
>143473_30 1000.0
PQITLWQRPIVIVKIGGQIREALLDTGADDTVFEDIDLPGRWTPKLIGGIGGFVRVRQYDQVPIEICGHKVISTVL
VGPTPANIIGRNLMTQLGCTLNF
>113050_0 1000.0
PQITLWQRPFVTVKIGGQITEALLDTGADDTILENINLPGRWKPKIIQGIGGFLKVRQYDQIPIEIEGHKVIGSVV
VGPTPVNVIGRNVMSQLGCTLNF
>117139_0 1000.0
PQITLWQRPIVTIKIGGQLKEALLDTGADDTVLEEMDLPGRWKPKMIVGIGGLVKVRQYDQIPIEICGHKTIGTV
LIGPTPANIIGRNLMTQLGCTLNF
>113192_1 1000.0

PQITLWQRPFVTMKIGGQLIEALLDTGADDTVLEEISLPGRWKPKIIGGIGGFVKVRQYDQVPIEVFGHKATTT
VLVGPTPVNVIGRNLMTQLGCTLNF
>113192_3 1000.0
PQITLWQRPFVTMKIGGQLIEALLDTGADDTVLEEISLPGRWKPKIIGGIGGFVKVRQYDQVPIEVFGQKATTT
VLVGPTPVNVIGRNLMTQLGCTLNF
>113192_5 1000.0
PQITLWQRPFVTMKIGGQLIEALLDTGADDTVLEEISLPGRWKPKIIGGIGGFVKVRQYDQVPVEVFGHKATTT
VLVGPTPVNVIGRNLMTQLGCTLNF
>113192_7 1000.0
PQITLWQRPFVTMKIGGQLIEALLDTGADDTVLEEISLPGRWKPKIIGGIGGFVKVRQYDQVPVEVFGQKATTT
VLVGPTPVNVIGRNLMTQLGCTLNF
>113192_9 1000.0
PQITLWQRPFVTMKIGGQIIEALLDTGADDTVLEEISLPGRWKPKIIGGIGGFVKVRQYDQVPIEVFGHKATTTV
LVGPTPVNVIGRNLMTQLGCTLNF
>113192_11 1000.0
PQITLWQRPFVTMKIGGQIIEALLDTGADDTVLEEISLPGRWKPKIIGGIGGFVKVRQYDQVPIEVFGQKATTTV
LVGPTPVNVIGRNLMTQLGCTLNF
>113192_13 1000.0
PQITLWQRPFVTMKIGGQIIEALLDTGADDTVLEEISLPGRWKPKIIGGIGGFVKVRQYDQVPVEVFGHKATTT
VLVGPTPVNVIGRNLMTQLGCTLNF
>113192_15 1000.0
PQITLWQRPFVTMKIGGQIIEALLDTGADDTVLEEISLPGRWKPKIIGGIGGFVKVRQYDQVPVEVFGQKATTT
VLVGPTPVNVIGRNLMTQLGCTLNF
>147991_0 1000.0
PQITLWQRPIVTVKIGGQLKEALLDTGADDTVIEGINLPGKWKPKMIGGIGGFVKVREYEQIPIEICGHKTISTVL
VGPTPVNVIGRNLMTQLGCTLNF
>147991_1 1000.0
PQITLWQRPIVTVKIGGQLKEALLDTGADDTVIEGINLPGKWKPKMIGGIGGFVKVREYEQIPIEICGHKTVSTV
LVGPTPVNVIGRNLMTQLGCTLNF
>147991_2 1000.0
PQITLWQRPIVTVKIGGQLKEALLDTGADDTVIEGINLPGKWKPKMIGGIGGFVKVKEYEQIPIEICGHKTISTVL
VGPTPVNVIGRNLMTQLGCTLNF
>147991_3 1000.0
PQITLWQRPIVTVKIGGQLKEALLDTGADDTVIEGINLPGKWKPKMIGGIGGFVKVKEYEQIPIEICGHKTVSTV
LVGPTPVNVIGRNLMTQLGCTLNF
>147991_4 1000.0
PQITLWQRPIVTVKIGGQLKEALLDTGADDTVIEGINLPGKWKPKMIGGIGGFVRVREYEQIPIEICGHKTISTVL
VGPTPVNVIGRNLMTQLGCTLNF
>147991_5 1000.0
PQITLWQRPIVTVKIGGQLKEALLDTGADDTVIEGINLPGKWKPKMIGGIGGFVRVREYEQIPIEICGHKTVSTV
LVGPTPVNVIGRNLMTQLGCTLNF
>147991_6 1000.0
PQITLWQRPIVTVKIGGQLKEALLDTGADDTVIEGINLPGKWKPKMIGGIGGFVRVKEYEQIPIEICGHKTISTVL
VGPTPVNVIGRNLMTQLGCTLNF
>147991_7 1000.0
PQITLWQRPIVTVKIGGQLKEALLDTGADDTVIEGINLPGKWKPKMIGGIGGFVRVKEYEQIPIEICGHKTVSTV
LVGPTPVNVIGRNLMTQLGCTLNF
>147991_8 1000.0
PQITLWQRPIVTVKIGGQLKEALLDTGADDTVIEGINLPGKWKPKIIGGIGGFVKVREYEQIPIEICGHKTISTVLV
GPTPVNVIGRNLMTQLGCTLNF
>147991_9 1000.0
PQITLWQRPIVTVKIGGQLKEALLDTGADDTVIEGINLPGKWKPKIIGGIGGFVKVREYEQIPIEICGHKTVSTVL
VGPTPVNVIGRNLMTQLGCTLNF
>147991_10 1000.0
PQITLWQRPIVTVKIGGQLKEALLDTGADDTVIEGINLPGKWKPKIIGGIGGFVKVKEYEQIPIEICGHKTISTVLV
GPTPVNVIGRNLMTQLGCTLNF
>147991_11 1000.0
PQITLWQRPIVTVKIGGQLKEALLDTGADDTVIEGINLPGKWKPKIIGGIGGFVKVKEYEQIPIEICGHKTVSTVL
VGPTPVNVIGRNLMTQLGCTLNF
>147991_12 1000.0
PQITLWQRPIVTVKIGGQLKEALLDTGADDTVIEGINLPGKWKPKIIGGIGGFVRVREYEQIPIEICGHKTISTVLV
GPTPVNVIGRNLMTQLGCTLNF
>147991_13 1000.0

PQITLWQRPIVTVKIGGQLKEALLDTGADDTVIEGINLPGKWKPKIIGGIGGFVRVREYEQIPIEICGHKTVSTVL
VGPTPVNVIGRNLMTQLGCTLNF
>147991_14 1000.0
PQITLWQRPIVTVKIGGQLKEALLDTGADDTVIEGINLPGKWKPKIIGGIGGFVRVKEYEQIPIEICGHKTISTVLV
GPTPVNVIGRNLMTQLGCTLNF
>147991_15 1000.0
PQITLWQRPIVTVKIGGQLKEALLDTGADDTVIEGINLPGKWKPKIIGGIGGFVRVKEYEQIPIEICGHKTVSTVL
VGPTPVNVIGRNLMTQLGCTLNF
>143473_1 1000.0
PQITLWQRPIVTVKIGGQLREALLDTGADDTVFEDIDLPGRWTPKLIGGIGGFVKVRQYDQVPIEICGHKITSTV
LVGPTPANIIGRNLMTQLGCTLNF
>187151_2 1000.0
PQITLWQRPIITVKIGGHLIEALLDTGADDTVLEEMNLPGRWKPKIIGGIGGFLKVRQYDQIPIEICGHKVMGTVL
VGPTPVNVIGRNLMTQIGCTLNF
>117041_0 1000.0
PQITLWQRPIVTIKVGGQLKEALLDTGADDTVFEEMNLPGRWKPKIIGGIGGFMKVRQYDQVPIEFFGHKALTT
ILVGPTPVNVIGRNLMTQIGVTLNF
>117041_1 1000.0
PQITLWQRPIVTIKVGGQLKEALLDTGADDTVFEEMNLPGRWKPKIIGGIGGFMKVRQYDQVPIEFFGHKALTT
ILVGPTPVNVIGRNLMTQMGVTLNF
>117041_2 1000.0
PQITLWQRPIVTIKVGGQLKEALLDTGADDTVFEEMNLPGRWKPKIIGGIGGFVKVRQYDQVPIEFFGHKALTT
ILVGPTPVNVIGRNLMTQIGVTLNF
>117041_3 1000.0
PQITLWQRPIVTIKVGGQLKEALLDTGADDTVFEEMNLPGRWKPKIIGGIGGFVKVRQYDQVPIEFFGHKALTT
ILVGPTPVNVIGRNLMTQMGVTLNF
>117041_4 1000.0
PQITLWQRPIVTIKVGGQLKEALLDTGADDTVFEEMDLPGRWKPKIIGGIGGFMKVRQYDQVPIEFFGHKALTT
ILVGPTPVNVIGRNLMTQIGVTLNF
>117041_5 1000.0
PQITLWQRPIVTIKVGGQLKEALLDTGADDTVFEEMDLPGRWKPKIIGGIGGFMKVRQYDQVPIEFFGHKALTT
ILVGPTPVNVIGRNLMTQMGVTLNF
>117041_6 1000.0
PQITLWQRPIVTIKVGGQLKEALLDTGADDTVFEEMDLPGRWKPKIIGGIGGFVKVRQYDQVPIEFFGHKALTT
ILVGPTPVNVIGRNLMTQIGVTLNF
>117041_7 1000.0
PQITLWQRPIVTIKVGGQLKEALLDTGADDTVFEEMDLPGRWKPKIIGGIGGFVKVRQYDQVPIEFFGHKALTT
ILVGPTPVNVIGRNLMTQMGVTLNF
>117041_8 1000.0
PQITLWQRPIVTIKVGGQLKEALLDTGADDTVFEEVNLPGRWKPKIIGGIGGFMKVRQYDQVPIEFFGHKALTT
ILVGPTPVNVIGRNLMTQIGVTLNF
>117041_9 1000.0
PQITLWQRPIVTIKVGGQLKEALLDTGADDTVFEEVNLPGRWKPKIIGGIGGFMKVRQYDQVPIEFFGHKALTT
ILVGPTPVNVIGRNLMTQMGVTLNF
>117041_10 1000.0
PQITLWQRPIVTIKVGGQLKEALLDTGADDTVFEEVNLPGRWKPKIIGGIGGFVKVRQYDQVPIEFFGHKALTTI
LVGPTPVNVIGRNLMTQIGVTLNF
>117041_11 1000.0
PQITLWQRPIVTIKVGGQLKEALLDTGADDTVFEEVNLPGRWKPKIIGGIGGFVKVRQYDQVPIEFFGHKALTTI
LVGPTPVNVIGRNLMTQMGVTLNF
>117041_12 1000.0
PQITLWQRPIVTIKVGGQLKEALLDTGADDTVFEEVDLPGRWKPKIIGGIGGFMKVRQYDQVPIEFFGHKALTT
ILVGPTPVNVIGRNLMTQIGVTLNF
>117041_13 1000.0
PQITLWQRPIVTIKVGGQLKEALLDTGADDTVFEEVDLPGRWKPKIIGGIGGFMKVRQYDQVPIEFFGHKALTT
ILVGPTPVNVIGRNLMTQMGVTLNF
>117041_14 1000.0
PQITLWQRPIVTIKVGGQLKEALLDTGADDTVFEEVDLPGRWKPKIIGGIGGFVKVRQYDQVPIEFFGHKALTTI
LVGPTPVNVIGRNLMTQIGVTLNF
>117041_15 1000.0
PQITLWQRPIVTIKVGGQLKEALLDTGADDTVFEEVDLPGRWKPKIIGGIGGFVKVRQYDQVPIEFFGHKALTTI
LVGPTPVNVIGRNLMTQMGVTLNF
>117041_16 1000.0

PQITLWQRPIVTIKVGGQLKEALLDTGADDTVFDEMNLPGRWKPKIIGGIGGFMKVRQYDQVPIEFFGHKALT
TILVGPTPVNVIGRNLMTQIGVTLNF
>117041_17 1000.0
PQITLWQRPIVTIKVGGQLKEALLDTGADDTVFDEMNLPGRWKPKIIGGIGGFMKVRQYDQVPIEFFGHKALT
TILVGPTPVNVIGRNLMTQMGVTLNF
>117041_18 1000.0
PQITLWQRPIVTIKVGGQLKEALLDTGADDTVFDEMNLPGRWKPKIIGGIGGFVKVRQYDQVPIEFFGHKALTT
ILVGPTPVNVIGRNLMTQIGVTLNF
>117041_19 1000.0
PQITLWQRPIVTIKVGGQLKEALLDTGADDTVFDEMNLPGRWKPKIIGGIGGFVKVRQYDQVPIEFFGHKALTT
ILVGPTPVNVIGRNLMTQMGVTLNF
>117041_20 1000.0
PQITLWQRPIVTIKVGGQLKEALLDTGADDTVFDEMDLPGRWKPKIIGGIGGFMKVRQYDQVPIEFFGHKALT
TILVGPTPVNVIGRNLMTQIGVTLNF
>117041_21 1000.0
PQITLWQRPIVTIKVGGQLKEALLDTGADDTVFDEMDLPGRWKPKIIGGIGGFMKVRQYDQVPIEFFGHKALT
TILVGPTPVNVIGRNLMTQMGVTLNF
>117041_22 1000.0
PQITLWQRPIVTIKVGGQLKEALLDTGADDTVFDEMDLPGRWKPKIIGGIGGFVKVRQYDQVPIEFFGHKALTT
ILVGPTPVNVIGRNLMTQIGVTLNF
>117041_23 1000.0
PQITLWQRPIVTIKVGGQLKEALLDTGADDTVFDEMDLPGRWKPKIIGGIGGFVKVRQYDQVPIEFFGHKALTT
ILVGPTPVNVIGRNLMTQMGVTLNF

Sequences for the SQV-susceptible ensemble
>102782_40 0.3
PQITLWQRPIVTIKVEGQLKEAPLDTGADDTVLEDVNLPGRWKPKMIGGLGGFIKVRQYDQIHIEICGHKAIGT
VLVGPTPVNIIGRNMLTQIGCTLNF
>102782_41 0.3
PQITLWQRPIVTIKVEGQLKEAPLDTGADDTVLEDVNLPGRWKPKMIGGLGGFIKVRQYDQIHIEICGHKAIGT
VLVGPTPVNIIGRSMLTQIGCTLNF
>102782_42 0.3
PQITLWQRPIVTIKVEGQLKEAPLDTGADDTVLEDVNLPGRWKPKMIGGLGGFIKVRQYDQIHIEICGHKAIGT
VLIGPTPVNIIGRNMLTQIGCTLNF
>102782_43 0.3
PQITLWQRPIVTIKVEGQLKEAPLDTGADDTVLEDVNLPGRWKPKMIGGLGGFIKVRQYDQIHIEICGHKAIGT
VLIGPTPVNIIGRSMLTQIGCTLNF
>13246_1 0.1
PQITLWQRPLVTIKIGGQLKEALLDTGADDTVLEEMNLPGRWKPKMIGGIGGFIKVRQYDQISVEICGHKATGT
VLIGPTPTNIIGRNLLTQIGCTLNF
>13246_3 0.1
PQITLWQRPLVTIKIGGQLKEALLDTGADDTVLEEMNLPGRWKPKIIGGIGGFIKVRQYDQISVEICGHKATGTV
LIGPTPTNIIGRNLLTQIGCTLNF
>13246_5 0.1
PQITLWQRPLVTIKIGGQLKEALLDTGADDTVLEELNLPGRWKPKMIGGIGGFIKVRQYDQISVEICGHKATGT
VLIGPTPTNIIGRNLLTQIGCTLNF
>13246_7 0.1
PQITLWQRPLVTIKIGGQLKEALLDTGADDTVLEELNLPGRWKPKIIGGIGGFIKVRQYDQISVEICGHKATGTV
LIGPTPTNIIGRNLLTQIGCTLNF
>13246_9 0.1
PQITLWQRPFVTIKIGGQLKEALLDTGADDTVLEEMNLPGRWKPKMIGGIGGFIKVRQYDQISVEICGHKATGT
VLIGPTPTNIIGRNLLTQIGCTLNF
>13246_11 0.1
PQITLWQRPFVTIKIGGQLKEALLDTGADDTVLEEMNLPGRWKPKIIGGIGGFIKVRQYDQISVEICGHKATGTV
LIGPTPTNIIGRNLLTQIGCTLNF
>13246_13 0.1
PQITLWQRPFVTIKIGGQLKEALLDTGADDTVLEELNLPGRWKPKMIGGIGGFIKVRQYDQISVEICGHKATGT
VLIGPTPTNIIGRNLLTQIGCTLNF
>13246_15 0.1
PQITLWQRPFVTIKIGGQLKEALLDTGADDTVLEELNLPGRWKPKIIGGIGGFIKVRQYDQISVEICGHKATGTV
LIGPTPTNIIGRNLLTQIGCTLNF
>13246_0 0.1
PQITLWQRPLVTIKIGGQLKEALLDTGADDTVLEEMNLPGRWKPKMIGGIGGFIKVRQYDQISVEICGHKATGT

VLIGPTPANIIGRNLLTQIGCTLNF
>13246_2 0.1
PQITLWQRPLVTIKIGGQLKEALLDTGADDTVLEEMNLPGRWKPKIIGGIGGFIKVRQYDQISVEICGHKATGTV
LIGPTPANIIGRNLLTQIGCTLNF
>13246_4 0.1
PQITLWQRPLVTIKIGGQLKEALLDTGADDTVLEELNLPGRWKPKMIGGIGGFIKVRQYDQISVEICGHKATGT
VLIGPTPANIIGRNLLTQIGCTLNF
>13246_6 0.1
PQITLWQRPLVTIKIGGQLKEALLDTGADDTVLEELNLPGRWKPKIIGGIGGFIKVRQYDQISVEICGHKATGTV
LIGPTPANIIGRNLLTQIGCTLNF
>13246_8 0.1
PQITLWQRPFVTIKIGGQLKEALLDTGADDTVLEEMNLPGRWKPKMIGGIGGFIKVRQYDQISVEICGHKATGT
VLIGPTPANIIGRNLLTQIGCTLNF
>13246_10 0.1
PQITLWQRPFVTIKIGGQLKEALLDTGADDTVLEEMNLPGRWKPKIIGGIGGFIKVRQYDQISVEICGHKATGTV
LIGPTPANIIGRNLLTQIGCTLNF
>13246_12 0.1
PQITLWQRPFVTIKIGGQLKEALLDTGADDTVLEELNLPGRWKPKMIGGIGGFIKVRQYDQISVEICGHKATGT
VLIGPTPANIIGRNLLTQIGCTLNF
>13246_14 0.1
PQITLWQRPFVTIKIGGQLKEALLDTGADDTVLEELNLPGRWKPKIIGGIGGFIKVRQYDQISVEICGHKATGTV
LIGPTPANIIGRNLLTQIGCTLNF
>55835_1 0.2
PQITLWQRPLVPIKIGGQIKEALLDTGADDTILEDINLPGRWKPKMAGGIGGFIKVRQYDQIPVEICGHKVIGTVL
VGPTPVNIIGRNLLTQLGCTLNF
>55835_3 0.2
PQITLWQRPLVPIKIGGQIKEALLDTGADDTILEDINLPGRWKPKMAGGIGGFIKVRQYDHIPVEICGHKVIGTVL
VGPTPVNIIGRNLLTQLGCTLNF
>55835_5 0.2
PQITLWQRPLVPIKIGGQIKEALLDTGADDTILEDINLPGRWKPKLAGGIGGFIKVRQYDQIPVEICGHKVIGTVL
VGPTPVNIIGRNLLTQLGCTLNF
>55835_7 0.2
PQITLWQRPLVPIKIGGQIKEALLDTGADDTILEDINLPGRWKPKLAGGIGGFIKVRQYDHIPVEICGHKVIGTVL
VGPTPVNIIGRNLLTQLGCTLNF
>55835_9 0.2
PQITLWQRPLVPIKIGGQIKEALLDTGADDTILEDINLPGKWKPKMAGGIGGFIKVRQYDQIPVEICGHKVIGTVL
VGPTPVNIIGRNLLTQLGCTLNF
>55835_11 0.2
PQITLWQRPLVPIKIGGQIKEALLDTGADDTILEDINLPGKWKPKMAGGIGGFIKVRQYDHIPVEICGHKVIGTVL
VGPTPVNIIGRNLLTQLGCTLNF
>55835_13 0.2
PQITLWQRPLVPIKIGGQIKEALLDTGADDTILEDINLPGKWKPKLAGGIGGFIKVRQYDQIPVEICGHKVIGTVL
VGPTPVNIIGRNLLTQLGCTLNF
>55835_15 0.2
PQITLWQRPLVPIKIGGQIKEALLDTGADDTILEDINLPGKWKPKLAGGIGGFIKVRQYDHIPVEICGHKVIGTVL
VGPTPVNIIGRNLLTQLGCTLNF
>55835_33 0.2
PQITLWQRPIVPIKIGGQIKEALLDTGADDTILEDINLPGRWKPKMAGGIGGFIKVRQYDQIPVEICGHKVIGTVL
VGPTPVNIIGRNLLTQLGCTLNF
>55835_35 0.2
PQITLWQRPIVPIKIGGQIKEALLDTGADDTILEDINLPGRWKPKMAGGIGGFIKVRQYDHIPVEICGHKVIGTVL
VGPTPVNIIGRNLLTQLGCTLNF
>55835_37 0.2
PQITLWQRPIVPIKIGGQIKEALLDTGADDTILEDINLPGRWKPKLAGGIGGFIKVRQYDQIPVEICGHKVIGTVL
VGPTPVNIIGRNLLTQLGCTLNF
>55835_39 0.2
PQITLWQRPIVPIKIGGQIKEALLDTGADDTILEDINLPGRWKPKLAGGIGGFIKVRQYDHIPVEICGHKVIGTVL
VGPTPVNIIGRNLLTQLGCTLNF
>55835_41 0.2
PQITLWQRPIVPIKIGGQIKEALLDTGADDTILEDINLPGKWKPKMAGGIGGFIKVRQYDQIPVEICGHKVIGTVL
VGPTPVNIIGRNLLTQLGCTLNF
>55835_43 0.2
PQITLWQRPIVPIKIGGQIKEALLDTGADDTILEDINLPGKWKPKMAGGIGGFIKVRQYDHIPVEICGHKVIGTVL

VGPTPVNIIGRNLLTQLGCTLNF
>55835_45 0.2
PQITLWQRPIVPIKIGGQIKEALLDTGADDTILEDINLPGKWKPKLAGGIGGFIKVRQYDQIPVEICGHKVIGTVL
VGPTPVNIIGRNLLTQLGCTLNF
>55835_47 0.2
PQITLWQRPIVPIKIGGQIKEALLDTGADDTILEDINLPGKWKPKLAGGIGGFIKVRQYDHIPVEICGHKVIGTVLV
GPTPVNIIGRNLLTQLGCTLNF
>55835_0 0.2
PQITLWQRPLVPIKIGGQIKEALLDTGADDTILEDINLPGRWKPKMAGGIGGFIKVRQYDQIPVEICGHKAIGTVL
VGPTPVNIIGRNLLTQLGCTLNF
>55835_2 0.2
PQITLWQRPLVPIKIGGQIKEALLDTGADDTILEDINLPGRWKPKMAGGIGGFIKVRQYDHIPVEICGHKAIGTVL
VGPTPVNIIGRNLLTQLGCTLNF
>55835_4 0.2
PQITLWQRPLVPIKIGGQIKEALLDTGADDTILEDINLPGRWKPKLAGGIGGFIKVRQYDQIPVEICGHKAIGTVL
VGPTPVNIIGRNLLTQLGCTLNF
>55835_6 0.2
PQITLWQRPLVPIKIGGQIKEALLDTGADDTILEDINLPGRWKPKLAGGIGGFIKVRQYDHIPVEICGHKAIGTVL
VGPTPVNIIGRNLLTQLGCTLNF
>55835_8 0.2
PQITLWQRPLVPIKIGGQIKEALLDTGADDTILEDINLPGKWKPKMAGGIGGFIKVRQYDQIPVEICGHKAIGTVL
VGPTPVNIIGRNLLTQLGCTLNF
>55835_10 0.2
PQITLWQRPLVPIKIGGQIKEALLDTGADDTILEDINLPGKWKPKMAGGIGGFIKVRQYDHIPVEICGHKAIGTVL
VGPTPVNIIGRNLLTQLGCTLNF
>55835_12 0.2
PQITLWQRPLVPIKIGGQIKEALLDTGADDTILEDINLPGKWKPKLAGGIGGFIKVRQYDQIPVEICGHKAIGTVL
VGPTPVNIIGRNLLTQLGCTLNF
>55835_14 0.2
PQITLWQRPLVPIKIGGQIKEALLDTGADDTILEDINLPGKWKPKLAGGIGGFIKVRQYDHIPVEICGHKAIGTVL
VGPTPVNIIGRNLLTQLGCTLNF
>55835_32 0.2
PQITLWQRPIVPIKIGGQIKEALLDTGADDTILEDINLPGRWKPKMAGGIGGFIKVRQYDQIPVEICGHKAIGTVL
VGPTPVNIIGRNLLTQLGCTLNF
>55835_34 0.2
PQITLWQRPIVPIKIGGQIKEALLDTGADDTILEDINLPGRWKPKMAGGIGGFIKVRQYDHIPVEICGHKAIGTVL
VGPTPVNIIGRNLLTQLGCTLNF
>55835_36 0.2
PQITLWQRPIVPIKIGGQIKEALLDTGADDTILEDINLPGRWKPKLAGGIGGFIKVRQYDQIPVEICGHKAIGTVL
VGPTPVNIIGRNLLTQLGCTLNF
>55835_38 0.2
PQITLWQRPIVPIKIGGQIKEALLDTGADDTILEDINLPGRWKPKLAGGIGGFIKVRQYDHIPVEICGHKAIGTVL
VGPTPVNIIGRNLLTQLGCTLNF
>55835_40 0.2
PQITLWQRPIVPIKIGGQIKEALLDTGADDTILEDINLPGKWKPKMAGGIGGFIKVRQYDQIPVEICGHKAIGTVL
VGPTPVNIIGRNLLTQLGCTLNF
>55835_42 0.2
PQITLWQRPIVPIKIGGQIKEALLDTGADDTILEDINLPGKWKPKMAGGIGGFIKVRQYDHIPVEICGHKAIGTVL
VGPTPVNIIGRNLLTQLGCTLNF
>55835_44 0.2
PQITLWQRPIVPIKIGGQIKEALLDTGADDTILEDINLPGKWKPKLAGGIGGFIKVRQYDQIPVEICGHKAIGTVL
VGPTPVNIIGRNLLTQLGCTLNF
>55835_46 0.2
PQITLWQRPIVPIKIGGQIKEALLDTGADDTILEDINLPGKWKPKLAGGIGGFIKVRQYDHIPVEICGHKAIGTVLV
GPTPVNIIGRNLLTQLGCTLNF
>68210_0 0.2
PQITLWQRPLVSVKVGGQIKEALLDTGADDTILEEMNLPGRWKPKMAGGIGGFIKVRQYDNILIEICGHKAIGT
VLVGPTPVNIIGRNLLTQIGCTLNF
>68210_1 0.2
PQITLWQRPLVSVKVGGQIKEALLDTGADDTILEEMNLPGRWKPKIAGGIGGFIKVRQYDNILIEICGHKAIGTV
LVGPTPVNIIGRNLLTQIGCTLNF
>102782_3 0.3
PQITLWQRPIVTIKVEGQLKEALLDTGADDTVLEDMNLPGRWKPKMIGGLGGFIKVRQYDQIHIEICGHKAIGT

VLIGPTPVNIIGRSMLTQIGCTLNF
>102782_8 0.3
PQITLWQRPIVTIKVEGQLKEALLDTGADDTVLEDVNLPGRWKPKMIGGLGGFIKVRQYDQIHIEICGHKAIGT
VLVGPTPVNIIGRNMLTQIGCTLNF
>102782_9 0.3
PQITLWQRPIVTIKVEGQLKEALLDTGADDTVLEDVNLPGRWKPKMIGGLGGFIKVRQYDQIHIEICGHKAIGT
VLVGPTPVNIIGRSMLTQIGCTLNF
>102782_10 0.3
PQITLWQRPIVTIKVEGQLKEALLDTGADDTVLEDVNLPGRWKPKMIGGLGGFIKVRQYDQIHIEICGHKAIGT
VLIGPTPVNIIGRNMLTQIGCTLNF
>102782_11 0.3
PQITLWQRPIVTIKVEGQLKEALLDTGADDTVLEDVNLPGRWKPKMIGGLGGFIKVRQYDQIHIEICGHKAIGT
VLIGPTPVNIIGRSMLTQIGCTLNF
>102782_136 0.3
PQITLWQRPIVTIKVEGQLTEALLDTGADDTVLEDVNLPGRWKPKMIGGLGGFIKVRQYDQIHIEICGHKAIGTV
LVGPTPVNIIGRNMLTQIGCTLNF
>102782_137 0.3
PQITLWQRPIVTIKVEGQLTEALLDTGADDTVLEDVNLPGRWKPKMIGGLGGFIKVRQYDQIHIEICGHKAIGTV
LVGPTPVNIIGRSMLTQIGCTLNF
>102782_138 0.3
PQITLWQRPIVTIKVEGQLTEALLDTGADDTVLEDVNLPGRWKPKMIGGLGGFIKVRQYDQIHIEICGHKAIGTV
LIGPTPVNIIGRNMLTQIGCTLNF
>102782_139 0.3
PQITLWQRPIVTIKVEGQLTEALLDTGADDTVLEDVNLPGRWKPKMIGGLGGFIKVRQYDQIHIEICGHKAIGTV
LIGPTPVNIIGRSMLTQIGCTLNF
>102782_144 0.3
PQITLWQRPIVTIKVEGQLTEALLDTGADDTVFEDMNLPGRWKPKMIGGLGGFIKVRQYDQIHIEICGHKAIGT
VLVGPTPVNIIGRNMLTQIGCTLNF
>55835_17 0.2
PQITLWQRPLVSIKIGGQIKEALLDTGADDTILEDINLPGRWKPKMAGGIGGFIKVRQYDQIPVEICGHKVIGTVL
VGPTPVNIIGRNLLTQLGCTLNF
>55835_19 0.2
PQITLWQRPLVSIKIGGQIKEALLDTGADDTILEDINLPGRWKPKMAGGIGGFIKVRQYDHIPVEICGHKVIGTVL
VGPTPVNIIGRNLLTQLGCTLNF
>55835_21 0.2
PQITLWQRPLVSIKIGGQIKEALLDTGADDTILEDINLPGRWKPKLAGGIGGFIKVRQYDQIPVEICGHKVIGTVL
VGPTPVNIIGRNLLTQLGCTLNF
>55835_23 0.2
PQITLWQRPLVSIKIGGQIKEALLDTGADDTILEDINLPGRWKPKLAGGIGGFIKVRQYDHIPVEICGHKVIGTVL
VGPTPVNIIGRNLLTQLGCTLNF
>55835_25 0.2
PQITLWQRPLVSIKIGGQIKEALLDTGADDTILEDINLPGKWKPKMAGGIGGFIKVRQYDQIPVEICGHKVIGTVL
VGPTPVNIIGRNLLTQLGCTLNF
>55835_27 0.2
PQITLWQRPLVSIKIGGQIKEALLDTGADDTILEDINLPGKWKPKMAGGIGGFIKVRQYDHIPVEICGHKVIGTVL
VGPTPVNIIGRNLLTQLGCTLNF
>55835_29 0.2
PQITLWQRPLVSIKIGGQIKEALLDTGADDTILEDINLPGKWKPKLAGGIGGFIKVRQYDQIPVEICGHKVIGTVL
VGPTPVNIIGRNLLTQLGCTLNF
>55835_31 0.2
PQITLWQRPLVSIKIGGQIKEALLDTGADDTILEDINLPGKWKPKLAGGIGGFIKVRQYDHIPVEICGHKVIGTVL
VGPTPVNIIGRNLLTQLGCTLNF
>55835_49 0.2
PQITLWQRPIVSIKIGGQIKEALLDTGADDTILEDINLPGRWKPKMAGGIGGFIKVRQYDQIPVEICGHKVIGTVL
VGPTPVNIIGRNLLTQLGCTLNF
>55835_51 0.2
PQITLWQRPIVSIKIGGQIKEALLDTGADDTILEDINLPGRWKPKMAGGIGGFIKVRQYDHIPVEICGHKVIGTVL
VGPTPVNIIGRNLLTQLGCTLNF
>55835_53 0.2
PQITLWQRPIVSIKIGGQIKEALLDTGADDTILEDINLPGRWKPKLAGGIGGFIKVRQYDQIPVEICGHKVIGTVL
VGPTPVNIIGRNLLTQLGCTLNF
>55835_55 0.2
PQITLWQRPIVSIKIGGQIKEALLDTGADDTILEDINLPGRWKPKLAGGIGGFIKVRQYDHIPVEICGHKVIGTVL

VGPTPVNIIGRNLLTQLGCTLNF
>55835_57 0.2
PQITLWQRPIVSIKIGGQIKEALLDTGADDTILEDINLPGKWKPKMAGGIGGFIKVRQYDQIPVEICGHKVIGTVL
VGPTPVNIIGRNLLTQLGCTLNF
>55835_59 0.2
PQITLWQRPIVSIKIGGQIKEALLDTGADDTILEDINLPGKWKPKMAGGIGGFIKVRQYDHIPVEICGHKVIGTVL
VGPTPVNIIGRNLLTQLGCTLNF
>55835_61 0.2
PQITLWQRPIVSIKIGGQIKEALLDTGADDTILEDINLPGKWKPKLAGGIGGFIKVRQYDQIPVEICGHKVIGTVL
VGPTPVNIIGRNLLTQLGCTLNF
>55835_63 0.2
PQITLWQRPIVSIKIGGQIKEALLDTGADDTILEDINLPGKWKPKLAGGIGGFIKVRQYDHIPVEICGHKVIGTVLV
GPTPVNIIGRNLLTQLGCTLNF
>55835_16 0.2
PQITLWQRPLVSIKIGGQIKEALLDTGADDTILEDINLPGRWKPKMAGGIGGFIKVRQYDQIPVEICGHKAIGTVL
VGPTPVNIIGRNLLTQLGCTLNF
>55835_18 0.2
PQITLWQRPLVSIKIGGQIKEALLDTGADDTILEDINLPGRWKPKMAGGIGGFIKVRQYDHIPVEICGHKAIGTVL
VGPTPVNIIGRNLLTQLGCTLNF
>55835_20 0.2
PQITLWQRPLVSIKIGGQIKEALLDTGADDTILEDINLPGRWKPKLAGGIGGFIKVRQYDQIPVEICGHKAIGTVL
VGPTPVNIIGRNLLTQLGCTLNF
>55835_22 0.2
PQITLWQRPLVSIKIGGQIKEALLDTGADDTILEDINLPGRWKPKLAGGIGGFIKVRQYDHIPVEICGHKAIGTVL
VGPTPVNIIGRNLLTQLGCTLNF
>55835_24 0.2
PQITLWQRPLVSIKIGGQIKEALLDTGADDTILEDINLPGKWKPKMAGGIGGFIKVRQYDQIPVEICGHKAIGTVL
VGPTPVNIIGRNLLTQLGCTLNF
>55835_26 0.2
PQITLWQRPLVSIKIGGQIKEALLDTGADDTILEDINLPGKWKPKMAGGIGGFIKVRQYDHIPVEICGHKAIGTVL
VGPTPVNIIGRNLLTQLGCTLNF
>55835_28 0.2
PQITLWQRPLVSIKIGGQIKEALLDTGADDTILEDINLPGKWKPKLAGGIGGFIKVRQYDQIPVEICGHKAIGTVL
VGPTPVNIIGRNLLTQLGCTLNF
>55835_30 0.2
PQITLWQRPLVSIKIGGQIKEALLDTGADDTILEDINLPGKWKPKLAGGIGGFIKVRQYDHIPVEICGHKAIGTVL
VGPTPVNIIGRNLLTQLGCTLNF
>55835_48 0.2
PQITLWQRPIVSIKIGGQIKEALLDTGADDTILEDINLPGRWKPKMAGGIGGFIKVRQYDQIPVEICGHKAIGTVL
VGPTPVNIIGRNLLTQLGCTLNF
>55835_50 0.2
PQITLWQRPIVSIKIGGQIKEALLDTGADDTILEDINLPGRWKPKMAGGIGGFIKVRQYDHIPVEICGHKAIGTVL
VGPTPVNIIGRNLLTQLGCTLNF
>55835_52 0.2
PQITLWQRPIVSIKIGGQIKEALLDTGADDTILEDINLPGRWKPKLAGGIGGFIKVRQYDQIPVEICGHKAIGTVL
VGPTPVNIIGRNLLTQLGCTLNF
>55835_54 0.2
PQITLWQRPIVSIKIGGQIKEALLDTGADDTILEDINLPGRWKPKLAGGIGGFIKVRQYDHIPVEICGHKAIGTVL
VGPTPVNIIGRNLLTQLGCTLNF
>55835_56 0.2
PQITLWQRPIVSIKIGGQIKEALLDTGADDTILEDINLPGKWKPKMAGGIGGFIKVRQYDQIPVEICGHKAIGTVL
VGPTPVNIIGRNLLTQLGCTLNF
>55835_58 0.2
PQITLWQRPIVSIKIGGQIKEALLDTGADDTILEDINLPGKWKPKMAGGIGGFIKVRQYDHIPVEICGHKAIGTVL
VGPTPVNIIGRNLLTQLGCTLNF
>55835_60 0.2
PQITLWQRPIVSIKIGGQIKEALLDTGADDTILEDINLPGKWKPKLAGGIGGFIKVRQYDQIPVEICGHKAIGTVL
VGPTPVNIIGRNLLTQLGCTLNF
>55835_62 0.2
PQITLWQRPIVSIKIGGQIKEALLDTGADDTILEDINLPGKWKPKLAGGIGGFIKVRQYDHIPVEICGHKAIGTVLV
GPTPVNIIGRNLLTQLGCTLNF
>75096_0 0.2
PQITLWQRPLVTIKIGGQLKEALLDTGADDTILEEMNLPGRWKPKMVGGIGGFIKVRQYDEIPIEICGHKAIGTV

LVGPTPANIIGRNLLTQLGCTLNF
>41547_0 0.2
PQITLWQRPLVTIKIGGQLKEALLDTGADDTVLEEMNLPGRWKPKMIGGIGGFIKVRQYDQILVEICGHKAMGT
VLVGPTPVNIIGRNLLTQIGCTLNF
>44074_0 0.1
PQITLWQRPLVTVKIGGQLKEALLDTGADDTVLEEMDLPGRWKPKMIGGIGGFIKVRQYDQILIEICGHRAIGT
VLVGPTPVNIIGRNLLTQIGCTLNF
>44074_1 0.1
PQITLWQRPLVTVRIGGQLKEALLDTGADDTVLEEMDLPGRWKPKMIGGIGGFIKVRQYDQILIEICGHRAIGT
VLVGPTPVNIIGRNLLTQIGCTLNF

Sequences for the TPV-resistant ensemble
>103779_0 200.0
PQITLWQRPIIKVKIGGQLVEALLDTGADDTILEGINLPGRWKPKIVGGIGGFMKVKEYDQIPVEICGHKIISTVLV
GPTPVNIIGRNVMTQIGCTLNF
>103779_1 200.0
PQITLWQRPIIKVKIGGQLVEALLDTGADDTILEGINLPGRWKPKIVGGIGGFMKVKEYDQIPVEICGHKIISTVLV
GPTPVNVIGRNVMTQIGCTLNF
>103779_2 200.0
PQITLWQRPIIKVKIGGQLVEALLDTGADDTILEGINLPGRWKPKIVGGIGGFMKVKEYDQIPVEICGHKVISTVL
VGPTPVNIIGRNVMTQIGCTLNF
>103779_3 200.0
PQITLWQRPIIKVKIGGQLVEALLDTGADDTILEGINLPGRWKPKIVGGIGGFMKVKEYDQIPVEICGHKVISTVL
VGPTPVNVIGRNVMTQIGCTLNF
>103779_4 200.0
PQITLWQRPIIKVKIGGQLVEALLDTGADDTILEGIDLPGRWKPKIVGGIGGFMKVKEYDQIPVEICGHKIISTVLV
GPTPVNIIGRNVMTQIGCTLNF
>103779_5 200.0
PQITLWQRPIIKVKIGGQLVEALLDTGADDTILEGIDLPGRWKPKIVGGIGGFMKVKEYDQIPVEICGHKIISTVLV
GPTPVNVIGRNVMTQIGCTLNF
>103779_6 200.0
PQITLWQRPIIKVKIGGQLVEALLDTGADDTILEGIDLPGRWKPKIVGGIGGFMKVKEYDQIPVEICGHKVISTVL
VGPTPVNIIGRNVMTQIGCTLNF
>103779_7 200.0
PQITLWQRPIIKVKIGGQLVEALLDTGADDTILEGIDLPGRWKPKIVGGIGGFMKVKEYDQIPVEICGHKVISTVL
VGPTPVNVIGRNVMTQIGCTLNF
>103779_8 200.0
PQITLWQRPIIKVKIGGQLVEALLDTGADDTIFEGINLPGRWKPKIVGGIGGFMKVKEYDQIPVEICGHKIISTVL
VGPTPVNIIGRNVMTQIGCTLNF
>103779_9 200.0
PQITLWQRPIIKVKIGGQLVEALLDTGADDTIFEGINLPGRWKPKIVGGIGGFMKVKEYDQIPVEICGHKIISTVL
VGPTPVNVIGRNVMTQIGCTLNF
>103779_10 200.0
PQITLWQRPIIKVKIGGQLVEALLDTGADDTIFEGINLPGRWKPKIVGGIGGFMKVKEYDQIPVEICGHKVISTVL
VGPTPVNIIGRNVMTQIGCTLNF
>103779_11 200.0
PQITLWQRPIIKVKIGGQLVEALLDTGADDTIFEGINLPGRWKPKIVGGIGGFMKVKEYDQIPVEICGHKVISTVL
VGPTPVNVIGRNVMTQIGCTLNF
>103779_12 200.0
PQITLWQRPIIKVKIGGQLVEALLDTGADDTIFEGIDLPGRWKPKIVGGIGGFMKVKEYDQIPVEICGHKIISTVL
VGPTPVNIIGRNVMTQIGCTLNF
>103779_13 200.0
PQITLWQRPIIKVKIGGQLVEALLDTGADDTIFEGIDLPGRWKPKIVGGIGGFMKVKEYDQIPVEICGHKIISTVL
VGPTPVNVIGRNVMTQIGCTLNF
>103779_14 200.0
PQITLWQRPIIKVKIGGQLVEALLDTGADDTIFEGIDLPGRWKPKIVGGIGGFMKVKEYDQIPVEICGHKVISTVL
VGPTPVNIIGRNVMTQIGCTLNF
>103779_15 200.0
PQITLWQRPIIKVKIGGQLVEALLDTGADDTIFEGIDLPGRWKPKIVGGIGGFMKVKEYDQIPVEICGHKVISTVL
VGPTPVNVIGRNVMTQIGCTLNF
>147550_0 200.0
PQITLWQRPIIKVKIGGQLVEALLDTGADDTIFEGIDLPGRWKPKIVGGIGGFMKVKEYDQIPVEVCGHKVISTV
LVGPTPVNVIGRNVMTQIGCTLNF

>117063_1 200.0
PQITLWQRPVVTVKVAGQLREALLDTGADDTIFEEINLPGRWTPKIVGGIGGFMKVRQYDQIPIEICGHKVISTV
LVGPTPTNIIGGNLMTQIGCTLNF
>123049_0 200.0
PQITLWQRPIITIKIGGQLKDVLMDTGADDTVLEDIDLPGRWKPKLIGGIGGFVKVRQYDHVPVEVCGHKVTTT
VLVGPTPVDVIGRNLLTQIGCTLNF
>123049_1 200.0
PQITLWQRPIITIKIGGQLKDVLMDTGADDTVLEDIDLPGRWKPKLIGGIGGFVKVRQYDNVPVEVCGHKVTTT
VLVGPTPVDVIGRNLLTQIGCTLNF
>123049_2 200.0
PQITLWQRPIITIKIGGQLKDVLMDTGADDTVLEDIDLPGRWKPKLIGGIGGFVKVKQYDHVPVEVCGHKVTTT
VLVGPTPVDVIGRNLLTQIGCTLNF
>115065_2 200.0
PQITLWQRPVVTVKIAGQLKEALIDTGADDTIFFEMNLPGRWKPKLIGGIGGFVKVRQYDQIPIEICGHKVVGT
VLIGPTPTNVIGRNLLTQIGCTLNF
>115065_3 200.0
PQITLWQRPVVTVKIAGQLKEALIDTGADDTIFFEMDLPGRWKPKLIGGIGGFVKVRQYDQIPIEICGHKVVGT
VLIGPTPTNVIGRNLLTQIGCTLNF
>117119_0 200.0
PQITLWQRPIVTVKIAGQLKEALLDTGADDTVFEEMNLPGRWTPKLIGGIGGFVKVRQYDQIPIEICGHKVIGPV
LVGPTPANVIGRNLMSQIGCTLNF
>117119_1 200.0
PQITLWQRPIVTVKIAGQLKEALLDTGADDTVFEEMNLPGRWTPKLIGGIGGFVKVRQYDQVPIEICGHKVIGP
VLVGPTPANVIGRNLMSQIGCTLNF
>117119_2 200.0
PQITLWQRPIVTVRIAGQLKEALLDTGADDTVFEEMNLPGRWTPKLIGGIGGFVKVRQYDQIPIEICGHKVIGP
VLVGPTPANVIGRNLMSQIGCTLNF
>117119_3 200.0
PQITLWQRPIVTVRIAGQLKEALLDTGADDTVFEEMNLPGRWTPKLIGGIGGFVKVRQYDQVPIEICGHKVIGP
VLVGPTPANVIGRNLMSQIGCTLNF
>116529_1 200.0
PQITLWQRPIVTVKIGGQVKEALLDTGADDTVFEEMNLPGRWKPKMIGGIGGFVKVRQYDQIPIEICGHKVIGP
VLVGPTPLNVIGRNLMTQMGCTLNF
>116529_2 200.0
PQITLWQRPIVTVKIGGQVKEALLDTGADDTVFEEMNLPGRWKPKMIGGIGGFVKVRQYDQVPIEICGHKVIG
PVLVGPTPLNVIGRNLMTQIGCTLNF
>116529_3 200.0
PQITLWQRPIVTVKIGGQVKEALLDTGADDTVFEEMNLPGRWKPKMIGGIGGFVKVRQYDQVPIEICGHKVIG
PVLVGPTPLNVIGRNLMTQMGCTLNF
>116529_4 200.0
PQITLWQRPIVTVKIGGQVKEALLDTGADDTVFEEMNLPGRWKPRMIGGIGGFVKVRQYDQIPIEICGHKVIGP
VLVGPTPLNVIGRNLMTQIGCTLNF
>116529_5 200.0
PQITLWQRPIVTVKIGGQVKEALLDTGADDTVFEEMNLPGRWKPRMIGGIGGFVKVRQYDQIPIEICGHKVIGP
VLVGPTPLNVIGRNLMTQMGCTLNF
>116529_6 200.0
PQITLWQRPIVTVKIGGQVKEALLDTGADDTVFEEMNLPGRWKPRMIGGIGGFVKVRQYDQVPIEICGHKVIG
PVLVGPTPLNVIGRNLMTQIGCTLNF
>116529_7 200.0
PQITLWQRPIVTVKIGGQVKEALLDTGADDTVFEEMNLPGRWKPRMIGGIGGFVKVRQYDQVPIEICGHKVIG
PVLVGPTPLNVIGRNLMTQMGCTLNF
>116529_8 200.0
PQITLWQRPIVTVKIGGQVKEALLDTGADDTVFEEMNLPGRWTPKMIGGIGGFVKVRQYDQIPIEICGHKVIGP
VLVGPTPLNVIGRNLMTQIGCTLNF
>116529_9 200.0
PQITLWQRPIVTVKIGGQVKEALLDTGADDTVFEEMNLPGRWTPKMIGGIGGFVKVRQYDQIPIEICGHKVIGP
VLVGPTPLNVIGRNLMTQMGCTLNF
>116529_10 200.0
PQITLWQRPIVTVKIGGQVKEALLDTGADDTVFEEMNLPGRWTPKMIGGIGGFVKVRQYDQVPIEICGHKVIG
PVLVGPTPLNVIGRNLMTQIGCTLNF
>116529_11 200.0
PQITLWQRPIVTVKIGGQVKEALLDTGADDTVFEEMNLPGRWTPKMIGGIGGFVKVRQYDQVPIEICGHKVIG
PVLVGPTPLNVIGRNLMTQMGCTLNF

>116529_12 200.0
PQITLWQRPIVTVKIGGQVKEALLDTGADDTVFEEMNLPGRWTPRMIGGIGGFVKVRQYDQIPIEICGHKVIGP
VLVGPTPLNVIGRNLMTQIGCTLNF
>116529_13 200.0
PQITLWQRPIVTVKIGGQVKEALLDTGADDTVFEEMNLPGRWTPRMIGGIGGFVKVRQYDQIPIEICGHKVIGP
VLVGPTPLNVIGRNLMTQMGCTLNF
>116529_14 200.0
PQITLWQRPIVTVKIGGQVKEALLDTGADDTVFEEMNLPGRWTPRMIGGIGGFVKVRQYDQVPIEICGHKVIG
PVLVGPTPLNVIGRNLMTQIGCTLNF
>116529_15 200.0
PQITLWQRPIVTVKIGGQVKEALLDTGADDTVFEEMNLPGRWTPRMIGGIGGFVKVRQYDQVPIEICGHKVIG
PVLVGPTPLNVIGRNLMTQMGCTLNF
>116529_16 200.0
PQITLWQRPIVTVKVGGQVKEALLDTGADDTVFEEMNLPGRWKPKMIGGIGGFVKVRQYDQIPIEICGHKVIG
PVLVGPTPLNVIGRNLMTQIGCTLNF
>116529_17 200.0
PQITLWQRPIVTVKVGGQVKEALLDTGADDTVFEEMNLPGRWKPKMIGGIGGFVKVRQYDQIPIEICGHKVIG
PVLVGPTPLNVIGRNLMTQMGCTLNF
>116529_18 200.0
PQITLWQRPIVTVKVGGQVKEALLDTGADDTVFEEMNLPGRWKPKMIGGIGGFVKVRQYDQVPIEICGHKVIG
PVLVGPTPLNVIGRNLMTQIGCTLNF
>116529_19 200.0
PQITLWQRPIVTVKVGGQVKEALLDTGADDTVFEEMNLPGRWKPKMIGGIGGFVKVRQYDQVPIEICGHKVIG
PVLVGPTPLNVIGRNLMTQMGCTLNF
>116529_20 200.0
PQITLWQRPIVTVKVGGQVKEALLDTGADDTVFEEMNLPGRWKPRMIGGIGGFVKVRQYDQIPIEICGHKVIG
PVLVGPTPLNVIGRNLMTQIGCTLNF
>116529_21 200.0
PQITLWQRPIVTVKVGGQVKEALLDTGADDTVFEEMNLPGRWKPRMIGGIGGFVKVRQYDQIPIEICGHKVIG
PVLVGPTPLNVIGRNLMTQMGCTLNF
>116529_22 200.0
PQITLWQRPIVTVKVGGQVKEALLDTGADDTVFEEMNLPGRWKPRMIGGIGGFVKVRQYDQVPIEICGHKVI
GPVLVGPTPLNVIGRNLMTQIGCTLNF
>116529_23 200.0
PQITLWQRPIVTVKVGGQVKEALLDTGADDTVFEEMNLPGRWKPRMIGGIGGFVKVRQYDQVPIEICGHKVI
GPVLVGPTPLNVIGRNLMTQMGCTLNF
>116529_24 200.0
PQITLWQRPIVTVKVGGQVKEALLDTGADDTVFEEMNLPGRWTPKMIGGIGGFVKVRQYDQIPIEICGHKVIG
PVLVGPTPLNVIGRNLMTQIGCTLNF
>116529_25 200.0
PQITLWQRPIVTVKVGGQVKEALLDTGADDTVFEEMNLPGRWTPKMIGGIGGFVKVRQYDQIPIEICGHKVIG
PVLVGPTPLNVIGRNLMTQMGCTLNF
>116529_26 200.0
PQITLWQRPIVTVKVGGQVKEALLDTGADDTVFEEMNLPGRWTPKMIGGIGGFVKVRQYDQVPIEICGHKVIG
PVLVGPTPLNVIGRNLMTQIGCTLNF
>116529_27 200.0
PQITLWQRPIVTVKVGGQVKEALLDTGADDTVFEEMNLPGRWTPKMIGGIGGFVKVRQYDQVPIEICGHKVIG
PVLVGPTPLNVIGRNLMTQMGCTLNF
>116529_28 200.0
PQITLWQRPIVTVKVGGQVKEALLDTGADDTVFEEMNLPGRWTPRMIGGIGGFVKVRQYDQIPIEICGHKVIG
PVLVGPTPLNVIGRNLMTQIGCTLNF
>116529_29 200.0
PQITLWQRPIVTVKVGGQVKEALLDTGADDTVFEEMNLPGRWTPRMIGGIGGFVKVRQYDQIPIEICGHKVIG
PVLVGPTPLNVIGRNLMTQMGCTLNF
>116529_30 200.0
PQITLWQRPIVTVKVGGQVKEALLDTGADDTVFEEMNLPGRWTPRMIGGIGGFVKVRQYDQVPIEICGHKVIG
PVLVGPTPLNVIGRNLMTQIGCTLNF
>116529_31 200.0
PQITLWQRPIVTVKVGGQVKEALLDTGADDTVFEEMNLPGRWTPRMIGGIGGFVKVRQYDQVPIEICGHKVIG
PVLVGPTPLNVIGRNLMTQMGCTLNF
>113056_13 200.0
PQITLWQRPIVTVKIGGQLKEALLDTGADDTVFEEVNLPGRWKPKLIGGIGGFVKVREYEQVPIEVCGHKMMG
TVLIGPTPTNVIGRNMLTQLGCTLNF

>113056_14 200.0
PQITLWQRPIVTVKIGGQLKEALLDTGADDTVFEEVNLPGRWKPKLIGGIGGFVKVREYEQVPIEVCGHTMIGT
VLIGPTPTNVIGRNMLTQLGCTLNF
>113056_15 200.0
PQITLWQRPIVTVKIGGQLKEALLDTGADDTVFEEVNLPGRWKPKLIGGIGGFVKVREYEQVPIEVCGHTMMG
TVLIGPTPTNVIGRNMLTQLGCTLNF
>113056_16 200.0
PQITLWQRPIVTVKIGGQLKEALLDTGADDTVFDEMNLPGRWKPKLIGGIGGFVKVREYEQVPIEICGHKMIGT
VLIGPTPTNVIGRNMLTQLGCTLNF
>113056_17 200.0
PQITLWQRPIVTVKIGGQLKEALLDTGADDTVFDEMNLPGRWKPKLIGGIGGFVKVREYEQVPIEICGHKMMG
TVLIGPTPTNVIGRNMLTQLGCTLNF
>113056_18 200.0
PQITLWQRPIVTVKIGGQLKEALLDTGADDTVFDEMNLPGRWKPKLIGGIGGFVKVREYEQVPIEICGHTMIGT
VLIGPTPTNVIGRNMLTQLGCTLNF
>113056_19 200.0
PQITLWQRPIVTVKIGGQLKEALLDTGADDTVFDEMNLPGRWKPKLIGGIGGFVKVREYEQVPIEICGHTMMG
TVLIGPTPTNVIGRNMLTQLGCTLNF
>113056_20 200.0
PQITLWQRPIVTVKIGGQLKEALLDTGADDTVFDEMNLPGRWKPKLIGGIGGFVKVREYEQVPIEVCGHKMIG
TVLIGPTPTNVIGRNMLTQLGCTLNF
>113056_21 200.0
PQITLWQRPIVTVKIGGQLKEALLDTGADDTVFDEMNLPGRWKPKLIGGIGGFVKVREYEQVPIEVCGHKMM
GTVLIGPTPTNVIGRNMLTQLGCTLNF
>113056_22 200.0
PQITLWQRPIVTVKIGGQLKEALLDTGADDTVFDEMNLPGRWKPKLIGGIGGFVKVREYEQVPIEVCGHTMIG
TVLIGPTPTNVIGRNMLTQLGCTLNF
>113056_23 200.0
PQITLWQRPIVTVKIGGQLKEALLDTGADDTVFDEMNLPGRWKPKLIGGIGGFVKVREYEQVPIEVCGHTMM
GTVLIGPTPTNVIGRNMLTQLGCTLNF
>113056_24 200.0
PQITLWQRPIVTVKIGGQLKEALLDTGADDTVFDEVNLPGRWKPKLIGGIGGFVKVREYEQVPIEICGHKMIGT
VLIGPTPTNVIGRNMLTQLGCTLNF
>113056_25 200.0
PQITLWQRPIVTVKIGGQLKEALLDTGADDTVFDEVNLPGRWKPKLIGGIGGFVKVREYEQVPIEICGHKMMG
TVLIGPTPTNVIGRNMLTQLGCTLNF
>113056_26 200.0
PQITLWQRPIVTVKIGGQLKEALLDTGADDTVFDEVNLPGRWKPKLIGGIGGFVKVREYEQVPIEICGHTMIGT
VLIGPTPTNVIGRNMLTQLGCTLNF
>113056_27 200.0
PQITLWQRPIVTVKIGGQLKEALLDTGADDTVFDEVNLPGRWKPKLIGGIGGFVKVREYEQVPIEICGHTMMG
TVLIGPTPTNVIGRNMLTQLGCTLNF
>113056_28 200.0
PQITLWQRPIVTVKIGGQLKEALLDTGADDTVFDEVNLPGRWKPKLIGGIGGFVKVREYEQVPIEVCGHKMIG
TVLIGPTPTNVIGRNMLTQLGCTLNF
>113056_29 200.0
PQITLWQRPIVTVKIGGQLKEALLDTGADDTVFDEVNLPGRWKPKLIGGIGGFVKVREYEQVPIEVCGHKMM
GTVLIGPTPTNVIGRNMLTQLGCTLNF
>113056_30 200.0
PQITLWQRPIVTVKIGGQLKEALLDTGADDTVFDEVNLPGRWKPKLIGGIGGFVKVREYEQVPIEVCGHTMIG
TVLIGPTPTNVIGRNMLTQLGCTLNF
>113056_31 200.0
PQITLWQRPIVTVKIGGQLKEALLDTGADDTVFDEVNLPGRWKPKLIGGIGGFVKVREYEQVPIEVCGHTMMG
TVLIGPTPTNVIGRNMLTQLGCTLNF
>113056_32 200.0
PQITLWQRPVVTVKIGGQLKEALLDTGADDTVFEEMNLPGRWKPKLIGGIGGFVKVREYEQVPIEICGHKMIG
TVLIGPTPTNVIGRNMLTQLGCTLNF
>113056_33 200.0
PQITLWQRPVVTVKIGGQLKEALLDTGADDTVFEEMNLPGRWKPKLIGGIGGFVKVREYEQVPIEICGHKMM
GTVLIGPTPTNVIGRNMLTQLGCTLNF
>113056_34 200.0
PQITLWQRPVVTVKIGGQLKEALLDTGADDTVFEEMNLPGRWKPKLIGGIGGFVKVREYEQVPIEICGHTMIG
TVLIGPTPTNVIGRNMLTQLGCTLNF

>113056_35 200.0
PQITLWQRPVVTVKIGGQLKEALLDTGADDTVFEEMNLPGRWKPKLIGGIGGFVKVREYEQVPIEICGHTMM
GTVLIGPTPTNVIGRNMLTQLGCTLNF
>113056_36 200.0
PQITLWQRPVVTVKIGGQLKEALLDTGADDTVFEEMNLPGRWKPKLIGGIGGFVKVREYEQVPIEVCGHKMIG
TVLIGPTPTNVIGRNMLTQLGCTLNF
>113056_37 200.0
PQITLWQRPVVTVKIGGQLKEALLDTGADDTVFEEMNLPGRWKPKLIGGIGGFVKVREYEQVPIEVCGHKMM
GTVLIGPTPTNVIGRNMLTQLGCTLNF
>113056_38 200.0
PQITLWQRPVVTVKIGGQLKEALLDTGADDTVFEEMNLPGRWKPKLIGGIGGFVKVREYEQVPIEVCGHTMIG
TVLIGPTPTNVIGRNMLTQLGCTLNF
>113056_39 200.0
PQITLWQRPVVTVKIGGQLKEALLDTGADDTVFEEMNLPGRWKPKLIGGIGGFVKVREYEQVPIEVCGHTMM
GTVLIGPTPTNVIGRNMLTQLGCTLNF
>113056_40 200.0
PQITLWQRPVVTVKIGGQLKEALLDTGADDTVFEEVNLPGRWKPKLIGGIGGFVKVREYEQVPIEICGHKMIG
TVLIGPTPTNVIGRNMLTQLGCTLNF
>113056_41 200.0
PQITLWQRPVVTVKIGGQLKEALLDTGADDTVFEEVNLPGRWKPKLIGGIGGFVKVREYEQVPIEICGHKMMG
TVLIGPTPTNVIGRNMLTQLGCTLNF
>113056_42 200.0
PQITLWQRPVVTVKIGGQLKEALLDTGADDTVFEEVNLPGRWKPKLIGGIGGFVKVREYEQVPIEICGHTMIGT
VLIGPTPTNVIGRNMLTQLGCTLNF
>113056_43 200.0
PQITLWQRPVVTVKIGGQLKEALLDTGADDTVFEEVNLPGRWKPKLIGGIGGFVKVREYEQVPIEICGHTMMG
TVLIGPTPTNVIGRNMLTQLGCTLNF
>113056_44 200.0
PQITLWQRPVVTVKIGGQLKEALLDTGADDTVFEEVNLPGRWKPKLIGGIGGFVKVREYEQVPIEVCGHKMIG
TVLIGPTPTNVIGRNMLTQLGCTLNF
>113056_45 200.0
PQITLWQRPVVTVKIGGQLKEALLDTGADDTVFEEVNLPGRWKPKLIGGIGGFVKVREYEQVPIEVCGHKMM
GTVLIGPTPTNVIGRNMLTQLGCTLNF
>113056_46 200.0
PQITLWQRPVVTVKIGGQLKEALLDTGADDTVFEEVNLPGRWKPKLIGGIGGFVKVREYEQVPIEVCGHTMIG
TVLIGPTPTNVIGRNMLTQLGCTLNF
>113056_47 200.0
PQITLWQRPVVTVKIGGQLKEALLDTGADDTVFEEVNLPGRWKPKLIGGIGGFVKVREYEQVPIEVCGHTMM
GTVLIGPTPTNVIGRNMLTQLGCTLNF
>113056_48 200.0
PQITLWQRPVVTVKIGGQLKEALLDTGADDTVFDEMNLPGRWKPKLIGGIGGFVKVREYEQVPIEICGHKMIG
TVLIGPTPTNVIGRNMLTQLGCTLNF
>113056_49 200.0
PQITLWQRPVVTVKIGGQLKEALLDTGADDTVFDEMNLPGRWKPKLIGGIGGFVKVREYEQVPIEICGHKMM
GTVLIGPTPTNVIGRNMLTQLGCTLNF
>113056_50 200.0
PQITLWQRPVVTVKIGGQLKEALLDTGADDTVFDEMNLPGRWKPKLIGGIGGFVKVREYEQVPIEICGHTMIG
TVLIGPTPTNVIGRNMLTQLGCTLNF
>113056_51 200.0
PQITLWQRPVVTVKIGGQLKEALLDTGADDTVFDEMNLPGRWKPKLIGGIGGFVKVREYEQVPIEICGHTMM
GTVLIGPTPTNVIGRNMLTQLGCTLNF
>113056_52 200.0
PQITLWQRPVVTVKIGGQLKEALLDTGADDTVFDEMNLPGRWKPKLIGGIGGFVKVREYEQVPIEVCGHKMI
GTVLIGPTPTNVIGRNMLTQLGCTLNF
>117069_0 200.0
PQITLWQRPILTVKIAGQLTEALLDTGADDTIFQDINLPGRWTPKIVGGIGGLLRVRQYEQVPIEVCGHKIITTVLI
GPTPVNVLGRNLMTQLGCTLNF
>117069_1 200.0
PQITLWQRPILTVKVAGQLTEALLDTGADDTIFQDINLPGRWTPKIVGGIGGLLRVRQYEQVPIEVCGHKIITTVL
IGPTPVNVLGRNLMTQLGCTLNF

Sequences for the TPV-susceptible ensemble
>216623_0 0.4

PQITLWQRPFVTIKIGGQLKEALLDTGADDTVLEEMALPGKWKPKMIGGIGGFIKVGQYDQITIDICGHKATGT
VLIGPTPVNIIGRNLLTQIGCTLNF
>148079_0 0.5
PQITLWQRPIVTVRIEGQLIEALLDTGADDTVLEDIDLPGNWKPKMIGGIGGFVKVRQYDQIHIEICGKRAIGSV
VVGPTPANIIGRNILSQIGCTLNF
>115747_1 0.4
PQITLWQRPIVTIKIGGQTKEALLDTGADDTVLEEMNLPGRWKPKMIVGIGGFVKVRQYDQILVEICGHKAIGT
VLVGPTPANIIGRNLMTQIGCTLNF
>115755_0 0.2
PQITLWQRPIVTIKVGGQIMEALIDTGADDTVLEDINLPGRWKPKMIQGIGGLVKVRQYDQIPIEICGHEVMSTV
LVGPTPANIIGRNLLTQIGCTLNF
>115755_1 0.2
PQITLWQRPIVTIKVGGQIMEALIDTGADDTVLEDINLPGRWKPKMIQGIGGLVKVRQYDQIPIEICGHEVMSTV
LVGPTPANIIGRNLLTQMGCTLNF
>115755_2 0.2
PQITLWQRPIVTIKVGGQIMEALIDTGADDTVLEDINLPGRWKPKMIQGIGGLVKVRQYDQIPIEICGHEVMSTIL
VGPTPANIIGRNLLTQIGCTLNF
>115755_3 0.2
PQITLWQRPIVTIKVGGQIMEALIDTGADDTVLEDINLPGRWKPKMIQGIGGLVKVRQYDQIPIEICGHEVMSTIL
VGPTPANIIGRNLLTQMGCTLNF
>117023_0 0.4
PQITLWQRPIVTIKIGGQLREALLDTGADDTVLEDIELPGRWKPKMIVGVGGFAKVRQYDQVPIEICGHKVVGT
VLVGPTPANIIGRNLMTQLGCTLNF
>117023_1 0.4
PQITLWQRPIVTIKIGGQLREALLDTGADDTVLEDIELPGRWKPKMIVGVGGFAKVKQYDQVPIEICGHKVVGT
VLVGPTPANIIGRNLMTQLGCTLNF
>147548_0 0.2
PQITLWQRPIVTIKIGGQLKEALLDTGADDTVLEDINLPGRWKPKMIVGVGGFVKVRQYDQVAIEICGHKVIGT
VLVGPTPTNIIGRNLLTQIGCTLNF
>75088_0 0.4
PQITLWQRPIVTIKIGGQLKEALLDTGADDTVLQEMNLPGRWKPKMIVGVGGFSKVRQYDQVPIEICGHKVVG
SVLIGPTPANIIGRNLLTQLGCTLNF
>75088_1 0.4
PQITLWQRPIVTIKIGGQLKEALLDTGADDTVLQEMNLPGRWKPKIIVGVGGFSKVRQYDQVPIEICGHKVVGS
VLIGPTPANIIGRNLLTQLGCTLNF
>75088_2 0.4
PQITLWQRPIVTIKIGGQLKEALLDTGADDTVLQEMNLPGRWKPKVIVGVGGFSKVRQYDQVPIEICGHKVVG
SVLIGPTPANIIGRNLLTQLGCTLNF
>75088_3 0.4
PQITLWQRPIVTIKIGGQLKEALLDTGADDTVFQEMNLPGRWKPKMIVGVGGFSKVRQYDQVPIEICGHKVVG
SVLIGPTPANIIGRNLLTQLGCTLNF
>75088_4 0.4
PQITLWQRPIVTIKIGGQLKEALLDTGADDTVFQEMNLPGRWKPKIIVGVGGFSKVRQYDQVPIEICGHKVVGS
VLIGPTPANIIGRNLLTQLGCTLNF
>75088_5 0.4
PQITLWQRPIVTIKIGGQLKEALLDTGADDTVFQEMNLPGRWKPKVIVGVGGFSKVRQYDQVPIEICGHKVVG
SVLIGPTPANIIGRNLLTQLGCTLNF
>148003_4 0.5
PQITLWQRPLVTIKIGGQLIEALLDTGADDTVLEEMDLPGKWKPKMIGGIGGFIKVRQYEQVPIEICGYKAEGTV
LVGPTPVNIIGRNLLTQIGCTLNF
>148003_5 0.5
PQITLWQRPLVTIKIGGQLIEALLDTGADDTVLEEMDLPGKWKPKMIGGIGGFIKVRQYEQVPLEICGYKAEGT
VLVGPTPVNIIGRNLLTQIGCTLNF
>148003_6 0.5
PQITLWQRPLVTIKIGGQLIEALLDTGADDTVLEEIDLPGKWKPKMIGGIGGFIKVRQYEQVPIEICGYKAEGTVL
VGPTPVNIIGRNLLTQIGCTLNF
>235707_18 0.5
PQITLWQRPLVAIKIGGQLKEALLDTGADDTVLEEMNLPGRWKPKMIGGIGGFIKVGQYDQVAIEICGHKAIGT
VLVGPTPVNIIGRNLLTQLGCTLNF
>116519_0 0.3
PQIPLWQRPFVTIKIGGQIKEALLDTGADDTVFQEMDLPGRWKPKIIGGIGGFLKVRQYDQIPIEICGHKILGTVL
IGPTPVNIIGRGLMTQLGCTLNF
>116519_1 0.3

PQIPLWQRPFVTIKIGGHIKEALLDTGADDTVFQEMDLPGRWKPKIIGGIGGFLKVRQYDQIPIEICGHKILGTVL
IGPTPVNIIGRGLMTQLGCTLNF
>117057_0 0.4
PQIPLWQRPFVTIKIGGQIKEALLDTGADDTVFQEMDLPGRWKPKIIGGIGGFLKVRQYDQIPIEICGHKIIGTVLI
GPTPVNIIGRGLMTQLGCTLNF
>117057_2 0.4
PQIPLWQRPFVTIKIGGHIKEALLDTGADDTVFQEMDLPGRWKPKIIGGIGGFLKVRQYDQIPIEICGHKIIGTVLI
GPTPVNIIGRGLMTQLGCTLNF
>115747_0 0.4
PQITLWQRPIVTIKIGGQIKEALLDTGADDTVLEEMNLPGRWKPKMIVGIGGFVKVRQYDQILVEICGHKAIGTV
LVGPTPANIIGRNLMTQIGCTLNF
>117139_0 0.2
PQITLWQRPIVTIKIGGQLKEALLDTGADDTVLEEMDLPGRWKPKMIVGIGGLVKVRQYDQIPIEICGHKTIGTV
LIGPTPANIIGRNLMTQLGCTLNF
>216607_0 0.4
PQITLWQRPLVTIKIGGQLKEALLDTGADDTVLEEMNLPGRWKPRMIGGIGGFIKVGQYDQMPIEICGHKTEG
TVLVGPTPVNIIGRNLLTQIGCTLNF
>216607_1 0.4
PQITLWQRPLVTIKIGGQLKEALLDTGADDTVLEEMNLPGRWKPRMIGGIGGFIKVGQYDQMPIEICGHKTEG
TVLVGPTPVNIIGRNLLTQMGCTLNF
>216607_2 0.4
PQITLWQRPLVTIKIGGQLKEALLDTGADDTVLEEINLPGRWKPRMIGGIGGFIKVGQYDQMPIEICGHKTEGT
VLVGPTPVNIIGRNLLTQIGCTLNF
>216607_3 0.4
PQITLWQRPLVTIKIGGQLKEALLDTGADDTVLEEINLPGRWKPRMIGGIGGFIKVGQYDQMPIEICGHKTEGT
VLVGPTPVNIIGRNLLTQMGCTLNF
>81903_0 0.3
PQITLWQRPFVTIKVGGQLIEALLDTGADDTVLEEINLPGRWTPKIIGGVGGLIKVRQYEQVPIEICGHKVIGPVL
VGPTPVNIIGRNLMTQIGCTLNF
>81903_1 0.3
PQITLWQRPFVTIKVGGQLIEALLDTGADDTVLEEINLPGRWTPKIIGGVGGLLKVRQYEQVPIEICGHKVIGPV
LVGPTPVNIIGRNLMTQIGCTLNF
>109470_1 0.4
PQITLWQRPLVTIKIGGQLKEALLDTGADDTVLEEMNLPGRWKPKMIEGIGGFIKVRQYDEISIDICGHKAVGTV
LIGPTPVNIIGRNLLTQIGCTLNF
>109470_3 0.4
PQITLWQRPLVTIKIGGQLKEALLDTGADDTVLEEMSLPGRWKPKMIEGIGGFIKVRQYDEISIDICGHKAVGTV
LIGPTPVNIIGRNLLTQIGCTLNF
>81909_0 0.4
PQITLWQRPLVTIKIGGQLIEALLDTGADDTILEEINLPGRWKPKMIGGIGGFLKVRQYDQIPIEICGHKVIGTVLV
GPTPTNIIGRNIMTQIGCTLNF
>81909_1 0.4
PQITLWQRPLVTIKIGGQLIEALLDTGADDTILEEINLPGRWKPKMIGGIGGFLKVRQYDQIPIEICGHKVIGTVLV
GPTPTNIIGRNMMTQIGCTLNF
>116920_3 0.4
PQITLWQRPLVTIKIGGQLKEALLDTGADDTVLEEMNLPGRWKPRMIGGLGGFIKVRQYDQVPIEICGHKTVS
TVLIGPTPANIIGRNLLTQLGCTLNF
>235707_6 0.5
PQITLWQRPLVTIKIGGQLKEALLDTGADDTVLEEMNLPGRWKPKMIGGIGGFIKVGQYDQVAIEICGHKAIGT
VLVGPTPVNIIGRNLLTQLGCTLNF
>235707_7 0.5
PQITLWQRPLVTIKIGGQLKEALLDTGADDTVLEEMNLPGRWKPKMIGGIGGFIKVGQYDQVAIEICGHKAIGT
VLIGPTPVNIIGRNLLTQLGCTLNF
>235707_24 0.5
PQITLWQRPIVTIKIGGQLKEALLDTGADDTVLEEMNLPGRWKPKMIGGIGGFIKVGQYDQIAIEICGHKAIGTV
LVGPTPVNIIGRNLLTQLGCTLNF
>235707_25 0.5
PQITLWQRPIVTIKIGGQLKEALLDTGADDTVLEEMNLPGRWKPKMIGGIGGFIKVGQYDQIAIEICGHKAIGTV
LIGPTPVNIIGRNLLTQLGCTLNF
>235707_30 0.5
PQITLWQRPIVTIKIGGQLKEALLDTGADDTVLEEMNLPGRWKPKMIGGIGGFIKVGQYDQVAIEICGHKAIGTV
LVGPTPVNIIGRNLLTQLGCTLNF
>235707_31 0.5

PQITLWQRPIVTIKIGGQLKEALLDTGADDTVLEEMNLPGRWKPKMIGGIGGFIKVGQYDQVAIEICGHKAIGTV
LIGPTPVNIIGRNLLTQLGCTLNF
>235707_2 0.5
PQITLWQRPLVTIKIGGQLKEALLDTGADDTVLEEMNLPGRWKPKMIGGIGGFIKVGQYDQISIEICGHKAIGTV
LVGPTPVNIIGRNLLTQLGCTLNF
>257931_3 0.5
PQITLWQRPFVTVKIGGQLKEALLDTGADNTVIEDISLPGRWKPQMIGGIGGFVKVGQYEQVSVEICGHKAMG
TVLVGPTPVNIIGRNLLTKIGCTLNF
>257931_4 0.5
PQITLWQRPFVTVKIGGQLKEALLDTGADNTVIEDISLPGRWKPQIIGGIGGFVKVGQYEQVSVEICGHKAIGT
VLVGPTPVNIIGRNLLTQIGCTLNF
>257931_5 0.5
PQITLWQRPFVTVKIGGQLKEALLDTGADNTVIEDISLPGRWKPQIIGGIGGFVKVGQYEQVSVEICGHKAIGT
VLVGPTPVNIIGRNLLTKIGCTLNF
>257931_6 0.5
PQITLWQRPFVTVKIGGQLKEALLDTGADNTVIEDISLPGRWKPQIIGGIGGFVKVGQYEQVSVEICGHKAMG
TVLVGPTPVNIIGRNLLTQIGCTLNF
>257931_7 0.5
PQITLWQRPFVTVKIGGQLKEALLDTGADNTVIEDISLPGRWKPQIIGGIGGFVKVGQYEQVSVEICGHKAMG
TVLVGPTPVNIIGRNLLTKIGCTLNF
>257931_8 0.5
PQITLWQRPFVTVKIGGQLKEALIDTGADNTVIEDISLPGRWKPQMIGGIGGFVKVGQYEQVSVEICGHKAIGT
VLVGPTPVNIIGRNLLTQIGCTLNF
>257931_9 0.5
PQITLWQRPFVTVKIGGQLKEALIDTGADNTVIEDISLPGRWKPQMIGGIGGFVKVGQYEQVSVEICGHKAIGT
VLVGPTPVNIIGRNLLTKIGCTLNF
>257931_10 0.5
PQITLWQRPFVTVKIGGQLKEALIDTGADNTVIEDISLPGRWKPQMIGGIGGFVKVGQYEQVSVEICGHKAMG
TVLVGPTPVNIIGRNLLTQIGCTLNF
>187121_4 0.4
PQITLWQRPLVTIKIGGHLKGALLDTGADDTVLEEMSLPGRWKPKMIGGIGGFIKVKQYDQIPIEICGHKAIGTV
LVGPTPVNIIGRNLLTQIGCTLNF
>187121_5 0.4
PQITLWQRPLVTIKIGGHLKGALLDTGADDTVLEEMSLPGRWKPKMIGGIGGFIKVKQYDQIPIEICGHKAIGTV
LIGPTPVNIIGRNLLTQIGCTLNF
>187121_6 0.4
PQITLWQRPLVTIKIGGHLKGALLDTGADDTVLEEMSLPGRWRPKMIGGIGGFIKVKQYDQIPIEICGHKAIGTV
LVGPTPVNIIGRNLLTQIGCTLNF
>187121_7 0.4
PQITLWQRPLVTIKIGGHLKGALLDTGADDTVLEEMSLPGRWRPKMIGGIGGFIKVKQYDQIPIEICGHKAIGTV
LIGPTPVNIIGRNLLTQIGCTLNF
>187121_12 0.4
PQITLWQRPLVTIKVGGHLKGALLDTGADDTVLEEMSLPGRWKPKMIGGIGGFIKVKQYDQIPIEICGHKAIGT
VLVGPTPVNIIGRNLLTQIGCTLNF
>187121_13 0.4
PQITLWQRPLVTIKVGGHLKGALLDTGADDTVLEEMSLPGRWKPKMIGGIGGFIKVKQYDQIPIEICGHKAIGT
VLIGPTPVNIIGRNLLTQIGCTLNF
>187121_14 0.4
PQITLWQRPLVTIKVGGHLKGALLDTGADDTVLEEMSLPGRWRPKMIGGIGGFIKVKQYDQIPIEICGHKAIGT
VLVGPTPVNIIGRNLLTQIGCTLNF
>187121_15 0.4
PQITLWQRPLVTIKVGGHLKGALLDTGADDTVLEEMSLPGRWRPKMIGGIGGFIKVKQYDQIPIEICGHKAIGT
VLIGPTPVNIIGRNLLTQIGCTLNF
>116920_1 0.4
PQITLWQRPLVTIKIGGQLKEALLDTGADDTVLEEMNLPGRWKPRMIGGLGGFIKVRQYDQVPIEICGHKIVST
VLIGPTPANIIGRNLLTQLGCTLNF
>143459_0 0.2
PQITLWQRPIVTIKIGGQLKEALLDTGADNTVFEEMNLPGRWKPKLIGGIGGLLKVREYDQIPIEICGHKTISTVL
IGPTPVNIIGRDLLTQIGCTLNF
>257929_0 0.4
PQITLWQRPLVTVKVGGQLKEALLDTGADDTVLEEMNLQGRWKPKMIGGIGGFIKVKQYDQIPVEICGHKAIG
TVLVGPTPVNIIGRNLLTQIGCTLNF
>81801_0 0.3

PQITLWQRPLVTIKIGGQLREALLDTGADDTVLEDIDLPGRWKPKMIGGIGGLIKVRQYDQIPIEICGKKTVGTV
LIGPTPANIIGRNLLTQIGCTLNF
>116920_2 0.4
PQITLWQRPLVTIKIGGQLKEALLDTGADDTVLEEMNLPGRWKPRMIGGLGGFIKVRQYDQVPIEICGHKTVG
TVLIGPTPANIIGRNLLTQLGCTLNF
>143336_2 0.5
PQITLWQRPLVTIKIGGQLKEALLDTGADDTVLEEMNLPGRWKPKMIGGIGGFIKVRQYDQVPIEICGHKVICT
VLIGPTPVNIIGRNLMTQLGCTLNF
>143336_6 0.5
PQITLWQRPLVTIKIGGQLKEALLDTGADDTVLEEMNLPGRWKPKMIGGLGGFIKVRQYDQVPIEICGHKVICT
VLIGPTPVNIIGRNLMTQLGCTLNF
>143336_10 0.5
PQITLWQRPLVTIKIGGQLKEALLDTGADDTILEEMNLPGRWKPKMIGGIGGFIKVRQYDQVPIEICGHKVICTV
LIGPTPVNIIGRNLMTQLGCTLNF
>143336_14 0.5
PQITLWQRPLVTIKIGGQLKEALLDTGADDTILEEMNLPGRWKPKMIGGLGGFIKVRQYDQVPIEICGHKVICTV
LIGPTPVNIIGRNLMTQLGCTLNF
>143336_18 0.5
PQITLWQRPLVTIKIGGQLKEAILDTGADDTVLEEMNLPGRWKPKMIGGIGGFIKVRQYDQVPIEICGHKVICTV
LIGPTPVNIIGRNLMTQLGCTLNF
>143336_22 0.5
PQITLWQRPLVTIKIGGQLKEAILDTGADDTVLEEMNLPGRWKPKMIGGLGGFIKVRQYDQVPIEICGHKVICT
VLIGPTPVNIIGRNLMTQLGCTLNF
>143336_26 0.5
PQITLWQRPLVTIKIGGQLKEAILDTGADDTILEEMNLPGRWKPKMIGGIGGFIKVRQYDQVPIEICGHKVICTVL
IGPTPVNIIGRNLMTQLGCTLNF
>143336_0 0.5
PQITLWQRPLVTIKIGGQLKEALLDTGADDTVLEEMNLPGRWKPKMIGGIGGFIKVRQYDQVPIEICGHKAICT
VLIGPTPVNIIGRNLMTQLGCTLNF
>143336_4 0.5
PQITLWQRPLVTIKIGGQLKEALLDTGADDTVLEEMNLPGRWKPKMIGGLGGFIKVRQYDQVPIEICGHKAICT
VLIGPTPVNIIGRNLMTQLGCTLNF
>143336_8 0.5
PQITLWQRPLVTIKIGGQLKEALLDTGADDTILEEMNLPGRWKPKMIGGIGGFIKVRQYDQVPIEICGHKAICTV
LIGPTPVNIIGRNLMTQLGCTLNF
>143336_12 0.5
PQITLWQRPLVTIKIGGQLKEALLDTGADDTILEEMNLPGRWKPKMIGGLGGFIKVRQYDQVPIEICGHKAICTV
LIGPTPVNIIGRNLMTQLGCTLNF
>143336_16 0.5
PQITLWQRPLVTIKIGGQLKEAILDTGADDTVLEEMNLPGRWKPKMIGGIGGFIKVRQYDQVPIEICGHKAICTV
LIGPTPVNIIGRNLMTQLGCTLNF
>143336_20 0.5
PQITLWQRPLVTIKIGGQLKEAILDTGADDTVLEEMNLPGRWKPKMIGGLGGFIKVRQYDQVPIEICGHKAICT
VLIGPTPVNIIGRNLMTQLGCTLNF
>143336_24 0.5
PQITLWQRPLVTIKIGGQLKEAILDTGADDTILEEMNLPGRWKPKMIGGIGGFIKVRQYDQVPIEICGHKAICTVL
IGPTPVNIIGRNLMTQLGCTLNF
>143336_28 0.5
PQITLWQRPLVTIKIGGQLKEAILDTGADDTILEEMNLPGRWKPKMIGGLGGFIKVRQYDQVPIEICGHKAICTV
LIGPTPVNIIGRNLMTQLGCTLNF
>109484_0 0.4
PQITLWQRPLVTIKIGGQLKEALLDTGADDTVLEEMNLPGRWKPKMIGGIGGFIKVRQYDQIPIEICGHKATGT
VLIGPTPVNIIGRNLLTQLGCTLNF
>198533_0 0.5
PQITLWQRPLVTIKIGGQLKEALLDTGADDTVLEEMELPGRWKPKMIGGIGGFIKVRQYDQIPIEICGHTARGT
VLVGPTPVNIIGRNLLTQIGCTLNF
>216639_0 0.5
PQITLWQRPIVTIKIGGQIKEALLDTGADDTVLEEITLPGKWKPKMIGGIGGFIKVGQYDQMPMEICGHKVIGTV
LVGPTPVNIIGRNLLTQLGCTLNF
>216639_1 0.5
PQITLWQRPIVTIKIGGQIKEALLDTGADDTVLEEITLPGKWKPRMIGGIGGFIKVGQYDQMPMEICGHKVIGTV
LVGPTPVNIIGRNLLTQLGCTLNF
>216639_2 0.5

PQITLWQRPVVTIKIGGQIKEALLDTGADDTVLEEITLPGKWKPKMIGGIGGFIKVGQYDQMPMEICGHKVIGT
VLVGPTPVNIIGRNLLTQLGCTLNF
>216639_3 0.5
PQITLWQRPVVTIKIGGQIKEALLDTGADDTVLEEITLPGKWKPRMIGGIGGFIKVGQYDQMPMEICGHKVIGT
VLVGPTPVNIIGRNLLTQLGCTLNF
>145683_0 0.5
PQITLWQRPIVTIKIGGQLKEALLDTGADDTVLEEMNLPGRWKPKMIGGIGGFIKVRQYDQIAIEICGHKAIGTV
LVGPTPVNIIGRNLLTQLGCTLNF
>145683_1 0.5
PQITLWQRPIVTIKIGGQLKEALLDTGADDTVLEEINLPGRWKPKMIGGIGGFIKVRQYDQIAIEICGHKAIGTVL
VGPTPVNIIGRNLLTQLGCTLNF
>109470_0 0.4
PQITLWQRPLVTIKIGGQLKEALLDTGADDTVLEEMNLPGRWKPKMIGGIGGFIKVRQYDEISIDICGHKAVGT
VLIGPTPVNIIGRNLLTQIGCTLNF
>109470_2 0.4
PQITLWQRPLVTIKIGGQLKEALLDTGADDTVLEEMSLPGRWKPKMIGGIGGFIKVRQYDEISIDICGHKAVGTV
LIGPTPVNIIGRNLLTQIGCTLNF
>148059_0 0.4
PQITLWQRPLVTIKIGGQLKEALLDTGADDTVLEEMNLPGRWKPKMIGGIGGFIKVKQYDQIQIEICGHKAIGTV
LVGPTPVNIIGRNLLTQIGCTLNF
>148111_0 0.4
PQITLWQRPLVTVKIGGQLKEALLDTGADDTVLEEMNLPGRWKPRMIGGIGGFIKVRQYDQISMEICGHKAIG
TVLIGPTPVNIIGRNLLTQIGCTLNF
>148111_1 0.4
PQITLWQRPLVTVKIGGQLKEALLDTGADDTVLEEMNLPGRWKPRMIGGIGGFIKVRQYDQVSMEICGHKAIG
TVLIGPTPVNIIGRNLLTQIGCTLNF
>172116_0 0.3
PQITLWQRPVVTIKIGGQLKEALLDTGADDTVLEDMDLPGRWKPKIIGGIGGFIKVRQYDQIPIEICGHKVISTVL
IGPTPVNIIGRNLMTQLGCTLNF
>172116_1 0.3
PQITLWQRPVVTIKIGGQLKEALLDTGADDTVLEDMDLPGRWKPKIIGGIGGFIRVRQYDQIPIEICGHKVISTVL
IGPTPVNIIGRNLMTQLGCTLNF
>116920_0 0.4
PQITLWQRPLVTIKIGGQLKEALLDTGADDTVLEEMNLPGRWKPRMIGGLGGFIKVRQYDQVPIEICGHKIVGT
VLIGPTPANIIGRNLLTQLGCTLNF
>75096_0 0.4
PQITLWQRPLVTIKIGGQLKEALLDTGADDTILEEMNLPGRWKPKMVGGIGGFIKVRQYDEIPIEICGHKAIGTV
LVGPTPANIIGRNLLTQLGCTLNF
>81869_0 0.4
PQITLWQRPIVTIKIGGQLKEALIDTGADDTVLEEMNLPGRWKPKMIGGIGGFIKVRQYDQIPVEICGHKALGTV
LIGPTPANIIGRNLLTQIGCTLNF
>109408_0 0.5
PQITLWQRPLVTVKIGGQLKEALLDTGADNTVLEEMNLPGRWKPKMIGGIGGFIKVRQYDQIPIEICGHRTIGT
VLIGPTPVNIIGRDLLTQIGCTLNF
>109408_1 0.5
PQITLWQRPIVTVKIGGQLKEALLDTGADNTVLEEMNLPGRWKPKMIGGIGGFIKVRQYDQIPIEICGHRTIGTV
LIGPTPVNIIGRDLLTQIGCTLNF
